# Supplementary material for: A pangenome reference of 36 Chinese populations
Source: Nature. 2023 Jun 14;619(7968):112–21. doi: 10.1038/s41586-023-06173-7 (PMC10322713; doi:10.1038/s41586-023-06173-7)
Supplement: Supplementary file 1 — This file contains the current complete list of members of the CPC and Supplementary Methods, References, Tables 1–26 and Figs. 1–22 (see contents page for details). [file 41586_2023_6173_MOESM1_ESM.pdf]

nature portfolio

https://doi.org/10.1038/s41586-023-06173-7

Supplementary information

A pangenome reference of 36 Chinese populations

In the format provided by the authors and unedited

SUPPLEMENTARY INFORMATION

A Pangenome Reference of 36 Chinese populations

Correspondence and requests for materials should be addressed to Dr. Shuhua Xu (xushua@fudan.edu.cn)

Contents

S1. Chinese Pangenome Consortium (CPC) Members 3
S2. Populations and Samples 5
S3. Ethical statement 5
S4. Establishment of Epstein-Barr Virus (EBV) immortalized B cell lines 6
S5. DNA Extraction 6
S6. DNA Quantification & Qualification 8
S7. Library construction and sequencing 8
S8. Hi-C sequencing 12
S9. High-Fidelity (HiFi) sequencing 13
S10. Oxford Nanopore (ONT) Sequencing 14
S11. Genome Assembly and Quality Control 15
S12. Complex structural variation detection 16
S13. Evaluate alignment quality of short-raeds using CPC reference 16
S14. Pangenome growth 17
S15. Statistics on variants 17
S16. Alignment, variant identification and filtering 17
S17. Population Genetic Analayses 18
S18. Functional annotation and enrichment analysis of genes 19
S19. Archaic introgression segments detection and functional annotation 20
S20. Testing for neutrality by estimating Tajima's *D* 21
S21. References 22
S22. Supplementary Tables 24
Supplementary Table 1 Information of the samples for sequencing in CPC 24
Supplementary Table 2 Summary statistics of the 116 CPC genome assemblies 25
Supplementary Table 3 The length (Mb) of different types of the sequences unaligned to GRCh38 reference genome per assembly 26
Supplementary Table 4 CPC-specific-CNV-related genes compared to HPRC assemblies 28
Supplementary Table 5 Functional enrichment of the novel duplicated genes in the CPC assembly set 37
Supplementary Table 6 Overlapped CNV-related-genes between CPC and HPRC 38
Supplementary Table 7 A comparison of variant calling based on linear genome reference and graph genome reference 40
Supplementary Table 8 Compare the size of HPRC graph reference and CPC graph reference. These include raw graph and simplified graph for short-reads mapping. 41

Supplementary Table 9 The number of small variants whether can be found in the gnomAD v0.1.8 in different regions defined in GIAB 3.0 42
Supplementary Table 10 Functional enrichment of genes affected by the novel SVs (≥ 1kb) in the CPC assembly set. 43
Supplementary Table 11 Functional enrichment of genes affected by the CPC-specific SV hotspots. 45
Supplementary Table 12 Human alpha globin genes haplotypes of CPC and HPRC haploid assemblies 46
Supplementary Table 13 Copy number of *RASA4(B)* genes in CPC samples 47
Supplementary Table 14 Counts of haplotypes with different *RASA4(B)* copy number in different populations 48
Supplementary Table 15 Frequnecy distribution of *RASA4(B)* copy number in different populations 49
Supplementary Table 16 Enrichment of novel SVs around the GWAS loci. 50
Supplementary Table 17 Genes and Frequencies of CDS Regions Covered by CPC Archaic Introgression Segments 51
Supplementary Table 18 Functional enrichment of genes with CDS intersecting with CPC-AIS in at least 5 samples 56
Supplementary Table 19 Functional enrichment of genes with CDS intersecting with CPC-AIS in at least 1 sample. 58
Supplementary Table 20 Functional enrichment of genes with CDS intersecting with CPC-AIS in at least 10 samples 60
Supplementary Table 21 Genes and Frequencies of CDS Regions Covered by CPC-specific Archaic Introgression Segments. 61
Supplementary Table 22 Functional enrichment of genes with CDS intersecting with CPC-specific-AIS 63
Supplementary Table 23 GeneAnalytics annotation of genes with CDS intersecting with CPC-specific-AIS 64
Supplementary Table 24 Population-specific archaic introgressed segments in CPC (absent in HPRC) with size >150 kb. 65
Supplementary Table 25 Summary of the population-specific archaic introgressed segments in CPC (absent in HPRC). 68
Supplementary Table 26 CPC-specific SV-related genes affected by the population-specific archaic introgressed segments in CPC (absent in HPRC). 69
S23. Supplementary Figures 76
Supplementary Fig. 1 | Geographical distribution of CPC samples. 76
Supplementary Fig. 2 | Detailed PCA of CPC samples in the context of East Asian populations. 77
Supplementary Fig. 3 | Evaluation of assembly correction of CPC genome assemblies. 78
Supplementary Fig. 4 | Evaluation of large-scale assembly errors of CPC genome assemblies. 79
Supplementary Fig. 5 | IGV visualization of small-scale assembly error cases in HIFI032069D-H1. 80
Supplementary Fig. 6 | Cumulative number of SVs by adding CPC samples one by one. 81

Supplementary Fig. 7 | The count of CSVs in each sample. 82
Supplementary Fig. 8 | The number of CSVs for each CSV type in each sample. 83
Supplementary Fig. 9 | Tajima's D estimated for the CNV genes identified in the CPC assemblies..84
Supplementary Fig. 10 | Number of identified small variants and SVs inferred from the pangenome graph. 85
Supplementary Fig. 11 | Statistics of CPC graph reference with different filtering standards. 86
Supplementary Fig. 12 | Mapping rate and perfect rate of 10 East Asian samples using CPC graph reference with different complexity. 87
Supplementary Fig. 13 | The correlation between the diversity of graph reference and the proportion of perfect alignment..88
Supplementary Fig. 14 | The difference of perfect alignment ratio between CPC reference and HPRC reference when processing East Asian samples and African samples. 89
Supplementary Fig. 15 | Comparison of SV distribution between CPC and HPRC reference Pangenomes...90
Supplementary Fig. 16 | Tajima's D estimated for the novel SVs identified in the CPC assemblies..91
Supplementary Fig. 17 | Functional enrichment of 2617 genes in CDS where CPC-AIS are located and observed in at least 5 samples. 92
Supplementary Fig. 18 | Functional enrichment of genes in CDS where AIS are located and observed in at least 1 sample. 93
Supplementary Fig. 19 | Functional enrichment of genes in CDS where AIS are located and observed in at least 10 samples. 94
Supplementary Fig. 20 | Total length of the archaic introgression segments detected in CPC compared with that in HPRC. 95
Supplementary Fig. 21 | Accumulative length of the archaic introgression segments in the East Asian populations studied in CPC...96
Supplementary Fig. 22 | Functional enrichment of 1211 genes in CDS where CPC-AIS-specific are located. 97

S1. Chinese Pangenome Consortium (CPC) Members

Yang Gao[1,2,3,4,§], Xiaofei Yang[5,6,7§], Hao Chen[3,§], Xinjiang Tan[3,§], Zhaoqing Yang[8,§], Lian Deng[1,§] , Baonan Wang[2], Shuang Kong[2], Songyang Li[2], Yuhang Cui[2], Chang Lei[1], Yimin Wang[3], Yuwen Pan[3], Sen Ma[3], Hao Sun[7], Fengxiao Bu[9] Guanglin He[9] Xiaohan Zhao[2], Yingbing Shi[1], Ziyi Yang[1], Chuangxue Mao[3], Yang Wu[9] Shaohua Fan[1], Juncheng Dai[10,11], Xingming Zhao[12], Jinchen Li[13,14], Chao Chen[14], Jian Yang[15,16], Chaochun Wei[17], Dongdong Wu[18], Shaoyuan Wu[19], Xin Jin[20], Xia Shen[1,21], Binyin Shi[22], Li Jin[1,2], Huijun Yuan[9], Zhibin Hu[10,11], Yan Lu[1]*, Jiayou Chu[8]*, Kai Ye[6,23,24]*, Shuhua Xu[1,2,4,16,25,26]*

[§]These authors contributed equally to this work.
*Correspondence: xushua@fudan.edu.cn (S.X.) , kaiye@xjtu.edu.cn (K.Y.), chujy@imbcams.com.cn (J.C.), lueyan@fudan.edu.cn (Y.L.)

[1]State Key Laboratory of Genetic Engineering, Center for Evolutionary Biology, Collaborative Innovation Center for Genetics and Development, School of Life Sciences, Fudan University, Shanghai 200438, China;
[2]Human Phenome Institute, Zhangjiang Fudan International Innovation Center, and Ministry of Education Key Laboratory of Contemporary Anthropology, Fudan University, Shanghai 201203, China
[3]Key Laboratory of Computational Biology, Shanghai Institute of Nutrition and Health, University of Chinese Academy of Sciences, Chinese Academy of Sciences, Shanghai 200031, China
[4]School of Life Science and Technology, ShanghaiTech University, Shanghai 201210, China
[5]School of Computer Science and Technology, Faculty of Electronic and Information Engineering, Xi'an Jiaotong University, Xi'an, Shaanxi, 710049, China
[6]MOE Key Lab for Intelligent Networks & Networks Security, Faculty of Electronic and Information Engineering, Xi'an Jiaotong University, Xi'an, Shaanxi, 710049, China
[7]Genome Institute, the First Affiliated Hospital of Xi'an Jiaotong University, Xi'an, Shaanxi, 710049, China
[8]Department of Medical Genetics, Institute of Medical Biology, Chinese Academy of Medical Sciences, Kunming 650118, China
[9]Institute of Rare Diseases, West China Hospital of Sichuan University, Sichuan University, Chengdu, 610041, China
[10]State Key Laboratory of Reproductive Medicine, Nanjing Medical University, Nanjing 211166, Jiangsu, China;
[11]Jiangsu Key Lab of Cancer Biomarkers, Prevention and Treatment, Collaborative Innovation Center for Cancer Personalized Medicine, Center for Global Health, School of Public Health, Nanjing Medical University, Nanjing 211166, Jiangsu, China
[12]Institute of Science and Technology for Brain-Inspired Intelligence, Ministry of Education Key (MOE) Laboratory of Computational Neuroscience and Brain-Inspired Intelligence, MOE Frontiers Center for Brain Science Fudan University, Shanghai, China
[13]Bioinformatics Center & National Clinical Research Centre for Geriatric Disorders, Xiangya Hospital, Central South University, Changsha, Hunan, China
[14]Center for Medical Genetics & Hunan Key Laboratory of Medical Genetics, School of Life Sciences, and Department of Psychiatry, The Second Xiangya Hospital, Central South University, Changsha, China
[15]School of Life Sciences, Westlake University, Hangzhou, Zhejiang 310024, China
[16]Westlake Laboratory of Life Sciences and Biomedicine, Hangzhou, Zhejiang 310024, China
[17]Department of Bioinformatics and Biostatistics, School of Life Sciences and Biotechnology, Shanghai Jiao Tong University, Shanghai 200240, China
[18]State Key Laboratory of Genetic Resources and Evolution, Kunming Institute of Zoology, Chinese Academy of Sciences, Kunming 650223, China
[19]Jiangsu Key Laboratory of Phylogenomics & Comparative Genomics, International Joint Center of Genomics of Jiangsu Province School of Life Sciences, Jiangsu Normal University, Xuzhou 211116, Jiangsu, China
[20]BGI-Research, Shenzhen 518083, China
[21]Center for Intelligent Medicine Research, Greater Bay Area Institute of Precision Medicine (Guangzhou), Fudan University, Guangzhou, 511458, China
[22]Department of Endocrinology, The First Affiliated Hospital of Xi'an Jiaotong University, Xi'an, China

[23]School of Automation Science and Engineering, Faculty of Electronic and Information Engineering, Xi'an Jiaotong University, Xi'an, Shaanxi, 710049, China
[24]School of Life Science and Technology, Xi'an Jiaotong University, Xi'an, Shaanxi, 710049, China
[25]Department of Liver Surgery and Transplantation Liver Cancer Institute, Zhongshan Hospital, Fudan University, Shanghai 200032, China
[26]Center for Excellence in Animal Evolution and Genetics, Chinese Academy of Sciences, Kunming 650223, China

S2. Populations and Samples

For the Phase I of CPC, we selected 68 samples from 731 individuals with genomes deep-sequenced using next-generation sequencing. It turned out that we applied a procedure similar to that of HPRC to select the representative samples of a subpopulation[1]. After quality control of the HiFi data, eventually 58 samples were retained for all the further analyses in this study. These include Achang (n=1), Bai (n=1), Blang (n=2), Bouyei (n=1), Chosen (n=3), Daur (n=2), Deang (n=2), Dong (n=2), Drung (n=1), Evenki (n=1), Hezhen (n=1), Hui (n=1), Jingpo (n=1), Jino (n=1), Kazakh (n=1), Khatso (n=1), Kyrgyz (n=1), Kinh (n=2), Lisu (n=1), Man (n=1), Miao (n=2), Mongol (n=3), Mosuo (n=3), Naxi (n=1), Oroqen (n=1), Qiang (n=3), Salar (n=2), She (n=3), Tu (n=2), Tujia (n=3), Uyghur (n=1), Wa (n=2), Yao (n=1), Yi (n=1), Yugur (n=2), and Zhuang (n=1).

S3. Ethical statement

Informed consent was obtained from all individual participants included in the study. The personal identifiers of all samples, if any existed, were stripped off before sequencing and analysis. All procedures were in accordance with the ethical standards of the Responsible Committee on Human Experimentation and the 1964 Helsinki Declaration, its later amendments (2000) or comparable ethical standards. The research content and procedures performed in studies involving human participants were approved by the Biomedical Research Ethics Committee of Shanghai Institutes for Biological Sciences (No. ER-SIBS-261408), the

Biomedical Research Ethics Committee of Kunming Institute of Zoology, Chinese Academy of Sciences (No. SMKX-20180715-154), the Biomedical Research Ethics Committee of the First Affiliated Hospital of Xi'an Jiaotong University (No. XJTU1AF2021LSK-051).

S4. Establishment of Epstein-Barr Virus (EBV) immortalized B cell lines

Blood collection and lymphocyte separation

For each participant, 3ml peripheral blood were collected in vacutainer tubes containing EDTA. The blood was diluted with 3mL RPMI1640 and spun at 1200G for 20min at room temperature (24°C). The opaque lymphocyte layer was collected down to the erythrocyte pellet and transferred to another 15-ml centrifuge tube. The cells were washed with centrifugation in 7mL RPMI1640 at 800G for 8min and then the clear top plasma layer was discarded.

Immortalization of lymphocytes

Lymphocytes were shaken at 120RPM for 1 hour with 4 ml EBV stock from Epstein-Barr virus (EBV)-transformed B95-8 marmoset cell line. The cells were grown at 37°C in RPMI1640 medium, supplemented with 20% fetal bovine serum and cyclosporine A with a final concentration of 2μg/ml. Every 4 days of incubation, the cultures were examined and photographed by microscopy. The cell aggregates of proliferative lymphoblast cells indicated successful transformation. These LCLs were limited in their growth to minimize genetic changes that may occur during extended cell culture. The LCLs were also examined for mycoplasma contamination using a polymerase chain reaction-based method and a culture assay; the results showed no infestation. The whole procedure was done in the Immortalize Cell Bank of Chinese Ethnics Groups hosted in the Institute of Medical Biology, CAMS.

S5. DNA Extraction

DNA preparation for NGS

Genomic DNA was extracted from the blood samples using QIAGEN DNeasy Blood & Tissue Kit. DNA concentrations were measured with the NanoDrop 2000 (Thermo Fisher Scientific), and sheared with Covaris S220 Sonicator (Covaris) to target of 500–600 base pairs (bp) average size. Fragmented DNA was purified using Sample Purification Beads (Illumina). Adapter-ligated libraries were prepared with the TruSeq Nano DNA Sample Prep Kits (Illumina) according to Illumina-provided protocol. DNA concentrations of the resulting sequencing libraries were measured with the Qubit 2.0 fluorometer dsDNA HS Assay (Thermo Fisher Scientific). Quantities and sizes of the resulting sequencing libraries were analyzed using Agilent BioAnalyzer 2100 (Agilent). The libraries were used in cluster formation on an Illumina cBOT cluster generation system with HiSeq X HD PE Cluster Kits (illumina). Whole-genome sequencing, with a target coverage 30× for 150 bp paired-end reads, was performed in WuxiNextCODE at Shanghai using an Illumina HiSeq X following Illumina-provided protocols. Each sample was run on a unique lane with at least 90 GB PF data (Supplementary Table 2) and reads data were quality controlled for ensuring that 80% of the bases achieved at least a base quality score of 30 (Supplementary Table 2).

DNA preparation for TGS

Genomic DNA was extracted from the peripheral blood samples and cells line using the optimized Cetyl Trimethyl Ammonium Bromide (CTAB) based protocol. Take frozen finely powdered tissues in a new 50 mL Falcon tube and mixed with the pre-heated extraction buffer (10 ml) for one sample. Briefly, vertex for 30sec to ensure sample is fully mixed with buffer. Fine grind is a key to obtaining high DNA quantity with lesser artifacts of resin. Next the falcon tube was kept into the 65°C incubator or water bath and mix gently by inversion after every 10 min till 45 min. After incubation, place the tube at room temperature for five min to reach to room temperature environment. Centrifuge the 50ml falcon tube for 5 min at 3000×g on room temperature. For the Third-Generation Sequencing (TGS), the greater the genome size, lower is speed of initial centrifugation. Transfer 1ml of

supernatant to each 2ml Eppendorf tubes already containing 1 ml of chloroform: isoamyl alcohol (24:1). Mix supernatant and chloroform: isoamyl alcohol by gentle inversions for 10 min and subsequently place the tube on ice for 10 min. Centrifuge the tube for 10 min at 5000 × g at 4°C. Transfer the upper aqueous phase into new 2ml tubes and add 5μl RNase A (10 mg/mL). Place the tube for 30 min at 37°C. After RNase treatment, add one volume of chloroform: isoamyl alcohol again and mix by inversions for 5 min. The tube is centrifuged for 10 min at 5000×g at 4°C. Transfer the clear supernatant into new 2ml tube and add half volume of 5 M NaCl to the sample and mix gently by inversions. Add 2 volumes of cold 95% ethanol and mix gently by inversion. The tubes are incubated at for 45 min in -20°C freezer. It should not be for more time as some remaining phenolics and resin may also precipitate with DNA. After incubation, the tubes were centrifuged at 5000 × g for 10 min at 4°C and the supernatant was gently removed. The pellet is washed two times with 1ml of 70% ethanol and the DNA is pellet by 5000 × g at 4°C for only 5 min. The supernatant is discarded and the pellet is air-dried (10 min). The pellet is allowed to re-suspend in 50μL of TE (10 mM Tris. HCl pH 8.0; 1 mM EDTA pH 8.0).

S6. DNA Quantification & Qualification

Total DNA was qualified and quantified as follows: (1) DNA purity and concentration were then examined using NanoDrop 2000; (2) Size distribution of and degradation degree DNA were measured by Pulsed-field Electrophoresis; (3) Accurate quantification of DNA were measured by Qubit Fluorometric Quantitation.

S7. Library construction and sequencing

Overview of the SMRTbell Express 2.0 Large-insert Library Workflow

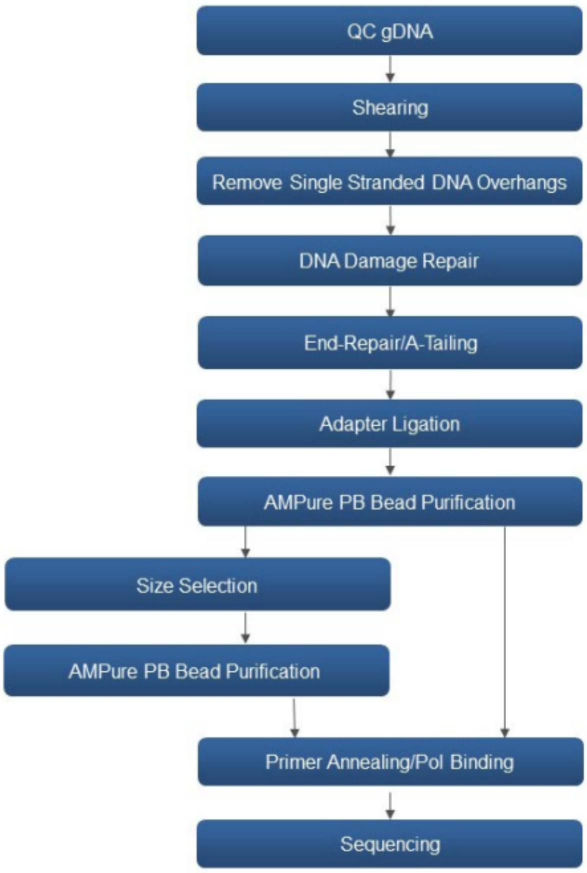

The library of 15 kb was constructed using a SMRTbell Express Template Prep Kit 2.0 (Pacific Biosciences, CA, USA). The construction includes **DNA shearing, damage repair, end repair, hairpin adapter ligation, size selection** and **purification of the library.** The process is as follows.

DNA shearing.

After examination of the quality of isolated DNA, dilute 2.0 μg gDNA into 200 μL 1X Elution Buffer to a final concentration of 10 ng/μL. Transfer gDNA to g-TUBE and centrifuge at 2400 x g (6000 rpm in the Eppendorf MiniSpin Plus) for 2 minutes. Then repeat spin until entire gDNA sample has passed through the orifice. (This may take 2-3 spins). Transfer the sheared gDNA to a fresh 1.5 mL Lo-bind microfuge tube. After that, concentrate DNA using AMPure PB beads to obtain the aimed DNA fragments for the construction of ＞15kb HiFi libraries.

DNA damage repair and end-repair/A-tailing

Use the following table to prepare the DNA damage repair reaction (Table 1). Firstly, pipette mix 10 times with wide-bore pipette tips. It is important to mix well. And spin down contents of tube with a quick spin in a microfuge. The incubate at 37°C for 30 minutes, then return the reaction to 4°C.

Use the following table to prepare the next end-repair/A-tailing processes (Table 2). Pipette mix 10 times with wide-bore pipette tips and spin down contents of tube with a quick spin in a microfuge. Then incubate at 20°C for 10 minutes and at 65°C for 30 minutes, then return the reaction to 4°C. The obtained samples are used to the next step.

Table 1 DNA damage repair reaction

Reagent (Reaction Mix 2)	Tube Cap Color	Volume	✓	Notes
Reaction Mix 1		55.0 μL		
DNA Damage Repair Mix v2		2.0 μL		
Total Volume		57.0 μL		

Table 2 End-Repair/A-tailing reaction

Reagent (Reaction Mix 3)		Volume	✓	Notes
Reaction Mix 2		57.0 μL		
End Prep Mix		3.0 μL		
Total Volume		60.0 μL		

Adapter ligation

Use the following table to prepare your reaction, adding the components below in the order listed. Pipette mix 10 times with wide-bore pipette tips, and spin down contents of tube with a quick spin in a microfuge. Then incubate at 20°C for 60 minutes, then return the reaction to 4°C.

Reagent (Reaction Mix 4)	Tube Cap Color	Volume	✓	Notes
Reaction Mix 3		60.0 µL		
Overhang Adapter v3		5.0 µL		
Ligation Mix		30.0 µL		
Ligation Additive		1.0 µL		
Ligation Enhancer		1.0 µL		
Total Volume		97.0 µL		

After quality control test, a SMRTbell library was obtained. The library was sequenced using a single 8 M SMAT Cell on the PacBio Sequel II/Sequel IIe platform (Pacific Biosciences, CA, USA).

Size selection and purification of SMRTbell Templates

When constructing large insert SMRTbell libraries for whole genome sequencing of complex organisms, it is beneficial to remove small insert SMRTbell templates by performing size selection with the BluePippin System (which collects fragments above a size cut-off threshold). And bring up the volume of eluted, size-selected DNA SMRTbell templates to 100 µL with 1X Elution Buffer to perform the purification of SMRTbell templates after size selection.

Anneal and Bind SMRTbell Library Templates and sequencing.

Use SMRT Link Sample Setup for instructions for primer annealing and polymerase binding. For primer annealing, use Sequencing Primer v4 (found in the SMRTbell Express Template Prep Kit v2). Sequencing Primer v4 is for diffusion loading only and cannot be used for MagBead loading. Wide bore pipette tips are required when constructing large insert libraries (>15 kb). The library was sequenced using a single 8 M SMAT Cell on the PacBio Sequel IIe platform (Pacific Biosciences, CA, USA).

Sequence quality checking and filtering

The PacBio SMRT-Analysis package (https://www.pacb.com) was used for the quality control of the raw polymerase reads; we removed the following types of polymerase reads: (1) Polymerase reads with length less than 50bp; (2)

Polymerase reads with the quality value lower than 0.8; (3) Polymerase reads containing the adaptor ligation to itself; （4）Removed the adaptor sequence in the polymerase reads. HiFi reads were generated by SMRTLink 9.0 software with parameters --min-passes=3 --min-rq=0.99.

S8. Hi-C sequencing

To anchor hybrid scaffolds onto the chromosome, genomic DNA was extracted for the Hi-C library from blood. We followed the standard protocol described previously with certain modifications (PMID: 19815776 and 25497547). In brief, we constructed the Hi-C library and obtained sequencing data via the Illumina Novaseq-6000 platform. About 1 ml blood sample was washed by PBS buffer and the pellet was resuspended and fixed in 2% formaldehyde in room temperature. Crosslinking was stopped by adding 0.125M glycine. The fixed sample was then resuspending and lysed in Hi-C lysis buffer. The purified nuclei were digested with 100 units of DpnII and marked by incubating with biotin-14-dATP. Biotin-14-dATP from non-ligated DNA ends was removed owing to the exonuclease activity of T4 DNA polymerase. The ligated DNA was sheared into 300-500 bp fragments, and then was blunt-end repaired and A-tailed, followed by purification through biotin-streptavidin-mediated pull down. Finally, the Hi-C libraries were quantified and sequenced using the Illumina Novaseq-6000 platform.

Genomic DNA preparation

DNA degradation and contamination were monitored on 1% agarose gels. DNA purity was checked using the NanoPhotometer®spectrophotometer (IMPLEN, CA, USA). DNA concentration was measured using Qubit®DNA Assay Kit in Qubit®2.0Flurometer (LifeTechnologies, CA, USA).

Library preparation and Illumina sequencing

A total amount of 1.5μg DNA per sample was used as input material for the DNA sample preparations. Sequencing libraries were generated using Truseq Nano DNA HT Sample preparation Kit (Illumina USA) following manufacturer's

recommendations and index codes were added to attribute sequences to each sample. Briefly, the DNA sample was fragmented by sonication to a size of 350bp, then DNA fragments were end polished, A-tailed, and ligated with the full-length adapter for Illumina sequencing with further PCR amplification. At last, PCR products were purified (AMPure XP system) and libraries were analyzed for size distribution by Agilent2100 Bioanalyzer and quantified using real-time PCR. These libraries constructed above were sequenced by Illumina NovaSeq-6000 platform and 150bp paired-end reads were generated with insert size around 350bp.

S9. High-Fidelity (HiFi) sequencing

Genomic DNA Preparation

High molecular weight genomic DNA was prepared by the CTAB method and followed by purification with QIAGEN® Genomic kit (Cat#13343, QIAGEN) for regular sequencing, according to the standard operating procedure provided by the manufacturer. The DNA degradation and contamination of the extracted DNA was monitored on 1% agarose gels. DNA purity was then detected using NanoDrop™ One UV-Vis spectrophotometer (Thermo Fisher Scientific, USA), of which OD260/280 ranging from 1.8 to 2.0 and OD 260/230 is between 2.0-2.2. At last, DNA concentration was further measured by Qubit® 4.0 Fluorometer (Invitrogen, USA).

Library preparation and sequencing

SMRTbell target size libraries were constructed for sequencing according to PacBio's standard protocol (Pacific Biosciences, CA, USA) using 15kb preparation solutions. The main steps for library preparation are: (1) gDNA shearing, (2) DNA damage repair, end repair and A-tailing, (3) ligation with hairpin adapters from the SMRTbell Express Template Prep Kit 2.0 (Pacific Biosciences), (4) nuclease treatment of SMRTbell library with SMRTbell Enzyme Cleanup Kit, (5) size selection, and (6) binding to polymerase. Briefly, a total amount of 15 µg DNA per sample was used for the DNA library preparations. The genomic DNA

sample was sheared by g-TUBEs (Covaris, USA) according to the expected size of the fragments for the library. Single-strand overhangs were then removed, and DNA fragments were damage repaired, end repaired and A-tailing. Then the fragments ligated with the hairpin adaptor for PacBio sequencing. And the library was treated by nuclease with SMRTbell Enzyme Cleanup Kit and purified by AMPure PB Beads. Target fragments were screened by the BluePippin (Sage Science, USA). The SMRTbell library was then purified by AMPure PB beads, and Agilent 2100 Bioanalyzer (Agilent technologies, USA) was used to detect the size of library fragments. Sequencing was performed on a PacBio Sequel II instrument with Sequencing Primer V2 and Sequel II Binding Kit 2.0 in Grandomics.

S10. Oxford Nanopore (ONT) Sequencing

Genomic DNA preparation

Samples were collected, and high molecular weight genomic DNA was prepared by the CTAB method and followed by purification with QIAGEN® Genomic kit (Cat#13343, QIAGEN) for regular sequencing, according to the standard operating procedure provided by the manufacturer. Ultra-long DNA was extracted by the SDS method without purification step to sustain the length of DNA. The DNA degradation and contamination of the extracted DNA was monitored on 1% agarose gels. DNA purity was then detected using NanoDrop ™ One UV-Vis spectrophotometer (Thermo Fisher Scientific, USA), of which OD260/280 ranging from 1.8 to 2.0 and OD 260/230 is between 2.0-2.2. At last, DNA concentration was further measured by Qubit® 4.0 Fluorometer (Invitrogen, USA).

Library preparation and sequencing

A total amount of 3-4 μg DNA per sample was used as input material for the ONT library preparations. After the sample was qualified, size-select (>50k and >100k) of long DNA fragments were performed using the PippinHT system (Sage Science, USA). Next, the ends of DNA fragments were repaired, and A-

ligation reaction were conducted with NEBNext Ultra II End Repair/dA-tailing Kit (Cat# E7546). The adapter in the SQK-LSK109 (Oxford Nanopore Technologies, UK) was used for further ligation reaction and DNA library was measured by Qubit® 4.0 Fluorometer (Invitrogen, USA).

S11. Genome Assembly and Quality Control

In our study, we collected 58 new HiFi samples covering 36 ethnic groups that represent genetic diversity in China. The 68 samples were sequenced on 2–5 SMRT Cells. The obtained subreads are converted into Hifi reads by the ccs v6.3.0 in Pacbio tools with *--hifi-kinetics --min-passes 3 --min-length 50*. At the individual level, we combined HiFi reads from multiple cells and applied hifiasm v0.16.1[2] to perform both primary assembly and diploid assembly for 68 PacBio HiFi samples.

As for 11 samples with Hi-C data, we ran hifiasm with the following command: *hifiasm -o $sample.asm -h1 sample.r1.fastq.gz -h2 sample.r2.fastq.gz -t 96 $sample.ccs.fastq.gz*. As for the remaining 57 samples, we ran hifiasm with the following command: *hifiasm -o $sample.asm -t 96 $sample.ccs.fastq.gz*.

We used QUAST v5.2.0[3] and Inspector v1.2[4] to assess the assembly quality of 68 primary assemblies and 136 haplotype assemblies. The assessment command of QUAST was as follows: *quast-lg.py $asm.fa -r $reference.fa -g $reference.gff.gz -o $asm/ --large --est-ref-size 3100000000 --no-icarus -t 64*. We removed 3 samples with primary assembly N50 < 20 Mb or contig number ≥ 2000, and 7 samples with two haplotype assembly N50 < 10 Mb or contig number ≥ 2000. Finally, 58 samples with 116 high-quality assemblies were retained for subsequent analyses.

We then annotated the unaligned sequences to different class of repeat including the interspersed repeats, low-complexity regions (LCRs), exact tandem repeats and centromeric satellites using RepeatMasker[5] (v4.1.2-p1) with NCBI/RMBLAST (v2.10.0) search engine and Dfam[6] (v3.3) database, SDUST

(v0.1, https://github.com/lh3/sdust), ETRF (Li, https://github.com/lh3/etrf) and dna-brnn[7] (v0.1) respectively.

S12. Complex structural variation detection

We aligned HiFi reads of 58 samples against T2T-CHM13[8] as reference by minimap2[9] with parameter "-ax map-hifi". We convert sam files to bam files and generate the corresponding index files by SAMtools[10]. Then we detected the complex structural variations (CSVs) by SVision[11] with parameters "*-s 5 --min_mapq 10 --min_sv_size 50 –qname --graph*" and pretrained model of "svision-cnn-model.ckpt". We selected CSVs (the term with <CSV> ALT column) from SVision results. To remove the coverage bias and get confidential CSV sets, we filtered CSVs with the ratio of supported read number to sequence coverage smaller than 25%. To get a nonredundant set of discovery CSVs from CSV calls from all samples, we adopted a merging strategy where HIFI032567D (with the most CSVs) served as the initial callset and new sites were added per sample. We excluded any CSVs in the sample that have 80% reciprocal overlap and the same type with an existing discovery CSV. This merging process yield 684 nonredundant CSVs. Samples were added by the descending order of CSV numbers. Each merged CSV is represented by a single CSV call in a single sample, but it is annotated with its discovery sample along with the corresponding genomic coordinate.

S13. Evaluate alignment quality of short-raeds using CPC reference

We selected samples from five East Asian populations of the 1000 Genomes Project (KGP) according to the standard of one male and one female per population. Fastq files of 10 samples were downloaded from ENA (study access: PRJEB31736). We first aligned the paired-end reads of each sample to the graph

genomes of CPC and HPRC by VG giraffe, then converted them to linear space (CHM13) using VG surject, and then marked the duplicate reads by Picard's markduplicate. We obtained the final BAM file for each sample. Through the samtools stats, we calculated the alignment quality of each sample.

S14. Pangenome growth

We covert the CPC graph to the vcf file by “vg deconstruct”, and calculated the haplotype depth of non-reference segments (presenting as SNPs and insertions in vcf file). The haplotype depth is defined as the frequency of non-reference sequences presenting in the assembled haplotypes. We counted the frequency of segments based on the GT information from the vcf file and calculated the segment length based on the ALT and REF sequences. We removed the super-large segments (> 3 Mb) from the results since they may be caused by graph constructing.

S15. Statistics on variants

We used vg deconstruct (https://github.com/vgteam/vg) to infer the variants and the genotype of assemblies from the graph, and variants with reference allele longer than 10 Mb were removed using vcfhub v1.0 (https://github.com/pangenome/vcfbub) because they are likely to be misjudged as deletions. Variants were classified as small variants (< 50 bp) and SVs (≥ 50 bp), and then were converted to per-sample biallelic VCF files using bcftools v1.14[12] separately to apply individual and haplotype level statistics.

S16. Alignment, variant identification and filtering

Per-individual sequence reads were aligned using ‘mem’ algorithm “*bwa mem –M –R @RG\tID:name\tSM:name*” in the Burrows-Wheeler Algorithm (BWA) v0.7.17-r1188[13] to the reference human genome (GRCh38), and then converted to BAM format, sorted by genomic position and indexed using SAMtools v1.15.1[10].

Picard toolkit v2.21.9 (http://broadinstitute.github.io/picard/) was used to mark the potential duplicate reads inherited from library construction step, in which the amplified PCR errors can introduce the wrong variants in variants calling[14,15]. The MarkDuplicates.jar in Picard was used for chromosome-wise duplicates marking per-individual.

BQSR module in the Genome Analysis Toolkit (GATK) v4.1.7.0 was applied to reduce the base quality score bias from the sequencer, and the 1000 Genomes phase I INDELs and dbSNP database (v151) were used as the training data sets. HaplotypeCaller module in GATK was used for SNPs and INDELs calling chromosome-wise simultaneously for each sample.

For population-based analyses, GATK GenotypeGVCFs module was applied to the GVCFs generated from previous step to call the variants for each chromosome of the combined samples.

The chromosome-wise raw variants were combined to genome-wide raw variants for population-based variants VCFs and individual-based GVCFs. GATK variants quality score recalibration (VQSR) module was used to filter the population based raw SNPs and INDELs, separately. Briefly speaking, VQSR used maximized sensitivity on these variants first, and then used some variants collections as training dataset to estimate the levels of specificity to filter these raw variants. For SNPs filtering, the variants collections contained HapMap 3.3 genotyping result, OMNI genotyping dataset, 1000 Genomes Phase I high confident SNPs and dbSNP dataset. For INDELs filtering, the variants collections contained Mills and 1000 Genomes Phase I gold standard INDELs.

S17. Population Genetic Analayses

To investigate the population diversity and coverage of CPC samples in the context of East Asia, we performed principal components analysis (PCA) at the individual level using SNPRelate v1.6.4[16] and evaluated with the first two pricinpal

components. We highlited HPRC and CPC samples with long-read sequencing data in the PC plot according to the linguatic classification.

To dissect diverse genetic components of 58 CPC core samples from different genetic background, we applied ADMIXTURE v1.3.0[17] to perform global ancestry inference using 36 populations corresponding to 58 CPC samples, 2 populations used in HPRC pangenome construction (CHS and KHV), and other KGP reference populations including African (YRI), South Asians (BEB, GIH, and ITU), and Europeans (CEU, FIN, and IBS). To reduce the bias of sample size, we performed ADMIXTURE analysis with 10 samples for each population. We first incorporate each HPRC and CPC sample to the corresponding population group, and further randomly incorporate remaning samples of each population to 10 samples. The ADMIXTURE analysis was replicated with 10 times from K=2 to K=12. The SNPs used in analyses were randomly selected from each 20 kb distance, and the final result was overall clustered from the results of 10 runs. The 58 CPC samples and 4 HPRC samples were labeled in the ADMIXTURE plot with short vertical lines.

S18. Functional annotation and enrichment analysis of genes

We characterized gene functions and further conducted enrichment analysis based on several knowledgebases or databases embedded in clusterProfiler v3.10.1[18], including the Gene Ontology (GO)[19], the Disease Ontology (DO)[20] , and the Kyoto Encyclopedia of Genes and Genomes (KEGG)[21]. Assuming that M in the N total background genes, and k in the n query genes are annotated to the functional category of interest, we calculated the odds ratio (OR) as $\frac{k/(n-k)}{(M-k)/(N-M-(n-k))}$. The P-values were calculated based on the one-sided Fisher's exact test. They were futher adjusted for multiple comparisons using the Benjamini-Hochberg (BH) procedure and were also adjusted for the false discovery rate (FDR). For some genes of particular interest, we applied

GeneAnalytics[22] online (geneanalytics.genecards.org) to obtain expression-based and function-based annotations.

We also searched for the NHGRI-EBI Catalog of human genome-wide association studies (GWAS Catalog, https://www.ebi.ac.uk/gwas/) for possible phenotypic consequences of the genes. The proportion of the approximately independent novel loci (adjacent novel SVs with distance < 50kb were merged) located < 50kb around the GWAS variants was compared with 1000 sets of randomly sampled common loci with matched size distribution. The *P*-values were estimated by the one-sided Wilcoxon rank-sum test, and were adjusted by the BH method across traits. Alternatively, we performed permutation test by randomly shuffling the novel SVs and the common loci with matched size. The *P*-values were calculated based on 1000 permutations, and the BH adjustment were performed across traits with more hits by the novel SVs than that by the common loci.

S19. Archaic introgression segments detection and functional annotation

We performed ArchaicSeeker v2.0[23] (AS2) to identify archaic introgression segments (AIS) in the CPC and HPRC genomes. Prior to the analysis, we converted the genomic coordinates in the phased VCFs from human genome assembly GRCh38 to GRCh37 as required by AS2, using LiftoverVcf embedded in Picard toolkit v1.117 (http://broadinstitute.github.io/picard/), and then removed the multi-allelic SNPs. We focused on the Neanderthal-like or Denisovan-like AISs identified by AS2, and then used GFF3 (Generic Feature Format Version 3) of GRCh37 version to annotate these AIS. To detect the novel AIS in CPC compared to HPRC, we applied BEDtools[24] to remove the overlapping regions in the AISs identified in CPC and HPRC. The sharing of archaic-introgression segments in any two given populations named as ancestry-sharing ratio was calculated as follows:

$$S_{ij} = \frac{\sum_{k=1}^{n}(p_{ik} \times p_{jk} \times L_k)}{\sqrt{P_i \times P_j} \times L}$$

$$p_{ik} = \begin{cases} 1, & (if\ i\ population\ has\ archaic\ introgression\ at\ position\ k) \\ 0, & (otherwise) \end{cases}$$

where P_i represents the genome-wide archaic introgression proportion of population i, p_{ik} denotes whether there is archaic introgression of population i at position k, L_k is the segment length, and $L = \sum_{k=1}^{n} L_k$ is the total length of the genome. S_{ij} measures the ratio of archaic-introgression-sharing to the random archaic-introgression-sharing of any two populations. We then used pheatmap1.0.12 to generate a heatmap for the ancestry-sharing visualization.

Alternatively, we applied vg giraffe to map the reads of Altai Neanderthal and Denisovan to the graph genome, and then use vg view to get the nodes from the mapped reads. We compared the graph nodes covered by the archaic reads across assemblies, and include those unaligned to the African genomes as potential archaic introgression SNVs/SVs. As linkage disequilibrium was not taken into consideration in this strategy, the estimated archaic introgression proportion would be much smaller than that obtained by ArchaicSeeker 2.0.

S20. Testing for neutrality by estimating Tajima's *D*

We measured the chromosome-wide genetic diversity by the estimator of Tajima's *D*[25] which may indicate possible signatures of natural selection. Tajima's D was calculated along the chromosomes with a sliding window of 20 kb in size, advanced by 10 kb, using in-house scripts. The theoretical *P*-values of Tajima's D were estimated following the β distribution, and were adjusted for multiple comparisons with the FDR method.

S21. References

1 Liao, W.-W. *et al.* A Draft Human Pangenome Reference. *bioRxiv*, 2022.2007.2009.499321, doi:10.1101/2022.07.09.499321 (2022).

2 Cheng, H., Concepcion, G. T., Feng, X., Zhang, H. & Li, H. Haplotype-resolved de novo assembly using phased assembly graphs with hifiasm. *Nat Methods* **18**, 170-175, doi:10.1038/s41592-020-01056-5 (2021).

3 Mikheenko, A., Prjibelski, A., Saveliev, V., Antipov, D. & Gurevich, A. Versatile genome assembly evaluation with QUAST-LG. *Bioinformatics* **34**, i142-i150, doi:10.1093/bioinformatics/bty266 (2018).

4 Chen, Y., Zhang, Y., Wang, A. Y., Gao, M. & Chong, Z. Accurate long-read de novo assembly evaluation with Inspector. *Genome Biol* **22**, 312, doi:10.1186/s13059-021-02527-4 (2021).

5 Tarailo-Graovac, M. & Chen, N. Using RepeatMasker to identify repetitive elements in genomic sequences. *Curr Protoc Bioinformatics* **Chapter 4**, 4.10.11-14.10.14, doi:10.1002/0471250953.bi0410s25 (2009).

6 Storer, J., Hubley, R., Rosen, J., Wheeler, T. J. & Smit, A. F. The Dfam community resource of transposable element families, sequence models, and genome annotations. *Mobile DNA* **12**, 2, doi:10.1186/s13100-020-00230-y (2021).

7 Li, H. Identifying centromeric satellites with dna-brnn. *Bioinformatics* **35**, 4408-4410, doi:10.1093/bioinformatics/btz264 (2019).

8 Nurk, S. *et al.* The complete sequence of a human genome. *Science* **376**, 44-53, doi:10.1126/science.abj6987 (2022).

9 Li, H. New strategies to improve minimap2 alignment accuracy. *Bioinformatics* **37**, 4572-4574, doi:10.1093/bioinformatics/btab705 (2021).

10 Li, H. *et al.* The Sequence Alignment/Map format and SAMtools. *Bioinformatics* **25**, 2078-2079, doi:10.1093/bioinformatics/btp352 (2009).

11 Lin, J. *et al.* SVision: a deep learning approach to resolve complex structural variants. *Nat Methods* **19**, 1230-1233, doi:10.1038/s41592-022-01609-w (2022).

12 Danecek, P. *et al.* Twelve years of SAMtools and BCFtools. *Gigascience* **10**, doi:10.1093/gigascience/giab008 (2021).

13 Li, H. & Durbin, R. Fast and accurate long-read alignment with Burrows-Wheeler transform. *Bioinformatics* **26**, 589-595, doi:10.1093/bioinformatics/btp698 (2010).

14 McKenna, A. *et al.* The Genome Analysis Toolkit: a MapReduce framework for analyzing next-generation DNA sequencing data. *Genome Res* **20**, 1297-1303, doi:10.1101/gr.107524.110 (2010).

15 DePristo, M. A. *et al.* A framework for variation discovery and genotyping using next-generation DNA sequencing data. *Nat Genet* **43**, 491-498, doi:10.1038/ng.806 (2011).

16 Zheng, X. *et al.* A high-performance computing toolset for relatedness and principal component analysis of SNP data. *Bioinformatics* **28**, 3326-3328, doi:10.1093/bioinformatics/bts606 (2012).

17 Alexander, D. H., Novembre, J. & Lange, K. Fast model-based estimation of ancestry in unrelated individuals. *Genome Res* **19**, 1655-1664, doi:10.1101/gr.094052.109 (2009).

18 Yu, G., Wang, L. G., Han, Y. & He, Q. Y. clusterProfiler: an R package for comparing biological themes among gene clusters. *OMICS* **16**, 284-287, doi:10.1089/omi.2011.0118 (2012).

19 Ashburner, M. *et al.* Gene ontology: tool for the unification of biology. The Gene Ontology Consortium. *Nat Genet* **25**, 25-29, doi:10.1038/75556 (2000).

20 Schriml, L. M. *et al.* Disease Ontology: a backbone for disease semantic integration. *Nucleic Acids Res* **40**, D940-946, doi:10.1093/nar/gkr972 (2012).

21 Kanehisa, M. & Goto, S. KEGG: kyoto encyclopedia of genes and genomes. *Nucleic Acids Res* **28**, 27-30, doi:10.1093/nar/28.1.27 (2000).

22 Ben-Ari Fuchs, S. *et al.* GeneAnalytics: An Integrative Gene Set Analysis Tool for Next Generation Sequencing, RNAseq and Microarray Data. *OMICS* **20**, 139-151, doi:10.1089/omi.2015.0168 (2016).

23 Yuan, K. *et al.* Refining models of archaic admixture in Eurasia with ArchaicSeeker 2.0. *Nat Commun* **12**, 6232, doi:10.1038/s41467-021-26503-5 (2021).

24 Quinlan, A. R. & Hall, I. M. BEDTools: a flexible suite of utilities for comparing genomic features. *Bioinformatics* **26**, 841-842, doi:10.1093/bioinformatics/btq033 (2010).

25 Tajima, F. Statistical method for testing the neutral mutation hypothesis by DNA polymorphism. *Genetics* **123**, 585-595, doi:10.1093/genetics/123.3.585 (1989).

S22. Supplementary Tables

Supplementary Table 1 Information of the samples for sequencing in CPC

Sample ID	Population	Gender	Sequencing Platform	Latitude	Longitude	Source
HIFI032682D	Achang	M	PacBio HiFi, NGS	24.43	98.59	CPC
HIFI032585D	Bai	M	PacBio HiFi, NGS	26.11	99.95	CPC
HIFI032069D	Blang	F	PacBio HiFi, NGS	21.96	100.45	CPC
HIFI032373D	Blang	M	PacBio HiFi, NGS	21.96	100.45	CPC
HIFI032487D	Bouyei	F	PacBio HiFi, NGS	24.98	105.81	CPC
HIFI032018D	Chosen	M	PacBio HiFi, NGS	42.91	129.51	CPC
HIFI032306D	Chosen	F	PacBio HiFi, NGS	42.91	129.51	CPC
RY05-CNCSM0001	Chosen	M	PacBio HiFi, ONT, Hi-C, NGS	43.12	128.91	CPC
HIFI032668D	Daur	M	PacBio HiFi, tell-seq, NGS	49.21	119.74	CPC
HIFI032698D	Daur	F	PacBio HiFi, NGS	49.21	119.74	CPC
HIFI032292D	Deang	F	PacBio HiFi, NGS	24.43	98.59	CPC
HIFI032462D	Deang	M	PacBio HiFi, NGS	24.43	98.59	CPC
HIFI032473D	Dong	M	PacBio HiFi, NGS	25.78	109.60	CPC
HIFI032510D	Dong	F	PacBio HiFi, NGS	25.78	109.60	CPC
HIFI032450D	Drung	M	PacBio HiFi, NGS	27.74	98.67	CPC
HIFI032706D	Evenki	M	PacBio HiFi, NGS	49.21	119.74	CPC
HIFI032007D	Kinh	F	PacBio HiFi, NGS	21.55	107.97	CPC
RY06-CNKGF0001	Kyrgyz	F	PacBio HiFi, ONT, Hi-C, NGS	39.15	75.95	CPC
RY07-CNKZF0001	Kazakh	F	PacBio HiFi, ONT, Hi-C, NGS	44.91	82.07	CPC
RY08-CNZHF0001	Zhuang	F	PacBio HiFi, ONT, Hi-C, NGS	23.74	106.92	CPC
RY09-CNHUM0001	Hui	M	PacBio HiFi, ONT, Hi-C, NGS	39.02	106.37	CPC
HIFI032513D	Kinh	M	PacBio HiFi, NGS	21.55	107.97	CPC
HIFI032685D	Hezhen	M	PacBio HiFi, NGS	47.64	132.51	CPC
HIFI032440D	Jingpo	M	PacBio HiFi, NGS	24.43	98.59	CPC
HIFI032429D	Jino	M	PacBio HiFi, NGS	22.01	100.80	CPC
HIFI032731D	Lisu	F	PacBio HiFi, NGS	25.85	98.85	CPC
HIFI032289D	Man	M	PacBio HiFi, NGS	40.28	123.29	CPC
HIFI032167D	Miao	M	PacBio HiFi, NGS	28.17	109.20	CPC
RY04-CNMHM0001	Miao	M	PacBio HiFi, ONT, Hi-C, NGS	26.58	109.70	CPC
HIFI032164D	Mongol	M	PacBio HiFi, NGS	39.82	109.96	CPC
RY10-CNMGF0001	Mongol	F	PacBio HiFi, ONT, Hi-C, NGS	41.60	119.35	CPC
HIFI032607D	Mongol	F	PacBio HiFi, NGS	41.70	110.43	CPC
HIFI032604D	Khatso	F	PacBio HiFi, NGS	24.11	102.77	CPC
HIFI032161D	Mosuo	F	PacBio HiFi, NGS	27.28	100.85	CPC
HIFI032422D	Mosuo	M	PacBio HiFi, tell-seq, NGS	27.28	100.85	CPC
HIFI032501D	Mosuo	M	PacBio HiFi, NGS	27.28	100.85	CPC
HIFI032693D	Naxi	M	PacBio HiFi, tell-seq, NGS	26.82	100.24	CPC
HIFI032335D	Oroqen	U*	PacBio HiFi, tell-seq, NGS	50.60	123.73	CPC
HIFI032420D	Qiang	M	PacBio HiFi, NGS	31.68	103.85	CPC
HIFI032567D	Qiang	F	PacBio HiFi, NGS	31.68	103.85	CPC
HIFI032662D	Qiang	M	PacBio HiFi, tell-seq, NGS	31.68	103.85	CPC
HIFI032453D	Salar	M	PacBio HiFi, NGS	35.85	102.49	CPC
HIFI032586D	Salar	F	PacBio HiFi, NGS	35.85	102.49	CPC
HIFI032302D	She	F	PacBio HiFi, NGS	27.09	119.65	CPC
HIFI032591D	She	M	PacBio HiFi, NGS	27.09	119.65	CPC
RY03-CNSHM0001	She	M	PacBio HiFi, ONT, Hi-C, NGS	22.73	114.25	CPC
HIFI032349D	Tu	M	PacBio HiFi, NGS	36.84	101.96	CPC
HIFI032454D	Tu	F	PacBio HiFi, NGS	36.84	101.96	CPC
HIFI032097D	Tujia	F	PacBio HiFi, NGS	29.49	109.41	CPC
HIFI032103D	Tujia	M	PacBio HiFi, NGS	29.49	109.41	CPC
RY11-CNTJM0001	Tujia	M	PacBio HiFi, ONT, Hi-C, NGS	30.01	108.12	CPC
HIFI032529D	Wa	F	PacBio HiFi, NGS	22.65	99.60	CPC
HIFI032711D	Wa	M	PacBio HiFi, NGS	22.65	99.60	CPC
HIFI032182D	Yi	M	PacBio HiFi, NGS	26.82	100.24	CPC
HIFI032566D	Yugur	M	PacBio HiFi, NGS	38.84	99.61	CPC
HIFI032692D	Yugur	F	PacBio HiFi, NGS	38.84	99.61	CPC
RY01-CNYAM0001	Yao	M	PacBio HiFi, ONT, Hi-C, NGS	24.82	111.28	CPC
RY02-CNUGM0001	Uyghur	M	PacBio HiFi, ONT, Hi-C, NGS	39.47	76.00	CPC
Han	Han-N	M	ONT, NGS	31.81	117.22	CPC
LX	Han-S	M	ONT, NGS	23.79	108.77	CPC
HZZ1	Han-C	F	ONT, NGS	34.76	113.66	CPC
HZZ2	Han-C	M	ONT, NGS	34.76	113.66	CPC
Xinjiang	Uyghur	M	ONT, NGS	41.21	85.33	CPC
Zang	Tibetan	M	ONT, NGS	31.68	88.17	CPC
Mos	Mosuo	M	ONT, NGS	27.72	100.79	CPC
HG00438	Han-S	F	PcaBio HiFi, ONT, Hi-C, BioNano, NGS	23.14	113.23	HPRC
HG00621	Han-S	M	PcaBio HiFi, ONT, Hi-C, BioNano, NGS	23.14	113.23	HPRC
HG00673	Han-S	M	PcaBio HiFi, ONT, Hi-C, BioNano, NGS	23.14	113.23	HPRC
HX1	Han-S	M	PacBio CLR, BioNano, 10X Genomics, NGS	23.79	108.77	Shi et al., 2016
NH1	Han-N	M	PacBio CLR, BioNano, NGS	36.77	117.05	Du et al., 2019
TJ1	Tujia	M	PacBio CLR, BioNano, Hi-C, 10X Genomics, NGS	30.65	111.31	Lou et al., 2022
ZF1	Tibetan	M	PacBio CLR, BioNano, Hi-C, 10X Genomics, NGS	29.28	88.90	Ouzhuluobu et al., 2020

* Uncertain due to inconsistency of self reported sex and that based on genetic data.

Supplementary Table 2 Summary statistics of the 116 CPC genome assemblies

Genome	Ethnicity	Contig N50 (Mb)	Contig Number	Assembly Size (Gb)	Genome	Ethnicity	Contig N50 (Mb)	Contig Number	Assembly Size (Gb)
HIFI032097D-H1	Tujia	49.18	470	3.02	HIFI032429D-H2	Jino	17.02	843	3.01
HIFI032097D-H2	Tujia	33.25	484	3.04	HIFI032440D-H1	Jingpo	33.69	568	3.04
HIFI032103D-H1	Tujia	33.10	644	3.02	HIFI032440D-H2	Jingpo	36.14	519	2.97
HIFI032103D-H2	Tujia	30.71	699	3.02	HIFI032450D-H1	Drung	59.52	559	3.03
RY11-CNTJM0001-H1	Tujia	42.61	718	2.89	HIFI032450D-H2	Drung	40.89	565	3.02
RY11-CNTJM0001-H2	Tujia	40.81	579	3.12	HIFI032453D-H1	Salar	51.43	458	3.02
RY10-CNMGF0001-H1	Mongol	42.62	533	3.06	HIFI032453D-H2	Salar	49.27	389	2.97
RY10-CNMGF0001-H2	Mongol	43.44	437	3.01	HIFI032462D-H1	Deang	19.76	1039	3.04
HIFI032164D-H1	Mongol	20.94	845	3.03	HIFI032462D-H2	Deang	11.66	1075	3.00
HIFI032164D-H2	Mongol	21.57	801	3.03	HIFI032473D-H1	Dong	43.19	593	3.01
HIFI032604D-H1	Khatso	42.74	541	3.02	HIFI032473D-H2	Dong	29.94	558	3.03
HIFI032604D-H2	Khatso	27.40	528	3.02	HIFI032487D-H1	Bouyei	41.02	881	3.03
RY04-CNMHM0001-H1	Miao	87.30	461	2.90	HIFI032487D-H2	Bouyei	16.29	955	3.00
RY04-CNMHM0001-H2	Miao	81.84	397	3.06	HIFI032501D-H1	Mosuo	29.87	763	3.01
HIFI032167D-H1	Miao	17.60	1192	3.00	HIFI032501D-H2	Mosuo	22.31	787	3.02
HIFI032167D-H2	Miao	11.71	1269	3.00	HIFI032510D-H1	Dong	28.41	807	3.03
RY01-CNYAM0001-H1	Yao	32.31	620	3.06	HIFI032510D-H2	Dong	22.02	705	3.02
RY01-CNYAM0001-H2	Yao	39.83	496	2.93	HIFI032529D-H1	Wa	33.34	640	3.02
RY03-CNSHM0001-H1	She	52.23	585	2.88	HIFI032529D-H2	Wa	33.83	615	3.00
RY03-CNSHM0001-H2	She	40.08	476	3.07	HIFI032567D-H1	Qiang	30.63	862	3.04
HIFI032302D-H1	She	26.21	733	3.01	HIFI032567D-H2	Qiang	14.68	986	3.01
HIFI032302D-H2	She	28.69	681	3.02	HIFI032585D-H1	Bai	34.36	893	3.00
HIFI032513D-H1	Kinh	36.39	690	3.01	HIFI032585D-H2	Bai	20.79	818	3.03
HIFI032513D-H2	Kinh	25.28	712	3.02	HIFI032586D-H1	Salar	44.02	577	3.03
HIFI032007D-H1	Kinh	23.40	962	3.02	HIFI032586D-H2	Salar	27.70	639	3.03
HIFI032007D-H2	Kinh	13.88	1106	3.02	HIFI032591D-H1	She	24.11	848	3.05
HIFI032018D-H1	Chosen	35.60	813	2.98	HIFI032591D-H2	She	21.87	901	3.00
HIFI032018D-H2	Chosen	28.86	752	3.01	HIFI032607D-H1	Mongol	38.11	497	3.04
HIFI032306D-H1	Chosen	39.26	590	3.03	HIFI032607D-H2	Mongol	32.01	515	3.02
RY05-CNCSM0001-H1	Chosen	58.98	533	3.06	HIFI032662D-H1	Qiang	34.99	1164	3.04
RY05-CNCSM0001-H2	Chosen	83.61	451	2.89	HIFI032662D-H2	Qiang	18.74	1128	2.97
HIFI032306D-H2	Chosen	25.23	644	3.01	HIFI032668D-H1	Daur	23.52	817	3.01
HIFI032692D-H1	Yugur	44.18	613	3.04	HIFI032668D-H2	Daur	24.92	749	3.02
HIFI032692D-H2	Yugur	40.25	549	3.04	HIFI032682D-H1	Achang	37.94	671	3.01
HIFI032566D-H1	Yugur	39.04	505	2.98	HIFI032682D-H2	Achang	23.29	690	2.99
HIFI032566D-H2	Yugur	36.57	517	3.00	HIFI032685D-H1	Hezhen	19.23	1004	3.01
RY02-CNUGM0001-H1	Uyghur	31.63	643	2.90	HIFI032685D-H2	Hezhen	26.91	784	3.04
RY02-CNUGM0001-H2	Uyghur	24.85	555	3.07	HIFI032693D-H1	Naxi	86.68	508	3.06
HIFI032069D-H1	Blang	37.33	681	3.02	HIFI032693D-H2	Naxi	70.62	316	2.93
HIFI032069D-H2	Blang	27.19	761	3.01	HIFI032698D-H1	Daur	37.78	561	3.04
HIFI032161D-H1	Mosuo	29.69	703	3.03	HIFI032698D-H2	Daur	37.73	536	3.02
HIFI032161D-H2	Mosuo	24.78	749	3.02	HIFI032706D-H1	Evenki	38.47	698	3.00
HIFI032182D-H1	Yi	45.14	493	3.04	HIFI032706D-H2	Evenki	40.79	588	3.02
HIFI032182D-H2	Yi	32.50	485	2.99	HIFI032711D-H1	Wa	57.22	550	3.02
HIFI032289D-H1	Man	57.76	502	3.02	HIFI032711D-H2	Wa	42.91	498	2.99
HIFI032289D-H2	Man	37.28	546	3.00	HIFI032731D-H1	Lisu	25.51	757	3.02
HIFI032292D-H1	Deang	19.43	1084	3.02	HIFI032731D-H2	Lisu	23.61	740	3.04
HIFI032292D-H2	Deang	16.80	1082	3.01	RY06-CNKGF0001-H1	Kyrgyz	68.01	399	3.00
HIFI032335D-H1	Oroqen	17.67	1286	3.05	RY06-CNKGF0001-H2	Kyrgyz	69.00	294	3.04
HIFI032335D-H2	Oroqen	12.54	1568	3.04	RY07-CNKZF0001-H1	Kazakh	51.74	529	3.01
HIFI032349D-H1	Tu	19.03	982	3.03	RY07-CNKZF0001-H2	Kazakh	69.84	415	3.05
HIFI032349D-H2	Tu	25.41	782	3.00	RY08-CNZHF0001-H1	Zhuang	52.55	521	3.03
HIFI032454D-H1	Tu	27.39	728	3.03	RY08-CNZHF0001-H2	Zhuang	52.72	463	3.03
HIFI032454D-H2	Tu	24.78	642	3.02	RY09-CNHUM0001-H1	Hui	51.71	563	2.88
HIFI032373D-H1	Blang	38.87	691	3.00	RY09-CNHUM0001-H2	Hui	49.71	487	3.07
HIFI032373D-H2	Blang	23.97	680	3.01					
HIFI032420D-H1	Qiang	36.22	484	3.04					
HIFI032420D-H2	Qiang	38.83	434	2.97					
HIFI032422D-H1	Mosuo	21.66	824	3.02					
HIFI032422D-H2	Mosuo	14.36	926	3.02					
HIFI032429D-H1	Jino	26.67	877	3.01					

Supplementary Table 3 The length (Mb) of different types of the sequences unaligned to GRCh38 reference genome per assembly

	Satellite	Low-repeat	Other-repeat	Other-TE	Other-LCR	VNTR	L1	STR	SVA	Alu	ERV	Sum
HIFI032007D_1	65.11	3.40	1.78	0.32	0.52	0.96	0.19	0.36	0.02	0.03	0.09	72.77
HIFI032007D_2	61.82	2.46	1.80	0.26	0.50	0.76	0.24	0.26	0.01	0.03	0.06	68.21
HIFI032018D_1	49.61	2.87	7.00	0.18	0.74	0.90	0.16	0.26	0.01	0.03	0.07	61.82
HIFI032018D_2	60.95	3.15	6.82	0.36	0.52	0.58	0.24	0.26	0.01	0.04	0.36	73.27
HIFI032069D_1	53.64	2.78	1.37	0.25	0.37	1.07	0.19	0.37	0.01	0.04	0.05	60.13
HIFI032069D_2	63.21	3.26	1.71	0.21	0.46	0.64	0.26	0.32	0.01	0.03	0.10	70.21
HIFI032097D_1	53.91	2.55	1.88	0.31	0.54	0.86	0.14	0.25	0.01	0.04	0.04	60.53
HIFI032097D_2	67.24	2.20	1.51	0.20	0.51	0.84	0.23	0.41	0.01	0.03	0.07	73.27
HIFI032103D_1	64.85	2.35	8.95	0.19	0.65	0.87	0.27	0.27	0.01	0.03	0.31	78.76
HIFI032103D_2	55.97	3.41	9.92	0.30	0.32	1.03	0.19	0.21	0.01	0.04	0.05	71.44
HIFI032161D_1	54.71	2.89	1.26	0.19	0.26	0.85	0.14	0.33	0.01	0.02	0.06	60.72
HIFI032161D_2	59.38	2.38	1.71	0.38	0.35	0.82	0.20	0.27	0.01	0.03	0.05	65.59
HIFI032164D_1	64.42	3.77	7.21	0.31	0.55	0.77	0.17	0.30	0.02	0.03	0.08	77.63
HIFI032164D_2	63.66	3.57	7.37	0.22	0.61	0.78	0.25	0.25	0.02	0.03	0.24	76.99
HIFI032167D_1	51.29	3.34	3.33	0.35	0.44	0.99	0.22	0.14	0.01	0.03	0.06	60.21
HIFI032167D_2	53.42	2.28	3.44	0.28	0.47	0.87	0.19	0.16	0.02	0.03	0.31	61.46
HIFI032182D_1	61.85	3.60	8.18	0.26	0.59	0.82	0.21	0.34	0.01	0.03	0.24	76.13
HIFI032182D_2	56.74	2.88	6.08	0.22	0.36	0.63	0.23	0.33	0.01	0.04	0.10	67.62
HIFI032289D_1	65.75	3.09	12.29	0.25	0.67	0.66	0.17	0.18	0.02	0.03	0.06	83.17
HIFI032289D_2	51.77	3.18	10.83	0.35	0.37	0.85	0.24	0.13	0.01	0.04	0.26	68.02
HIFI032292D_1	55.86	2.74	1.21	0.25	0.57	0.59	0.19	0.26	0.01	0.03	0.08	61.78
HIFI032292D_2	54.99	2.68	1.26	0.34	0.46	0.88	0.21	0.22	0.02	0.03	0.07	61.16
HIFI032302D_1	59.62	2.70	1.37	0.25	0.40	0.67	0.20	0.27	0.01	0.03	0.04	65.57
HIFI032302D_2	54.29	3.06	1.63	0.25	0.40	0.70	0.26	0.36	0.01	0.03	0.04	61.04
HIFI032306D_1	54.94	3.80	1.91	0.18	1.24	0.76	0.23	0.32	0.01	0.02	0.06	63.48
HIFI032306D_2	51.45	2.56	1.92	0.29	0.42	0.85	0.19	0.27	0.01	0.03	0.05	58.03
HIFI032335D_1	46.21	3.15	10.55	0.29	0.55	0.76	0.26	0.32	0.01	0.03	0.19	62.31
HIFI032335D_2	52.33	2.68	9.46	0.18	0.52	0.85	0.19	0.07	0.01	0.03	0.20	66.52
HIFI032349D_1	59.15	3.83	7.66	0.33	0.41	0.77	0.19	0.18	0.02	0.03	0.09	72.65
HIFI032349D_2	59.40	2.91	5.80	0.22	0.52	0.54	0.18	0.22	0.01	0.04	0.28	70.13
HIFI032373D_1	54.68	3.34	4.56	0.23	0.53	0.76	0.26	0.17	0.02	0.03	0.27	64.84
HIFI032373D_2	52.71	3.26	4.94	0.35	0.57	0.98	0.20	0.14	0.01	0.03	0.06	63.26
HIFI032420D_1	57.46	3.92	5.89	0.30	1.19	0.66	0.30	0.28	0.01	0.03	0.29	70.33
HIFI032420D_2	56.99	3.40	5.65	0.41	0.50	1.24	0.23	0.40	0.01	0.03	0.06	68.93
HIFI032422D_1	58.97	2.73	11.13	0.33	0.53	0.77	0.21	0.17	0.01	0.03	0.09	74.97
HIFI032422D_2	52.36	3.42	9.33	0.20	0.53	0.92	0.21	0.25	0.01	0.03	0.28	67.53
HIFI032429D_1	49.90	3.05	4.50	0.22	1.23	0.64	0.19	0.23	0.01	0.03	0.23	60.23
HIFI032429D_2	65.94	3.49	4.81	0.25	0.54	0.79	0.20	0.32	0.02	0.03	0.08	76.48
HIFI032440D_1	68.00	3.21	9.58	0.26	1.43	1.01	0.19	0.30	0.01	0.03	0.06	84.07
HIFI032440D_2	45.74	2.44	8.35	0.24	0.32	0.82	0.15	0.34	0.01	0.03	0.26	58.72
HIFI032450D_1	54.67	3.38	5.12	0.23	0.54	0.95	0.17	0.45	0.02	0.04	0.31	65.88
HIFI032450D_2	65.82	3.35	7.04	0.28	0.57	0.74	0.17	0.44	0.01	0.03	0.09	78.55
HIFI032453D_1	61.70	3.02	7.45	0.21	0.34	0.75	0.18	0.42	0.01	0.02	0.09	74.18
HIFI032453D_2	64.32	2.81	6.82	0.37	1.16	0.97	0.27	0.30	0.01	0.03	0.10	77.16
HIFI032454D_1	64.37	3.34	1.73	0.29	0.59	0.77	0.13	0.18	0.01	0.02	0.06	71.48
HIFI032454D_2	60.33	2.57	1.32	0.18	0.49	0.71	0.24	0.10	0.01	0.04	0.10	66.07
HIFI032462D_1	64.93	3.10	8.84	0.20	0.61	0.70	0.21	0.37	0.01	0.02	0.37	79.36
HIFI032462D_2	58.84	2.56	8.62	0.39	0.61	0.88	0.26	0.16	0.02	0.03	0.08	72.45
HIFI032473D_1	64.94	3.13	9.80	0.28	0.41	0.69	0.19	0.14	0.01	0.04	0.25	79.88
HIFI032473D_2	69.21	3.30	8.93	0.18	0.54	0.85	0.14	0.17	0.01	0.03	0.22	83.56
HIFI032487D_1	57.11	2.94	4.80	0.36	0.58	0.78	0.20	0.27	0.01	0.03	0.05	67.13
HIFI032487D_2	65.10	2.57	5.33	0.17	0.50	0.74	0.19	0.28	0.02	0.03	0.21	75.13
HIFI032501D_1	53.49	2.92	10.22	0.25	0.50	0.63	0.17	0.48	0.01	0.04	0.25	68.96
HIFI032501D_2	55.14	2.96	8.47	0.49	0.50	0.87	0.23	0.24	0.02	0.03	0.09	69.04
HIFI032510D_1	59.36	3.21	2.02	0.34	0.41	0.87	0.30	0.15	0.02	0.04	0.06	66.78
HIFI032510D_2	61.06	2.31	1.53	0.39	0.49	0.66	0.23	0.34	0.01	0.04	0.08	67.14
HIFI032513D_1	62.18	3.03	7.00	0.25	0.47	0.98	0.27	0.28	0.01	0.04	0.31	74.82
HIFI032513D_2	56.03	2.60	5.63	0.34	0.50	1.07	0.22	0.22	0.01	0.04	0.30	66.97
HIFI032529D_1	62.93	1.78	2.05	0.25	1.21	0.83	0.18	0.40	0.01	0.03	0.06	69.73
HIFI032529D_2	49.22	3.22	1.04	0.29	0.40	0.91	0.22	0.22	0.01	0.03	0.09	55.65
HIFI032566D_1	57.96	3.54	5.37	0.30	0.43	0.88	0.17	0.49	0.01	0.04	0.06	69.25
HIFI032566D_2	57.07	3.08	5.20	0.29	0.55	0.75	0.22	0.37	0.01	0.04	0.33	67.90
HIFI032567D_1	64.33	2.53	1.72	0.24	0.47	0.82	0.20	0.20	0.01	0.03	0.08	70.63
HIFI032567D_2	51.28	2.69	1.48	0.22	0.36	0.70	0.19	0.26	0.01	0.04	0.07	57.30
HIFI032585D_1	53.38	3.18	9.55	0.26	0.59	0.94	0.21	0.44	0.01	0.03	0.10	68.68
HIFI032585D_2	49.80	2.54	10.05	0.19	0.70	0.70	0.20	0.14	0.01	0.03	0.36	64.72
HIFI032586D_1	54.59	3.56	2.32	0.26	0.53	0.65	0.19	0.50	0.01	0.03	0.07	62.71
HIFI032586D_2	69.46	2.47	2.57	0.23	0.39	0.75	0.18	0.36	0.01	0.04	0.08	76.53
HIFI032591D_1	59.80	3.54	5.89	0.26	0.52	0.80	0.23	0.36	0.01	0.04	0.31	71.77
HIFI032591D_2	55.41	3.38	6.42	0.27	0.59	1.00	0.24	0.10	0.01	0.03	0.29	67.74
HIFI032604D_1	52.09	3.15	1.51	0.26	0.55	1.31	0.22	0.61	0.01	0.03	0.05	59.79
HIFI032604D_2	58.71	3.12	1.75	0.22	0.45	0.81	0.20	0.14	0.01	0.03	0.05	65.50
HIFI032607D_1	57.37	3.00	1.69	0.22	0.50	0.76	0.21	0.18	0.01	0.03	0.09	64.07
HIFI032607D_2	62.89	3.33	1.80	0.26	0.51	0.92	0.19	0.17	0.01	0.03	0.05	70.16
HIFI032662D_1	50.33	2.57	7.50	0.25	0.50	0.81	0.27	0.29	0.01	0.03	0.35	62.90
HIFI032662D_2	48.66	2.84	6.67	0.23	0.41	0.84	0.27	0.20	0.01	0.03	0.39	60.55
HIFI032668D_1	66.77	3.45	9.04	0.29	0.65	0.91	0.20	0.33	0.01	0.03	0.12	81.81
HIFI032668D_2	51.07	2.86	9.55	0.18	0.40	0.77	0.21	0.11	0.02	0.03	0.26	65.47
HIFI032682D_1	54.22	3.27	6.37	0.31	0.52	0.92	0.20	0.39	0.01	0.03	0.33	66.58
HIFI032682D_2	56.22	2.59	5.36	0.26	0.38	0.64	0.21	0.23	0.01	0.03	0.09	66.01

HIFI032685D_1	51.55	2.93	7.34	0.25	0.53	0.60	0.23	0.42	0.01	0.02	0.26	64.14
HIFI032685D_2	65.36	2.65	7.91	0.23	0.56	0.74	0.21	0.22	0.01	0.03	0.06	77.96
HIFI032692D_1	61.47	2.90	1.58	0.25	0.36	0.86	0.26	0.32	0.01	0.03	0.09	68.13
HIFI032692D_2	62.25	2.61	1.89	0.42	0.45	0.88	0.25	0.17	0.01	0.04	0.08	69.05
HIFI032693D_1	66.90	3.80	6.14	0.30	0.52	1.25	0.25	0.38	0.01	0.05	0.31	79.91
HIFI032693D_2	57.16	4.33	7.43	0.16	0.40	0.90	0.19	0.37	0.01	0.03	0.10	71.08
HIFI032698D_1	61.90	3.16	2.41	0.33	0.56	0.86	0.23	0.27	0.01	0.03	0.10	69.86
HIFI032698D_2	60.34	2.40	1.56	0.33	0.47	0.70	0.23	0.23	0.01	0.03	0.11	66.39
HIFI032706D_1	51.52	3.26	8.51	0.35	0.53	0.77	0.19	0.35	0.01	0.04	0.28	65.82
HIFI032706D_2	61.50	3.52	11.09	0.24	0.40	0.79	0.20	0.35	0.01	0.02	0.33	78.46
HIFI032711D_1	58.78	2.61	8.50	0.25	0.70	0.64	0.24	0.41	0.01	0.03	0.32	72.50
HIFI032711D_2	51.10	3.34	9.31	0.22	0.45	0.94	0.23	0.37	0.01	0.05	0.07	66.09
HIFI032731D_1	50.58	2.61	1.89	0.25	0.44	0.93	0.18	0.22	0.02	0.03	0.07	57.23
HIFI032731D_2	62.97	2.44	2.23	0.19	0.38	0.74	0.20	0.43	0.01	0.03	0.10	69.73
RY01_1	60.30	2.99	9.16	0.20	0.39	0.83	0.23	0.23	0.01	0.03	0.08	74.45
RY01_2	65.16	4.15	13.78	0.23	0.61	0.92	0.21	0.23	0.01	0.03	0.33	85.65
RY02_1	47.34	3.43	8.51	0.28	0.59	0.73	0.18	0.27	0.01	0.02	0.28	61.66
RY02_2	68.52	2.79	7.81	0.30	0.54	0.81	0.16	0.28	0.01	0.03	0.05	81.30
RY03_1	53.09	4.07	5.36	0.35	0.54	0.83	0.20	0.37	0.01	0.03	0.05	64.89
RY03_2	65.39	3.10	7.12	0.15	0.52	0.87	0.19	0.13	0.01	0.03	0.29	77.79
RY04_1	46.26	3.26	7.18	0.42	0.55	0.80	0.19	0.38	0.02	0.03	0.26	59.35
RY04_2	59.38	2.70	6.03	0.28	0.37	0.93	0.21	0.43	0.01	0.04	0.11	70.48
RY05_1	75.14	4.00	6.14	0.24	0.56	0.73	0.19	0.20	0.01	0.03	0.12	87.35
RY05_2	42.18	3.62	8.39	0.21	0.62	0.77	0.22	0.42	0.01	0.02	0.30	56.77
RY06_1	52.95	2.93	1.79	0.28	0.35	0.87	0.16	0.16	0.01	0.02	0.06	59.58
RY06_2	62.58	2.80	2.53	0.32	0.43	0.79	0.17	0.28	0.02	0.04	0.05	70.02
RY07_1	47.71	2.37	1.68	0.20	0.44	0.73	0.18	0.17	0.01	0.03	0.09	53.61
RY07_2	74.04	3.89	1.98	0.29	0.44	0.72	0.23	0.33	0.01	0.03	0.05	82.01
RY08_1	54.68	2.55	1.68	0.26	0.37	0.74	0.25	0.27	0.01	0.02	0.06	60.90
RY08_2	59.20	3.53	2.22	0.28	0.43	0.83	0.16	0.17	0.01	0.03	0.10	66.95
RY09_1	39.65	3.38	7.75	0.22	0.55	1.12	0.25	0.42	0.01	0.02	0.35	53.72
RY09_2	70.41	2.84	6.70	0.32	0.46	0.69	0.25	0.20	0.01	0.03	0.05	81.97
RY10_1	70.92	2.73	1.90	0.28	0.48	0.98	0.20	0.27	0.01	0.03	0.08	77.89
RY10_2	45.71	3.37	2.29	0.33	0.38	0.89	0.18	0.21	0.01	0.03	0.06	53.47
RY11_1	44.24	3.26	6.83	0.22	0.42	0.77	0.24	0.22	0.01	0.03	0.11	56.34
RY11_2	74.18	3.08	9.18	0.35	0.67	0.85	0.21	0.27	0.01	0.03	0.26	89.09

Note: Other TE: a site involving mixed classes of transposable elements. VNTR: variable-number tandem repeat, a tandem repeat with the unit motif length ≥7bp. STR: short tandem repeat, a tandem repeat with the unit motif length ≤6bp. Other LCR: low-complexity regions with mixed VNTR/STR and low-complexity regions without a clear VNTR/STR pattern. Other repeat: a site involving mixed classes of repeats.

Supplementary Table 4 CPC-specific-CNV-related genes compared to HPRC assemblies

Gene	CPC Count (n=116)	HPRC.EAS	HPRC.nEAS	Archaic Proportion (%)	Tajima's D	Gene	CPC Count (n=116)	HPRC.EAS	HPRC.nEAS	Archaic Proportion (%)	Tajima's D
KCNJ18	115	×	×	0	NA	*MTRNR2L6*	115	×	×	0	-0.61
OR8U1	101	×	×	0	NA	*FRG2C*	74	×	×	0.0001	1.53
GOLGA8G	56	×	×	0	NA	*POTEH*	56	×	×	0	NA
FOXD4L4	51	×	×	0	NA	*OR2A7*	51	×	×	0	NA
PGA4	49	×	×	0.000324	NA	*GOLGA6B*	46	×	×	0	-1.91
GAGE12E	43	×	×	0	NA	*UPK3BL2*	42	×	×	0	NA
OR4F4	40	×	×	0	NA	*POLR2J2*	40	×	×	0	NA
GOLGA6L6	39	×	×	0	NA	*OR4K5*	37	×	×	0	NA
CTAGE4	36	×	×	0	NA	*SPANXA2*	35	×	×	0	NA
OR2A42	34	×	×	0	NA	*RFPL4AL1*	34	×	×	0	-0.78
CCL4L2	33	×	×	0	NA	*CLEC18B*	33	×	×	0	NA
CTAGE8	33	×	×	0	NA	*NPIPB15*	33	×	×	0	NA
NBPF19	30	×	○	0	NA	*DEFB103B*	28	×	×	0	NA
SLX1B	28	×	×	0	NA	*XAGE1B*	28	×	×	0	NA
GAGE12B	27	×	×	0	NA	*OR4M1*	27	×	○	0	NA
USP17L7	27	×	×	0	NA	*NOTCH2NLA*	24	×	×	0	NA
OR11H1	24	×	×	0	NA	*USP17L23*	24	×	×	0	NA
POTEM	22	×	×	0	NA	*CFC1*	21	×	○	0	NA
LGALS9C	21	×	×	0	NA	*NPIPA9*	21	×	×	0	NA
SPAG11B	21	×	×	0	NA	*SPDYE11*	21	×	×	0	NA
MBD3L3	19	×	×	0	0.38	*POTEG*	19	×	×	0	NA
USP17L22	19	×	×	0	NA	*BOLA2-SMG1P6*	18	×	×	0	NA
DEFA1B	18	×	×	0	NA	*NPIPA7*	18	×	×	0	NA
TRIM64B	18	×	×	0	NA	*GOLGA8F*	17	×	×	0	NA
PNMA6A	17	×	○	0	NA	*TBC1D3B*	17	×	×	0	NA
AMY1C	16	×	×	0	NA	*CTAGE6*	16	×	×	0	NA
ELOA3CP	16	×	×	0	1.83	*GAGE12G*	16	×	×	0	NA
HCAR2	16	×	×	0	NA	*OR4M2B*	16	×	×	0	NA
ZDHHC11B	16	×	×	0	2.16	*POLR2J3*	15	×	×	0	NA
SPANXB1	15	×	×	0	NA	*USP17L20*	15	×	×	0	NA
TBC1D3G	14	×	×	0	NA	*FCGR3B*	13	×	×	0	NA
GOLGA8K	13	×	×	0	NA	*NPIPB12*	13	×	○	0	NA
EIF3C	12	×	○	0	NA	*FOXD4L3*	12	×	×	0	NA
GSTT4	12	×	○	0	NA	*H4C15*	12	×	×	0	NA
OR11H12	12	×	×	0	NA	*OR4M2*	12	×	×	0	NA
OR4N2	12	×	○	0	NA	*OR4N4C*	12	×	×	0	NA
AMY1B	11	×	×	0	NA	*FAM90A23P*	11	×	×	0	NA
GOLGA6L4	11	×	○	0	NA	*HNRNPCL2*	11	×	×	0	NA
LIMS4	11	×	○	0	NA	*USP17L24*	11	×	×	0	NA
PRAMEF15	10	×	×	0	NA	*RIMBP3C*	10	×	×	0	NA
TBC1D3E	10	×	×	0	NA	*TRIM49*	10	×	×	0	NA
TRIM49D2	10	×	×	0	NA	*ELOA3D*	9	×	×	0	NA
GOLGA8N	9	×	○	0	NA	*GOLGA8O*	9	×	×	0	NA
GOLGA8R	9	×	×	0	NA	*H3C13*	9	×	×	0	NA
H3C14	9	×	×	0	NA	*OR4N4*	9	×	×	0	NA
OR52E5	9	×	×	0	NA	*TAF11L2*	9	×	×	0	NA
USP17L8	9	×	×	0	NA	*CBWD3*	8	×	×	0	NA
GAGE12H	8	×	×	0	NA	*H2AC19*	8	×	×	0	NA
LRRC37A2	8	×	×	0	NA	*OR4C46*	8	×	×	0	NA
OR52E4	8	×	×	0	3.01	*POTEB3*	8	×	×	0	NA
SERF1B	8	×	×	0	NA	*TAF11L10*	8	×	×	0	NA
TCP11X2	8	×	×	0	NA	*USP17L18*	8	×	×	0	NA
CT45A5	7	×	×	0	NA	*CTAG1A*	7	×	○	0	NA
ELOA2	7	×	×	0	2.81	*ELOA3*	7	×	×	0	NA
ELOA3B	7	×	×	0	NA	*F8A1*	7	×	×	0	NA
GAGE2A	7	×	×	0	NA	*H2AC20*	7	×	×	0	NA
H2AC21	7	×	×	0	NA	*MAGED4B*	7	×	×	0	NA
NPIPA8	7	×	×	0	NA	*NTAN1*	7	×	○	0	NA
PPIAL4D	7	×	×	0	NA	*PRAMEF10*	7	×	○	0	NA
PSAPL1	7	×	×	0	**4.32**	*RRN3*	7	×	○	0	NA
SSU72P2	7	×	×	0	0.11	*SSU72P5*	7	×	×	0	0.11
TRIM77	7	×	×	0	NA	*USP17L3*	7	×	×	0	NA
ABC7-42404400C24.1	6	×	○	0	NA	*ACTR1B*	6	×	×	0	NA
CEL	6	×	×	0	-0.59	*CENPVL2*	6	×	×	0	NA
COX5B	6	×	×	0	NA	*FAM25C*	6	×	×	0	NA
H2BC21	6	×	×	0	NA	*MBD3L2B*	6	×	×	0	NA
NPIPA2	6	×	×	0	NA	*NPIPB7*	6	×	×	0	NA
NXF2B	6	×	×	0	NA	*PRAMEF8*	6	×	○	0	NA
PWWP4	6	×	×	0	NA	*SPATA31D4*	6	×	×	0	**-3.01**
TAF11L4	6	×	×	0	NA	*TBC1D3H*	6	×	×	0	NA

Gene	CPC Count (n=116)	HPRC.EAS	HPRC.nEAS	Archaic Proportion (%)	Tajima's D	Gene	CPC Count (n=116)	HPRC.EAS	HPRC.nEAS	Archaic Proportion (%)	Tajima's D
TMEM191B	6	×	○	0	NA	*ZNF658*	6	×	○	0	NA
ARHGAP11A	5	×	×	0	NA	*CCNL2*	5	×	×	0	NA
CXorf51B	5	×	×	0	NA	*FAHD2B*	5	×	○	0	NA
GAGE12F	5	×	×	0	NA	*GOLGA6L9*	5	×	×	0	NA
H2AB1	5	×	×	0	NA	*H3-2*	5	×	○	0	NA
HBA2	5	×	×	0.0000289	0.23	*MRPL20*	5	×	×	0	NA
PRAMEF2	5	×	○	0	NA	*PRAMEF26*	5	×	×	0	NA
PRAMEF4	5	×	○	0	NA	*PRPF31*	5	×	×	0	NA
SPDYE13	5	×	○	0	NA	*SYT15*	5	×	○	0	NA
TAF11L13	5	×	×	0	NA	*TAF11L3*	5	×	×	0	NA
TAF11L6	5	×	×	0	NA	*TAF11L7*	5	×	×	0	NA
TAF11L8	5	×	×	0	NA	*TBC1D3K*	5	×	×	0	NA
TP53TG3D	5	×	×	0	NA	*TSPY1*	5	×	○	0	NA
USP17L17	5	×	×	0	NA	*USP17L19*	5	×	×	0	NA
ANKRD20A2P	4	×	×	0	NA	*AURKAIP1*	4	×	×	0	NA
BOLA1	4	○	×	0	NA	*C2orf92*	4	×	×	0	NA
CD99	4	×	×	0	NA	*CHRFAM7A*	4	×	○	0	NA
CRLF2	4	×	×	0	NA	*CSAG3*	4	×	×	0	NA
CT45A3	4	×	×	0	NA	*CTAG2*	4	×	×	0	NA
DGCR6	4	×	×	0	NA	*GOLGA6C*	4	×	×	0	NA
GPR148	4	×	×	0	NA	*HNRNPCL3*	4	×	×	0	NA
KIR2DL3	4	×	×	0	1.05	*MAGEA6*	4	×	×	0	NA
NBPF15	4	×	×	0	NA	*OR4A5*	4	×	×	0	NA
OR4C15	4	×	×	0	NA	*OR4K15*	4	×	×	0	NA
OR51A2	4	×	×	0	-0.72	*OR5W2*	4	×	×	0	NA
PGA5	4	×	×	0.000356	NA	*POTEJ*	4	×	×	0	NA
RBMY1E	4	×	○	0	NA	*SCG5*	4	×	×	0	NA
SMN2	4	×	×	0	NA	*SSX2*	4	×	○	0	NA
TAF11L9	4	×	×	0	NA	*USP17L13*	4	×	×	0	NA
ZAP70	4	×	×	0.000448	1.46	*ZNF468*	4	×	×	0	NA
ZNF92	4	×	×	0	NA	*AC007326.13*	3	×	×	0	NA
ADGRE1	3	×	×	0	-2.45	*ALKBH4*	3	×	×	0	NA
ANKRD20A4P	3	×	○	0	NA	*ANKRD36B*	3	○	×	0	NA
ANKRD65	3	×	×	0	NA	*C4B*	3	×	×	0.000716	NA
C8orf33	3	×	×	0	NA	*CATSPER2*	3	×	○	0	NA
CBWD5	3	×	×	0	NA	*DDTL*	3	×	○	0	NA
DEFA5	3	×	○	0	NA	*ERV3-1*	3	×	○	0	NA
FAHD2A	3	×	○	0	NA	*FAM110C*	3	×	×	0	NA
FAM178B	3	×	○	0	NA	*FAM236B*	3	×	×	0	NA
FAM236D	3	×	×	0	NA	*FAM246A*	3	×	×	0	NA
FAM83G	3	×	×	0	NA	*FAM86B2*	3	×	×	0	NA
FCGR3A	3	×	×	0	NA	*FOXD4L6*	3	×	×	0	NA
GRAPL	3	×	×	0	NA	*GTPBP6*	3	×	×	0	NA
GZMM	3	×	×	0	NA	*HIC2*	3	×	○	0	NA
HSPA6	3	×	×	0	1.6	*IZUMO2*	3	×	○	0	NA
LILRA5	3	×	×	0	NA	*LIMS3*	3	×	○	0	NA
LRWD1	3	×	×	0	NA	*MAGEA2*	3	×	×	0	NA
MAGEA3	3	×	×	0	NA	*MAGEH1*	3	×	×	0	NA
MPV17L2	3	×	×	0	NA	*NDUFA3*	3	×	×	0	NA
OR10AG1	3	×	×	0	NA	*OR2T29*	3	×	×	0	1.6
OR2Z1	3	×	×	0	NA	*OR4A15*	3	×	×	0	NA
OR4A16	3	×	×	0	NA	*OR4C12*	3	×	×	0	NA
OR4C13	3	×	×	0	NA	*OR4C16*	3	×	×	0	NA
OR4K13	3	×	×	0	NA	*OR4K14*	3	×	×	0	NA
OR4L1	3	×	×	0	NA	*OR5D13*	3	×	×	0	NA
OR5D14	3	×	×	0	NA	*OR5D16*	3	×	×	0	NA
OR5D18	3	×	×	0	NA	*OR5D3P*	3	×	×	0	NA
OR5F1	3	×	×	0	NA	*OR5I1*	3	×	×	0	NA
OR5L1	3	×	×	0	NA	*OR5L2*	3	×	×	0	NA
ORAI2	3	×	×	0	NA	*OSCAR*	3	×	×	0	NA
PRAG1	3	×	×	0	NA	*PRAMEF27*	3	×	○	0	NA
PRAMEF9	3	×	×	0	NA	*PRODH*	3	×	×	0	NA
RP11-69H14.10	3	×	×	0	NA	*RPIA*	3	×	○	0	NA
SCGB1C1	3	×	×	0	NA	*SEMA4C*	3	×	○	0	NA
SLC16A8	3	×	○	0	NA	*SLC5A10*	3	×	○	0	NA
SMPDL3B	3	×	×	0	NA	*SPDYE6*	3	×	×	0	-1.35
SULT2A1	3	×	○	0	NA	*TAF11L11*	3	×	×	0	NA
TBC1D3D	3	×	×	0	NA	*TBC1D3L*	3	×	×	0	NA
TCAF2C	3	×	×	0	NA	*TFPT*	3	×	×	0	NA
TNFRSF6B	3	×	×	0	NA	*TRIM43B*	3	×	×	0	NA
TRIM49C	3	×	×	0	NA	*TRIM51GP*	3	×	×	0	NA
TSEN34	3	×	○	0	NA	*USP17L10*	3	×	×	0	NA
USP17L25	3	×	×	0	NA	*USP51*	3	×	○	0	NA

Gene	CPC Count (n=116)	HPRC.EAS	HPRC.nEAS	Archaic Proportion (%)	Tajima's D	Gene	CPC Count (n=116)	HPRC.EAS	HPRC.nEAS	Archaic Proportion (%)	Tajima's D
VAMP7	3	×	×	0	NA	*XKR8*	3	×	×	0	NA
YTHDF1	3	×	×	0	3.18	*ZNF117*	3	×	×	0	NA
ZNF14	3	×	×	0	3.01	*ACTL9*	2	×	×	0	NA
AMY2B	2	×	×	0	NA	*ANKRD36*	2	×	○	0	NA
ANKRD39	2	×	×	0	-0.98	*ANKS4B*	2	×	×	0	NA
ANTXRL	2	×	○	0	NA	*ANXA8L1*	2	×	×	0	NA
APOBR	2	×	○	0	NA	*ARFRP1*	2	×	×	0	NA
ARHGAP11B	2	×	×	0	NA	*ASL*	2	×	×	0	-1.96
ATAD3C	2	×	×	0	NA	*BFAR*	2	×	○	0	NA
BIRC7	2	×	×	0	NA	*BSPH1*	2	×	○	0	NA
BTN3A1	2	×	×	0.00045	NA	*C17orf99*	2	×	×	0	3.11
CBWD6	2	○	×	0	NA	*CCDC116*	2	×	×	0	NA
CCDC74B	2	×	×	0	**-2.71**	*CCR5*	2	×	×	0.000183	2.2
CCRL2	2	×	×	0.000202	**4.07**	*CDC34*	2	×	×	0	NA
CDK11B	2	×	○	0	NA	*CHORDC1*	2	×	×	0	1.72
CLDN23	2	×	×	0	NA	*CLEC4G*	2	×	×	0	1.27
CLN3	2	×	○	0	NA	*CNNM3*	2	×	○	0	NA
CNNM4	2	×	○	0	NA	*CRX*	2	×	○	0	NA
CRYM	2	×	×	0	NA	*CSAG1*	2	×	×	0	NA
CT47A10	2	×	×	0	NA	*CT55*	2	×	○	0	NA
CYP21A2	2	×	○	0	NA	*CYP2C9*	2	×	×	0.00179	-2.7
DAP3	2	×	×	0	NA	*DDIT4L*	2	×	×	0	0.29
DEFA4	2	×	×	0	NA	*DEFA6*	2	×	×	0	NA
DEFB1	2	×	×	0	NA	*DHFR2*	2	×	×	0	NA
DHRS11	2	×	×	0	NA	*DHRS2*	2	×	×	0	3.11
DHRS4L2	2	×	×	0	1.51	*DMRTC1B*	2	×	○	0	NA
EHD2	2	×	×	0	NA	*EIF2AK3*	2	×	○	0	NA
EIF3CL	2	×	○	0	NA	*ELSPBP1*	2	×	×	0	NA
EMB	2	×	○	0	NA	*EML2*	2	×	×	0	2.34
EPHA8	2	×	×	0	1.99	*ETDC*	2	×	×	0	NA
FAM72B	2	×	○	0	NA	*FAM72C*	2	×	○	0	NA
FAM72D	2	×	×	0	NA	*FAM90A9P*	2	×	×	0	NA
FAM9A	2	×	×	0	NA	*FAN1*	2	×	×	0	NA
FGFBP1	2	×	×	0	1.85	*FOLH1*	2	×	×	0	NA
FOXD4L5	2	×	×	0	NA	*FOXI3*	2	×	○	0	NA
GGNBP2	2	×	×	0	NA	*GGT2*	2	×	×	0	NA
GOLGA6L22	2	×	×	0	NA	*GSPT2*	2	×	×	0	NA
GUSB	2	×	×	0	NA	*H2AB2*	2	×	×	0	NA
H3Y1	2	×	×	0	NA	*HNRNPCL1*	2	×	○	0	NA
HSFX2	2	×	○	0	NA	*IGHV3OR16-17*	2	×	×	0	NA
IGSF6	2	×	○	0	NA	*IL27*	2	×	○	0	NA
IL9R	2	×	×	0	NA	*KANSL1*	2	×	×	0	NA
LILRB3	2	×	×	0	NA	*LILRB5*	2	×	○	0	NA
LIME1	2	×	×	0	-0.13	*LMAN2L*	2	×	○	0	NA
MAGEA12	2	×	×	0	NA	*MAGEA2B*	2	×	×	0	NA
MAPT	2	×	×	0	NA	*MRM1*	2	×	×	0	NA
MRM3	2	×	×	0.000353	-1.11	*MT4*	2	×	×	0	NA
MTMR10	2	×	×	0	NA	*MTRNR2L8*	2	×	×	0	NA
MYO19	2	×	×	0	NA	*NAALAD2*	2	×	×	0	1.57
NBPF11	2	×	×	0	NA	*NBPF4*	2	×	×	0	0.64
NDUFAF8	2	×	○	0	NA	*NECAP2*	2	○	×	0	NA
NMB	2	×	○	0	NA	*NOP53*	2	×	×	0	NA
NOTCH2NLC	2	×	○	0	NA	*NUPR1*	2	×	○	0	NA
NUTM2B	2	×	×	0	NA	*OR11H2*	2	×	×	0	NA
OR2A4	2	×	×	0	1.25	*OR2F1*	2	×	×	0	NA
OR2F2	2	×	×	0	NA	*OR2V1*	2	×	×	0	NA
OR2V2	2	×	×	0	NA	*OR4K17*	2	×	×	0	NA
OR56A3	2	×	×	0	NA	*OR56A5*	2	×	×	0	**3.92**
OR5AS1	2	×	×	0	NA	*OR5J2*	2	×	×	0	NA
OR5T2	2	×	×	0	NA	*OR5T3*	2	×	×	0	NA
OR6B1	2	×	×	0	NA	*OR8H2*	2	×	×	0	NA
OR8H3	2	×	×	0	NA	*OR8I2*	2	×	×	0	NA
OR8J3	2	×	×	0	NA	*OR8K5*	2	×	×	0	NA
PCDHB16	2	×	○	0	NA	*PCDHB8*	2	×	×	0	1.43
PDXDC1	2	×	○	0	NA	*PGBD2*	2	×	×	0	-1.53
PIAS2	2	×	×	0	2.81	*PIGW*	2	×	×	0	NA
PRRT4	2	×	×	0.0000925	-0.1	*PTCHD1*	2	×	×	0	NA
RIMBP3	2	×	×	0	NA	*RNPC3*	2	×	×	0	NA
RP1-321E8.5	2	×	×	0	NA	*RP11-315A19.1*	2	×	○	0	NA
RP11-353J17.5	2	×	○	0	NA	*RP11-435I10.4*	2	×	○	0	NA
RP11-736I24.5	2	×	×	0	NA	*RP4-583P15.14*	2	×	×	0	NA
RP4-583P15.15	2	×	×	0	NA	*RPL35A*	2	×	×	0	0.63

Gene	CPC Count (n=116)	HPRC.EAS	HPRC.nEAS	Archaic Proportion (%)	Tajima's D	Gene	CPC Count (n=116)	HPRC.EAS	HPRC.nEAS	Archaic Proportion (%)	Tajima's D
RPS10	2	×	×	0.000299	**4.18**	RPS12	2	×	×	0.000104	-1.37
RPS17	2	×	×	0	NA	RPS9	2	×	×	0	NA
SCCPDH	2	×	×	0	NA	SDF2L1	2	×	×	0	NA
SEC22B	2	×	×	0	NA	SELENOW	2	×	○	0	NA
SGCA	2	×	×	0.000407	-0.51	SH2D2A	2	×	×	0	3.11
SHOX	2	×	×	0	NA	SLC2A4RG	2	×	×	0	0.16
SPANXN5	2	×	×	0	NA	SPDYE21	2	×	×	0	NA
SSU72P3	2	×	×	0	-1.39	SSU72P4	2	×	×	0	NA
STH	2	×	×	0	NA	STRC	2	×	○	0	NA
SYNGR2	2	×	×	0	2.71	TAF11L12	2	×	×	0	NA
TAF11L14	2	×	×	0	NA	TBC1D3I	2	×	○	0	NA
TEX37	2	×	○	0	NA	TEX38	2	×	×	0.000164	**4.49**
TMC8	2	×	×	0	1.88	TMEM106B	2	×	×	0	NA
TMEM131	2	×	×	0.00831	2.6	TMSB15A	2	×	×	0	NA
TPRX1	2	×	○	0	NA	TPSB2	2	×	○	0	NA
TRIM49B	2	×	×	0	NA	TRIM64C	2	×	×	0	NA
TRIML1	2	×	○	0	NA	USP17L1	2	×	×	0	NA
USP17L11	2	×	×	0	NA	USP17L15	2	×	×	0	NA
USP17L4	2	×	×	0	NA	VWA1	2	×	×	0	NA
VWDE	2	×	×	0	NA	WDR73	2	×	○	0	NA
YDJC	2	×	×	0	NA	YY1AP1	2	×	×	0	NA
ZBED1	2	×	×	0	NA	ZGPAT	2	×	×	0	NA
ZNF138	2	×	×	0	NA	ZNF273	2	×	×	0	NA
ZNF333	2	×	×	0	2.81	ZNF33B	2	×	×	0	NA
ZNF705G	2	×	×	0	NA	ZNHIT3	2	×	×	0	NA
ZSCAN2	2	×	○	0	NA	ZSWIM9	2	×	×	0	NA
ABHD17A	1	×	×	0	-0.62	ABT1	1	×	○	0	NA
AC006486.9	1	×	×	0	NA	AC008079.12	1	×	×	0	NA
AC068533.7	1	×	×	0	NA	ACADVL	1	×	×	0	-1.04
ACAP3	1	×	×	0	NA	ACBD4	1	×	×	0	3.7
ACP5	1	×	×	0	NA	ACSM2B	1	×	×	0	NA
ACTRT2	1	×	×	0.0000498	1.97	ADAM30	1	×	×	0	NA
ADAMTS10	1	×	×	0	NA	ADAMTSL5	1	×	×	0	**4.01**
AGPAT2	1	○	×	0	NA	AGPAT5	1	×	×	0	NA
AJUBA	1	×	×	0	-0.12	AKAP17A	1	×	×	0	NA
AKAP5	1	×	×	0	3.04	AL928654.7	1	×	○	0	NA
ALG10	1	×	○	0	NA	ALG10B	1	×	×	0.00045	NA
ALPG	1	×	×	0	NA	ALPI	1	×	×	0	NA
ALPK3	1	×	×	0.00195	-1.16	ALPP	1	×	×	0	NA
AMER2	1	×	×	0.000368	-0.69	AMER3	1	×	×	0	NA
AMY2A	1	×	×	0	NA	ANGEL1	1	×	×	0	**3.53**
ANKRD20A1	1	×	×	0	NA	AP000471.4	1	×	×	0	NA
AQP12B	1	×	○	0	NA	ARGLU1	1	×	×	0	-0.17
ARL14EPL	1	×	×	0	-0.56	ARMCX5	1	×	×	0	NA
ASS1	1	×	×	0	-0.28	ASXL1	1	×	○	0	NA
ATF7	1	×	×	0	NA	ATF7-NPFF	1	×	×	0	NA
ATP12A	1	×	×	0.000203	1.83	ATP13A1	1	×	×	0	-0.91
ATP13A2	1	×	×	0	-1.24	ATP6V0E2	1	○	×	0	NA
AURKA	1	×	×	0	2.74	B3GALT6	1	×	×	0	NA
B4GALT7	1	×	×	0	0.47	BAHD1	1	×	×	0.00099	NA
BBS5	1	×	○	0	NA	BDH1	1	×	×	0	NA
BDP1	1	×	○	0	NA	BEST2	1	×	×	0	-2.33
BHLHA9	1	×	×	0	NA	BHMT	1	○	×	0	NA
BICRA	1	×	×	0	NA	BRD9	1	×	×	0	NA
BTN1A1	1	×	×	0.000351	-1.21	BTN2A1	1	×	×	0.00065	-1.5
BTN2A2	1	×	×	0.000409	NA	BTN3A2	1	×	×	0.000457	-0.8
BTN3A3	1	×	×	0.000449	-1.5	C11orf42	1	×	×	0	-1.66
C13orf46	1	×	×	0	NA	C15orf39	1	×	×	0	NA
C17orf64	1	×	×	0	1.68	C1QTNF12	1	×	×	0	NA
C2	1	×	×	0.00166	-2.07	C22orf39	1	×	×	0	NA
C2CD4C	1	×	×	0	0.2	C4orf48	1	×	×	0	3.4
C8orf58	1	×	×	0	NA	C8orf74	1	×	×	0	-1.91
CA4	1	×	×	0	NA	CABP5	1	×	×	0	NA
CACNA1B	1	×	×	0	NA	CALML5	1	×	×	0	NA
CALY	1	×	×	0	NA	CAPNS1	1	×	×	0	NA
CAPNS2	1	×	×	0.0000348	-0.43	CASP1	1	×	×	0	3.07
CASP5	1	×	×	0	3.07	CASS4	1	×	×	0	**4.32**
CBR1	1	×	×	0	NA	CCDC115	1	×	×	0	NA
CCDC125	1	×	×	0	NA	CCDC17	1	×	×	0.000139	-0.23
CCKBR	1	×	×	0	-1.59	CCL11	1	×	×	0	0.37
CCL2	1	×	×	0	NA	CCL3	1	×	×	0.0000664	2
CCL7	1	×	×	0	NA	CCN5	1	×	×	0.0000638	-1.05
CCNB1	1	×	×	0	NA	CCT6A	1	×	×	0.000427	NA

Gene	CPC Count (n=116)	HPRC.EAS	HPRC.nEAS	Archaic Proportion (%)	Tajima's D	Gene	CPC Count (n=116)	HPRC.EAS	HPRC.nEAS	Archaic Proportion (%)	Tajima's D
CCT8L2	1	×	×	0	2.16	CD177	1	×	×	0	-0.99
CD1B	1	×	×	0	-0.55	CD1E	1	×	×	0	-1.29
CD276	1	×	×	0	-0.57	CD2BP2	1	○	×	0	NA
CDC16	1	×	×	0.00131	-0.45	CDC45	1	×	×	0	NA
CDK7	1	×	×	0	NA	CDKL4	1	×	×	0	1.71
CDR2	1	×	×	0	NA	CEACAM6	1	×	×	0	NA
CEBPD	1	×	×	0	NA	CELA3B	1	×	×	0	1.61
CELSR2	1	×	○	0	NA	CENPH	1	×	×	0	NA
CENPJ	1	×	×	0.00141	0.46	CENPL	1	×	×	0	NA
CENPVL3	1	×	×	0	NA	CES1	1	×	○	0	NA
CES3	1	×	×	0	-0.91	CFAP126	1	×	×	0	2.72
CFAP20	1	×	×	0	NA	CFAP410	1	×	×	0	NA
CFAP45	1	×	×	0	1.03	CFB	1	×	×	0.000223	-1.88
CFHR1	1	×	×	0.000605	0.68	CFHR4	1	×	×	0.00107	-2.24
CFL2	1	×	×	0	0.45	CGB2	1	×	×	0	-1.96
CGRRF1	1	×	×	0	2.66	CH17-159N18.5	1	×	○	0	NA
CHAC2	1	×	×	0	0.35	CHAMP1	1	×	×	0	-1.24
CHCHD2	1	×	×	0.00017	NA	CHMP1A	1	×	×	0	NA
CHMP2A	1	×	×	0	NA	CHRAC1	1	×	×	0	NA
CHST14	1	×	×	0.0000755	NA	CINP	1	×	×	0.000757	**3.72**
CKMT1B	1	×	×	0	NA	CLDN5	1	×	×	0	NA
CLTCL1	1	×	×	0	NA	CNGA4	1	×	×	0	-1.63
CNIH1	1	×	×	0	**4.03**	COL1A1	1	×	×	0.000276	-0.74
COL6A1	1	×	○	0	NA	COL6A2	1	×	○	0	NA
COL9A3	1	×	×	0	NA	COX7A1	1	×	×	0	NA
CPLANE2	1	×	×	0.000191	NA	CRCP	1	×	×	0.00139	-0.85
CRHR1	1	×	×	0	NA	CRIP1	1	×	○	0	NA
CRIP2	1	×	○	0	NA	CSAG2	1	×	×	0	NA
CSNK2A2	1	×	×	0	NA	CSTF1	1	×	×	0	3.27
CT45A7	1	×	×	0	NA	CTD-2587H24.4	1	×	×	0	NA
CTD-3051L14.15	1	×	×	0	NA	CTLA4	1	×	×	0	0.58
CUEDC2	1	×	×	0	-1.59	CYHR1	1	×	×	0	NA
CYP2A6	1	×	○	0	NA	CYP46A1	1	×	×	0	NA
DARS2	1	×	×	0	NA	DBNDD1	1	×	×	0	NA
DCAF16	1	×	×	0.000351	-0.11	DCTD	1	×	×	0	1.63
DCUN1D4	1	×	○	0	NA	DDX41	1	×	×	0	-1.13
DDX51	1	×	×	0.000268	-1.67	DECR2	1	×	×	0.000143	-0.6
DEF8	1	×	×	0	NA	DEFA3	1	×	×	0	NA
DEFB105A	1	×	○	0	NA	DEFB126	1	×	×	0	0.81
DGCR2	1	×	×	0	NA	DGKE	1	×	×	0	0.98
DHH	1	×	×	0	0.2	DHODH	1	×	×	0	NA
DHRS4	1	×	×	0	1.51	DHX36	1	×	×	0	-0.8
DNAAF3	1	×	×	0	-0.64	DOK3	1	×	×	0	-1.13
DPF2	1	×	×	0	-1.8	DXO	1	×	×	0.0000861	-1.03
DYRK4	1	×	×	0	3.08	E2F4	1	×	×	0	-1.93
EAPP	1	×	×	0	NA	EBAG9	1	×	×	0	-2.43
ECEL1	1	×	×	0	NA	EDEM2	1	×	×	0	-1.27
EEF1A2	1	×	×	0.00039	-0.78	EEF2K	1	×	×	0	NA
EFNA2	1	×	×	0	NA	EGFL7	1	○	×	0	NA
EHMT2	1	×	×	0.000623	-1.64	EID3	1	×	×	0	NA
EIF5A2	1	×	×	0	-0.07	ELMO3	1	×	×	0	-1.22
ELOB	1	×	×	0	-0.21	ENSG00000283886	1	×	×	0	NA
EPHB3	1	×	○	0	NA	EPN1	1	×	○	0	NA
EPPIN	1	×	×	0	-1.08	ERF	1	×	×	0	-1.93
ERI1	1	×	×	0	NA	ERLEC1	1	×	×	0	-0.45
ERVV-1	1	×	×	0	NA	ESS2	1	×	×	0	NA
EXOC3L1	1	×	×	0	-2.55	EYA3	1	×	×	0	-0.91
F3	1	×	×	0	NA	FAM107A	1	×	×	0	1.76
FAM153A	1	×	×	0	2.78	FAM160A2	1	×	×	0	NA
FAM193B	1	×	×	0	-1.13	FAM209A	1	×	×	0	3.04
FAM209B	1	×	×	0	3.04	FAM210B	1	×	×	0	2.35
FAM240C	1	×	×	0.000297	NA	FAM25G	1	×	×	0	NA
FAM3A	1	×	×	0	NA	FAM50A	1	×	×	0	NA
FAM83E	1	×	×	0	-0.71	FAM90A26	1	×	×	0	NA
FASTKD1	1	×	○	0	NA	FBXL15	1	×	×	0	-1.49
FBXO42	1	×	×	0.00367	NA	FBXW10	1	×	×	0	NA
FCER1A	1	×	×	0.000642	0.55	FCGR1A	1	×	○	0	NA
FCGR1B	1	×	×	0	NA	FGF21	1	×	×	0	3.71
FGFBP2	1	×	×	0	2.32	FLII	1	×	×	0	-1.8
FMC1	1	×	×	0	1.85	FNDC10	1	×	×	0	NA
FOXD3	1	×	×	0	-0.36	FOXL3	1	×	×	0	NA
FRG2B	1	×	×	0	NA	FRMD8	1	×	×	0	-2.07
FSCB	1	×	×	0.000109	**4.26**	FUOM	1	×	×	0	NA
FUT1	1	×	×	0	3.71	FUZ	1	×	×	0	-1.04

Gene	CPC Count (n=116)	HPRC.EAS	HPRC.nEAS	Archaic Proportion (%)	Tajima's D	Gene	CPC Count (n=116)	HPRC.EAS	HPRC.nEAS	Archaic Proportion (%)	Tajima's D
FZD2	1	×	×	0	NA	*G6PD*	1	×	×	0	NA
GABRD	1	×	○	0	NA	*GADD45B*	1	×	×	0	-0.42
GALE	1	×	×	0	-1.66	*GAS8*	1	×	×	0	NA
GCNT7	1	×	×	0	3.04	*GDF10*	1	×	×	0	NA
GDF2	1	×	×	0	NA	*GDPD3*	1	×	×	0	NA
GET4	1	×	×	0	NA	*GGT5*	1	×	×	0	**4.54**
GGTLC2	1	×	×	0	NA	*GGTLC3*	1	×	○	0	NA
GMFB	1	×	×	0	3.18	*GMIP*	1	×	×	0	-0.91
GOLGA6D	1	×	×	0	-1.81	*GPER1*	1	×	×	0	NA
GPR12	1	×	×	0.000194	-2.16	*GPR42*	1	×	×	0.0000657	-0.35
GPR65	1	×	×	0.000336	-1.35	*GPRASP1*	1	×	×	0	NA
GPRASP2	1	×	×	0	NA	*GREM1*	1	×	×	0	NA
GSC2	1	×	×	0	NA	*GSTA3*	1	×	×	0	NA
GSTA5	1	×	×	0	NA	*GSX1*	1	×	○	0	NA
GTF2IRD2B	1	×	×	0	NA	*GTF3C6*	1	×	×	0	2.44
H2AC11	1	×	×	0.0000171	-2.47	*H2BC18*	1	×	○	0	NA
H2BW2	1	×	×	0	NA	*H3Y2*	1	×	×	0	NA
HAUS4	1	×	×	0	NA	*HBA1*	1	×	×	0.0000292	-0.89
HBQ1	1	×	×	0.000025	-0.89	*HDGFL1*	1	×	×	0.0000729	-0.19
HDHD2	1	×	×	0	3.79	*HEXIM1*	1	×	×	0	3.7
HEXIM2	1	×	×	0	2.3	*HHATL*	1	×	×	0	-0.34
HHIPL1	1	×	×	0	NA	*HIC1*	1	×	×	0	NA
HILPDA	1	×	×	0.0000892	-1.02	*HIRA*	1	×	×	0	NA
HMGN4	1	×	×	0.000297	-0.51	*HRAS*	1	×	×	0.000176	NA
HSBP1L1	1	×	×	0	-0.24	*HSPA1A*	1	×	×	0.0000834	-1.61
HSPA1B	1	×	×	0.0000873	-1.5	*HSPA1L*	1	×	×	0.000197	-1.61
HSPA2	1	×	×	0	2.34	*HTR6*	1	×	×	0.000559	1.15
HYPK	1	×	×	0	-0.54	*IER3IP1*	1	×	×	0	2.96
IFNL3	1	×	×	0	NA	*IGLL5*	1	×	×	0	NA
IMP4	1	×	×	0	NA	*IMPDH1*	1	×	×	0.000624	-0.81
INSYN1	1	×	×	0	-0.82	*INTS14*	1	×	×	0	-1.97
ISLR	1	×	×	0.00011	-1.37	*ISLR2*	1	×	○	0	NA
IZUMO1	1	×	×	0	3.71	*JMY*	1	×	×	0	2.87
JOSD2	1	×	×	0	-0.51	*JUP*	1	×	×	0	NA
KAT14	1	×	×	0	-1.1	*KCNF1*	1	×	×	0.0000333	-0.69
KCNK15	1	×	×	0	0.88	*KCNT1*	1	×	×	0	3.76
KCTD5	1	×	×	0	-2.13	*KCTD7*	1	×	×	0.000701	NA
KIF5B	1	×	×	0	NA	*KLF13*	1	×	×	0	-0.82
KLHDC7A	1	×	×	0	-0.64	*KLHL20*	1	×	×	0	NA
KLHL40	1	×	×	0	-0.99	*KLHL41*	1	×	○	0	NA
KLK6	1	×	×	0	-1.33	*KLK7*	1	×	×	0	1.39
KPTN	1	×	×	0	NA	*KRT18*	1	×	×	0.00014	0.38
LAGE3	1	×	×	0	NA	*LAIR1*	1	×	○	0	NA
LAPTM5	1	×	×	0	NA	*LCE2D*	1	×	×	0	1.26
LCN15	1	×	○	0	NA	*LHFPL4*	1	×	×	0.00193	-1.57
LIG1	1	×	×	0	NA	*LILRB2*	1	×	×	0	NA
LINC02210-CRHR1	1	×	×	0	NA	*LINC02218*	1	×	×	0	-1.61
LMBR1L	1	×	×	0	0.63	*LMNB2*	1	×	×	0	NA
LMNTD2	1	×	×	0.0012	1.8	*LONRF1*	1	×	×	0	NA
LOXL1	1	×	×	0	2.7	*LPAR2*	1	×	×	0	-0.91
LPCAT2	1	×	×	0.00269	1.48	*LRP10*	1	×	×	0	1.41
LRRC56	1	×	×	0.000603	1.8	*LRRC66*	1	×	×	0	NA
LRRC75B	1	×	×	0	NA	*LRRFIP2*	1	×	×	0	-2.06
LSM2	1	×	×	0.000332	-1.61	*LSM8*	1	×	×	0	0
LSS	1	×	○	0	NA	*LYPLA2*	1	×	×	0	-1.66
MAGEA1	1	×	×	0	NA	*MAGEA11*	1	×	×	0	NA
MAP3K12	1	×	×	0	NA	*MAPK1*	1	×	×	0	NA
MAPK3	1	×	×	0	NA	*MARVELD2*	1	×	×	0	NA
MATN1	1	×	×	0	NA	*MBD3L5*	1	×	×	0	NA
MC1R	1	×	×	0	NA	*MCCC2*	1	×	○	0	NA
MCM4	1	×	×	0	NA	*MCRIP2*	1	×	×	0.000405	0
METTL26	1	×	×	0.0000673	-0.58	*METTL2B*	1	×	×	0.00104	-1.1
METTL9	1	×	○	0	NA	*MFHAS1*	1	×	×	0	NA
MIEF2	1	×	×	0	-1.5	*MIER2*	1	×	×	0	-1.33
MMP14	1	×	×	0	1.71	*MMP2*	1	×	×	0.00318	-1.81
MMP9	1	×	×	0.000266	0.86	*MOK*	1	×	×	0.0028	1.26
MPG	1	×	×	0	-0.19	*MPV17L*	1	×	○	0	NA
MRPL28	1	×	×	0.00015	-0.4	*MRPL40*	1	×	×	0	NA
MRPL52	1	×	×	0	**3.56**	*MRPS36*	1	×	×	0	NA

Gene	CPC Count (n=116)	HPRC.EAS	HPRC.nEAS	Archaic Proportion (%)	Tajima's D	Gene	CPC Count (n=116)	HPRC.EAS	HPRC.nEAS	Archaic Proportion (%)	Tajima's D
MTHFD1	1	×	×	0	3.04	*MTLN*	1	×	○	0	NA
MTMR6	1	×	×	0.00143	-0.87	*MTRNR2L12*	1	×	×	0	NA
MYBPHL	1	×	×	0	1.86	*MYEOV*	1	×	○	0	NA
MZF1	1	×	×	0	NA	*MZT2A*	1	×	×	0	-2.55
NAPA	1	×	×	0	NA	*NAT8L*	1	×	×	0	3.44
NBPF10	1	×	×	0	NA	*NBPF14*	1	×	×	0	NA
NBPF20	1	×	×	0	NA	*NBPF6*	1	×	×	0	NA
NELFE	1	×	×	0.000241	-1.36	*NEU1*	1	×	×	0	-1.54
NFAT5	1	×	×	0	3.59	*NFILZ*	1	×	×	0	NA
NFKB2	1	×	×	0	NA	*NKD2*	1	×	×	0	**4.55**
NKTR	1	×	×	0	-0.77	*NME8*	1	×	×	0.000943	**4.41**
NMI	1	×	×	0	**5.28**	*NOB1*	1	×	○	0	NA
NOBOX	1	×	×	0	-2.19	*NOC3L*	1	×	×	0	-1.56
NOP14	1	×	×	0	NA	*NOTCH2NLR*	1	×	○	0	NA
NPFF	1	×	×	0	NA	*NPHP1*	1	×	○	0	NA
NPIPB3	1	×	×	0	NA	*NPIPB6*	1	×	×	0	NA
NPIPB9	1	×	×	0	NA	*NPTN*	1	×	×	0	**3.97**
NQO1	1	×	×	0	3.64	*NRGN*	1	×	×	0	-1.17
NSUN3	1	×	×	0	2.76	*NSUN5*	1	×	○	0	NA
NUP58	1	×	×	0.00222	-1.62	*NUPR2*	1	×	×	0.0000603	NA
NUTM2D	1	×	×	0	-1.25	*NUTM2G*	1	×	×	0.000415	NA
OCLN	1	×	×	0	NA	*ODAPH*	1	×	×	0	1.22
ODF2L	1	×	×	0	NA	*ODF3L2*	1	×	×	0.000404	1.31
OPN1MW3	1	×	○	0	NA	*OR10A6*	1	×	×	0	0.29
OR13F1	1	×	×	0	2.02	*OR13G1*	1	×	×	0	1.75
OR13J1	1	×	×	0	0.25	*OR1E1*	1	×	×	0.0000453	-0.39
OR1F1	1	×	×	0	NA	*OR1G1*	1	×	×	0	NA
OR1I1	1	×	×	0	-1.01	*OR2A12*	1	×	×	0	NA
OR2A14	1	×	×	0	NA	*OR2A2*	1	×	×	0	NA
OR2A25	1	×	×	0	NA	*OR2A5*	1	×	×	0	NA
OR2T3	1	×	×	0	-0.54	*OR2T34*	1	×	×	0	1.32
OR4A47	1	×	×	0	NA	*OR4B1*	1	×	×	0	NA
OR4C3	1	×	×	0	NA	*OR4C5*	1	×	×	0	NA
OR4F15	1	×	×	0	NA	*OR4F6*	1	×	×	0	NA
OR4N5	1	×	×	0	-0.91	*OR4S1*	1	×	×	0	NA
OR4X1	1	×	×	0	NA	*OR4X2*	1	×	×	0	NA
OR52L1	1	×	×	0	**4.09**	*OR56A4*	1	×	×	0	**4.09**
OR5AP2	1	×	×	0	NA	*OR5AR1*	1	×	×	0	NA
OR5M1	1	×	×	0	NA	*OR5M10*	1	×	×	0	NA
OR5M11	1	×	×	0	NA	*OR5M3*	1	×	×	0	NA
OR5M8	1	×	×	0	NA	*OR5M9*	1	×	×	0	NA
OR5T1	1	×	×	0	NA	*OR8H1*	1	×	×	0	NA
OR8J1	1	×	×	0	NA	*OR8K1*	1	×	×	0	NA
OR8K3	1	×	×	0	NA	*OR8U3*	1	×	×	0	NA
ORM2	1	×	×	0.000117	-1.31	*OTOA*	1	×	○	0	NA
P2RX1	1	×	×	0	-0.93	*P2RX3*	1	×	×	0.000494	**3.87**
P3H4	1	×	×	0	NA	*PABPC3*	1	×	×	0	-0.3
PARN	1	×	○	0	NA	*PARP8*	1	×	×	0	-1.5
PCNA	1	×	×	0	2.63	*PCSK4*	1	×	×	0	NA
PDCD1	1	×	×	0.000313	-1.01	*PDF*	1	×	×	0	-1.93
PDLIM7	1	×	×	0	-1.12	*PDX1*	1	×	○	0	NA
PET117	1	×	×	0	0.18	*PEX26*	1	×	×	0	NA
PGLYRP1	1	×	×	0	NA	*PGM5*	1	×	×	0	NA
PHKG1	1	×	×	0.000441	NA	*PIM3*	1	×	×	0	-0.46
PITHD1	1	×	×	0	-1.66	*PIWIL3*	1	×	×	0	NA
PKP3	1	×	×	0	NA	*PLA2G10*	1	×	○	0	NA
PLA2G4C	1	×	×	0	NA	*PLCD3*	1	×	×	0	2.71
PLCXD1	1	×	×	0	NA	*PLGLB2*	1	×	×	0	NA
PLIN4	1	×	×	0	NA	*PLIN5*	1	×	×	0	NA
PLK5	1	×	×	0	**4.01**	*PLXNA3*	1	×	×	0	NA
PML	1	×	×	0	3.24	*PNMA6F*	1	×	×	0	NA
POLR3E	1	×	×	0	NA	*POU4F1*	1	×	×	0	-2.47
PPDPF	1	×	×	0.00005	0.75	*PPIAL4C*	1	×	×	0	NA
PPIAL4G	1	×	×	0	NA	*PPIAL4H*	1	×	×	0	NA
PPIL2	1	×	×	0	NA	*PPM1F*	1	×	×	0	NA
PPP1R36	1	×	×	0	2.63	*PPP1R3B*	1	×	×	0	NA
PRAMEF1	1	×	×	0	NA	*PRAMEF14*	1	×	×	0	0.23
PRAMEF25	1	×	×	0	NA	*PRAMEF5*	1	×	○	0	NA
PRAP1	1	×	×	0	NA	*PRB1*	1	×	×	0	NA
PRDM7	1	×	×	0	NA	*PRDX3*	1	×	×	0	NA
PRG2	1	×	×	0	1.88	*PRG3*	1	×	×	0	3.8
PRH2	1	×	×	0	-0.35	*PRKDC*	1	×	×	0	NA
PRMT5	1	×	×	0	1.37	*PRR33*	1	×	×	0	-0.59
PRSS27	1	×	×	0	-2.31	*PSD*	1	×	×	0	-1.49
PSG6	1	×	×	0	-1.32	*PSG7*	1	×	×	0	-1.13
PSG8	1	×	×	0	-1.69	*PSPH*	1	×	×	0.00141	NA

Gene	CPC Count (n=116)	HPRC.EAS	HPRC.nEAS	Archaic Proportion (%)	Tajima's D	Gene	CPC Count (n=116)	HPRC.EAS	HPRC.nEAS	Archaic Proportion (%)	Tajima's D
PSRC1	1	×	○	0	NA	*PTK6*	1	×	○	0	NA
PTPN18	1	×	×	0	NA	*PTPN20*	1	×	×	0	NA
PUSL1	1	×	×	0	NA	*QPRT*	1	×	×	0	NA
RAB12	1	×	×	0	NA	*RABGEF1*	1	×	×	0.00449	-0.52
RAD17	1	×	×	0	NA	*RAD51AP1*	1	×	×	0	NA
RASSF7	1	×	×	0.000126	1.8	*RBM23*	1	×	×	0	2.53
RBM28	1	×	×	0	-0.55	*RBM43*	1	×	×	0	NA
RBMY1D	1	×	○	0	NA	*RDH16*	1	×	×	0	-0.12
REEP6	1	×	×	0	NA	*REM2*	1	×	×	0	1.88
REXO4	1	×	×	0.000418	2.49	*RFLNB*	1	×	×	0	0.14
RGCC	1	×	×	0	-0.95	*RGPD4*	1	×	×	0	NA
RHBDF1	1	×	×	0	-0.19	*RHOF*	1	×	×	0	3.48
RIF1	1	×	×	0	2.92	*RILPL2*	1	×	×	0	2.81
RNASE2	1	×	×	0.0000329	-1.03	*RNASE3*	1	×	×	0.0000334	**-2.58**
RNF112	1	×	×	0.000214	NA	*RNF17*	1	×	×	0.00402	0.13
RNF223	1	×	×	0	NA	*RNF227*	1	×	×	0	0.29
ROGDI	1	×	×	0	-1.52	*RP1-37C10.8*	1	×	×	0	NA
RP11-152F13.10	1	×	×	0	NA	*RP11-298I3.5*	1	×	×	0	NA
RP11-343C2.11	1	×	×	0	NA	*RP11-364B14.3*	1	×	×	0	NA
RP11-426L16.10	1	×	×	0	NA	*RP11-473M10.3*	1	×	×	0	NA
RP11-49K24.6	1	×	×	0	NA	*RP11-566K11.2*	1	×	×	0	NA
RP11-724O16.1	1	×	○	0	NA	*RP11-792A8.5*	1	×	×	0	NA
RP11-872D17.8	1	×	×	0	NA	*RPEL1*	1	×	×	0	-1.47
RSC1A1	1	×	×	0.0000805	-1.39	*RTF2*	1	×	×	0	NA
RTP5	1	×	×	0.000147	NA	*RUSF1*	1	×	○	0	NA
SAMD10	1	×	×	0	-2.15	*SAPCD1*	1	×	×	0	-1.46
SARS1	1	×	○	0	NA	*SBDS*	1	×	×	0.000278	-0.01
SBK1	1	×	○	0	NA	*SDF4*	1	×	×	0	NA
SDR42E2	1	×	×	0	NA	*SEC11A*	1	×	○	0	NA
SEC22C	1	×	×	0	0.23	*SERINC4*	1	×	×	0	-0.54
SERPINA12	1	×	×	0	-0.95	*SERPINA4*	1	×	×	0	0.02
SERPINA5	1	×	×	0	0.02	*SETD4*	1	×	×	0	NA
SFXN4	1	×	×	0	NA	*SGCB*	1	×	○	0	NA
SHC2	1	×	×	0.00124	0.25	*SHMT1*	1	×	×	0	-0.76
SKIV2L	1	×	×	0.000371	-1.07	*SLC10A3*	1	×	×	0	NA
SLC25A1	1	×	×	0	NA	*SLC25A45*	1	×	×	0	-0.99
SLC27A5	1	×	×	0	NA	*SLC28A1*	1	×	×	0.00212	-0.45
SLC30A5	1	×	×	0	NA	*SLC35E2B*	1	×	×	0	-0.33
SLC43A3	1	×	×	0	-1.76	*SLC44A4*	1	×	×	0.00000392	-1.54
SLC52A3	1	×	×	0.000484	1.27	*SLC5A2*	1	×	○	0	NA
SLC6A2	1	×	×	0.00143	0.45	*SLC8A2*	1	×	×	0	NA
SMCR8	1	×	×	0	-0.76	*SMIM10L2B*	1	×	×	0	NA
SMIM27	1	×	×	0	-1.74	*SMPD4*	1	×	×	0	**-2.97**
SNURF	1	×	×	0	-1.44	*SOHLH1*	1	×	○	0	NA
SOS2	1	×	×	0	3.06	*SOX7*	1	×	×	0	0.82
SPACA5B	1	×	○	0	NA	*SPATA18*	1	×	○	0	NA
SPATA21	1	○	×	0	NA	*SPATA31A3*	1	×	×	0	NA
SPATA31D3	1	×	×	0	**-3.01**	*SPATA33*	1	×	×	0	NA
SPDYE16	1	×	×	0	NA	*SPIDR*	1	×	×	0	NA
SPN	1	×	×	0	NA	*SPPL2C*	1	×	×	0	NA
SPRR2D	1	×	×	0.0000763	2.39	*SRRM2*	1	×	×	0	-1.28
SS18L2	1	×	×	0	-0.4	*SSRP1*	1	×	×	0.000344	2.37
SSX4B	1	×	○	0	NA	*STAR*	1	×	×	0	-0.72
STING1	1	×	×	0	NA	*STK19*	1	×	×	0.000407	-1.03
STKLD1	1	×	×	0.000971	2.49	*STOML1*	1	×	×	0	3.24
SULT1A2	1	×	○	0	NA	*SUMF2*	1	×	×	0.000579	NA
SURF1	1	×	×	0.000171	-1.62	*SURF2*	1	×	×	0.00016	1.79
SURF4	1	×	×	0.000511	1.79	*SYDE1*	1	×	×	0	0.89
SZRD1	1	×	×	0.000268	NA	*TACSTD2*	1	×	×	0.0000631	-1.09
TAF9	1	×	×	0	NA	*TARBP2*	1	×	×	0	NA
TARM1	1	×	×	0	NA	*TAS2R13*	1	×	×	0	1.02
TBC1D21	1	×	×	0	2.78	*TBC1D3F*	1	×	×	0	NA
TCFL5	1	×	×	0	NA	*TDRD1*	1	×	×	0	1.5
TEDC1	1	×	○	0	NA	*TEX13A*	1	×	×	0	NA
TEX50	1	×	×	0	NA	*THEG*	1	×	×	0	-0.91
THY1	1	×	○	0	NA	*TIGD3*	1	×	×	0	-1.4
TIMM13	1	×	×	0	NA	*TIMM22*	1	×	×	0	NA
TKTL1	1	×	×	0	NA	*TMED9*	1	×	×	0	1.09

Gene	CPC Count (n=116)	HPRC.EAS	HPRC.nEAS	Archaic Proportion (%)	Tajima's D	Gene	CPC Count (n=116)	HPRC.EAS	HPRC.nEAS	Archaic Proportion (%)	Tajima's D
TMEM121	1	×	○	0	NA	*TMEM129*	1	×	×	0	-0.62
TMEM158	1	×	×	0	0.23	*TMEM18*	1	×	×	0.000142	1.07
TMEM191C	1	×	×	0	NA	*TMEM211*	1	×	×	0	NA
TMEM230	1	×	×	0	3.54	*TMEM240*	1	×	×	0	-1.03
TMEM248	1	×	×	0.0013	-0.87	*TMEM252*	1	×	×	0	NA
TNFAIP6	1	×	×	0	**4.31**	*TNFRSF18*	1	×	×	0	NA
TNFRSF4	1	×	×	0	NA	*TNKS1BP1*	1	×	×	0.000879	2.92
TNNI3	1	×	×	0	NA	*TOP3A*	1	×	×	0	-0.59
TOP3B	1	×	×	0	NA	*TP53TG3F*	1	×	×	0	NA
TPGS1	1	×	×	0	NA	*TPI1*	1	×	×	0.000133	-0.68
TRARG1	1	×	×	0	NA	*TRIM25*	1	×	×	0	-1.37
TRIM28	1	×	×	0	NA	*TRIM74*	1	×	×	0	NA
TRIML2	1	×	×	0.000636	1.41	*TRIP13*	1	×	○	0	NA
TRIR	1	×	×	0	-1.58	*TRMT9B*	1	×	×	0	NA
TRPM1	1	×	×	0	3.52	*TSPAN3*	1	×	×	0	NA
TSSK2	1	×	×	0	NA	*TTYH1*	1	×	×	0	NA
TUBA3C	1	×	×	0	-0.37	*TUBA8*	1	×	×	0	NA
TUBAL3	1	×	×	0	NA	*TUBB3*	1	×	×	0	NA
TVP23B	1	×	×	0	NA	*UBE2M*	1	×	×	0	NA
UBL4A	1	×	×	0	NA	*UCN2*	1	×	×	0	-1.67
UCN3	1	×	×	0	NA	*UFD1*	1	×	×	0	NA
UGT2B28	1	×	○	0	NA	*UPF3A*	1	×	×	0.000841	-0.59
USF1	1	×	×	0	NA	*USP17L12*	1	×	×	0	NA
USP17L2	1	×	×	0	NA	*USP17L27*	1	×	×	0	NA
USP17L29	1	×	×	0	NA	*USP2*	1	×	○	0	NA
USP32	1	×	×	0	1.68	*VASH1*	1	×	×	0	**3.51**
VCPKMT	1	×	×	0	2.56	*VGLL3*	1	×	×	0	NA
VPS37D	1	×	×	0	NA	*VPS4A*	1	×	×	0	-1.93
VSTM1	1	×	×	0	0.73	*VWA3A*	1	×	○	0	NA
VWCE	1	×	×	0.00129	-2.16	*WDR1*	1	×	×	0.0017	3.67
WFIKKN1	1	×	×	0.000169	-0.58	*WWP2*	1	×	×	0	3.7
XAGE5	1	×	×	0	NA	*XKR5*	1	×	×	0	NA
XXbac-BPG116M5.17	1	×	×	0	NA	*YPEL1*	1	×	×	0	NA
ZBTB1	1	×	×	0	2.29	*ZBTB12*	1	×	×	0.0000858	-1.64
ZBTB25	1	×	×	0	3.04	*ZBTB45*	1	×	×	0	NA
ZBTB47	1	×	×	0	-1.36	*ZCCHC18*	1	×	×	0	NA
ZFAND2A	1	×	×	0	NA	*ZFP14*	1	×	○	0	NA
ZFP82	1	×	○	0	NA	*ZIM3*	1	×	×	0	1.45
ZNF101	1	×	×	0	0.47	*ZNF107*	1	×	×	0	-1.44
ZNF146	1	×	×	0	NA	*ZNF200*	1	×	○	0	NA
ZNF217	1	×	×	0.00107	-1.73	*ZNF281*	1	×	×	0	3.68
ZNF324	1	×	×	0	NA	*ZNF446*	1	×	×	0	NA
ZNF449	1	×	○	0	NA	*ZNF496*	1	×	×	0	NA
ZNF511	1	×	×	0	NA	*ZNF541*	1	×	×	0	NA
ZNF558	1	×	×	0	NA	*ZNF565*	1	×	×	0	NA
ZNF592	1	×	×	0.00201	-1.75	*ZNF679*	1	×	×	0.00133	-0.08
ZNF680	1	×	×	0	NA	*ZNF705D*	1	×	○	0	NA
ZNF716	1	×	○	0	NA	*ZNF735*	1	×	×	0.000454	NA
ZNF736	1	×	×	0	0.87	*ZNF750*	1	×	○	0	NA
ZNF75D	1	×	○	0	NA	*ZNF761*	1	×	×	0	NA
ZNF799	1	×	×	0	-2.2	*ZNF813*	1	×	×	0	NA
ZNF839	1	×	×	0.000879	3.24	*ZP2*	1	×	×	0	NA

Note: The presence/absence of the CNV-related gene in HPRC is indicated with "○"/"×"; Archaic proportion is calculated as the length of archaic introgressed segments (AIS) in each gene devided by the total AIS in the genome. The Tajima's *D* estimates with FDR-adjusted $P < 0.05$ are highlighted in red fonts.

Supplementary Table 5 Functional enrichment of the novel duplicated genes in the CPC assembly set.

Database	Term ID	Description	GeneRatio	BgRatio	Odds ratio	BH-adjusted *p*-value	*q*-value
GO:BP	GO:0007608	Sensory perception of smell	98/837	453/18493	6.46	2.22×10^{-36}	2.22×10^{-36}
GO:BP	GO:0050911	Detection of chemical stimulus involved in sensory perception of smell	95/837	427/18493	6.68	2.22×10^{-36}	2.22×10^{-36}
GO:BP	GO:0050907	Detection of chemical stimulus involved in sensory perception	97/837	477/18493	5.96	7.08×10^{-34}	7.08×10^{-34}
GO:MF	GO:0004984	Olfactory receptor activity	95/839	427/17632	6.33	4.61×10^{-35}	4.61×10^{-35}
GO:MF	GO:0005549	Odorant binding	29/839	96/17632	8.94	1.53×10^{-13}	1.53×10^{-13}
GO:MF	GO:0004843	Thiol-dependent ubiquitin-specific protease activity	21/839	105/17632	5.11	4.77×10^{-6}	4.77×10^{-6}
GO:MF	GO:0004197	Cysteine-type endopeptidase activity	22/839	118/17632	4.68	6.32×10^{-6}	6.32×10^{-6}
GO:MF	GO:0036459	Thiol-dependent ubiquitinyl hydrolase activity	21/839	123/17632	4.20	4.10×10^{-5}	4.10×10^{-5}
GO:MF	GO:0101005	Ubiquitinyl hydrolase activity	21/839	123/17632	4.20	4.10×10^{-5}	4.10×10^{-5}
GO:MF	GO:0019783	Ubiquitin-like protein-specific protease activity	21/839	133/17632	3.82	1.34×10^{-4}	1.34×10^{-4}
GO:MF	GO:0008234	Cysteine-type peptidase activity	25/839	195/17632	3.00	5.98×10^{-4}	5.98×10^{-4}
GO:MF	GO:0051787	Misfolded protein binding	6/839	23/17632	7.11	0.048	0.048
KEGG	hsa04740	Olfactory transduction	97/392	439/8163	7.14	8.35×10^{-38}	8.35×10^{-38}

Note: GeneRatio denotes the ratio of input genes that are annotated in a term, and BgRatio denotes the ratio of all genes that are annotated in a term. The *P*-values are obtained by one-sided Fisher's exact test. They are further adjusted for multiple comparisons using the Benjamini-Hochberg procedure (denoted as the BH-adjusted *P*-value), and are also adjusted for the false discovery rate (denoted as the q-value).

Supplementary Table 6 Overlapped CNV-related-genes between CPC and HPRC

Gene	CPC Frequency (n=116)	HPRC Frequency (n=88)	HPRC.EAS Frequency (n=8)	HPRC.nEAS Frequency (n=80)	Gene	CPC Frequency (n=116)	HPRC Frequency (n=88)	HPRC.EAS Frequency (n=8)	HPRC.nEAS Frequency (n=80)
CYP2D6	0.26	0.06	0.00	0.05	*TSEN34*	0.03	0.02	0.00	0.03
CFC1	0.18	0.06	0.00	0.06	*RPIA*	0.03	0.02	0.00	0.03
PNMA6A	0.15	0.03	0.00	0.04	*CATSPER2*	0.03	0.02	0.00	0.03
GOLGA6L4	0.09	0.02	0.00	0.03	*OR4Q3*	0.25	0.25	0.13	0.25
TSPY8	0.16	0.10	0.13	0.10	*NBPF19*	0.26	0.26	0.00	0.29
LIMS4	0.09	0.05	0.00	0.05	*MTLN*	0.01	0.01	0.00	0.01
CTAG1A	0.06	0.01	0.00	0.01	*COL6A2*	0.01	0.01	0.00	0.01
POTEB2	0.14	0.09	0.13	0.06	*SBK1*	0.01	0.01	0.00	0.01
ARHGEF35	0.07	0.02	0.13	0.01	*RUSF1*	0.01	0.01	0.00	0.01
GOLGA8N	0.08	0.03	0.00	0.04	*ZFP82*	0.01	0.01	0.00	0.01
GOLGA6L9	0.04	0.00	0.00	0.00	*MCCC2*	0.01	0.01	0.00	0.01
ZNF658	0.05	0.01	0.00	0.01	*NOB1*	0.01	0.01	0.00	0.01
NOTCH2NLB	0.10	0.07	0.13	0.06	*DEFB105A*	0.01	0.01	0.00	0.01
CD99	0.03	0.00	0.00	0.00	*LCN15*	0.01	0.01	0.00	0.01
CRLF2	0.03	0.00	0.00	0.00	*MPV17L*	0.01	0.01	0.00	0.01
FAHD2B	0.04	0.01	0.00	0.01	*EPHB3*	0.01	0.01	0.00	0.01
PRAMEF4	0.04	0.01	0.00	0.01	*ZNF200*	0.01	0.01	0.00	0.01
PRAMEF2	0.04	0.01	0.00	0.01	*UGT2B28*	0.01	0.01	0.00	0.01
PRAMEF10	0.06	0.03	0.00	0.04	*FASTKD1*	0.01	0.01	0.00	0.01
VAMP7	0.03	0.00	0.00	0.00	*AGPAT2*	0.01	0.01	0.13	0.00
GTPBP6	0.03	0.00	0.00	0.00	*ZNF750*	0.01	0.01	0.00	0.01
CHRFAM7A	0.03	0.01	0.00	0.01	*BDP1*	0.01	0.01	0.00	0.01
SSX2	0.03	0.01	0.00	0.01	*SOHLH1*	0.01	0.01	0.00	0.01
BOLA1	0.03	0.01	0.13	0.00	*PDX1*	0.01	0.01	0.00	0.01
H3-2	0.04	0.02	0.00	0.03	*ISLR2*	0.01	0.01	0.00	0.01
SYT15	0.04	0.02	0.00	0.03	*H2BC18*	0.01	0.01	0.00	0.01
PRAMEF8	0.05	0.03	0.00	0.04	*GSX1*	0.01	0.01	0.00	0.01
ZBED1	0.02	0.00	0.00	0.00	*ASXL1*	0.01	0.01	0.00	0.01
IL9R	0.02	0.00	0.00	0.00	*VWA3A*	0.01	0.01	0.00	0.01
SHOX	0.02	0.00	0.00	0.00	*PSRC1*	0.01	0.01	0.00	0.01
NTAN1	0.06	0.05	0.00	0.05	*RP11-724O16.1*	0.01	0.01	0.00	0.01
RRN3	0.06	0.05	0.00	0.05	*NOTCH2NLR*	0.01	0.01	0.00	0.01
PRAMEF27	0.03	0.01	0.00	0.01	*LSS*	0.01	0.01	0.00	0.01
SULT2A1	0.03	0.01	0.00	0.01	*FCGR1A*	0.01	0.01	0.00	0.01
DEFA5	0.03	0.01	0.00	0.01	*SGCB*	0.01	0.01	0.00	0.01
FAM178B	0.03	0.01	0.00	0.01	*AQP12B*	0.01	0.01	0.00	0.01
ANKRD20A4P	0.03	0.01	0.00	0.01	*GABRD*	0.01	0.01	0.00	0.01
USP51	0.03	0.01	0.00	0.01	*USP2*	0.01	0.01	0.00	0.01
LIMS3	0.03	0.01	0.00	0.01	*BBS5*	0.01	0.01	0.00	0.01
IZUMO2	0.03	0.01	0.00	0.01	*KLHL41*	0.01	0.01	0.00	0.01
ERV3-1	0.03	0.01	0.00	0.01	*PTK6*	0.01	0.01	0.00	0.01
ANKRD36B	0.03	0.01	0.13	0.00	*SULT1A2*	0.01	0.01	0.00	0.01
FAHD2A	0.03	0.01	0.00	0.01	*SARS1*	0.01	0.01	0.00	0.01
SEMA4C	0.03	0.01	0.00	0.01	*CELSR2*	0.01	0.01	0.00	0.01
SLC16A8	0.03	0.01	0.00	0.01	*EPN1*	0.01	0.01	0.00	0.00
SLC5A10	0.03	0.01	0.00	0.01	*SLC5A2*	0.01	0.01	0.00	0.01
RRP7A	0.11	0.10	0.13	0.10	*ATP6V0E2*	0.01	0.01	0.13	0.00
RGPD5	0.04	0.03	0.13	0.03	*ZNF705D*	0.01	0.01	0.00	0.01
AKAP17A	0.01	0.00	0.00	0.00	*PRAMEF5*	0.01	0.01	0.00	0.01
PLCXD1	0.01	0.00	0.00	0.00	*NPHP1*	0.01	0.01	0.00	0.01
CD2BP2	0.01	0.00	0.00	0.00	*SPATA21*	0.01	0.01	0.13	0.00
CRX	0.02	0.01	0.00	0.01	*EGFL7*	0.01	0.01	0.13	0.00
NECAP2	0.02	0.01	0.13	0.00	*OPN1MW3*	0.01	0.01	0.00	0.01
LILRB5	0.02	0.01	0.00	0.01	*ZFP14*	0.01	0.01	0.00	0.01
PCDHB16	0.02	0.01	0.00	0.01	*TRIP13*	0.01	0.01	0.00	0.01
FAM72C	0.02	0.01	0.00	0.01	*BHMT*	0.01	0.01	0.13	0.00
CNNM4	0.02	0.01	0.00	0.01	*ABT1*	0.01	0.01	0.00	0.01
CBWD6	0.02	0.01	0.13	0.00	*ZNF716*	0.01	0.01	0.00	0.01
HNRNPCL1	0.02	0.01	0.00	0.01	*THY1*	0.01	0.01	0.00	0.01
WDR73	0.02	0.01	0.00	0.01	*SPATA18*	0.01	0.01	0.00	0.01
EIF3CL	0.02	0.01	0.00	0.01	*SEC11A*	0.01	0.01	0.00	0.01
FAM72B	0.02	0.01	0.00	0.01	*DCUN1D4*	0.01	0.01	0.00	0.01
PDXDC1	0.02	0.01	0.00	0.01	*PARN*	0.01	0.01	0.00	0.01
CDK11B	0.02	0.01	0.00	0.01	*ALG10*	0.01	0.01	0.00	0.01
TPRX1	0.02	0.01	0.00	0.01	*EIF2AK3*	0.02	0.02	0.00	0.03
TRIML1	0.02	0.01	0.00	0.01	*NOTCH2NLC*	0.02	0.02	0.00	0.03
NMB	0.02	0.01	0.00	0.01	*CLN3*	0.02	0.02	0.00	0.03
IL27	0.02	0.01	0.00	0.01	*APOBR*	0.02	0.02	0.00	0.03
BSPH1	0.02	0.01	0.00	0.01	*RP11-315A19.1*	0.02	0.02	0.00	0.03
RP11-435I10.4	0.02	0.01	0.00	0.01	*FOXI3*	0.02	0.02	0.00	0.03
HSFX2	0.02	0.01	0.00	0.00	*IGSF6*	0.02	0.02	0.00	0.03
NDUFAF8	0.02	0.01	0.00	0.01	*HIC2*	0.03	0.03	0.00	0.04
ZSCAN2	0.02	0.01	0.00	0.01	*OR4N2*	0.10	0.11	0.00	0.11
LMAN2L	0.02	0.01	0.00	0.01	*FAM156B*	0.03	0.05	0.13	0.04
SELENOW	0.02	0.01	0.00	0.01	*SSX4B*	0.01	0.02	0.00	0.03
ANKRD36	0.02	0.01	0.00	0.01	*CES1*	0.01	0.02	0.00	0.03
CNNM3	0.02	0.01	0.00	0.01	*CYP2A6*	0.01	0.02	0.00	0.03
NUPR1	0.02	0.01	0.00	0.01	*AL928654.7*	0.01	0.02	0.00	0.01
OR4M1	0.23	0.23	0.00	0.24	*SPACA5B*	0.01	0.02	0.00	0.03

Gene	CPC Frequency (n=116)	HPRC Frequency (n=88)	HPRC.EAS Frequency (n=8)	HPRC.nEAS Frequency (n=80)	Gene	CPC Frequency (n=116)	HPRC Frequency (n=88)	HPRC.EAS Frequency (n=8)	HPRC.nEAS Frequency (n=80)
ZNF449	0.01	0.02	0.00	0.03	SPDYE2	0.28	0.74	0.38	0.75
PLA2G10	0.01	0.02	0.00	0.03	ARL17A	0.16	0.65	0.50	0.64
ATF7IP2	0.01	0.02	0.13	0.01	NBPF1	0.47	0.98	0.75	0.98
CH17-159N18.5	0.01	0.02	0.00	0.03	DEFB107A	0.46	0.98	0.75	0.98
MYEOV	0.01	0.02	0.00	0.03	NOMO1	0.44	0.98	0.75	0.98
COL6A1	0.01	0.02	0.00	0.03	OR2A1	0.43	0.98	0.75	0.98
LAIR1	0.01	0.02	0.00	0.03	GAGE2E	0.18	0.74	0.50	0.74
CRIP1	0.01	0.02	0.00	0.01	SLX1A	0.37	0.98	0.75	0.98
OTOA	0.01	0.02	0.00	0.03	OR4F17	0.35	0.98	0.75	0.98
ZNF75D	0.01	0.02	0.00	0.03	RASA4	0.33	0.98	0.75	0.98
TEDC1	0.01	0.02	0.00	0.01	UPK3BL1	0.32	0.98	0.75	0.98
XAGE3	0.01	0.02	0.13	0.01	GPAT2	0.04	0.77	0.63	0.78
TMEM191B	0.05	0.07	0.00	0.08	DEFB4A	0.23	0.98	0.75	0.98
ABC7-42404400C24.1	0.05	0.07	0.00	0.06	PRR23D1	0.23	0.98	0.75	0.98
CYP21A2	0.02	0.03	0.00	0.04	DEFB104A	0.22	0.98	0.75	0.98
CT55	0.02	0.03	0.00	0.04	DEFB106A	0.22	0.98	0.75	0.98
CORO1A	0.02	0.03	0.25	0.01	PRR20A	0.22	0.98	0.75	0.98
EMB	0.02	0.03	0.00	0.04	GOLGA6L10	0.17	0.98	0.75	0.98
TPSB2	0.02	0.03	0.00	0.04	DEFA1	0.17	0.98	0.75	0.98
TEX37	0.02	0.03	0.00	0.04	C4A	0.16	0.98	0.75	0.98
BFAR	0.02	0.03	0.00	0.04	BOLA2	0.16	0.98	0.75	0.98
DDTL	0.03	0.05	0.00	0.05	MBD3L2	0.14	0.98	0.75	0.98
OR9G1	0.49	0.51	0.38	0.53	RFPL4A	0.13	0.98	0.75	0.98
METTL9	0.01	0.03	0.00	0.04	AMY1A	0.12	0.98	0.75	0.98
TMEM121	0.01	0.03	0.00	0.03	KIR2DL1	0.12	0.98	0.75	0.98
CRIP2	0.01	0.03	0.00	0.01	NPY4R	0.11	0.98	0.75	0.98
RCC1L	0.01	0.03	0.13	0.03	ORM1	0.10	0.98	0.75	0.98
STRC	0.02	0.05	0.00	0.05	GOLGA6A	0.10	0.98	0.75	0.98
RBMY1B	0.02	0.05	0.13	0.04	CLEC18A	0.09	0.98	0.75	0.98
TSPY1	0.04	0.08	0.00	0.09	GOLGA6L1	0.09	0.98	0.75	0.98
NSUN5	0.01	0.05	0.00	0.05	LRRC37A	0.08	0.98	0.75	0.98
RBMY1A1	0.02	0.06	0.13	0.05	POTEB	0.08	0.98	0.75	0.98
RP11-353J17.5	0.02	0.06	0.00	0.06	SPAG11A	0.06	0.98	0.75	0.98
ANTXRL	0.02	0.06	0.00	0.05	SIGLEC14	0.04	0.98	0.75	0.98
TBC1D3I	0.02	0.07	0.00	0.08	CXorf49	0.04	0.98	0.75	0.98
NPIPB12	0.11	0.17	0.00	0.19	GRAP	0.03	0.98	0.75	0.98
GGTLC3	0.01	0.07	0.00	0.08	ETDA	0.03	0.98	0.75	0.98
RBMY1D	0.01	0.07	0.00	0.08	TRIM43	0.03	0.98	0.75	0.98
WASH6P	0.02	0.08	0.13	0.06	TBC1D3	0.03	0.98	0.75	0.98
DMRTC1B	0.02	0.08	0.00	0.09	GYPA	0.03	0.98	0.75	0.98
GSTT4	0.10	0.17	0.00	0.19	CT45A1	0.03	0.98	0.75	0.98
OR4K1	0.32	0.39	0.25	0.39	PICK1	0.03	0.98	0.75	0.98
OR4K2	0.31	0.39	0.25	0.39	CKMT1A	0.03	0.98	0.75	0.98
PDPR	0.39	0.47	0.25	0.48	FAM90A1	0.03	0.98	0.75	0.98
SPDYE2B	0.16	0.25	0.25	0.25	LILRA6	0.03	0.98	0.75	0.98
RBMY1E	0.03	0.13	0.00	0.14	H4C14	0.03	0.98	0.75	0.98
SPDYE13	0.04	0.14	0.00	0.15	LILRA4	0.03	0.98	0.75	0.98
TSPY10	0.05	0.15	0.25	0.14	TRIM51	0.03	0.98	0.75	0.98
EIF3C	0.10	0.20	0.00	0.23	FRG2	0.03	0.98	0.75	0.98
GPRIN2	0.82	0.93	0.75	0.93	POLR2J	0.03	0.98	0.75	0.98
TSPY9P	0.08	0.19	0.25	0.19	SMN1	0.02	0.98	0.75	0.98
FRG1	0.02	0.15	0.13	0.15	PRAMEF11	0.02	0.98	0.75	0.98
MRGPRX1	0.12	0.26	0.13	0.28	FCGR2A	0.02	0.98	0.75	0.98
DEFB105B	0.20	0.34	0.38	0.34	TRIM48	0.02	0.98	0.75	0.98
ANKRD20A3P	0.01	0.16	0.00	0.15	ANKRD23	0.02	0.98	0.75	0.98
TPTE	0.42	0.58	0.38	0.60	TRIM64	0.02	0.98	0.75	0.98
TSPY4	0.03	0.19	0.25	0.19	CGB1	0.01	0.98	0.75	0.98
CCL3L1	0.28	0.47	0.63	0.44	SMIM10L2A	0.01	0.98	0.75	0.98
PRAMEF18	0.40	0.58	0.13	0.60	CCL4	0.01	0.98	0.75	0.98
TSPY3	0.01	0.19	0.25	0.19	FAM86B1	0.01	0.98	0.75	0.98
CLPS	0.34	0.55	0.63	0.53	GGT1	0.01	0.98	0.75	0.98
RP11-577H5.5	0.27	0.48	0.25	0.48	ZDHHC11	0.01	0.98	0.75	0.98
SULT1A4	0.13	0.34	0.38	0.34	NPIPA1	0.01	0.98	0.75	0.98
RP11-514P8.7	0.24	0.49	0.38	0.49	SPATA31D1	0.01	0.98	0.75	0.98
SULT1A3	0.37	0.64	0.25	0.66	PLG	0.01	0.98	0.75	0.98
RP11-514P8.6	0.30	0.57	0.38	0.58	OPN1LW	0.01	0.98	0.75	0.98
DUSP22	0.56	0.83	0.63	0.83	NUTM2A	0.01	0.98	0.75	0.98
BOLA2B	0.42	0.70	0.38	0.73	XAGE1A	0.01	0.98	0.75	0.98
LINC02203	0.02	0.41	0.63	0.36	PSG1	0.01	0.98	0.75	0.98
NBPF9	0.02	0.44	0.25	0.45	ANXA8	0.01	0.98	0.75	0.98

Supplementary Table 7 A comparison of variant calling based on linear genome reference and graph genome reference

Sample ID	Population	Gender	NGS				TGS	
			#SNV	#Indel	#Small	#SV	#Small	#SV
HIFI032682D	Achang	M	3,981,870	934,745	4,916,615	6,522	4,599,222	26,541
HIFI032585D	Bai	M	3,946,080	903,023	4,849,103	6,475	4,578,607	26,513
HIFI032069D	Blang	F	3,967,098	914,684	4,881,782	6,653	4,606,504	26,705
HIFI032373D	Blang	M	3,945,193	922,679	4,867,872	6,485	4,571,708	26,631
HIFI032487D	Bouyei	F	3,951,056	909,839	4,860,895	6,434	4,557,349	26,489
HIFI032018D	Chosen	M	3,958,448	922,895	4,881,343	6,958	4,585,641	26,538
HIFI032306D	Chosen	F	3,956,333	919,731	4,876,064	6,442	4,599,628	26,934
RY05-CNCSM0001	Chosen	M	3,986,610	981,715	4,968,325	6,395	4,568,397	26,611
HIFI032668D	Daur	M	3,941,864	919,041	4,860,905	6,766	4,562,386	26,384
HIFI032698D	Daur	F	4,004,782	922,601	4,927,383	6,426	4,631,431	27,105
HIFI032292D	Deang	F	3,971,930	913,793	4,885,723	6,616	4,644,571	26,661
HIFI032462D	Deang	M	3,956,954	909,444	4,866,398	6,392	4,602,115	26,413
HIFI032473D	Dong	M	3,930,632	913,711	4,844,343	6,590	4,584,493	26,871
HIFI032510D	Dong	F	3,953,361	923,789	4,877,150	6,733	4,602,817	26,869
HIFI032450D	Drung	M	3,869,859	897,694	4,767,553	6,420	4,487,380	26,093
HIFI032706D	Evenki	M	3,956,037	926,899	4,882,936	6,602	4,601,577	27,049
HIFI032007D	Kinh	F	3,964,429	901,121	4,865,550	6,264	4,627,015	27,046
RY06-CNKGF0001	Kyrgyz	F	3,978,361	977,645	4,956,006	6,341	4,574,266	26,905
RY07-CNKZF0001	Kazakh	F	4,086,612	995,131	5,081,743	6,482	4,696,823	27,628
RY08-CNZHF0001	Zhuang	F	4,013,892	991,844	5,005,736	6,355	4,588,727	26,786
RY09-CNHUM0001	Hui	M	4,032,994	989,653	5,022,647	6,486	4,615,882	27,000
HIFI032513D	Kinh	M	3,945,516	919,825	4,865,341	6,344	4,582,526	26,533
HIFI032685D	Hezhen	M	3,932,237	924,893	4,857,130	8,154	4,584,970	26,739
HIFI032440D	Jingpo	M	3,964,992	919,729	4,884,721	6,860	4,589,200	26,329
HIFI032429D	Jino	M	3,894,987	883,763	4,778,750	6,317	4,529,639	26,174
HIFI032731D	Lisu	F	3,938,758	911,987	4,850,745	5,919	4,566,520	26,344
HIFI032289D	Man	M	3,933,484	913,460	4,846,944	6,779	4,630,959	26,889
HIFI032167D	Miao	M	3,943,574	914,567	4,858,141	6,428	4,578,903	26,449
RY04-CNMHM0001	Miao	M	4,001,321	978,182	4,979,503	6,475	4,554,917	26,635
HIFI032164D	Mongol	M	3,971,878	919,973	4,891,851	6,491	4,604,914	26,844
RY10-CNMGF0001	Mongol	F	4,046,681	987,976	5,034,657	6,431	4,647,535	26,988
HIFI032607D	Mongol	F	3,989,424	920,192	4,909,616	6,291	4,653,140	27,110
HIFI032604D	Khatso	F	3,991,529	921,907	4,913,436	6,081	4,647,405	26,859
HIFI032161D	Mosuo	F	3,986,438	921,085	4,907,523	6,550	4,613,822	27,035
HIFI032422D	Mosuo	M	3,962,229	913,173	4,875,402	6,571	4,587,426	26,332
HIFI032501D	Mosuo	M	3,941,005	908,076	4,849,081	6,609	4,570,766	26,674
HIFI032693D	Naxi	M	3,973,593	923,681	4,897,274	6,793	4,570,682	27,079
HIFI032335D	Oroqen	U*	3,975,892	933,232	4,909,124	6,800	4,584,312	26,710
HIFI032420D	Qiang	M	3,962,567	923,202	4,885,769	6,498	4,594,111	26,676
HIFI032567D	Qiang	F	3,963,390	918,122	4,881,512	6,776	4,613,808	26,786
HIFI032662D	Qiang	M	3,939,887	922,339	4,862,226	6,969	4,575,002	26,367
HIFI032453D	Salar	M	3,985,636	924,962	4,910,598	6,923	4,650,093	26,809
HIFI032586D	Salar	F	4,009,785	940,648	4,950,433	6,389	4,647,321	27,275
HIFI032302D	She	F	3,994,318	934,364	4,928,682	6,999	4,599,200	26,811
HIFI032591D	She	M	3,951,041	908,420	4,859,461	6,225	4,584,528	26,403
RY03-CNSHM0001	She	M	3,993,538	982,478	4,976,016	6,270	4,569,518	26,661
HIFI032349D	Tu	M	3,927,367	916,009	4,843,376	8,645	4,615,686	26,942
HIFI032454D	Tu	F	3,990,219	920,184	4,910,403	6,773	4,657,164	27,089
HIFI032097D	Tujia	F	3,975,408	920,010	4,895,418	6,805	4,631,580	26,835
HIFI032103D	Tujia	M	3,961,654	917,803	4,879,457	6,747	4,571,844	26,623
RY11-CNTJM0001	Tujia	M	4,004,957	938,380	4,943,337	7,082	4,574,980	26,537
HIFI032529D	Wa	F	3,967,728	929,913	4,897,641	6,604	4,602,395	26,593
HIFI032711D	Wa	M	3,926,544	908,897	4,835,441	6,613	4,548,274	26,349
HIFI032182D	Yi	M	3,951,309	920,145	4,871,454	6,843	4,579,802	26,744
HIFI032566D	Yugur	M	3,987,602	918,758	4,906,360	6,324	4,629,655	26,820
HIFI032692D	Yugur	F	4,033,224	945,721	4,978,945	6,826	4,667,689	27,293
RY01-CNYAM0001	Yao	M	3,968,734	974,209	4,942,943	6,182	4,587,778	26,404
RY02-CNUGM0001	Uyghur	M	4,083,527	998,171	5,081,698	6,284	4,704,908	27,152

Supplementary Table 8 Compare the size of HPRC graph reference and CPC graph reference. These include raw graph and simplified graph for short-reads mapping.

Filter	Genome	No.nodes	No.edges	Complexity	Length
raw	HPRC	93,912,262	129,516,414	1.379	3,350,442,208
	CPC	64,474,746	89,583,031	1.389	3,284,609,818
maf>=0.1	HPRC	67,422,754	80,783,425	1.198	3,166,207,441
	CPC	47,247,698	57,905,003	1.225	3,154,872,380

The complexity of a graph is calculated by the ratio of edges to nodes (edges/nodes).

Supplementary Table 9 The number of small variants whether can be found in the gnomAD v0.1.8 in different regions defined in GIAB 3.0

	CPC_specific	HPRC_specific	common
GIAB_difficult | not_gnomad	470,575	695,935	3,652,516
GIAB_difficult | gnomad	747,025	2,445,856	3,963,791
GIAB_easy | not_gnomad	934,874	622,404	171,494
GIAB_easy | gnomad	2,367,509	8,213,124	6,247,382

Note: To make it comparable to GRCh38-based gnomAD and GIAB, the number of small variants was calculated from the joint MC pangenome vcf file which used CHM13 as the reference (the first assembly) and the GRCh38 (the second assembly) as the coordinate with parameters "--reference CHM13v2 --vcfReference GRCh38" in the "cactus-graphmap-join" step.

Supplementary Table 10 Functional enrichment of genes affected by the novel SVs (≥ 1kb) in the CPC assembly set.

Database	Term ID	Description	GeneRatio	BgRatio	Odds ratio	BH-adjusted *p*-value	*q*-value
GO:BP	GO:0002455	humoral immune response mediated by circulating immunoglobulin	54/1891	149/18493	5.11	8.50×10^{-14}	8.50×10^{-14}
GO:BP	GO:0006958	complement activation, classical pathway	51/1891	137/18493	5.32	9.03×10^{-14}	9.03×10^{-14}
GO:BP	GO:0006956	complement activation	57/1891	170/18493	4.54	2.21×10^{-13}	2.21×10^{-13}
GO:BP	GO:0072376	protein activation cascade	61/1891	194/18493	4.13	4.55×10^{-13}	4.55×10^{-13}
GO:BP	GO:0006959	humoral immune response	85/1891	349/18493	2.91	1.64×10^{-11}	1.64×10^{-11}
GO:BP	GO:0006910	phagocytosis, recognition	35/1891	84/18493	6.37	5.29×10^{-11}	5.29×10^{-11}
GO:BP	GO:0002429	immune response-activating cell surface receptor signaling pathway	93/1891	414/18493	2.62	1.25×10^{-10}	1.25×10^{-10}
GO:BP	GO:0002768	immune response-regulating cell surface receptor signaling pathway	97/1891	445/18493	2.53	2.25×10^{-10}	2.25×10^{-10}
GO:BP	GO:0006909	phagocytosis	80/1891	342/18493	2.76	5.40×10^{-10}	5.40×10^{-10}
GO:BP	GO:0043547	positive regulation of GTPase activity	88/1891	405/18493	2.51	2.77×10^{-9}	2.77×10^{-9}
GO:BP	GO:0099024	plasma membrane invagination	40/1891	121/18493	4.41	3.54×10^{-9}	3.54×10^{-9}
GO:BP	GO:0006911	phagocytosis, engulfment	38/1891	112/18493	4.58	4.43×10^{-9}	4.43×10^{-9}
GO:BP	GO:0010324	membrane invagination	41/1891	129/18493	4.16	6.83×10^{-9}	6.83×10^{-9}
GO:BP	GO:0043087	regulation of GTPase activity	96/1891	481/18493	2.25	3.44×10^{-8}	3.44×10^{-8}
GO:BP	GO:0016064	immunoglobulin mediated immune response	54/1891	212/18493	3.06	5.97×10^{-8}	5.97×10^{-8}
GO:BP	GO:0019724	B cell mediated immunity	54/1891	213/18493	3.04	6.48×10^{-8}	6.48×10^{-8}
GO:BP	GO:0030449	regulation of complement activation	35/1891	107/18493	4.33	6.48×10^{-8}	6.48×10^{-8}
GO:BP	GO:2000257	regulation of protein activation cascade	35/1891	108/18493	4.27	8.18×10^{-8}	8.18×10^{-8}
GO:BP	GO:0002433	immune response-regulating cell surface receptor signaling pathway involved in phagocytosis	39/1891	135/18493	3.62	3.24×10^{-7}	3.24×10^{-7}
GO:BP	GO:0038096	Fc-gamma receptor signaling pathway involved in phagocytosis	39/1891	135/18493	3.62	3.24×10^{-7}	3.24×10^{-7}
GO:BP	GO:0002920	regulation of humoral immune response	37/1891	126/18493	3.70	4.96×10^{-7}	4.96×10^{-7}
GO:BP	GO:0038094	Fc-gamma receptor signaling pathway	39/1891	138/18493	3.51	5.91×10^{-7}	5.91×10^{-7}
GO:BP	GO:0002431	Fc receptor mediated stimulatory signaling pathway	39/1891	140/18493	3.44	8.88×10^{-7}	8.88×10^{-7}
GO:BP	GO:0038093	Fc receptor signaling pathway	47/1891	187/18493	3.00	9.02×10^{-7}	9.02×10^{-7}
GO:BP	GO:0008037	cell recognition	50/1891	213/18493	2.74	3.24×10^{-6}	3.24×10^{-6}
GO:BP	GO:0050871	positive regulation of B cell activation	38/1891	144/18493	3.19	6.22×10^{-6}	6.22×10^{-6}
GO:BP	GO:0050853	B cell receptor signaling pathway	34/1891	122/18493	3.44	7.38×10^{-6}	7.38×10^{-6}
GO:BP	GO:0038095	Fc-epsilon receptor signaling pathway	33/1891	117/18493	3.49	8.19×10^{-6}	8.19×10^{-6}
GO:BP	GO:0042742	defense response to bacterium	65/1891	319/18493	2.29	8.20×10^{-6}	8.20×10^{-6}
GO:BP	GO:0002377	immunoglobulin production	43/1891	180/18493	2.80	1.60×10^{-5}	1.60×10^{-5}
GO:BP	GO:0070613	regulation of protein processing	41/1891	170/18493	2.83	2.32×10^{-5}	2.32×10^{-5}
GO:BP	GO:1903317	regulation of protein maturation	41/1891	172/18493	2.79	3.16×10^{-5}	3.16×10^{-5}
GO:BP	GO:0002460	adaptive immune response based on somatic recombination of immune receptors built from immunoglobulin superfamily domains	68/1891	354/18493	2.13	3.79×10^{-5}	3.79×10^{-5}
GO:BP	GO:0002673	regulation of acute inflammatory response	37/1891	149/18493	2.94	3.90×10^{-5}	3.90×10^{-5}
GO:BP	GO:0050851	antigen receptor-mediated signaling pathway	54/1891	259/18493	2.35	4.31×10^{-5}	4.31×10^{-5}
GO:BP	GO:0002526	acute inflammatory response	46/1891	211/18493	2.48	8.41×10^{-5}	8.41×10^{-5}
GO:BP	GO:0002449	lymphocyte mediated immunity	65/1891	344/18493	2.08	1.14×10^{-4}	1.14×10^{-4}
GO:BP	GO:0090630	activation of GTPase activity	26/1891	91/18493	3.55	1.23×10^{-4}	1.23×10^{-4}
GO:BP	GO:0050864	regulation of B cell activation	39/1891	183/18493	2.41	0.001	0.001
GO:BP	GO:0006898	receptor-mediated endocytosis	61/1891	346/18493	1.91	0.002	0.002
GO:BP	GO:0002440	production of molecular mediator of immune response	50/1891	268/18493	2.04	0.003	0.003
GO:BP	GO:0016485	protein processing	55/1891	310/18493	1.92	0.005	0.005
GO:BP	GO:0051251	positive regulation of lymphocyte activation	57/1891	333/18493	1.84	0.009	0.009
GO:BP	GO:0002696	positive regulation of leukocyte activation	62/1891	372/18493	1.78	0.010	0.010
GO:BP	GO:0016266	O-glycan processing	17/1891	61/18493	3.41	0.011	0.011
GO:BP	GO:0050900	leukocyte migration	75/1891	478/18493	1.66	0.014	0.014
GO:BP	GO:0050867	positive regulation of cell activation	62/1891	384/18493	1.71	0.023	0.023
GO:BP	GO:0031589	cell-substrate adhesion	56/1891	338/18493	1.77	0.023	0.023
GO:BP	GO:0034330	cell junction organization	49/1891	287/18493	1.83	0.027	0.027
GO:BP	GO:0015671	oxygen transport	7/1891	15/18493	7.71	0.040	0.040
GO:BP	GO:0030198	extracellular matrix organization	56/1891	348/18493	1.70	0.046	0.046
GO:CC	GO:0042571	immunoglobulin complex, circulating	33/2028	72/19659	7.46	9.09×10^{-12}	9.04×10^{-12}
GO:CC	GO:0019814	immunoglobulin complex	33/2028	76/19659	6.77	3.12×10^{-11}	3.10×10^{-11}
GO:CC	GO:0009897	external side of plasma membrane	67/2028	359/19659	2.03	2.55×10^{-4}	2.53×10^{-4}
GO:CC	GO:0005581	collagen trimer	21/2028	87/19659	2.78	0.027	0.027
GO:CC	GO:0072562	blood microparticle	30/2028	147/19659	2.25	0.027	0.027
GO:CC	GO:0005796	Golgi lumen	23/2028	103/19659	2.52	0.031	0.031
GO:MF	GO:0003823	antigen binding	78/1808	199/17632	5.85	2.85×10^{-24}	2.82×10^{-24}
GO:MF	GO:0034987	immunoglobulin receptor binding	33/1808	76/17632	6.82	3.97×10^{-11}	3.93×10^{-11}
GO:MF	GO:0005096	GTPase activator activity	63/1808	279/17632	2.61	3.91×10^{-7}	3.88×10^{-7}
GO:MF	GO:0060589	nucleoside-triphosphatase regulator activity	74/1808	353/17632	2.38	3.91×10^{-7}	3.88×10^{-7}
GO:MF	GO:0030695	GTPase regulator activity	67/1808	309/17632	2.48	4.39×10^{-7}	4.35×10^{-7}
GO:MF	GO:0017171	serine hydrolase activity	60/1808	277/17632	2.47	2.62×10^{-6}	2.60×10^{-6}
GO:MF	GO:0008236	serine-type peptidase activity	59/1808	273/17632	2.46	3.22×10^{-6}	3.19×10^{-6}
GO:MF	GO:0004252	serine-type endopeptidase activity	54/1808	250/17632	2.45	1.13×10^{-5}	1.12×10^{-5}
GO:MF	GO:0017137	Rab GTPase binding	40/1808	185/17632	2.45	4.44×10^{-4}	4.40×10^{-4}
GO:MF	GO:0005201	extracellular matrix structural constituent	35/1808	155/17632	2.58	0.001	0.001
GO:MF	GO:0030020	extracellular matrix structural constituent conferring tensile strength	14/1808	37/17632	5.36	0.001	0.001
GO:MF	GO:0005089	Rho guanyl-nucleotide exchange factor activity	22/1808	80/17632	3.35	0.001	0.001

GO:MF	GO:0017048	Rho GTPase binding	35/1808	177/17632	2.18	0.008	0.008
GO:MF	GO:0005344	oxygen carrier activity	7/1808	14/17632	8.78	0.016	0.016
GO:MF	GO:0048407	platelet-derived growth factor binding	6/1808	11/17632	10.53	0.024	0.023
KEGG	hsa03008	Ribosome biogenesis in eukaryotes	32/798	118/8219	3.56	1.45×10^{-5}	1.40×10^{-5}
KEGG	hsa04974	Protein digestion and absorption	24/798	103/8219	2.88	0.004	0.004
KEGG	hsa04510	Focal adhesion	38/798	201/8219	2.23	0.004	0.004
KEGG	hsa04512	ECM-receptor interaction	20/798	88/8219	2.78	0.018	0.018
KEGG	hsa03010	Ribosome	30/798	167/8219	2.08	0.040	0.039
KEGG	hsa05150	Staphylococcus aureus infection	20/798	96/8219	2.48	0.040	0.039
DO	DOID:3437	laryngitis	7/767	11/8007	16.66	0.007	0.007
DO	DOID:786	laryngeal disease	7/767	11/8007	16.66	0.007	0.007

Note: GeneRatio denotes the ratio of input genes that are annotated in a term, and BgRatio denotes the ratio of all genes that are annotated in a term. The *P*-values are obtained by one-sided Fisher's exact test. They are further adjusted for multiple comparisons using the Benjamini-Hochberg procedure (denoted as the BH-adjusted *P*-value), and are also adjusted for the false discovery rate (denoted as the q-value).

Supplementary Table 11 Functional enrichment of genes affected by the CPC-specific SV hotspots.

Database	Term ID	Description	GeneRatio	BgRatio	Odds ratio	BH-adjusted *P*-value	*q*-value
GO:BP	GO:0015671	oxygen transport	6/534	15/18493	22.66	0.008	0.008
GO:BP	GO:0043087	regulation of GTPase activity	33/534	481/18493	2.57	0.008	0.008
GO:BP	GO:0043547	positive regulation of GTPase activity	29/534	405/18493	2.69	0.010	0.010
GO:BP	GO:0015669	gas transport	6/534	19/18493	15.69	0.011	0.011
GO:CC	GO:0031838	haptoglobin-hemoglobin complex	5/568	11/19659	28.25	0.003	0.003
GO:CC	GO:0005833	hemoglobin complex	5/568	12/19659	24.21	0.003	0.003
GO:MF	GO:0005344	oxygen carrier activity	6/521	14/17632	24.91	0.001	0.001
GO:MF	GO:0031720	haptoglobin binding	5/521	10/17632	33.15	0.002	0.002
GO:MF	GO:0030695	GTPase regulator activity	24/521	309/17632	2.85	0.004	0.003
GO:MF	GO:0060589	nucleoside-triphosphatase regulator activity	26/521	353/17632	2.70	0.004	0.003
GO:MF	GO:0019825	oxygen binding	7/521	36/17632	8.02	0.011	0.010
GO:MF	GO:0005096	GTPase activator activity	21/521	279/17632	2.74	0.011	0.010
GO:MF	GO:0004197	cysteine-type endopeptidase activity	12/521	118/17632	3.78	0.020	0.019
GO:MF	GO:0004843	thiol-dependent ubiquitin-specific protease activity	11/521	105/17632	3.90	0.025	0.023

Note: GeneRatio denotes the ratio of input genes that are annotated in a term, and BgRatio denotes the ratio of all genes that are annotated in a term. The *P*-values are obtained by one-sided Fisher's exact test. They are further adjusted for multiple comparisons using the Benjamini-Hochberg procedure (denoted as the BH-adjusted *P*-value), and are also adjusted for the false discovery rate (denoted as the q-value).

Supplementary Table 12 Human alpha globin genes haplotypes of CPC and HPRC haploid assemblies

Assembly	Population	Haplotype	Assembly	Population	Haplotype	Assembly	Population	Haplotype
CPC-HIFI032007D_1	Kinh	Z2A2	CPC-HIFI032604D_1	Khatso	Z2A2	HG01258_1	CLM	Z2A2
CPC-HIFI032007D_2	Kinh	Z3A2	CPC-HIFI032604D_2	Khatso	Z2A2	HG01258_2	CLM	Z2A3
CPC-HIFI032018D_1	Chosen	Z2A2	CPC-HIFI032607D_1	Mongol	Z2A2	HG01358_1	CLM	Z2A2
CPC-HIFI032018D_2	Chosen	Z2A2	CPC-HIFI032607D_2	Mongol	Z2A2	HG01358_2	CLM	Z2A2
CPC-HIFI032069D_1	Blang	Z2A2	CPC-HIFI032662D_1	Qiang	Z2A2	HG01361_1	CLM	Z2A2
CPC-HIFI032069D_2	Blang	Z2A2	CPC-HIFI032662D_2	Qiang	Z3A2	HG01361_2	CLM	Z2A2
CPC-HIFI032097D_1	Tujia	Z2A2	CPC-HIFI032668D_1	Daur	Z2A2	HG01891_1	ACB	Z2A2
CPC-HIFI032097D_2	Tujia	Z2A2	CPC-HIFI032668D_2	Daur	Z2A2	HG01891_2	ACB	Z2A2
CPC-HIFI032103D_1	Tujia	Z2A2	CPC-HIFI032682D_1	Achang	Z2A2	HG01928_1	PEL	Z2A2
CPC-HIFI032103D_2	Tujia	Z2A2	CPC-HIFI032682D_2	Achang	Z2A1	HG01928_2	PEL	Z2A2
CPC-HIFI032161D_1	Mosuo	Z2A3	CPC-HIFI032685D_1	Hezhen	Z2A2	HG01952_1	PEL	Z2A2
CPC-HIFI032161D_2	Mosuo	Z2A2	CPC-HIFI032685D_2	Hezhen	Z2A2	HG01952_2	PEL	Z2A2
CPC-HIFI032164D_1	Mongol	Z2A2	CPC-HIFI032692D_1	Yugur	Z2A2	HG01978_1	PEL	Z2A2
CPC-HIFI032164D_2	Mongol	Z2A2	CPC-HIFI032692D_2	Yugur	Z2A2	HG01978_2	PEL	Z2A2
CPC-HIFI032167D_1	Miao	Z2A2	CPC-HIFI032693D_1	Naxi	Z2A2	HG02055_1	ACB	Z2A2
CPC-HIFI032167D_2	Miao	Z2A2	CPC-HIFI032693D_2	Naxi	Z2A2	HG02055_2	ACB	Z2A2
CPC-HIFI032182D_1	Yi	Z2A2	CPC-HIFI032698D_1	Daur	Z2A2	HG02080_1	KHV	Z2A1
CPC-HIFI032182D_2	Yi	Z2A2	CPC-HIFI032698D_2	Daur	Z2A2	HG02080_2	KHV	Z2A2
CPC-HIFI032289D_1	Man	Z2A2	CPC-HIFI032706D_1	Evenki	Z2A2	HG02109_1	ACB	Z2A1
CPC-HIFI032289D_2	Man	Z2A2	CPC-HIFI032706D_2	Evenki	Z2A2	HG02109_2	ACB	Z2A1
CPC-HIFI032292D_1	Deang	Z2A1	CPC-HIFI032711D_1	Wa	Z2A2	HG02145_1	ACB	Z2A2
CPC-HIFI032292D_2	Deang	Z2A2	CPC-HIFI032711D_2	Wa	Z2A2	HG02145_2	ACB	Z2A2
CPC-HIFI032302D_1	She	Z2A2	CPC-HIFI032731D_1	Lisu	Z2A2	HG02148_1	PEL	Z2A2
CPC-HIFI032302D_2	She	Z2A2	CPC-HIFI032731D_2	Lisu	Z2A2	HG02148_2	PEL	Z2A2
CPC-HIFI032306D_1	Chosen	Z2A2	CPC-RY01_1	Yao	Z2A2	HG02257_1	ACB	Z2A2
CPC-HIFI032306D_2	Chosen	Z2A2	CPC-RY01_2	Yao	Z2A2	HG02257_2	ACB	Z2A2
CPC-HIFI032335D_1	Oroqen	Z2A2	CPC-RY02_1	Uyghur	Z2A2	HG02486_1	ACB	Z2A2
CPC-HIFI032335D_2	Oroqen	Z2A2	CPC-RY02_2	Uyghur	Z2A3	HG02486_2	ACB	Z2A2
CPC-HIFI032349D_1	Tu	Z2A2	CPC-RY03_1	She	Z2A2	HG02559_1	ACB	Z2A2
CPC-HIFI032349D_2	Tu	Z2A2	CPC-RY03_2	She	Z2A2	HG02559_2	ACB	Z2A2
CPC-HIFI032373D_1	Blang	Z2A2	CPC-RY04_1	Miao	Z2A2	HG02572_1	GWD	Z2A2
CPC-HIFI032373D_2	Blang	Z2A1	CPC-RY04_2	Miao	Z2A2	HG02572_2	GWD	Z2A2
CPC-HIFI032420D_1	Qiang	Z2A2	CPC-RY05_1	Chosen	Z2A2	HG02622_1	GWD	Z2A2
CPC-HIFI032420D_2	Qiang	Z2A2	CPC-RY05_2	Chosen	Z2A2	HG02622_2	GWD	Z2A2
CPC-HIFI032422D_1	Mosuo	Z2A2	CPC-RY06_1	Kyrgyz	Z2A2	HG02630_1	GWD	Z2A2
CPC-HIFI032422D_2	Mosuo	Z2A2	CPC-RY06_2	Kyrgyz	Z2A2	HG02630_2	GWD	Z2A2
CPC-HIFI032429D_1	Jino	Z2A2	CPC-RY07_1	Kazakh	Z2A2	HG02717_1	GWD	Z2A2
CPC-HIFI032429D_2	Jino	Z2A2	CPC-RY07_2	Kazakh	Z2A2	HG02717_2	GWD	Z2A2
CPC-HIFI032440D_1	Jingpo	Z2A2	CPC-RY08_1	Zhuang	Z2A3	HG02723_1	GWD	Z2A2
CPC-HIFI032440D_2	Jingpo	Z2A2	CPC-RY08_2	Zhuang	Z2A2	HG02723_2	GWD	Z2A2
CPC-HIFI032450D_1	Drung	Z2A2	CPC-RY09_1	Hui	Z2A2	HG02818_1	GWD	Z2A2
CPC-HIFI032450D_2	Drung	Z2A2	CPC-RY09_2	Hui	Z2A2	HG02818_2	GWD	Z2A2
CPC-HIFI032453D_1	Salar	Z2A2	CPC-RY10_1	Mongol	Z2A2	HG02886_1	GWD	Z2A2
CPC-HIFI032453D_2	Salar	Z2A2	CPC-RY10_2	Mongol	Z2A2	HG02886_2	GWD	Z2A2
CPC-HIFI032454D_1	Tu	Z2A2	CPC-RY11_1	Tujia	Z2A2	HG03098_1	MSL	Z2A2
CPC-HIFI032454D_2	Tu	Z2A1	CPC-RY11_2	Tujia	Z2A2	HG03098_2	MSL	Z2A2
CPC-HIFI032462D_1	Deang	Z2A2	HG00438_1	CHS	Z2A2	HG03453_1	MSL	Z2A1
CPC-HIFI032462D_2	Deang	Z2A2	HG00438_2	CHS	Z2A2	HG03453_2	MSL	Z2A1
CPC-HIFI032473D_1	Dong	Z2A0	HG00621_1	CHS	Z2A2	HG03486_1	MSL	Z2A2
CPC-HIFI032473D_2	Dong	Z2A2	HG00621_2	CHS	Z2A2	HG03486_2	MSL	Z2A2
CPC-HIFI032487D_1	Bouyei	Z2A2	HG00673_1	CHS	Z2A2	HG03492_1	PJL	Z2A2
CPC-HIFI032487D_2	Bouyei	Z2A1	HG00673_2	CHS	Z2A2	HG03492_2	PJL	Z2A2
CPC-HIFI032501D_1	Mosuo	Z2A2	HG00733_1	PUR	Z2A2	HG03516_1	ESN	Z2A2
CPC-HIFI032501D_2	Mosuo	Z2A2	HG00733_2	PUR	Z2A2	HG03516_2	ESN	Z2A2
CPC-HIFI032510D_1	Dong	Z2A2	HG00735_1	PUR	Z2A3	HG03540_1	GWD	Z2A2
CPC-HIFI032510D_2	Dong	Z2A2	HG00735_2	PUR	Z2A2	HG03540_2	GWD	Z2A2
CPC-HIFI032513D_1	Kinh	Z2A2	HG00741_1	PUR	Z2A2	HG03579_1	MSL	Z2A2
CPC-HIFI032513D_2	Kinh	Z2A2	HG00741_2	PUR	Z2A2	HG03579_2	MSL	Z2A2
CPC-HIFI032529D_1	Wa	Z2A2	HG01071_1	PUR	Z2A2	NA18906_1	YRI	Z2A2
CPC-HIFI032529D_2	Wa	Z2A2	HG01071_2	PUR	Z2A2	NA18906_2	YRI	Z2A2
CPC-HIFI032566D_1	Yugur	Z2A2	HG01106_1	PUR	Z2A2	NA20129_1	ASW	Z2A2
CPC-HIFI032566D_2	Yugur	Z2A2	HG01106_2	PUR	Z2A2	NA20129_2	ASW	Z2A2
CPC-HIFI032567D_1	Qiang	Z2A2	HG01109_1	PUR	Z2A2	NA21309_1	HAPMAP-MAASAI_IN_KINYAWA,KENYA	Z2A2
CPC-HIFI032567D_2	Qiang	Z2A2	HG01109_2	PUR	Z2A2	NA21309_2	HAPMAP-MAASAI_IN_KINYAWA,KENYA	Z2A2
CPC-HIFI032585D_1	Bai	Z2A2	HG01123_1	CLM	Z2A2			
CPC-HIFI032585D_2	Bai	Z3A3	HG01123_2	CLM	Z2A2			
CPC-HIFI032586D_1	Salar	Z2A2	HG01175_1	PUR	Z2A2			
CPC-HIFI032586D_2	Salar	Z3A2	HG01175_2	PUR	Z2A2			
CPC-HIFI032591D_1	She	Z2A2	HG01243_1	PUR	Z2A2			
CPC-HIFI032591D_2	She	Z2A2	HG01243_2	PUR	Z2A2			

Supplementary Table 13 Copy number of *RASA4(B)* genes in CPC samples

Assembly	Ethnic group	RASA4(B) Dosage	Assembly	Ethnic group	RASA4(B) Dosage
HG00438.1	Han	5copy	HIFI032487D.1	Bouyei	4copy
HG00438.2	Han	5copy	HIFI032501D.1	Mosuo	3copy+partial
HG00621.1	Han	4copy+partial	HIFI032501D.2	Mosuo	4copy
HG00621.2	Han	1copy+partial	HIFI032510D.1	Dong	4copy
HIFI032007D.1	Kinh	1copy+partial	HIFI032510D.2	Dong	2copy
HIFI032007D.2	Kinh	6copy	HIFI032513D.1	Kinh	3copy+partial
HIFI032018D.1	Chosen	4copy+partial	HIFI032513D.2	Kinh	3copy+partial
HIFI032018D.2	Chosen	4copy	HIFI032529D.2	Wa	2copy
HIFI032097D.1	Tujia	3copy	HIFI032566D.1	Yugur	2copy
HIFI032097D.2	Tujia	2copy	HIFI032566D.2	Yugur	3copy+partial
HIFI032103D.1	Tujia	3copy+partial	HIFI032567D.1	Qiang	2copy+partial
HIFI032103D.2	Tujia	2copy	HIFI032567D.2	Qiang	2copy
HIFI032161D.1	Mosuo	5copy	HIFI032585D.1	Bai	2copy
HIFI032161D.2	Mosuo	3copy+partial	HIFI032585D.2	Bai	6copy
HIFI032167D.1	Miao	6copy	HIFI032586D.1	Salar	2copy
HIFI032289D.1	Man	6copy	HIFI032586D.2	Salar	2copy
HIFI032289D.2	Man	4copy+partial	HIFI032591D.1	She	2copy
HIFI032292D.1	Deang	4copy+partial	HIFI032591D.2	She	2copy
HIFI032292D.2	Deang	2copy+partial	HIFI032604D.1	Khatso	5copy
HIFI032302D.1	She	2copy	HIFI032604D.2	Khatso	2copy
HIFI032302D.2	She	5copy	HIFI032607D.1	Mongol	2copy
HIFI032306D.1	Chosen	3copy+partial	HIFI032607D.2	Mongol	5copy
HIFI032306D.2	Chosen	3copy	HIFI032662D.1	Qiang	2copy
HIFI032335D.1	Oroqen	4copy	HIFI032662D.2	Qiang	2copy
HIFI032335D.2	Oroqen	1copy+partial	HIFI032668D.1	Daur	5copy
HIFI032349D.1	Tu	1copy+partial	HIFI032682D.1	Achang	3copy+partial
HIFI032349D.2	Tu	3copy+partial	HIFI032682D.2	Achang	5copy
HIFI032373D.2	Blang	4copy+partial	HIFI032685D.1	Hezhen	2copy
HIFI032420D.1	Qiang	3copy+partial	HIFI032685D.2	Hezhen	2copy
HIFI032420D.2	Qiang	4copy+partial	HIFI032692D.1	Yugur	3copy+partial
HIFI032422D.1	Mosuo	1copy+partial	HIFI032692D.2	Yugur	3copy+partial
HIFI032422D.2	Mosuo	3copy+partial	HIFI032693D.1	Naxi	4copy
HIFI032429D.1	Jino	3copy	HIFI032693D.2	Naxi	1copy+partial
HIFI032440D.1	Jingpo	1copy+partial	HIFI032698D.1	Daur	4copy
HIFI032450D.1	Drung	5copy	HIFI032698D.2	Daur	1copy+partial
HIFI032450D.2	Drung	2copy	HIFI032706D.1	Evenki	3copy+partial
HIFI032453D.1	Salar	2copy	HIFI032706D.2	Evenki	5copy
HIFI032453D.2	Salar	3copy+partial	HIFI032711D.1	Wa	5copy
HIFI032454D.2	Tu	5copy	HIFI032711D.2	Wa	2copy+partial
HIFI032462D.1	Deang	3copy+partial	HIFI032731D.1	Lisu	4copy+partial
HIFI032462D.2	Deang	3copy+partial	HIFI032731D.2	Lisu	4copy+partial
HIFI032473D.1	Dong	4copy+partial	RY06.1	Kyrgyz	2copy
RY01.1	Yao	3copy+partial	RY06.2	Kyrgyz	2copy
RY01.2	Yao	5copy	RY07.1	Kazakh	2copy
RY02.1	Uyghur	4copy+partial	RY07.2	Kazakh	3copy+partial
RY03.1	She	3copy+partial	RY08.1	Zhuang	2copy
RY03.2	She	4copy	RY08.2	Zhuang	2copy
RY04.1	Miao	1copy+partial	RY09.1	Hui	2copy+partial
RY04.2	Miao	4copy	RY09.2	Hui	3copy+partial
RY05.1	Chosen	4copy	RY10.1	Mongol	3copy+partial
RY05.2	Chosen	3copy	RY10.2	Mongol	5copy
			RY11.1	Tujia	3copy+partial
			RY11.2	Tujia	3copy+partial

Supplementary Table 14 Counts of haplotypes with different *RASA4(B)* copy number in different populations

	1copy+partial	2copy	2copy+partial	3copy	3copy+partial	4copy	4copy+partial	5copy
Austro-Asiatic	1	1	2	0	4	0	2	1
Turkic	1	7	0	0	6	0	1	1
Hmong-Mien	1	3	0	0	2	2	0	2
Mongolic	1	1	0	0	1	1	0	3
Sino-Tibetan	4	8	2	2	9	2	4	6
Koreanic	0	0	0	2	1	2	1	0
Tungusic	1	2	0	0	1	1	1	1
Tai-Kadai	0	3	0	0	0	2	1	0

Note: reference assembly GRCh38 is with 2 copy of RASA4(B) and CHM13 is with 2 copy and a 14.9 Kb fragment of RASA4(B).

Supplementary Table 15 Frequnecy distribution of *RASA4(B)* copy number in different populations

	1copy+partial	2copy	2copy+partial	3copy	3copy+partial	4copy	4copy+partial	5copy
Austro-Asiatic	0.0833	0.0833	0.1667	0	0.3333	0	0.1667	0.0833
Turkic	0.0625	0.4375	0	0	0.3750	0	0.0625	0.0625
Hmong-Mien	0.0909	0.2727	0	0	0.1818	0.1818	0	0.1818
Mongolic	0.1429	0.1429	0	0	0.1429	0.1429	0	0.4286
Sino-Tibetan	0.1053	0.2105	0.0526	0.0526	0.2368	0.0526	0.1053	0.1579
Koreanic	0	0	0	0.3333	0.1667	0.3333	0.1667	0
Tungusic	0.1250	0.2500	0	0	0.1250	0.1250	0.1250	0.1250
Tai-Kadai	0	0.5000	0	0	0	0.3333	0.1667	0

Supplementary Table 16 Enrichment of novel SVs around the GWAS loci.

Phenotype	% Segments around the GWAS loci (%)		
	Novel	Common (median and quartiles)	*P*-value
Analysis of the GWAS loci reported in global populations			
Height	50.7	[48.4,49.4]	0.044
Metabolite levels	12.4	[11.1,11.8]	0.048
Red blood cell count	9.4	[7.9,8.5]	0.043
Total PHF-tau (SNP x SNP interaction)	9.2	[7.9,8.4]	0.044
Core binding factor acute myeloid leukemia	9.0	[7.2,7.8]	0.042
Blood protein levels	8.6	[7.2,7.8]	0.043
Triglyceride levels	7.2	[6.2,6.7]	0.048
Obesity-related traits	6.7	[5.5,5.9]	0.042
Mean platelet volume	5.9	[4.6,5.0]	0.042
Mean spheric corpuscular volume	5.7	[4.6,5.0]	0.042
Mean reticulocyte volume	4.6	[3.6,4.0]	0.044
Apolipoprotein A1 levels	4.6	[3.5,3.9]	0.042
High light scatter reticulocyte percentage of red cells	4.5	[3.6,4.0]	0.043
Reticulocyte count	4.4	[3.6,4.0]	0.047
Monocyte percentage of white cells	4.3	[3.1,3.5]	0.042
Hemoglobin levels	3.3	[2.6,2.9]	0.044
DNA methylation variation (age effect)	3.3	[2.7,3.0]	0.049
Serum total protein level	3.3	[2.6,3.0]	0.048
Creatinine levels	3.3	[2.5,2.8]	0.045
Electrocardiogram morphology (amplitude at temporal datapoints)	3.1	[2.4,2.6]	0.043
Multiple sclerosis	2.9	[2.2,2.5]	0.045
Gut microbiota (bacterial taxa, hurdle binary method)	2.7	[2.0,2.3]	0.045
Liver enzyme levels (alkaline phosphatase)	2.7	[2.0,2.3]	0.043
Rheumatoid arthritis	2.2	[1.7,1.9]	0.046
Carotid Intima-media thickness (mean of the maximum cIMT)	2.1	[1.6,1.8]	0.050
COVID-19 (covid vs negative)	2.0	[1.3,1.5]	0.042
Non-melanoma skin cancer	1.9	[1.3,1.6]	0.046
Lymphocyte-to-monocyte ratio	1.8	[1.2,1.4]	0.044
Chronic inflammatory diseases (ankylosing spondylitis, Crohn's disease, psoriasis, primary sclerosing cholangitis, ulcerative colitis) (pleiotropy)	1.7	[1.1,1.4]	0.042
Nicotine dependence symptom count	1.7	[1.1,1.4]	0.045
Rate of cognitive decline in Alzheimer's disease	1.5	[1.1,1.3]	0.048
Age at menopause	1.4	[1.0,1.2]	0.046
COVID-19 (hospitalized vs not hospitalized)	1.2	[0.9,1.0]	0.047
Ebbinghaus illusion (overestimation)	1.2	[0.7,0.9]	0.043
Basal cell carcinoma	1.1	[0.7,0.8]	0.043
Average diameter for HDL particles	1.1	[0.6,0.8]	0.042
Gut microbiota (bacterial taxa, rank normal transformation method)	1.1	[0.7,0.9]	0.044
Cholesterol levels in large HDL	1.1	[0.7,0.9]	0.045
Cholesteryl ester levels in large HDL	1.1	[0.7,0.9]	0.045
Squamous cell lung carcinoma	1.1	[0.7,0.9]	0.047
Lung cancer in ever smokers	1.1	[0.6,0.8]	0.044
Concentration of large HDL particles	1.1	[0.7,0.8]	0.046
Urinary metabolite levels in chronic kidney disease	1.0	[0.7,0.8]	0.045
Amyotrophic lateral sclerosis (sporadic)	1.0	[0.6,0.8]	0.042
Phospholipid levels in large HDL	1.0	[0.6,0.8]	0.044
Analysis of the GWAS loci reported in East Asian populations			
Height	47.7	46.0 [45.5,46.5]	0.045
Red blood cell count	5.1	4.5 [4.3,4.7]	0.049
Lymphocyte count	4.3	3.7 [3.5,3.8]	0.043
Neutrophil count	3.8	3.1 [3.0,3.3]	0.043
Aspartate aminotransferase levels	3.2	2.7 [2.5,2.8]	0.045
Alanine aminotransferase levels	2.6	2.1 [2.0,2.2]	0.045
Mean corpuscular hemoglobin concentration	1.9	1.5 [1.4,1.6]	0.046
Creatinine levels	1.4	1.0 [1.0,1.1]	0.045
Ebbinghaus illusion (overestimation)	1.2	0.8 [0.7,0.9]	0.043
Apolipoprotein A1 levels	1.1	0.8 [0.7,0.9]	0.044
High light scatter reticulocyte count	1.1	0.8 [0.7,0.8]	0.044
Reticulocyte fraction of red cells	1.0	0.7 [0.7,0.8]	0.044

Note: Proportion of the novel loci located < 50kb around the GWAS variants was compared with 100 sets of randomly sampled common loci with matched size. Here shows the raw *P*-values obtained by the one-sided Wilcoxon rank-sum test. None of them remains significant after adjusting for multiple comparisons (BH-adjusted $P > 0.05$).

Supplementary Table 17 Genes and Frequencies of CDS Regions Covered by CPC Archaic Introgression Segments

Frequency	Genes
60	*PALM2AKAP2*
58	*TXNDC8*
57	*HHAT*
56	*TXN,TBC1D1*
55	*POU2F3,NLRC5,CTNNA3*
53	*NRXN3*
52	*SIPA1L2*
51	*SNED1,C9orf152*
50	*SVEP1,STAB2,CSMD2*
49	*OAF*
48	*KRT82,IL17RA,COL27A1,BOD1*
47	*UTP25,SYT14,MACROD2,IRF6,ENSG00000289700*
46	*SERTAD4,PTPN14,EEF1A2,CAPN11,ANKS1B*
45	*ZFP37,OSBPL10,LRRTM3,KIAA1217,HELZ2*
44	*ZMYND10,USH2A,TUSC2,TMEM115,SMYD2,SLC38A3,SEMA3B,RASSF1,NPRL2,NAA80,LSMEM2,KRT85,KRT84,KLF3,KCNQ2,IFRD2,HYAL3,HYAL2,HYAL1,GNAI2,ENSG00000272104,DNAJC12,CYB561D2,CACNA2D2*
43	*TRIM62,SEMA3F,HSD11B1,GNAT1,GMEB2,ENSG00000277971,CTNND2,CHST15,C12orf42,AK2*
42	*ZFAND1,TRAF3IP3,SLC10A5,PTTG2,ONECUT2,EPC2,CNTN5,C1orf74*
41	*RHPN2,RBM19,PTPRN2,G0S2,CDH23,AZIN2*
40	*UTRN,TSPAN11,SASH1,MUSK,KRT75,KRT6C,KRT6B,KRT6A,KLHL22,FKBP15,CROCC2,CAMK2D*
39	*TENM3,TBX5,SLC6A5,SLC35F3,SIRT1,SCARF2,NCOR2,MYBPC1,HERC4,CERS6*
38	*TMTC1,RIMBP2,PRDM16,PPDPF,NXN,NT5DC3,NELL1,JHY,HSP90B1,ENSG00000289325,DDX1,CRYBG1,AGBL1*
37	*ZNF362,WWOX,SLC35F4,SH3RF2,PTGR1,OSTN,LAMB3,KRT81,HECW2,GRK5,GLP1R,EYA4,ETFBKMT,CPNE4,BORCS5*
36	*ZBTB43,NAV2,MED15,KLF10,IL17F,FAT1,DKK3,CAMK1G,ABCB11*
35	*UTS2B,TDG,SPATA2,SMLR1,SLC31A2,SEMA3C,RNF114,RAB18,PTPN3,PPP3R1,PCSK6,MEI4,GLT8D2,FAIM2,ENSG00000273398,DNAAF10,CRTAM,CCDC80,C14orf132,C12orf73,ANTXR1,AMN1*
34	*ZNF444,UBASH3B,TACC2,SYK,SLC9A8,SAYSD1,RNF19B,RANBP3L,NWD2,MAP3K1,KRT5,ITGB6,GLB1,GALP,FKSG48,ENSG00000275740,DNAH11,CPNE7,COL14A1,CCDC50,ADAMTS7*
33	*ZNF483,WASHC3,TMPO,ST6GAL1,ST20-MTHFS,SMIM35,SLC17A6,RBMS1,PNO1,PMCH,PGM2,PARPBP,OSMR,NUP37,NRIP1,NEBL,MYPN,MTHFS,FXYD6-FXYD2,FRMD3,EPB41L2,CTSH,CSTA,COL13A1,CLYBL,ADRA1D,AC024580.1*
32	*ZNRF4,TWIST2,TLR6,TLR1,STK17B,PLA2G2A,LMX1B,LHFPL3,KCNK1,DNAH7,COL12A1,BTBD16*
31	*TMEM121B,TIAM1,SPATA13,SLC39A10,ROR2,PTCHD3,MROH7-TTC4,MORF4L1,LY75-CD302,LY75,LMX1A,KRT86,KRT83,GABBR2,FZD10,EPB41L4B,ENSG00000287694,DROSHA,CSNK1A1,CNTNAP4,CMTM8,CHRNB4,BCL2A1,BAZ2B,A3GALT2*
30	*TRA2B,RELL1,PYDC2,PRKCH,PANK1,NFKBIE,NEDD9,MROH7,MAGI2,LDLRAD3,ITPR2,IGF1,HTATIP2,FHL2,DAPK1,CHFR,CDH15,CACNA2D3,ARHGAP42*
29	*SSR1,SSPN,SMOX,SLC22A31,SKP2,RASGRF1,PASK,NPAS3,NIM1K,NADK2,KCNH1,IMPG1,IL10RA,GRM1,GPD1L,GOLGA3,GHR,ENSG00000284299,ECPAS,DGKB,CSMD1,CHD5,CCDC102B,C1QTNF9B,ATP2C1,ANKLE2*
28	*ZNF778,TRIM39-RPP21,SLC35B2,SLC31A1,RPP21,REEP3,PRPF4,PLXNA2,PLPP3,PLD5,OSBPL6,MYO3B,MRPS15,LEPROTL1,JMJD1C,HSP90AB1,GRIN2B,GNPTAB,FYB2,FXYD6,EGR2,DRAM1,DOK6,CSF3R,CDC26,CALB2,ARHGEF37,ANKRD22*
27	*WDR31,UBE2H,TLL1,SYCP3,STT3B,SRRM4,SLC25A3,ROS1,PRMT3,PLK4,PLA2R1,P2RX6,OSCP1,OR2K2,NRBF2,NDST1,NALCN,MYOCD,MMP17,MFSD8,MACROH2A2,LIPM,LARP1B,KSR2,ITGA6,IRAG2,HTR4,HSPA4L,HK1,GRID2,ESRRG,ENSG00000285837,CPNE2,CNRIP1,CHPT1,ASTN2,APAF1,ABHD18*
26	*ZNF131,WARS2,SLC25A31,RGS10,PPP1R7,PLAC8L1,PDSS2,P2RX2,OVCH1,NNT,MMP20,MBOAT4,MANSC1,KIF16B,KAZN,INTU,IKBIP,GAS7,FMN1,FAM102A,FAH,CGNL1,CDC5L,BSPRY,ANO7,ANO5,ANKRD26,ACBD5*
25	*ZFHX3,TUNAR,TMOD1,TMEM63B,TIMM21,THAP4,SLC28A3,SERPINB13,NUDT7,MRPL14,MECOM,LIFR,KIF20B,INTS13,GLDN,GCLC,FOS,FBXO15,ENSG00000273167,EGF,CPLX4,COL25A1,CAPN8,CAPN2,C5orf22,C2orf49,ATG4B,ARRDC4,ARID5B*
24	*YME1L1,USP47,TRIM32,TLR10,TGFBRAP1,SPATS1,RAB3C,PXYLP1,POU4F3,PLA2G5,PIP5KL1,MCPH1,MASTL,LIMD1,KRT2,KCNQ5,ING5,HEXB,GRM7,GRIN3A,FOXO6,EPHX1,ELOVL6,DYSF,DPM2,CHRNA5,CHRNA3,B9D1,ATOH8,ANXA2R,ADO,ABLIM3*
23	*ZFAND6,TUT4,TTN,TNFRSF19,TLE3,STRA8,SLC24A4,RNF39,RBM27,RASSF8,PRRT1,PPT2-EGFL8,PPT2,PPP1R11,POLR1H,NPAS2,MYL7,MMP7,LARGE1,KALRN,HMX2,GCK,FGD3,EPN2,ENSG00000285085,DNAJC25-GNG10,DNAJC25,DNAJC13,DNAH8,D2HGDH,CTXND1,CDKN2B,BUB3,ARHGEF16,ARHGAP44,ARHGAP26,ANK2*
22	*ZNF727,ZNF722,ZNF354B,WRAP73,TRIM71,TRIM31,TPRG1L,TMEM54,SYNPO,SLC4A10,SLC16A12,SLC15A4,RNF122,PSMA4,PREP,PGRMC2,PCLO,OR10P1,MCM3,LARS1,KIAA1958,IL2RA,IL17RB,HYKK,HPCA,EYS,ENSG00000285330,DHRS3,DECR1,CPE,CD200R1L,CD200R1,CALB1,CACNA1D,C8orf48,ARSJ,AMPH,ADGB,ACSF3*
21	*ZNF860,ZNF787,UNC5B,TMEM132C,TMED3,THAP7,SYDE2,STAMBPL1,SRMS,SMARCAL1,SLC9A9,SINHCAF,SELENOP,RIN3,RELN,PXMP2,PTK6,PTGIR,PPARGC1B,POLE,POLD2,PGAM5,PDZD2,PDK1,NEK2,MTERF4,LZTR1,KRT74,KRT1,JAKMIP2,IFIH1,HSD17B3,FSTL5,FNDC11,FAM241B,DUSP16,CRTC3,CGAS,CELA1,CCDC152,C20orf27,AKAP7,AEBP1,ADAMTS9,ADAMTS18,AC002472.1*
20	*ZNF454,ZFP69B,ZFP2,USP6NL,USP53,TUBGCP6,TRPC6,TMPRSS4,TMEM132D,TINAGL1,SUCLG2,SLC28A1,RTN4IP1,RASL10B,RAD51B,PRDM5,PPP2R5E,OTUD1,NELL2,NDUFB1,KRT72,KCNQ1,HDAC10,GPR31,GAS2L2,GAR1,GALNT12,FAT2,ESPNL,DYNC2I1,DNAI3,DDX60L,DBX2,CPSF2,CDKN2A,APP,ANAPC4,ALPK3*

Frequency	Genes
19	ZNF891,ZNF592,YARS1,VSTM2B,TNXB,TNS2,TMEM248,SYNPO2,STK32A,SRP9,SPINK4,SORL1,SMAD3,SELENOO,SCMH1,S100PBP,RALGPS1,QRSL1,PYGL,PRKCQ,PMP22,PI4K2B,NPHP3-ACAD11,NPHP3,NEPRO,MYO16,MRPS35,MN1,MGAT5,KRT73,KIAA1522,IMPA1,IGSF8,IGFBP6,HTR1B,HEYL,HECW1,GTPBP8,GRM6,GRID1,GLT1D1,GARNL3,GALNT18,FNDC5,FBRSL1,ENG,DPYSL3,DDX43,CWF19L2,CFI,BIN2,ARHGEF10L
18	WDR91,ULK4,UGT3A2,TRAK1,TP73,TMEM151B,TLE1,TCTE1,TCL1A,TBC1D4,TBATA,SYNPR,STRIT1,SPRYD3,SOBP,SNAI1,SLFNL1,SLC30A10,RNF183,PYCR2,PTCH1,PPFIBP1,PLEKHH1,PLEKHA1,PCSK2,PBX3,NTRK2,MYO6,MME,LEFTY2,KRT77,KCNA6,JAG1,HDLBP,GNG10,GCG,GALNT6,FGF10,FAP,ESYT2,ENSG00000255835,DUSP26,DHRS9,CTPS1,CCDC27,C4orf19,ATRNL1,AIFM2,ACTA2,ACADSB
17	ZNF84,ZNF605,ZNF26,ZNF140,ZCCHC4,ZBTB34,WDR72,UBA5,TTC4,TTC29,TTC22,TP63,TP53I11,TMEM267,TM2D3,TBR1,TAS1R2,TARS3,SP140,SMAD7,SLAMF6,SF3B5,SENP6,SEC62,SCML4,SAMD7,SAMD4A,RSPRY1,RPUSD1,ROBO2,RBM17,QRFPR,PSME3IP1,PRKAG2,PFKFB3,PDZRN4,PCDH7,PARS2,PAPPA,OOEP,NME8,NALF1,MYOZ2,MS4A13,MROH2B,MAPK12,IQGAP1,IFT140,HTRA1,HSDL2,HMGCS1,HDHD5,GABRB3,GABRA5,GAB4,ENSG00000283952,ENSG00000272442,EFCAB13,CXADR,COL24A1,CCNE1,C7,ATXN7L1,AP2B1,ANO6,ACAD11,AARS2
16	XPA,XIRP2,WSCD1,VASH2,UGCG,TSTD2,TRIM39,TNFSF8,TMEM88,TMEM63A,TM4SF18,THSD7B,TELO2,SPP2,SMIM38,SLFN14,SLC52A3,SHOC1,SEPTIN2,SELENOK,SCARA5,RP13-279N23.2,RHBDL2,RBFOX1,RASSF4,PSTK,PRKRA,PPP1R26,PPM1H,PLEKHG1,PJVK,PAIP1,OR6B3,ONECUT1,OMA1,NOSTRIN,NLRP5,NHSL1,NFYB,NCBP1,NCAPG2,NAA38,MFGE8,MEGF6,MATN2,MARCHF7,MAP3K5,LUZP2,LMBRD2,LIPI,LINGO2,LEMD1,IFFO2,HSPA8,HIVEP1,HCFC2,GJA9,FXYD2,FLT4,FKBPL,FKBP7,FILIP1,FARP2,ENSG00000274944,DIS3L,CYB5D1,CTSL,CLMP,CLDN11,CD302,CCL28,C5orf34,BMP10,ATP1A4,ATF6B,ANGPTL2,ALPK1,AGBL4,ADARB2,ACTR8,ABCG1
15	ZNF774,ZNF684,ZFP69,XXYLT1,WNK2,VNN1,VEGFA,USP5,UBE2F-SCLY,UBE2F,UBD,TSPAN18,TRABD,TNC,TMPRSS13,TM7SF3,SUSD3,STK25,SLC41A2,SLC17A8,SKIL,SIGLEC1,SETD9,SCEL,RRAGC,RNASET2,REP15,RARG,PUS7,PSMD14,PRKCI,PRKAA2,PIGH,PEX12,OR2I1P,NLRP7,NKAIN1,NDFIP1,MYCBP,MMP8,MMP27,MIER3,MAPKAP1,ING2,IL15RA,IKZF5,HMGB4,H3-5,FZD3,FPGS,FOXE3,FJX1,FGFR1OP2,FAM114A2,EXO5,ENSG00000285218,ENSG00000283877,ENSG00000249141,EEF1A1,DRICH1,DNAI7,CXCL12,COX7A2,COMMD2,CLEC4E,CLEC4D,CDCA3,CD74,CD36,CD226,CCND2,CAND1,BRD1,ATG5,ARMS2,ARHGEF3,ANKUB1,ALDH1L2,ACKR4
14	ZNF823,ZNF740,ZNF248,WDR64,WDR49,TRIT1,TRGV10,TMEM72,TMEM268,TMEM14A,TMCO4,TEKT3,TDGF1,TBC1D32,SUSD4,SPSB4,SOAT2,SLC38A1,SHANK2,SGCZ,SEC23IP,SCLY,SATB1,RTP3,RPL30,RICTOR,RASGEF1A,RAMP1,PTCD3,PSD3,POLR1A,PLCZ1,PDE4D,PCNX2,NTRK3,NOTCH4,MYO1G,MYMX,MCMBP,MAPK11,MAN1A1,LRRC2,LIPA,LEFTY1,KRT71,KLF18,KLF17,KCNH7,ITGB7,INPP5F,IMMT,IFNE,GSTA2,GPSM3,GPR142,GPBP1,GM2A,GKN1,GAL3ST2,FOXD2,FBXL3,FAT4,FAM78B,FAM120A,ERI3,ENSG00000288711,ELAC2,DSCAML1,DOCK2,DMAP1,DIRAS2,DDAH1,CTDP1,CSGALNACT2,CSAD,CPB1,CPA3,COL19A1,CLN5,CLEC3A,CEP43,CDK9,CCSER1,CCR4,CCDC69,CACNA2D1,BLACAT1,BAG3,ATXN3,ARID3A,ADAMTS14,ACBD3
13	ZNFX1,ZNF717,ZNF484,ZNF25,ZCRB1,YIPF1,VEZT,TYW1,TMEM204,TMED4,SUGCT,STAT1,SPOCK3,SLC47A1,SHC2,SH3TC2,SESTD1,RRH,RRBP1,RET,RCAN1,PTPRF,PRKN,PRICKLE1,PREX1,PRDM2,PPHLN1,PLK2,PHF2,PDLIM5,PALM,OLIG3,OGDH,NOPCHAP1,NFIA,NEUROG3,NEU4,NECTIN1,MYNN,MTMR6,MSR1,MGST1,LRRC34,LRRC23,LGMN,KRAS,KCNB1,IFNA1,IFIT1B,IFIT1,HSPA12B,GRK7,GRB10,GAPT,GALNT9,FYB1,FRMD4A,FRK,FOXP4,FASTKD1,FAM120AOS,ETFRF1,ENSG00000285558,ENSG00000282218,ENO2,EIF4B,DYNC2H1,DIO1,DDX56,DDX27,COPS9,CNOT11,CDH20,CCR6,CCM2,C1orf174,C18orf63,C10orf143,BSX,AVP,ATN1,ARHGAP33,ARHGAP31,ARHGAP25,ANO4,ADCY1,ACTRT3,ACOT11,ABHD12B
12	ZNF782,ZNF510,ZNF441,ZNF22,ZGRF1,ZBTB32,ZBED4,WWTR1,WSB2,WDR27,VGLL2,UNC79,UNC13C,U2AF1L4,TWIST1,TUSC3,TTI2,TRGV11,TRGJP1,TPCN2,TMEM30A,TMEM247,TINCR,TCOF1,TACSTD2,STAC,SLFN12L,SLC66A1,SLC36A2,SGSM1,SERPINI1,SCG3,SAFB2,RTN4R,RPL3L,RNF17,RNF149,PTPDC1,PSMA2,PSENEN,PRTFDC1,PROSER3,PRMT8,PRKCZ,PRKCE,PPIG,POP4,PLBD2,PHOSPHO2,PHC3,PDE11A,PCYOX1L,OTOS,OR2H2,NUTM2G,NUP58,NLRP4,NDUFA12,MTO1,MS4A18,MRPL55,MRPL35,MRPL32,MMP10,MMP1,MCUB,MAK16,LYPD6B,LRIT3,LRCOL1,LPCAT2,LIN37,LEXM,LDLRAD4,LDB2,LARP7,KMT2B,KLHL41,KISS1R,KHDC3L,KCNQ4,KCNK5,ITPR3,ITGA11,IL5RA,IL17B,IGFLR1,IFIT5,IBA57,HSPB6,GUK1,GPR160,GPC6,GNAT3,GJC2,GABBR1,FUT10,FERMT2,FERD3L,FCER1A,FAM120B,EYA2,ERICH5,ERCC6L2,EPRS1,EPB41L3,ENSG00000288715,ENSG00000285733,ENSG00000267120,ENSG00000261732,ENSG00000256646,ENSG00000251569,ENSG00000188223,ELMO1,DPPA5,DLL1,DHRS12,DDX60,CXCR5,CTSV,CRELD2,CRAMP1,COX8C,COQ6,COMMD9,CITED4,CFAP54,CFAP210,CENPJ,C7orf25,C1orf35,BTBD7,BFSP1,BBS5,BACH2,ASCL1,ARF1,ANKRD27,ANKRD11,ALG12,AGAP1,ADA2,ACAP2
11	ZNF491,ZNF385B,ZNF365,ZFYVE26,ZCCHC17,XCR1,WNT3A,WDFY2,VPS53,UQCC2,TRPM3,TRIP11,TPI1,TP53BP2,TOMM40,TNR,TNFSF15,TMCO3,TIPIN,TIAL1,TBRG4,TAFA1,STAT4,STARD3NL,SPTLC1,SPSB2,SPRR2G,SPC25,SNRNP40,SLC13A3,SIDT1,SH2D4A,SFRP4,SARAF,SAMD5,RTKN2,RFX2,RFC5,RDH12,RAI14,PYGO1,PLXDC2,PIGF,PDCD10,PCSK9,PAX7,PACRG,P3R3URF-PIK3R3,OBSCN,NLRP8,NEIL3,NECTIN2,NACAD,MS4A8,MRO,MMP28,MMP2,MLN,MEX3C,MARCHF1,MAPK8IP3,MAP7,MAP2K1,MALRD1,LTF,LRRK2,LRRFIP1,LIPN,LIN52,LELP1,KRTAP5-7,KRT78,KLHL9,KIRREL3,KDSR,JPT2,IP6K3,IGDCC3,IFNA8,IFNA5,IFNA2,IFIT3,IFIT2,ICE1,HSPA5,HMX3,GRPEL2,GKN2,GAS2,GAPVD1,FKBP5,FHOD3,FBH1,EXO1,EPDR1,EP400,ENTPD5,DPP6,DDX6,DCUN1D2,DBX1,CYP7A1,CYP4B1,CYLC2,CRIPT,CRACR2A,CPXM2,COL6A6,COA7,CNTNAP2,CHI3L1,CHDH,CCDC185,CASP6,CAPRIN2,C1orf52,C17orf50,BBOF1,ATP2B4,ARHGEF12,ARG2,ANXA6,ANKRD16,ALDH6A1,ADTRP,ACTL8,ABCB5
10	ZNF440,ZNF439,ZMYND8,XAF1,WNT9A,VTI1B,UTP20,ULK2,UBXN2B,TSPAN1,TRPC7,TRIM44,TRIM42,TRGJP2,TRGJP,TRGJ2,TRGJ1,TRGC2,TRGC1,TRAPPC4,TRAF3IP1,TPST1,TLCD5,TFDP1,TARP,TAAR1,ST8SIA3,ST3GAL5,SNCAIP,SMCO4,SLC37A4,SLC29A1,SLC25A25,SLC15A5,SERINC2,SEC16B,SCN4B,SCN1A,SCAI,RPS25,RPS14,RNF7,RAPGEF4,RABGEF1,RABEPK,PTGES2,PPP6C,PLA2G12A,PIWIL1,PHACTR2,PDE7B,PCDH9,PANX2,P3R3URF,P3H3,OR6C4,OR10J3,OAS3,OAS2,OAS1,NEK11,NAIF1,MYO1B,MYH13,MTX2,MOV10L1,MLNR,MLC1,MAPRE1,LYZL6,LYSMD2,LRRC31,LMAN1,LEMD2,LCP1,KRT76,KCTD7,IFNA6,IFNA13,HHIPL2,GZMH,GCA,G6PC2,FRG2C,FOXR1,FNDC3A,FLRT2,FBLN5,FAM83B,FABP3,ENSG00000284461,ENSG00000249319,EGFLAM,DUSP10,DPY19L3,DMBT1,DLX2,DDX11,DAB1,CYTIP,CRYL1,CRCP,CENATAC,CDH22,CDADC1,CCSER2,CCDC25,CAMK1D,CAB39L,C11orf96,C10orf90,BCL2L14,BCL10,BANK1,ATP8A2,ANKRD44,ALDH3A1,AKNA,ADAMTS16,ACTRT2,AC114494.1
9	ZHX2,YAF2,VSX2,VEPH1,UPK1B,ULK1,UGT3A1,TTC21B,TRPV3,TRGV9,TRGV8,TPST2,TMEM260,TMA16,TKTL2,TDRD9,TAF15,STXBP6,SPATA31E1,SMIM1,SLC6A20,SLC36A1,SLC22A2,SLC17A5,SHISAL2A,SEPSECS,SDS,RTP5,RNF112,RFTN1,RDM1,RDH11,RBM47,RBM44,RASA3,R3HDM4,PRSS54,PRSS37,PRR16,PROX1,PRODH2,PLXNB2,PIGR,PDGFD,PDE4B,PDCD1,PCNX4,ORM2,ORM1,OR9A4,OR9A1P,OR6C76,OR6C75,OR6C70,OR6C68,OR6C65,OR6C2,NUDT16,NOC4L,NEDD1,NCKAP1L,MYOZ3,MYBPH,MMP13,MIX23,MFAP4,MAPK7,MAP3K20,LUC7L,LRRIQ4,LRRC9,LRRC63,LRP8,LMBRD1,KRT79,KLRD1,IPO8,IGSF21,HOXC4,HEATR9,HBZ,HBQ1,HBM,HBA2,HBA1,GINS4,GCM1,GALNT13,GABRB2,FAM24A,FAM240C,FAM234A,FAM184B,FAM162A,FAM107B,EPM2A,ENSG00000287725,ENSG00000258466,ELOVL5,EBF3,DMBX1,DLGAP1,DLG2,DISC1,DHRS7,DGKH,DFFB,DEPP1,DDX50,DDX21,DAPL1,DAD1,CYP4A11,CSNK2A3,CORO2B,CNIH3,CLEC5A,CFAP97D2,CES5A,CCR1,CCL5,CCDC175,CCDC113,CARD19,CAMKK2,CACNG6,C1orf94,C10orf88,BTN3A3,BTN3A2,BTN3A1,BTN2A2,BTG3,BOK,BLM,BCL11B,B4GALT4,ATP1B3,ATP1A2,ARNTL2,ALG10,ADORA1,ADGRV1,ADGRA3,ABCD4
8	ZNF735,ZNF713,ZNF679,ZNF598,ZNF597,ZC2HC1B,ZAP70,YAE1,VSIG10,USP44,USP36,USP24,USF2,TYSND1,TSPAN33,TRMO,TMEM30B,TDRD12,TBL3,TATDN2,TACR2,SYNGR3,SV2C,STAT2,SRPK2,SPINK6,SPINK5,SNX5,SMIM12,SMAD6,SLC7A9,SLC7A2,SLC39A5,SLC35C2,SLC2A8,SLC1A3,SIAH3,SHPRH,SEZ6L,SERPIND1,SEC63,SACM1L,RUNX2,RTN1,RHCG,RGS9BP,RGS5,REEP1,RAB32,PUS1,PSEN2,PLCH1,PKIB,PI4KA,PHLDB2,PHC2,PDE8A,PDE5A,PDCD5,PCED1B,PCDH20,PBK,PAN2,OSTM1,OR10G3,NUDT19,NR3C1,NPW,NOXO1,NMNAT2,NME3,NIPSNAP2,NINJ1,NEUROG2,NEDD4,NCAM2,NCALD,NAV3,NAGK,NADSYN1,NAA60,NAA30,MYCL,MUC19,MSRB2,MRPS34,MRPS17,MPHOSPH10,MLIP,MIA3,MGME1,METAP2,MED21,MCEE,MAPK9,MAPK4,MAGOH,LRRC47,LNPEP,LASP1,KMT2E,KLHL23,KIFBP,KHDC1L,KHDC1,KCTD16,INTS7,INTS6,IL23A,IGSF11,IFNA7,IFNA4,IFNA21,IFNA17,IFNA16,IFNA14,IFNA10,GXYLT1,GRHL2,GNB3,GNB1L,GLS,GFER,FLG,FCAMR,FBXO45,FBXO30,FAT3,FANCF,FAM161B,FABP2,EVA1C,ESCO2,ENSG00000289360,ENSG00000288914,ENSG00000272410,ENSG00000259164,ENSG00000249773,ENSG00000244255,ENSG00000243501,ENSG00000197991,ENSG00000144785,EME2,ELMO2,DTL,DSTN,DMXL2,DLG5,DDX51,DAAM1,CZIB,CYTH1,CS,CPT2,COQ10A,COL6A3,CNTN4,CNPY2,CNOT10,CMAS,CIPC,CHD1L,CFB,CEP89,CDC42BPA,CDC16,CD109,CCDC180,CCDC141,C4orf3,C2,C1orf116,C12orf71,BUD13,BRF1,BCL9L,BARX1,ASTE1,ASGR2,ARHGEF28,AQP12B,AQP12A,APOF,APLF,ANO3,ANKRD52,ALKBH3,AK1,AGFG1,ADRA1A,ADCY10,AC005606.1,ABHD4

Frequency	Genes
7	ZPR1,ZNF79,ZNF678,ZNF334,ZNF322,ZDHHC8,ZDHHC22,WDR86,WDR53,WASHC4,VWA3B,USP2,UPF3A,UBQLN1,UBE2C,TTC23L,TRMT2A,TRGV5,TRGV4,TRGV3,TRGV2,TRGV1,TRDN,TRAC,TRABD2B,TNNC2,TM4SF1,TFDP2,TDRD7,TCP11L2,TCERG1L,TBPL1,TBC1D17,TAGLN3,SYT16,SULT1E1,SULT1C4,STK38L,STK19,STAM,ST8SIA2,SPTAN1,SPINK13,SP8,SNX21,SNRPA1,SNAP47,SNAP29,SMOC2,SMO,SLCO3A1,SLC6A2,SLC39A11,SLC35A5,SLC2A12,SLAMF1,SKIV2L,SIK3,SELENOS,SCGB3A2,SCGB2B2,SBDS,RPL12,RPH3AL,RNF5,RGL4,RBM22,RBM20,RASEF,RANBP1,PRRX2,PROKR1,PRIM2,PPT1,PPP2R2B,POLR3B,PNKP,PLEKHA3,PLA2G2D,PIP4K2C,PIGM,PHF21B,PEX3,PERP,PDGFRB,PALLD,PAK5,ORC5,OR5AN1,OR10J4,OR10J1,OMD,OGN,OAT,NUGGC,NUDT14,NTMT1,NRDC,NOL8,NME4,NLRP11,NIBAN2,NELFE,NDRG4,NAB1,MS4A12,MMP3,MLPH,MKRN1,MFSD5,METAP1D,MDN1,MAG,LSR,LOXHD1,LMNTD1,LIMS1,LAX1,LAMP5,L3HYPDH,KIF5A,KCNIP4,JMJD4,JKAMP,JAG2,IRX6,IQSEC3,IFNGR1,IFNB1,IDNK,IARS1,HSD17B12,HMGN4,HEMGN,HAMP,GRIK3,GPR162,GPR141,GPR135,GPM6A,GOT2,GOLGA1,GLO1,GLE1,GCC2,GAREM1,FUCA2,FOXE1,FOCAD,FECH,FBXO9,FBXO38,ETV6,ETNK1,ENSG00000289503,ENSG00000288646,ENSG00000288529,ENSG00000286135,ENSG00000284695,ENSG00000284686,ENSG00000283536,ENOX1,EGFL8,ECM2,DXO,DTX3,DPH6,DGCR8,DECR2,DCTN4,DAOA,CYP4Z1,CYP4X1,CYP21A2,CRKL,CREBBP,COQ8A,CLDN8,CEP104,CENPP,CDCP1,CD82,CD22,CCR3,CASP8AP2,CAP1,CADPS,C9orf50,C5orf46,C4B,C4A,C19orf12,C10orf120,BTN2A1,BTN1A1,BTLA,BTBD6,ATP1B1,ATG3,ASPN,ASB6,ARSI,ARHGEF25,APPL2,APOC3,APOA5,APOA4,APOA1,ANKRD31,ALDH4A1,AL645922.1,AKT1S1,AKAP13,AKAP12,AJAP1,AGPAT1,AGER,ADAMTSL3,ABTB1,ABT1,ABHD10
6	ZRANB1,ZNF335,ZNF302,ZC3H11A,ZBTB8A,ZBTB38,ZBED6,YIPF5,WFDC3,WDR11,VIPR1,UNC45B,UMAD1,UHRF1BP1L,UBXN7,UBE3D,TXNDC15,TVP23C-CDRT4,TSPYL5,TSPAN8,TRMT61A,TRIM40,TRIM29,TRIM26,TRIM15,TRIM10,TMCO1,TM9SF2,TIMP2,TEX55,TCL1B,TANK,TAF1A,TAAR2,STOX2,STIMATE-MUSTN1,STIMATE,ST6GALNAC4,SPRR2A,SPINT4,SPINK9,SPINK7,SPICE1,SPECC1,SLIT2,SLC8A3,SLC5A8,SLC39A12,SLC36A3,SLC35G3,SLC22A3,SLC12A5,SH2D3C,SFMBT1,SEC61A2,SEC24B,SDK2,SCN2B,RWDD4,RPS6KC1,RPRM,RNASE2,RINT1,RFT1,RD3,RBM45,RAX,PRSS38,POLK,POC5,PLEKHF1,PLAAT1,PCIF1,PCBD2,PBX2,PAXIP1,PARP1,PAMR1,PAK1,P2RX7,OTUD3,OSER1,OR5A1,OR4D6,OR4D10,OR10J5,OLFML2B,OCSTAMP,NRF1,NPY1R,NMUR2,NEB,MYO5C,MVB12B,MUSTN1,MTAP,MRPL3,MOB3B,MMP9,MMP12,MLLT3,MISP,MGLL,MGAM2,MFSD2A,METTL17,MEOX1,MARK3,MARCOL,MAP3K21,MACC1,LTV1,LRRC52,LPAR1,LOXL2,LAMC2,KRTAP5-9,KRTAP5-8,KRTAP5-10,KIAA0895,KDM3A,KCNJ10,JPH2,JAML,ITIH4,ITIH3,ITGAE,ISM2,IL7R,IFNW1,HMHB1,HMGCR,HERC1,HACD4,GPR35,GPR161,GOLIM4,GOLGA7,GNG12,GNAI1,GLI3,GDAP1L1,GCNT4,GABRA6,FXYD4,FRMD1,FPR2,FOXN3,FOXG1,FCRLB,FCRLA,FAM240A,F13A1,EPHB2,ENSG00000289763,ENSG00000289653,ENSG00000288644,ENSG00000285943,ENSG00000272305,ENSG00000254553,ENSG00000251012,ENSG00000243696,DYNC2I2,DTYMK,DPP4,DNTTIP1,DNAH5,DLK1,DHCR7,DERA,DCTN6,DCTN2,DCBLD1,DAPK2,CYP4A22,CTBP2,CREBL2,COL28A1,COL16A1,COBL,CNOT4,CMTM7,CLBA1,CKB,CERT1,CERS3,CEP170B,CELF2,CDRT4,CDKN2AIP,CDCA4,CCRL2,CCR5,CCR2,CCL3,CATSPER3,CASQ1,CAPSL,CAPN13,CAGE1,BRPF3,BANF2,BAK1,AZIN1,ATP13A5,ARPP19,ANKMY1,ANKDD1B,ANGEL2,AKAP11,ADCK1,ADA,ACYP2,ACTBL2,ACCSL,ACCS
5	ZSWIM3,ZSWIM1,ZSCAN2,ZNF829,ZNF80,ZNF599,ZNF568,ZNF487,ZNF420,ZNF181,ZFYVE28,ZFYVE19,ZBTB8B,XRCC4,WWC2,WDR73,WDR38,WDR20,VPS16,USP3,UPK2,UNC5A,UCK2,UBR7,UAP1,TTYH2,TTC7B,TTC16,TRMT5,TRAV1-2,TRAM2,TOR2A,TNFRSF1B,TNFRSF10A,TMTC4,TMOD2,TMEM251,TMEM196,TMEM131,TMEM117,TM4SF19-DYNLT2B,TM4SF19,TLN2,TLCD3A,TIE1,TEX53,TEX48,TESK2,TEKT1,TCF7L2,TBX3,TBC1D16,TASOR2,SYN3,SYCE1L,SULT1C3,STXBP1,STX11,ST8SIA1,ST6GALNAC6,SRGAP3,SPSB3,SPRR2F,SPRR2E,SPRR2B,SPINK14,SPATA25,SP4,SP3,SMPDL3A,SMPD3,SMIM31,SMAP2,SLFN11,SLC51A,SLC38A6,SLC38A11,SLC30A1,SLC29A3,SLC16A13,SLC16A11,SKA1,SIX4,SH3GLB2,SH3BGRL2,SETD6,SEC11A,SCYGR1,SCN2A,SCHIP1,SAR1A,RWDD2A,RPS6KA2,RPS12,RPL39L,RNLS,RNASEK-C17orf49,RNASEK,RLF,RIPOR2,RFX3,RCSD1,RASGRP3,RANBP2,RAB37,R3HCC1,PTX3,PTRH1,PTK7,PTK2B,PRSS57,PRSS41,PROM1,PRMT7,PRDX1,PRDM1,PPP1R9A,PPP1R14D,PPA1,POP1,POMT2,PLTP,PLSCR1,PLCXD2,PLCH2,PLAGL1,PLA2G2E,PIK3R3,PIGN,PGM3,PDE6H,PCYT1A,PCED1A,P2RX4,OVOL2,OR5A2,OR4E2,OR4E1,NUP188,NUBP2,NUAK1,NTM,NMB,NKX6-1,NKX1-2,NGB,NFE2L2,NEURL2,NCAPG,NASP,MTPAP,MRM3,MPZL3,MPC2,MON1B,MOK,MOAP1,MNAT1,MMACHC,MKNK1,MICOS10-NBL1,MICOS10,MGST3,MEGF10,MED6,ME2,ME1,MDGA2,MAST2,MAPK10,MAP4K2,MAP2K7,MAB21L4,LYZL4,LRSAM1,LRRC8E,LRRC1,LRP2,LRIT2,LRIT1,LMF1,LINC00672,LGR6,LECT2,LCORL,KRTAP27-1,KRTAP26-1,KRTAP25-1,KRTAP24-1,KRTAP23-1,KRTAP13-2,KLHL30,KLHL25,KIF27,KCNJ9,ITIH1,ITGB2,IRS2,IRAK2,IQCJ-SCHIP1,IPP,INSR,IMPACT,IL22RA2,IL20,IKBKE,HUNK,HSP90AA1,HS3ST2,HRH4,HNRNPF,HELB,HACD1,GREB1,GPX2,GPR42,GPR19,GPR176,GPR132,GPBP1L1,GON7,GOLGA6L4,GLRX5,GLRX3,GLOD4,GLIS1,GKAP1,GHRL,GEMIN4,GDI2,GALNT17,FZD8,FITM2,FGF9,FFAR3,FBXL22,FAM98A,FAM110A,FABP7,FAAP24,ENSG00000288674,ENSG00000288208,ENSG00000285446,ENSG00000280148,ENSG00000272741,ENSG00000267360,ENSG00000259066,ENSG00000258989,ENSG00000257524,ENSG00000251184,ENSG00000249209,ENSG00000214558,ENPEP,EMC1,ELP3,ELMOD3,EFCAB10,EEF1E1-BLOC1S5,EEF1E1,EDN2,EDDM13,EBF2,DYNLT2B,DYNC1LI1,DUSP5,DUSP28,DUSP23,DSE,DRD3,DPYSL2,DOP1A,DNAJC17,DNAH14,DLX1,DCAF6,DCAF16,CXCL13,CTSA,CPNE8,CPA2,COLCA2,COL15A1,CNTN3,CNOT1,CMTM6,CLIC5,CILK1,CHURC1-FNTB,CHURC1,CFAP157,CDHR1,CD84,CD4,CCL23,CCL20,CCDC17,CCDC163,CCDC138,CBLB,CAPG,CANT1,C5orf49,C5orf15,C17orf49,C10orf67,C10orf105,BTBD17,BTBD11,BICD2,BEGAIN,BCL6B,ATP6V1C1,ATP5PO,ATP2B2,ART4,ARRDC3,ARNT2,ANP32B,ANKRD30A,ANGPT4,ANAPC5,ALOX12,AL109659.1,AKR1A1,AIG1,AHNAK2,AGXT,AFAP1L2,ADAT2,ADAM20,ACTR6,ACOT8
4	ZNF804A,ZNF763,ZNF701,ZNF700,ZNF69,ZNF438,ZEB2,ZDHHC19,ZBTB40,WWC1,WHRN,WBP4,VNN2,VIPR2,UQCRH,ULK3,UBR4,TTC30B,TRMT1L,TRIM35,TREH,TRAV1-1,TRAJ9,TRAJ7,TRAJ6,TRAJ5,TRAJ4,TRAJ3,TRAJ26,TRAJ25,TRAJ24,TRAJ23,TRAJ22,TRAJ21,TRAJ20,TRAJ2,TRAJ19,TRAJ18,TRAJ17,TRAJ16,TRAJ14,TRAJ13,TRAJ12,TRAJ11,TRAJ10,TRAJ1,TRAF6,TRAF5,TPCN1,TMEM69,TMEM26,TMEM209,TMEM183A,TMEM167A,TMEM127,TMEM120B,TMED8,TLE7,TINAG,TIGIT,THEM5,TEX38,TENT5A,TECPR2,TCF7L1,TBC1D5,TARBP1,TAGLN,TAB1,SZT2,SYT2,SYNGR1,SYNE3,SWT1,SUSD2,SULT1C2,STRN3,STRAP,STIL,STARD7,SSMEM1,SRPK1,SRGAP2,SQOR,SPRR4,SPRR3,SPRR2D,SPRR1B,SPRR1A,SPIRE1,SPATA5,SOX8,SNX19,SNRNP200,SMCO3,SLC8B1,SLC38A7,SLC35F1,SLC2A9,SLC26A8,SLC25A36,SLC18B1,SLC15A1,SKA3,SIM1,SIDT2,SERPINA6,SERPINA2,SERPINA1,SEMA6D,SEC61B,SEC13,SCYL2,SCGB2A2,SCGB1D4,SCGB1A1,SCAMP2,RSU1,RPP25,RPL35,RNPEPL1,RNF2,RNF166,RNASE3,RMDN2,RIN2,RIMS3,RIMS2,RIMOC1,RHOJ,RHOH,RECQL,RALA,RAD54L,RABIF,QSOX2,PYROXD1,PURB,PTPRA,PRR5L,PRIMA1,PPP1R2,PPM1A,PPIA,PPARD,PON3,PON2,PON1,POMGNT1,PLXNC1,PLEKHG4B,PLD4,PIK3R4,PHLDB1,PHGR1,PGLYRP4,PGLYRP3,PEF1,PCSK7,PCSK5,PATJ,PAQR8,PAFAH1B2,OXCT1,OSBPL1A,ORC4,ORAI1,OR8S1,OR5BS1P,OR13A1,OR10G2,ODF3L2,OASL,NUDT5,NSUN4,NLRP13,NFE2,NFATC1,NEURL3,NCAPH,NBL1,N4BP2,MYCBP2,MTHFD1L,MTARC1,MRTO4,MRPS26,MRPL57,MRPL2,MPL,MPI,MORN3,MOB3C,MIXL1,MFSD4A,METTL8,MED8,MAPK14,MAP3K7,LURAP1,LTA4H,LRRC41,LRP1B,LORICRIN,LIN9,LHX4,LHX3,LHFPL5,LCE5A,LCA5,LBR,LALBA,KRT8,KRT39,KNSTRN,KLHL14,KLHL12,KLC4,KIF19,KDM2B,JAZF1,IVNS1ABP,IVD,ITPRIPL1,ITPKB,IQCF5,IQCF3,IQCF2,IPPK,IL9,IL24,IL17REL,IL17A,IFTAP,HYI,HNRNPA1,HEATR5A,HCRTR1,HCN2,HAS2,HAL,H2AZ2,GSTA4,GRIP1,GRIK2,GRAMD2B,GPX7,GPR26,GPC5,GOPC,GOLM1,GNRH2,GNB5,GALNTL6,GALNT3,GALNS,FXYD7,FXYD1,FSTL4,FMOD,FLT1,FGF2,FFAR1,FCMR,FBXO4,FAM219B,FAM177B,FAAH,ESR1,ERAP1,ENSG00000288716,ENSG00000288684,ENSG00000288614,ENSG00000288564,ENSG00000286231,ENSG00000285749,ENSG00000283228,ENSG00000267699,ENSG00000267179,ENSG00000249624,ENAH,ELOVL1,ELF1,ELAC1,EIF2AK4,EFHC1,EFCAB14,EEPD1,EDIL3,EDEM3,DYNC1I1,DYNC1H1,DOCK1,DMRTB1,DISP2,DHTKD1,DEPDC4,DENND5B,DEF6,DDX58,DCUN1D5,DAZL,DAW1,CYP4F8,CUL7,CRCT1,CPA4,COX5A,COPZ1,COL3A1,COBLL1,CMPK1,CLPSL1,CLIP1,CIAO1,CEP295NL,CDC20,CD9,CCSAP,CCL15-CCL14,CCL15,CCL14,CCDC88C,CCDC149,CBX5,CASZ1,CAPN15,CACNB4,CABIN1,C1orf87,C12orf60,C12orf54,BTBD9,BROX,BPIFA3,BOD1L2,BIRC3,BCL2L10,BAALC,B4GALT5,ATPAF1,ATP8B1,ATP6V1G1,ASRGL1,ASCC3,ARPC5L,ARMC12,ARL5A,ARHGDIB,ARFGEF3,APCS,AP5S1,ANP32D,ANGPT2,AMER2,ALG2,ALG10B,AKR7L,AKR7A3,AKR7A2,AK4P3,AIDA,AHRR,AGTR1,AGPS,ACKR3,AAK1
3	ZNHIT6,ZNF850,ZNF839,ZNF790,ZNF777,ZNF76,ZNF662,ZNF618,ZNF567,ZNF529,ZNF461,ZNF410,ZNF382,ZNF367,ZNF345,ZER1,ZDHHC12,ZC3H11B,YBX3,XPO7,WEE2,WDR7,VDR,VDAC1,VAT1L,VANGL2,UST,UHMK1,TXNDC16,TTC39A,TTC33,TTC30A,TTC3,TSTD3,TSPEAR,TSC22D2,TRPV1,TRIML2,TRIML1,TRIM9,TRIM66,TRAPPC2L,TPD52L1,TNIP1,TNFAIP8,TNFAIP3,TMPPE,TMOD3,TMEM82,TMEM163,TMEM123,TMEM106C,TKT,THTPA,TGFBI,TFRC,TCF4,TBRG1,TBC1D8,TBC1D13,TAX1BP1,TATDN3,TAS2R5,TAS2R4,TAS2R38,TAS2R3,TAL1,TAF3,SYNE1,SVIP,SUMF2,SUMF1,STXBP5,STX18,STK32C,STAB1,SSBP1,SRGN,SPX,SPRR5,SPOUT1,SPNS3,SPATA6,SPATA45,SOX5,SNX30,SNRPB2,SNAI3,SMIM29,SMIM21,SMCO1,SMARCB1,SMAD4,SLX4IP,SLITRK1,SLCO1B1,SLC7A10,SLC6A3,SLC5A7,SLC5A12,SLC47A2,SLC35D2,SLC2A6,SLC2A13,SLC2A1,SLC25A34,SLC25A15,SLC19A2,SLC17A3,SLC17A2,SLC17A1,SKOR1,SIGLEC8,SET,SERPINI2,SERPINA3,SERPINA10,SEPTIN9,SDHA,SDE2,SDC3,SDC2,SCUBE3,SCGB2A1

Frequency	Genes
	,SCGB1D2,SCGB1D1,SCARA3,SAP18,RSL24D1,RSC1A1,RRP9,RRP36,RPUSD2,RPS24,RPS2,RPL37,RPL36AL,RPL29,RPAP3,RNF34,RNF168,RNF151,RNASE1,RMDN3,RGS7,RGS6,RGL1,RFX4,RESF1,RCOR3,RASSF2,RAPGEF3,RAG2,RAG1,RAD51,RAB4A,QTRT2,PTPRB,PTGR2,PTGER4,PTF1A,PSPH,PSMG2,PRTG,PRNP,PRKAA1,PRDM4,PRDM13,PPP4R4,PPP2R5D,POLR3A,POLE2,PLEKHM2,PLEKHJ1,PLCXD3,PKN3,PIWIL4,PHYHD1,PHKG1,PHF5A,PEX7,PEX6,PDCD6-AHRR,PDCD6,PCBP4,PARP3,PANX3,PABPN1L,NUPR2,NUDT6,NTNG1,NTN4,NSL1,NR3C2,NPM2,NNAT,NME7,NLGN1,NISCH,NDUFB10,NCS1,NCOA5,NCF2,NAF1,MXI1,MUC13,MTRF1,MRPS31,MRE11,MPC1,MMP11,MLLT1,MIS18BP1,MIOS,MIGA2,MICU3,MGAT2,MEIOB,MEA1,MBD1,MAPK6,MAPK13,MAP2K5,MAML2,LZTFL1,LYZL1,LSM14A,LRRC8A,LRRC14B,LRR1,LRP3,LPIN1,LIPK,LHFPL4,LGI4,LGALS3BP,LEO1,LDLRAP1,LBHD2,LARP1,LAPTM4B,KYAT1,KRTAP5-11,KRT80,KRBOX1,KNL1,KMT2A,KLHDC3,KIN,KIF11,KCTD9,KCNMA1,JRKL,JPH4,JDP2,ISG20L2,IQCH,IQCD,INIP,IL1RAP,IDE,HTT,HSD17B4,HOOK1,HMGA1,HLX,HIBADH,HHEX,HFM1,HASPIN,HAGH,HABP4,GULP1,GSTZ1,GSAP,GRB14,GPR83,GPR62,GPR137C,GPC1,GOLT1B,GNRH1,GNMT,GLRA1,GFPT2,GFOD1,GCHFR,GBA3,GASK1A,GADD45G,G3BP1,FYCO1,FXYD5,FXYD3,FOXO1,FLVCR1,FLG2,FGF17,FGD4,FBXO39,FARS2,FANCM,FANCC,FAHD1,EXOC3L4,ERP27,ERCC2,ENSG00000289722,ENSG00000289025,ENSG00000286112,ENSG00000286001,ENSG00000285827,ENSG00000285526,ENSG00000285269,ENSG00000273291,ENSG00000258653,ENSG00000257065,ENDOU,ENDOG,EHD3,EGR3,DOT1L,DOLK,DOK2,DOCK5,DOCK10,DNAJC16,DNAI2,DNAH9,DNAAF2,DLC1,DISP1,DIPK1C,DERL3,DDI2,DCP1A,DCAF17,DAP,CYP2J2,CYBRD1,CYB5R1,CXXC1,CXCR6,CRNN,CR1L,COTL1,COLEC12,COL6A5,COL2A1,COG2,CNPY3,CLPS,CINP,CHSY3,CHSY1,CHST8,CHST14,CHCHD2,CFAP53,CFAP107,CEP76,CENPF,CDK18,CD34,CD163L1,CCT6A,CCR9,CCNC,CCL18,CCL16,CCDC82,CCDC32,CCDC191,CCDC181,CCDC177,CCDC127,CBFA2T3,CASP9,CARD6,CACNG8,CACFD1,C6,C3orf33,C15orf62,BTG2,BTBD2,BSG,BPIFA2,BLZF1,BLCAP,BIRC2,BICD1,BHLHE41,BCLAF1,BCAT1,BATF3,BAHD1,B3GALT5,ATXN7L3B,ATP2A3,ATP12A,ATOX1,ASCL4,ASB1,ARPC5,ARHGEF7,ARHGAP10,APOBEC4,AP4S1,AP1G2,ANKS6,ANKRD50,AMDHD1,ALK,AKAP6,AKAIN1,AGPAT4,AGMAT,AFAP1L1,ADRB2,ADIPOR1,ADGRB3,ADCY9,ADAMTS5,ADAMTS13,ADAM12,ACY1,ACP3,ACO2,ABHD14B,ABHD14A-ACY1,ABHD14A,ABCC4,AADACL3
2	ZNF865,ZNF827,ZNF746,ZNF616,ZNF615,ZNF37A,ZNF33A,ZNF217,ZNF189,ZMIZ2,ZFYVE21,ZFHX2,ZDHHC20,ZC3H12A,ZBTB41,ZBTB22,ZBTB2,ZBBX,XRCC3,XPR1,XNDC1N-ZNF705EP-ALG1L9P,XDH,WIPF3,WDR89,WDR46,VWF,VWA8,VPS52,VIPAS39,VAV1,VARS1,USP45,URM1,URGCP-MRPS24,URGCP,URB1,UCHL5,UBOX5,UBE2T,UBE2D4,UBAP1L,U2SURP,TXNRD2,TXNL1,TXLNB,TUFT1,TTC36,TTBK1,TSPAN31,TSPAN17,TSFM,TSEN2,TRUB2,TRPC4,TRMT44,TRIM38,TRAV39,TRAF3IP2,TPH2,TNS4,TNKS1BP1,TNFRSF11A,TMEM63C,TMEM275,TMEM25,TMEM192,TMEM179,TMEM121,TMEFF2,TMCC3,TLR5,TIAM2,TIA1,THUMPD3,TGFB2,TGFA,TFEB,TEX9,TESPA1,TEDC1,TDRKH,TCERG1,TBX15,TBX1,TASP1,TAPBP,SWI5,STK33,STIM2,STAT6,ST8SIA5,ST18,SSRP1,SRSF6,SPRTN,SPNS1,SPG7,SPARC,SOCS4,SNX27,SNRPG,SNIP1,SMIM41,SMIM40,SMIM34,SMIM11,SMC3,SMC2,SMARCC2,SLCO1B3-SLCO1B7,SLCO1B3,SLCO1A2,SLC9C1,SLC6A7,SLC6A6,SLC5A4,SLC51B,SLC45A3,SLC36A4,SLC35B3,SLC27A4,SLC25A29,SLC25A21,SLC22A5,SLC22A4,SLC22A18,SLC1A2,SLC16A10,SLC11A1,SKOR2,SKI,SIX6,SIX1,SIGMAR1,SHPK,SH3GL3,SH2D6,SGPP1,SFTPC,SF3B1,SF3A2,SETD5,SERPINB6,SEPTIN14,SENP5,SEMA4D,SEMA3A,SEL1L2,SDHAF4,SCYL3,SCYGR4,SCYGR3,SCYGR2,SCYGR10,SCN8A,SCN3A,SCGB3A1,SCG2,SAMD15,RTCB,RSPO1,RPS6KA5,RPS29,RPS18,RPP25L,RPL13,RPA3,RORC,RNF41,RNF26,RNF11,RNASE12,RNASE11,RMND1,RIIAD1,RIC8B,RHEX,RGS3,RGS13,RGS1,RGR,RGL2,RFX8,RFX7,RFTN2,RFPL3,RFPL2,REV3L,REPS1,RELCH,RD3L,RASSF9,RASL12,RAP1GAP2,RABEP2,RAB6C,RAB3IP,RAB27A,RAB19,PWP1,PTPRK,PTPRG,PTPA,PROSER1,PRND,PRLR,PRKCD,PRELP,PRELID2,PPP1R13B,PPP1R12B,POPDC3,POLR1F,PLSCR4,PLD5P1,PLCB4,PLBD1,PLA2G4A,PKD1L1,PIP5K1C,PIGZ,PIEZO1,PHYHIP,PHLDA2,PGR,PGAP6,PGAP1,PFDN6,PEX14,PEX10,PELI2,PEBP4,PDZD3,PDHA2,PDE3A,PDE1A,PCYOX1,PCGF3,PCDH18,PBOV1,PARP16,PAH,P4HA2,P2RX5-TAX1BP3,P2RX5,P2RX3,OXT,OTUB2,OS9,OR7E24,OR6C6,OR6C3,OR5V1,OR5AK3P,OR5AK2,OR13D1,OR13C9,OR13C8,OR13C5,OR13C2,OR12D3,OPTC,OPN4,OLFML2A,OAZ3,NXPH1,NUS1,NTS,NTPCR,NSG1,NRG3,NR4A1,NR1H4,NPS,NPBWR1,NOXRED1,NOS1AP,NOL10,NLRX1,NIPSNAP3B,NIPSNAP3A,NIN,NHLRC3,NFYC,NFU1,NFATC2IP,NEMF,NDUFAF5,NDFIP2,NCSTN,NCBP2AS2,NCBP2,NABP2,MYRFL,MYO5A,MYLK,MYL3,MYH8,MYH4,MYH2,MYH1,MTFMT,MTA1,MSRB1,MRPS6,MRPS24,MRPL9,MRPL28,MRPL22,MRGPRG,MPZL1,MORC3,MOB4,MNS1,MIPOL1,MIF,MICU2,MGP,MGAM,MFSD4B,MFSD1,METTL1,MED12L,MEAF6,ME3,MAST4,MARK1,MARCHF9,MAPK8,MAPK1IP1L,MAP4K3,MAN1B1,MAGI1,LYN,LTBP1,LSM2,LRRK1,LRRC55,LRP5,LPP,LIX1,LIPJ,LIPF,LINGO4,LINC00696,LHFPL6,LDB3,LAT,L3MBTL1,L1TD1,KRTDAP,KRTAP12-4,KRTAP12-3,KRTAP12-2,KRTAP12-1,KRTAP10-9,KRTAP10-11,KRTAP10-10,KRT18,KLHDC2,KLHDC1,KLF6,KLF12,KLC1,KIFAP3,KIF1A,KIAA1614,KIAA0586,KDM4C,KCTD3,KCNE2,KBTBD13,JSRP1,JCAD,JAGN1,IVL,ITPR1,ITIH2,IRF2,IRF1,IQCF1,IMPDH1,IL11RA,IL10RB,IGSF9,IGLL1,IFNAR2,IFNAR1,IFI27,IER5L,IBTK,HSPE1-MOB4,HSPE1,HSPD1,HSPB8,HSPA13,HSD17B7,HS3ST6,HOXA9,HOXA7,HOXA6,HOXA5,HOXA4,HOXA3,HOXA2,HNRNPA3,HIST1H4B,HINFP,HILPDA,HIF1A,HHIP,HFE,HERPUD1,H4C1,H3C3,H3C2,H3C10,H3C1,H3-3A,H2BC3,H2BC13,H2AC4,H2AC14,H2AC13,H1-2,H1-1,GYS2,GUCY2C,GTF3C4,GTF3C3,GSTT2B,GSTT2,GRM4,GRIN1,GRAMD1C,GPR151,GPR107,GPHB5,GP2,GORAB,GOLGA5,GNL2,GLYATL2,GLYATL1,GLYAT,GFPT1,GEMIN5,GCSAM,GCNT2,GAS2L3,GALT,GAK,FSHB,FOXD1,FNBP1,FLRT3,FLJ00388,FIGLA,FGF20,FER,FEM1B,FCER2,FBXO42,FBN2,FAXDC2,FAM219A,FAM184A,FAM136A,FAM135A,FAF1,F13B,EXT2,EXOC8,EXOC1L,EXOC1,EVX1,EVL,ESF1,ERC2,EPHA7,EPAS1,ENSG00000289697,ENSG00000289258,ENSG00000288520,ENSG00000288000,ENSG00000287908,ENSG00000287856,ENSG00000285762,ENSG00000285064,ENSG00000262304,ENSG00000259171,ENSG00000259060,ENSG00000258728,ENSG00000258052,ENSG00000257921,ENSG00000257341,ENSG00000257184,ENSG00000257062,ENSG00000256500,ENSG00000251357,ENSG00000235007,ENSG00000203546,ENSG00000187186,ENHO,ENDOD1,ELOVL4,ELK4,ELAVL4,EIF5,EIF4E1B,EGLN1,EFEMP1,EEF1AKMT3,EDNRB,ECT2L,ECHDC3,E2F7,DUSP7,DTWD2,DTD2,DRD5,DPYS,DPP7,DPAGT1,DOLPP1,DOCK9,DNPH1,DNALI1,DNAI1,DMRTA2,DMKN,DEUP1,DENND2B,DEGS2,DEFB108B,DDX31,DDTL,DDT,DDR2,DCTN3,DCC,DAXX,DAB2IP,CYP7B1,CYP27B1,CYP1B1,CUL9,CUBN,CTSE,CTNS,CTDSP2,CRY1,CRP,CRIP2,CRIP1,CRAT,CRADD,CRACDL,CRACD,CPLANE2,CPA5,CPA1,COQ4,COQ10B,COPS8,COLQ,COL4A1,COL20A1,COA8,CNTN6,CNTN1,CNTLN,CNOT8,CNOT6,CNKSR3,CNGB3,CNGA3,CLU,CLN6,CLDN24,CLDN22,CIDEC,CHRM2,CHMP7,CHIT1,CHD2,CHAF1B,CFHR5,CFHR3,CFHR2,CFHR1,CFH,CEP83,CEP41,CEP350,CEP164,CELF3,CELA2B,CDX1,CDT1,CDKN2C,CDK4,CDH13,CDC73,CDC7,CD6,CD53,CD46,CD300LF,CD19,CCNL1,CCNB1IP1,CCL27,CCL19,CCDC83,CCDC68,CCDC197,CCDC179,CCDC170,CCDC153,CCDC150,CCDC146,CBL,CATSPERB,CAST,CAPNS2,CABCOCO1,C9orf43,C5orf63,C4BPB,C4BPA,C3orf52,C3,C2orf66,C2CD5,C2CD4D,C2CD2L,C22orf42,C21orf140,C1QTNF5,C1orf185,C1orf134,C14orf39,C14orf180,C10orf99,BVES,BTD,BMP1,BMF,BEND4,BBOX1,BAZ1A,BAG5,BAAT,B4GALT6,B4GALNT1,B3GALT4,B3GALT2,AXIN1,AVIL,ATRN,ATP6V1C2,ATP5MJ,ATP23,ATG101,ASPM,ASPG,ASPA,ARPIN-AP3S2,ARL14EP,ARID3C,ARHGEF19,ARHGAP22,ARFGAP1,ARF6,AQP3,APRT,APOLD1,APCDD1L,AP3S2,AP3D1,AOPEP,ANXA4,ANKRD9,ANKRD66,ANKRD34C,ANKRD28,ANG,AMIGO2,AMH,ALX4,ALDH1A3,AL589765.1,AKR1C8,AHSA1,AHNAK,AGMO,AGAP2,ADGRA1,ADD2,ADAMTSL1,ACOX3,ACBD6,ABI3BP,ABCG4,ABCA1,AAGAB,AADACL4
1	ZW10,ZSCAN9,ZSCAN31,ZSCAN26,ZSCAN23,ZSCAN16,ZSCAN12,ZP4,ZNF83,ZNF814,ZNF670-ZNF695,ZNF670,ZNF669,ZNF649,ZNF613,ZNF609,ZNF582,ZNF579,ZNF577,ZNF536,ZNF534,ZNF524,ZNF521,ZNF518B,ZNF518A,ZNF507,ZNF485,ZNF418,ZNF417,ZNF391,ZNF350,ZNF32,ZNF318,ZNF30,ZNF256,ZNF239,ZNF236,ZNF219,ZNF184,ZNF165,ZNF133,ZNF124,ZKSCAN8P1,ZKSCAN8,ZKSCAN4,ZKSCAN3,ZKSCAN2,ZHX3,ZDHHC23,ZDHHC14,ZCCHC2,ZC3H12C,ZC3H10,ZBTB9,ZBTB39,ZBTB20,ZBTB12,ZBED9,XRN1,XPO6,XPO4,XPNPEP1,XNDC1N,XKR3,WTIP,WRAP53,WNT5B,WLS,WFIKKN1,WDR90,WDR88,WDR37,WDR25,WDR24,WDR1,WASF3,WAPL,VWCE,VWA7,VSTM2A,VPS37A,VPS18,VPS13D,VPS11,VIM,VHL,VAPB,VAMP8,VAMP5,USP8,USP50,USP28,USP10,USF3,URI1,UPP2,UPF2,UNC5C,UNC119B,UHRF1,UGT1A9,UGT1A8,UGT1A7,UGT1A6,UGT1A5,UGT1A4,UGT1A3,UGT1A10,UGT1A1,UBR2,UBE2V1,UBE2R2,UBAP2,UBAP1,UBAC2,UBAC1,UBA2,UAP1L1,TYMP,TXNDC9,TWF1,TUFM,TUBB,TTLL8,TTK,TTC6,TTC5,TTC27,TSPYL4,TSPYL1,TSPAN9,TSPAN15,TSNAX-DISC1,TSGA10,TSC1,TRPM8,TRPM7,TRPM5,TRPC1,TRNT1,TRIP4,TRIAP1,TRHDE,TREML4,TRDV3,TRDJ4,TRDJ3,TRDJ2,TRDJ1,TRDD3,TRDC,TRBV20OR9-2,TRAV9-2,TRAV8-6,TRAV2,TRAV14DV4,TRAV12-3,TRAPPC3L,TPTE2,TPM1,TPBG,TP53,TOX2,TOP1,TOMT,TOMM20L,TOGARAM2,TOGARAM1,TNS1,TNRC6C,TNIP3,TNIK,TNFSF14,TNFRSF14,TNFAIP8L3,TNFAIP2,TMPRSS7,TMPRSS3,TMPRSS2,TMPRSS15,TMIE,TMEM53,TMEM40,TMEM255B,TMEM253,TMEM250,TMEM242,TMEM239,TMEM233,TMEM216,TMEM178B,TMEM178A,TMEM138,TMEM132B,TMEM126B,TMEM126A,TMEM125,TMEM108,TKFC,TIMP3,TIMM9,THUMPD2,THRAP3,THBS2,THBS1,TGM6,TGFBR1,TEX49,TEX264,TESMIN,TERT,TDRD6,TCP11,TCN1,TCF21,TBP,TBC1D22A,TASOR,TANGO2,TAFA5,TAC3,SYTL2,SYT9,SYNJ2BP-COX16,SYNJ2BP,SYNGAP1,SYCE3,SVIL,SUSD6,SUSD5,SURF6,SURF4,SURF2,SURF1,SULT6B1,STYK1,STX3,STX2,STUB1,STRA6,STPG2,STON2,STOML3,STMP1,STKLD1,STK40,STK32B,STK17A,STIM1,ST8SIA6,ST3GAL4,SRSF9,SRSF5,SRP54,SRGAP1,SRF,SRD5A3,SPTB,SPRY1,SPPL3,SPPL2A,SPOPL,SPNS2,SPINT1,SPG21,SPEF2,SPEF1,SPECC1L-ADORA2A,SPECC1L,SPDYE1,SPATA46,SPATA22,SPATA17,SPATA12,SPACA9,SOX18,SORCS2,SORBS1,SOGA3,SOCS5,SOCS3,

Frequency	Genes
	SNX22,SNX13,SNX10,SNX1,SNW1,SNAP25,SMURF1,SMNDC1,SMIM32,SMG7,SMCO2,SMARCD1,SMAD5,SLIT3,SLIRP,SLC6A19,SLC6A18,SLC5A9,SLC5A1,SLC49A4,SLC44A4,SLC43A2,SLC41A1,SLC39A8,SLC39A7,SLC38A9,SLC37A3,SLC37A1,SLC35E3,SLC35D3,SLC35C1,SLC26A9,SLC25A35,SLC25A27,SLC25A12,SLC22A7,SLC1A4,SLC19A3,SLC16A7,SLC13A5,SLC13A4,SLC12A6,SLC10A1,SLAMF8,SLAIN1,SKIDA1,SIM2,SIGLEC11,SHOX2,SHC3,SH3D21,SH2B1,SGPP2,SGCA,SFXN5,SFXN1,SFMBT2,SETD7,SESN3,SERPINB12,SERINC1,SEMA7A,SEMA4F,SECISBP2,SEC61G,SEC24A,SEC16A,SDHAF3,SDHAF2,SCTR,SCP2D1,SCO2,SCN3B,SCN10A,SCLT1,SCARF1,SCAMP5,SBF1,SAV1,SARNP,SAPCD2,SAMM50,SAMD14,SALL3,SALL2,S1PR4,RYR2,RXYLT1,RXRG,RXRB,RUFY4,RUBCNL,RTL10,RTL1,RSRC1,RSPO3,RRP1B,RPS8,RPS27L,RPS10-NUDT3,RPS10,RPL7A,RPL41,RPL38,RPL31,RPL27A,RPL26L1,RPL10L,RPGRIP1L,ROPN1,ROBO4,ROBO3,RO60,RNH1,RNGTT,RNF220,RNF214,RNF181,RNF121,RNF113B,RNF10,RNASE9,RITA1,RIPOR3,RIOK2,RING1,RIMKLB,RILP,RIDA,RHOV,RHOT2,RHOG,RHBDL1,RGS21,RGS18,RGS17,RGMA,RFLNA,REXO4,REV1,RELB,RDX,RDH5,RDH10,RBPMS2,RBP4,RBM26,RBM15B,RASSF7,RASSF5,RASAL1,RARRES1,RAP1B,RANGRF,RAF1,RAD54L2,RACGAP1,RABL6,RAB8B,RAB40C,RAB3GAP2,RAB36,RAB31,RAB29,RAB27B,RAB20,RAB15,RAB11FIP3,R3HDML,QSOX1,QSER1,QPCT,PXT1,PXDN,PWWP2B,PUS7L,PUM1,PTTG1IP,PTPRR,PTPRM,PTPRH,PTPN6,PTPN1,PTH1R,PTGIS,PTDSS2,PTCHD4,PTBP1,PSMD1,PSMB1,PSMA6,PSAT1,PRXL2B,PRSS50,PRSS3,PRSS23,PRSS16,PRSS12,PRRT4,PRRT3,PRRG4,PRR5-ARHGAP8,PRR35,PRR3,PRPF8,PRPF39,PRORP,PROC,PRKD3,PRDM6,PRB3,PPP6R3,PPP6R2,PPP5C,PPP4R3B,PPP4R1,PPP2R3C,PPP1R9B,PPP1R32,PPP1R18,PPP1R10,PPM1L,PPIL1,PPIB,PPCDC,POU6F2,POU4F2,POP5,POM121L2,POLRMT,POLR3G,POLR3D,POLG,PNPT1,PNPLA3,PNP,PNKD,PNISR,PMEL,PM20D1,PLXNA4,PLS1,PLPPR5,PLPPR3,PLOD1,PLEKHD1,PLEK,PLCL1,PLCG1,PLCB1,PLB1,PLA2G7,PKHD1,PIWIL2,PIK3AP1,PIGQ,PIF1,PICALM,PIBF1,PHRF1,PHPT1,PHOX2A,PHIP,PHF10,PHF1,PGM1,PGBD5,PGBD1,PGAP2,PGA5,PGA4,PGA3,PEX19,PEPD,PEDS1-UBE2V1,PEDS1,PEA15,PDXP,PDXK,PDIA6,PDE6A,PDE1B,PDCD2L,PDCD2,PCOLCE2,PCLAF,PCDH1,PCARE,PAX3,PATL1,PARVB,PARP2,PAQR9,PALD1,PACS2,PA2G4,P3H2,P2RY14,OTULINL,OTUD4,OSBPL8,OSBPL3,OSBPL11,OSBP2,OSBP,ORMDL2,OR6Q1,OR6C74,OR5C1,OR5AU1,OR52M1,OR4D9,OR4D11,OR3A3,OR3A1,OR2B8P,OR2B6,OR2B2,OR1R1P,OR1L6,OR1L4,OR1L3,OR1E2,OR1E1,OR10V1,ODF3B,ODF2,OAZ2,NUP35,NUP205,NUP107,NUMA1,NUDT3,NUDT2,NUCKS1,NUBPL,NUAK2,NT5DC1,NRROS,NRP2,NRM,NRG1,NR2C1,NPY5R,NPEPPS,NPDC1,NOM1,NKAPL,NHLRC4,NHLH1,NFKB1,NETO1,NEMP1,NEK4,NDUFAF7,NDUFA10,NDST3,NDRG2,NCL,NCAPH2,NCAM1,NBPF3,NAB2,NAA35,MYT1L,MYSM1,MYORG,MYO5B,MYO1A,MYO10,MYL6B,MYL6,MYL10,MVD,MTUS1,MTRR,MTNR1B,MTMR7,MTIF3,MTDH,MTCL1,MSX1,MSI1,MRS2,MRPS18B,MRPL50,MRPL46,MRPL30,MRPL21,MRPL16,MRGPRE,MRAP,MPPED2,MPHOSPH8,MNX1,MMP21,MMP19,MMEL1,MLLT10,MLF1,MLEC,MKRN3,MKRN2,MKKS,MKI67,MITD1,MIOX,MGAT4C,MGAT4A,MFAP5,METTL7B,METTL6,METTL2B,METTL26,METRN,MERTK,MEP1B,MEP1A,MED28,MED22,MED10,MDM2,MDFIC2,MDFI,MDC1,MCRIP2,MCM9,MCAM,MBP,MBOAT1,MAS1L,MARS2,MAPKAPK3,MAPKAPK2,MAPK8IP2,MAPK8IP1,MAP7D1,MAP4K5,MAP3K19,MANF,MANBA,MAMDC4,MAGOHB,MAF,MAD2L1,M1AP,LZTS3,LYZL2,LYSMD3,LYG2,LYG1,LXN,LSAMP,LRTOMT,LRRTM1,LRRC58,LRRC56,LRRC51,LRRC10,LRFN2,LPIN3,LPGAT1,LPAR3,LONRF2,LNPK,LMNTD2,LMF2,LMAN1L,LIPT1,LHX9,LGI2,LGALS3,LGALS1,LCN9,LARS2,LAP3,LAMTOR1,LACTB,LACRT,KRTAP10-5,KRTAP10-4,KRTAP10-12,KRT19,KRT15,KRBA1,KNCN,KLK14,KLHL3,KLHL28,KLHDC8A,KLHDC7B,KLF5,KLF14,KIRREL1,KIFC1,KIF6,KIF4B,KIF2C,KIF24,KIAA2013,KIAA1109,KIAA0408,KDM6B,KCTD15,KCNMB4,KCNK2,KCNJ6,KCNH5,KCNF1,KCNE4,KCNE1,KCNAB1,KATNBL1,KANSL2,KAAG1,JUN,JMJD8,JADE1,ITIH5,ITGB1,ITGA7,ITGA1,ISLR,IRAK4,IRAK1BP1,IQCF6,IQCA1,INPP5A,INO80,INHBB,ILKAP,IL31RA,IL21,IL2,IL18BP,IL17RD,IL17D,IKZF3,IKZF2,IGHMBP2,IGFL1,IGFALS,IFT74,IFT43,IFI27L2,IFI27L1,IER3,IARS2,HYOU1,HTR6,HTR3B,HTR3A,HTR2B,HSPBAP1,HSPA1L,HSPA1B,HSPA1A,HSF2BP,HSF2,HSD17B8,HS3ST3B1,HRAS,HPGD,HPCAL1,HOXD9,HOXD13,HOXD12,HOXD11,HOXD10,HOXA1,HMGCLL1,HMGB2,HMCN1,HMBS,HLA-E,HLA-DRB5,HK2,HIBCH,HGD,HESX1,HEMK1,HECA,HDGFL1,HDDC2,HDAC11,HAT1,HACL1,H4C9,H4C13,H4C12,H4C11,H3C12,H3C11,H2BC17,H2BC15,H2BC14,H2BC12L,H2BC12,H2BC11,H2AX,H2AC17,H2AC16,H2AC15,H2AC12,H2AC11,H1-5,GYS1,GYPC,GTSF1,GTF3A,GSG1L2,GSC,GRXCR2,GRP,GRM2,GRK1,GRHL1,GPX6,GPX5,GPX3,GPRC5C,GPR65,GPR33,GPR32,GPR183,GPR18,GPR171,GPR158,GPR156,GPR137B,GPR12,GPI,GPD1,GPATCH2L,GPATCH2,GPATCH1,GPAT4,GNL1,GNG2,GNAO1,GMDS,GLT6D1,GLRX2,GLRA3,GLDC,GK5,GK2,GJB6,GJA10,GGCX,GFRA4,GFRA2,GFM1,GDF11,GCH1,GBGT1,GATC,GAS1,GARRE1,GARIN1B,GARIN1A,GALNT7,GALC,GAL,GABRA1,GABARAPL1,FZD4,FUT7,FURIN,FSTL3,FSTL1,FSIP2,FSIP1,FSCB,FRZB,FRY,FRS2,FRMD5,FREM2,FOXL1,FOXA1,FMO4,FMN2,FLYWCH2,FLYWCH1,FLOT1,FLJ14816,FKBP3,FIZ1,FIGN,FIG4,FGR,FGL1,FGFR2,FGF12,FGD6,FFAR4,FES,FBXO48,FBXO34,FBXO21,FBXL17,FBXL16,FBN3,FBLIM1,FAXC,FASTKD5,FASTKD3,FAS,FARP1,FANCI,FANCD2OS,FANCD2,FAM24B,FAM214A,FAM205A,FAM180A,FAM177A1,FAM167A,FAM151A,FAM131B,F5,EXOC3L2,EXOC2,EVX2,EVC,EVA1B,ESYT1,ESRRB,ESD,ERMARD,ERGIC1,ERFE,ERBB4,ERBB3,ENTPD2,ENSG00000288712,ENSG00000288695,ENSG00000288645,ENSG00000288623,ENSG00000286131,ENSG00000286088,ENSG00000285982,ENSG00000285471,ENSG00000285304,ENSG00000285245,ENSG00000284776,ENSG00000283321,ENSG00000279073,ENSG00000277611,ENSG00000276302,ENSG00000273155,ENSG00000269825,ENSG00000269476,ENSG00000269179,ENSG00000266997,ENSG00000266953,ENSG00000260234,ENSG00000260170,ENSG00000259316,ENSG00000258790,ENSG00000258465,ENSG00000258311,ENSG00000257411,ENSG00000257390,ENSG00000256591,ENSG00000255330,ENSG00000249240,ENSG00000241962,ENSG00000241690,ENSG00000206549,ENSG00000170846,ENSG00000111780,EN1,EMILIN3,EMC4,EMC3,ELOVL7,EIF5B,EIF2S3B,EIF2AK2,EHMT2,EGFR,EFNB3,EEF1AKMT1,EDRF1,EDF1,EDARADD,EBNA1BP2,EAF1,DYRK3,DYNLT2,DYNLL1,DYNC1I2,DTD1,DST,DSPP,DRD1,DPYSL4,DPY19L2,DOP1B,DOK1,DOCK3,DNAJC14,DNAJC10,DNAJB6,DMXL1,DMRTA1,DLX6,DLX5,DLL4,DLK2,DLGAP5,DLG1,DIS3,DHX34,DHX16,DHX15,DHRS7C,DHCR24,DEPDC7,DEPDC1B,DENND6B,DENND1B,DEFB131B,DDX54,DDX46,DDX4,DDX24,DDB1,DCSTAMP,DCLK3,DCLK1,DCDC2,DCD,DCBLD2,DCAF8,DCAF12,DCAF1,DBI,DAPP1,DACT1,DACH1,CYP8B1,CYP39A1,CYP2C9,CYP24A1,CYB561A3,CUZD1,CUTA,CTU2,CTNNBL1,CTNNB1,CSRNP3,CSNK1G1,CRY2,CRTAP,CRMP1,CRIP3,CREG2,CREG1,CREBZF,CRB1,CPT1B,CPT1A,CPSF7,CPSF6,CPQ,CPNE5,CPM,CPLX3,CPAMD8,COX6A1,COX16,COQ5,COQ3,COPA,COL9A1,COL5A1,COL4A2,COL1A1,COL11A2,COG6,COCH,COA1,CNTNAP5,CNTFR,CNR1,CNOT2,CNDP2,CNBD1,CLRN1,CLPTM1L,CLPTM1,CLPSL2,CLPB,CLNK,CLEC1B,CLEC19A,CLEC12B,CLEC12A,CLDN6,CLDN17,CLCA4,CLASP2,CKS2,CKAP4,CIZ1,CISH,CIAO2A,CHST2,CHODL,CHL1,CHKB-CPT1B,CHKB,CHD6,CHD3,CHAC1,CH25H,CFHR4,CFAP77,CFAP73,CFAP61,CFAP57,CFAP300,CFAP299,CERCAM,CEP55,CENPB,CEBPZOS,CEBPZ,CEBPB,CDYL,CDKN1C,CDKN1B,CDH24,CDH18,CDCA2,CDC42BPB,CDC25B,CD80,CD63,CD200,CD163,CCT2,CCNT2,CCNT1,CCNJ,CCND1,CCN5,CCN1,CCL21,CCDC9B,CCDC91,CCDC89,CCDC85A,CCDC8,CCDC74A,CCDC73,CCDC7,CCDC66,CCDC60,CCDC33,CCDC198,CCDC190,CCDC183,CCDC148,CCDC14,CCBE1,CC2D2B,CBR4,CBLIF,CAVIN3,CAV3,CATSPERE,CATIP,CASP4,CAND2,CAMSAP1,CAMLG,CAMK2A,CALHM6,CALHM5,CACNA1S,CACNA1I,CABS1,CABP1,CA6,CA12,C9orf24,C6orf89,C6orf62,C6orf136,C6orf120,C5orf24,C5AR2,C5AR1,C4orf51,C4orf33,C3orf18,C2orf88,C2orf76,C2orf72,C2orf15,C1R,C1orf53,C1orf226,C1orf210,C1GALT1,C15orf61,C14orf28,C11orf53,BSND,BRK1,BPNT1,BPIFC,BORCS6,BOLL,BMPR1B,BMP5,BLVRA,BLOC1S1,BLNK,BLID,BEST4,BEST3,BEND5,BCR,BCHE,BBS12,BBS10,BBLN,BACE2,AVPR1B,ATXN2L,ATR,ATP6V0A4,ATP5F1C,ATP4B,ATP4A,ATP2A1,ATL2,ATL1,ATG14,ATAT1,ASIC1,ASF1A,ASB7,ASB4,ARVCF,ARSA,ARPP21,ARPIN,ARMH1,ARMC3,ARL15,ARID1B,ARHGEF40,ARHGAP8,ARHGAP5,ARHGAP21,ARHGAP20,ARCN1,AQP7,AQP6,AQP5,AQP2,APOD,APOC4-APOC2,APOC4,APOC2,APLNR,APH1B,AP000695.1,AOAH,ANXA10,ANKS1A,ANKRD10,ANKDD1A,ANK3,ANAPC15,ANAPC10,AMOTL1,ALS2CL,ALOX15,ALDOB,AKT1,AKR1C4,AKR1C1,AK8,AK7,AJM1,AGA,AFF3,AF131215.5,ADORA2A,ADM2,ADH7,ADGRF5,ADD3,ADAMTS2,ADAM33,ADAM21,ADAD1,ACTR3B,ACTN1,ACTB,ACSL3,ACOT6,ACOT13,ACMSD,ACKR2,ACAN,ACADS,ACAA2,ABRAXAS2,ABRACL,ABHD2,ABHD17C,ABCF1,ABCC10,ABCA4,ABCA2,A2ML1

Supplementary Table 18 Functional enrichment of genes with CDS intersecting with CPC-AIS in at least 5 samples

Database	ID	Description	GeneRatio	BgRatio	Odds ratio	p.adjust	qvalue
BP	GO:0033141	positive regulation of peptidyl-serine phosphorylation of STAT protein	17/1992	20/18800	48.22	1.23E-10	1.17E-10
BP	GO:0033139	regulation of peptidyl-serine phosphorylation of STAT protein	17/1992	23/18800	24.10	3.99E-09	3.77E-09
BP	GO:0042501	serine phosphorylation of STAT protein	17/1992	27/18800	14.46	1.47E-07	1.38E-07
BP	GO:0043330	response to exogenous dsRNA	23/1992	49/18800	7.54	1.86E-07	1.75E-07
BP	GO:0033138	positive regulation of peptidyl-serine phosphorylation	35/1992	107/18800	4.16	5.13E-07	4.84E-07
BP	GO:0043331	response to dsRNA	24/1992	56/18800	6.39	5.13E-07	4.84E-07
BP	GO:0033135	regulation of peptidyl-serine phosphorylation	41/1992	144/18800	3.41	1.72E-06	1.63E-06
BP	GO:0009913	epidermal cell differentiation	55/1992	230/18800	2.70	3.59E-06	3.39E-06
BP	GO:0018209	peptidyl-serine modification	71/1992	338/18800	2.29	7.73E-06	7.30E-06
BP	GO:0031424	keratinization	28/1992	85/18800	4.19	1.23E-05	1.17E-05
BP	GO:0030216	keratinocyte differentiation	43/1992	167/18800	2.97	1.23E-05	1.17E-05
BP	GO:0002323	natural killer cell activation involved in immune response	16/1992	32/18800	8.50	1.34E-05	1.26E-05
BP	GO:0018105	peptidyl-serine phosphorylation	65/1992	315/18800	2.23	4.43E-05	4.18E-05
BP	GO:0008544	epidermis development	70/1992	355/18800	2.11	8.58E-05	8.10E-05
BP	GO:0030198	extracellular matrix organization	61/1992	307/18800	2.13	0.000361	0.000341
BP	GO:0045109	intermediate filament organization	22/1992	68/18800	4.07	0.000361	0.000341
BP	GO:0043062	extracellular structure organization	61/1992	308/18800	2.12	0.000361	0.000341
BP	GO:0045229	external encapsulating structure organization	61/1992	310/18800	2.10	0.000427	0.000403
BP	GO:0030574	collagen catabolic process	16/1992	42/18800	5.23	0.000832	0.000785
BP	GO:0045104	intermediate filament cytoskeleton organization	25/1992	88/18800	3.38	0.000847	0.0008
BP	GO:0006816	calcium ion transport	76/1992	424/18800	1.88	0.000848	0.000801
BP	GO:0045103	intermediate filament-based process	25/1992	89/18800	3.33	0.000964	0.00091
BP	GO:0046425	regulation of receptor signaling pathway via JAK-STAT	27/1992	105/18800	2.95	0.002373	0.002241
BP	GO:0042100	B cell proliferation	26/1992	100/18800	2.99	0.00267	0.002521
BP	GO:0043588	skin development	56/1992	296/18800	2.00	0.002791	0.002635
BP	GO:1902305	regulation of sodium ion transmembrane transport	20/1992	67/18800	3.62	0.002791	0.002635
BP	GO:2000649	regulation of sodium ion transmembrane transporter activity	18/1992	58/18800	3.82	0.004015	0.003791
BP	GO:1904892	regulation of receptor signaling pathway via STAT	28/1992	116/18800	2.71	0.004818	0.004549
BP	GO:0051607	defense response to virus	54/1992	290/18800	1.96	0.005434	0.00513
BP	GO:0140546	defense response to symbiont	54/1992	291/18800	1.95	0.005798	0.005474
BP	GO:0019722	calcium-mediated signaling	41/1992	202/18800	2.17	0.006119	0.005777
BP	GO:0070588	calcium ion transmembrane transport	57/1992	314/18800	1.90	0.006367	0.006012
BP	GO:0019932	second-messenger-mediated signaling	55/1992	303/18800	1.90	0.008416	0.007946
BP	GO:0010959	regulation of metal ion transport	68/1992	403/18800	1.74	0.012394	0.011702
BP	GO:0009615	response to virus	66/1992	392/18800	1.73	0.016317	0.015406
BP	GO:0007626	locomotory behavior	38/1992	192/18800	2.10	0.017159	0.016201
BP	GO:0048839	inner ear development	38/1992	192/18800	2.10	0.017159	0.016201
BP	GO:0032963	collagen metabolic process	24/1992	101/18800	2.65	0.017277	0.016312
BP	GO:0006814	sodium ion transport	46/1992	249/18800	1.93	0.018709	0.017664
BP	GO:0002028	regulation of sodium ion transport	22/1992	90/18800	2.75	0.020078	0.018957
BP	GO:0071287	cellular response to manganese ion	7/1992	13/18800	9.88	0.020514	0.019368
BP	GO:0002286	T cell activation involved in immune response	26/1992	116/18800	2.46	0.023108	0.021817
BP	GO:0034765	regulation of ion transmembrane transport	76/1992	476/18800	1.63	0.023981	0.022641
BP	GO:0035725	sodium ion transmembrane transport	35/1992	177/18800	2.10	0.026931	0.025427
BP	GO:0006874	cellular calcium ion homeostasis	73/1992	456/18800	1.63	0.028093	0.026524
BP	GO:0032412	regulation of ion transmembrane transporter activity	47/1992	263/18800	1.86	0.030178	0.028493
BP	GO:0032943	mononuclear cell proliferation	52/1992	300/18800	1.79	0.030687	0.028973
BP	GO:1904062	regulation of cation transmembrane transport	59/1992	352/18800	1.72	0.030687	0.028973
BP	GO:0007259	receptor signaling pathway via JAK-STAT	34/1992	173/18800	2.08	0.033368	0.031505
BP	GO:0022898	regulation of transmembrane transporter activity	48/1992	273/18800	1.82	0.035417	0.033439
BP	GO:0046651	lymphocyte proliferation	51/1992	296/18800	1.78	0.037854	0.03574
BP	GO:0072503	cellular divalent inorganic cation homeostasis	77/1992	494/18800	1.58	0.038506	0.036355
BP	GO:0042391	regulation of membrane potential	68/1992	425/18800	1.63	0.03919	0.037001
BP	GO:0043583	ear development	40/1992	219/18800	1.90	0.044537	0.04205
BP	GO:0097696	receptor signaling pathway via STAT	35/1992	184/18800	2.00	0.044537	0.04205
BP	GO:0001655	urogenital system development	58/1992	352/18800	1.68	0.044537	0.04205
BP	GO:1904645	response to amyloid-beta	15/1992	55/18800	3.18	0.044537	0.04205
BP	GO:2000650	negative regulation of sodium ion transmembrane transporter activity	7/1992	15/18800	7.41	0.044537	0.04205
BP	GO:0048525	negative regulation of viral process	21/1992	91/18800	2.55	0.044537	0.04205
BP	GO:0055074	calcium ion homeostasis	73/1992	468/18800	1.58	0.045981	0.043413
CC	GO:0043025	neuronal cell body	82/2084	482/19594	1.75	0.005963	0.005485
CC	GO:0045095	keratin filament	26/2084	102/19594	2.90	0.005963	0.005485
MF	GO:0005132	type I interferon receptor binding	16/2031	17/18410	130.05	7.62E-12	7.18E-12
MF	GO:0030280	structural constituent of skin epidermis	23/2031	37/18410	13.39	6.10E-11	5.74E-11
MF	GO:0001637	G protein-coupled chemoattractant receptor activity	11/2031	26/18410	5.94	0.012493	0.011768
MF	GO:0004950	chemokine receptor activity	11/2031	26/18410	5.94	0.012493	0.011768
MF	GO:0016493	C-C chemokine receptor activity	10/2031	23/18410	6.23	0.016829	0.015853
MF	GO:0004896	cytokine receptor activity	24/2031	97/18410	2.67	0.018575	0.017497
MF	GO:0019957	C-C chemokine binding	10/2031	24/18410	5.78	0.018575	0.017497
MF	GO:0008237	metallopeptidase activity	38/2031	189/18410	2.05	0.024113	0.022714
MF	GO:0046873	metal ion transmembrane transporter activity	71/2031	428/18410	1.63	0.035286	0.033238
MF	GO:0004222	metalloendopeptidase activity	25/2031	111/18410	2.36	0.040932	0.038557
KEGG	hsa04622	RIG-I-like receptor signaling pathway	23/897	70/8164	4.04	0.000244	0.000221
KEGG	hsa05167	Kaposi sarcoma-associated herpesvirus infection	42/897	194/8164	2.30	0.001229	0.001112

Database	ID	Description	GeneRatio	BgRatio	Odds ratio	p.adjust	qvalue
BP	GO:0033141	positive regulation of peptidyl-serine phosphorylation of STAT protein	17/1992	20/18800	48.22	1.23E-10	1.17E-10
BP	GO:0033139	regulation of peptidyl-serine phosphorylation of STAT protein	17/1992	23/18800	24.10	3.99E-09	3.77E-09
BP	GO:0042501	serine phosphorylation of STAT protein	17/1992	27/18800	14.46	1.47E-07	1.38E-07
BP	GO:0043330	response to exogenous dsRNA	23/1992	49/18800	7.54	1.86E-07	1.75E-07
KEGG	hsa04974	Protein digestion and absorption	27/897	103/8164	2.94	0.001229	0.001112
KEGG	hsa04630	JAK-STAT signaling pathway	35/897	162/8164	2.28	0.004782	0.004325
KEGG	hsa05162	Measles	31/897	139/8164	2.37	0.005353	0.004842
KEGG	hsa05417	Lipid and atherosclerosis	41/897	215/8164	1.95	0.013155	0.0119
KEGG	hsa04620	Toll-like receptor signaling pathway	24/897	104/8164	2.47	0.013155	0.0119
KEGG	hsa04623	Cytosolic DNA-sensing pathway	17/897	63/8164	3.03	0.013155	0.0119
KEGG	hsa05163	Human cytomegalovirus infection	42/897	225/8164	1.90	0.013287	0.012019
KEGG	hsa04151	PI3K-Akt signaling pathway	59/897	354/8164	1.55	0.019527	0.017664
KEGG	hsa05161	Hepatitis B	32/897	162/8164	8.08	0.019527	0.017664
KEGG	hsa05152	Tuberculosis	34/897	180/8164	7.03	0.028317	0.025615
KEGG	hsa04724	Glutamatergic synapse	24/897	114/8164	5.98	0.030302	0.02741

Note: GeneRatio denotes the ratio of input genes that are annotated in a term, and BgRatio denotes the ratio of all genes that are annotated in a term. The *P*-values are obtained by one-sided Fisher's exact test. They are further adjusted for multiple comparisons using the Benjamini-Hochberg procedure (denoted as the BH-adjusted *P*-value), and are also adjusted for the false discovery rate (denoted as the q-value).

Supplementary Table 19 **Functional enrichment of genes with CDS intersecting with CPC-AIS in at least 1 sample.**

Database	ID	Description	GeneRatio	BgRatio	Odds ratio	p.adjust	qvalue
BP	GO:0031424	keratinization	42/3538	85/18800	4.25	8.98E-07	8.46E-07
BP	GO:0033141	positive regulation of peptidyl-serine phosphorylation of STAT protein	17/3538	20/18800	24.56	8.98E-07	8.46E-07
BP	GO:0030216	keratinocyte differentiation	65/3538	167/18800	2.78	1.68E-06	1.58E-06
BP	GO:0033139	regulation of peptidyl-serine phosphorylation of STAT protein	18/3538	23/18800	15.60	1.68E-06	1.58E-06
BP	GO:0009913	epidermal cell differentiation	80/3538	230/18800	2.33	7.68E-06	7.23E-06
BP	GO:0042501	serine phosphorylation of STAT protein	19/3538	27/18800	10.30	7.68E-06	7.23E-06
BP	GO:0046942	carboxylic acid transport	90/3538	279/18800	2.08	4.19E-05	3.94E-05
BP	GO:0043330	response to exogenous dsRNA	26/3538	49/18800	4.91	6.16E-05	5.80E-05
BP	GO:0015849	organic acid transport	97/3538	318/18800	1.92	0.000193	0.000182
BP	GO:0006814	sodium ion transport	80/3538	249/18800	2.07	0.000193	0.000182
BP	GO:0015711	organic anion transport	107/3538	361/18800	1.84	0.000202	0.00019
BP	GO:0043588	skin development	91/3538	296/18800	1.94	0.000237	0.000223
BP	GO:0018209	peptidyl-serine modification	101/3538	338/18800	1.86	0.000237	0.000223
BP	GO:0043331	response to dsRNA	27/3538	56/18800	4.04	0.000242	0.000228
BP	GO:0006816	calcium ion transport	121/3538	424/18800	1.75	0.000242	0.000228
BP	GO:0018105	peptidyl-serine phosphorylation	94/3538	315/18800	1.86	0.000505	0.000475
BP	GO:0008544	epidermis development	103/3538	355/18800	1.79	0.000614	0.000578
BP	GO:0033135	regulation of peptidyl-serine phosphorylation	51/3538	144/18800	2.39	0.000614	0.000578
BP	GO:0033138	positive regulation of peptidyl-serine phosphorylation	41/3538	107/18800	2.70	0.000614	0.000578
BP	GO:0070588	calcium ion transmembrane transport	93/3538	314/18800	1.84	0.000654	0.000616
BP	GO:0030198	extracellular matrix organization	91/3538	307/18800	1.84	0.000769	0.000724
BP	GO:0043062	extracellular structure organization	91/3538	308/18800	1.83	0.000852	0.000802
BP	GO:0045229	external encapsulating structure organization	91/3538	310/18800	1.81	0.001094	0.00103
BP	GO:0006688	glycosphingolipid biosynthetic process	18/3538	33/18800	5.20	0.001237	0.001164
BP	GO:0006874	cellular calcium ion homeostasis	124/3538	456/18800	1.63	0.001534	0.001443
BP	GO:0006687	glycosphingolipid metabolic process	26/3538	60/18800	3.32	0.002611	0.002458
BP	GO:0002323	natural killer cell activation involved in immune response	17/3538	32/18800	4.91	0.003199	0.003011
BP	GO:0055074	calcium ion homeostasis	125/3538	468/18800	1.59	0.003199	0.003011
BP	GO:0019722	calcium-mediated signaling	63/3538	202/18800	1.97	0.003315	0.00312
BP	GO:0072503	cellular divalent inorganic cation homeostasis	130/3538	494/18800	1.56	0.004395	0.004137
BP	GO:0035725	sodium ion transmembrane transport	55/3538	177/18800	1.96	0.011431	0.010759
BP	GO:2000649	regulation of sodium ion transmembrane transporter activity	24/3538	58/18800	3.06	0.011431	0.010759
BP	GO:0010959	regulation of metal ion transport	107/3538	403/18800	1.58	0.014124	0.013293
BP	GO:0042100	B cell proliferation	35/3538	100/18800	2.34	0.017284	0.016268
BP	GO:0035330	regulation of hippo signaling	12/3538	21/18800	5.77	0.01837	0.01729
BP	GO:1902305	regulation of sodium ion transmembrane transport	26/3538	67/18800	2.75	0.01837	0.01729
BP	GO:0070085	glycosylation	70/3538	244/18800	1.75	0.01837	0.01729
BP	GO:0046425	regulation of receptor signaling pathway via JAK-STAT	36/3538	105/18800	2.26	0.020048	0.018868
BP	GO:0060326	cell chemotaxis	86/3538	315/18800	1.64	0.021175	0.01993
BP	GO:0097396	response to interleukin-17	8/3538	11/18800	11.53	0.021917	0.020628
BP	GO:0097398	cellular response to interleukin-17	8/3538	11/18800	11.53	0.021917	0.020628
BP	GO:0097400	interleukin-17-mediated signaling pathway	8/3538	11/18800	11.53	0.021917	0.020628
BP	GO:0051480	regulation of cytosolic calcium ion concentration	95/3538	356/18800	1.59	0.021917	0.020628
BP	GO:0009100	glycoprotein metabolic process	101/3538	386/18800	1.54	0.029822	0.028068
BP	GO:0048705	skeletal system morphogenesis	65/3538	228/18800	1.73	0.031655	0.029794
BP	GO:0043406	positive regulation of MAP kinase activity	38/3538	116/18800	2.11	0.031854	0.029981
BP	GO:0032412	regulation of ion transmembrane transporter activity	73/3538	263/18800	1.67	0.031854	0.029981
BP	GO:0038093	Fc receptor signaling pathway	21/3538	52/18800	2.93	0.032426	0.030519
BP	GO:0019932	second-messenger-mediated signaling	82/3538	303/18800	1.61	0.032426	0.030519
BP	GO:0006865	amino acid transport	45/3538	145/18800	1.95	0.033515	0.031543
BP	GO:0051924	regulation of calcium ion transport	70/3538	251/18800	1.68	0.033515	0.031543
BP	GO:0015718	monocarboxylic acid transport	38/3538	117/18800	2.09	0.034358	0.032337
BP	GO:0002028	regulation of sodium ion transport	31/3538	90/18800	2.28	0.036536	0.034387
BP	GO:0009247	glycolipid biosynthetic process	26/3538	71/18800	2.50	0.036536	0.034387
BP	GO:0001573	ganglioside metabolic process	13/3538	26/18800	4.33	0.036536	0.034387
BP	GO:0009615	response to virus	101/3538	392/18800	1.51	0.042201	0.039719
BP	GO:0045109	intermediate filament organization	25/3538	68/18800	2.52	0.042201	0.039719
BP	GO:1901019	regulation of calcium ion transmembrane transporter activity	31/3538	91/18800	2.24	0.042369	0.039877
BP	GO:0043507	positive regulation of JUN kinase activity	18/3538	43/18800	3.12	0.042369	0.039877
BP	GO:0006664	glycolipid metabolic process	34/3538	103/18800	2.14	0.042369	0.039877
BP	GO:0051928	positive regulation of calcium ion transport	38/3538	119/18800	2.03	0.042369	0.039877
BP	GO:0071875	adrenergic receptor signaling pathway	15/3538	33/18800	3.61	0.042369	0.039877
BP	GO:1905039	carboxylic acid transmembrane transport	46/3538	152/18800	1.88	0.042369	0.039877
BP	GO:0051607	defense response to virus	78/3538	290/18800	1.60	0.042369	0.039877

Database	ID	Description	GeneRatio	BgRatio	Odds ratio	p.adjust	qvalue
BP	GO:0022898	regulation of transmembrane transporter activity	74/3538	273/18800	1.62	0.042811	0.040293
BP	GO:0019221	cytokine-mediated signaling pathway	121/3538	486/18800	1.45	0.042811	0.040293
BP	GO:0098657	import into cell	66/3538	238/18800	1.67	0.042811	0.040293
BP	GO:0006486	protein glycosylation	63/3538	225/18800	1.69	0.042811	0.040293
BP	GO:0043413	macromolecule glycosylation	63/3538	225/18800	1.69	0.042811	0.040293
BP	GO:0140546	defense response to symbiont	78/3538	291/18800	1.59	0.042811	0.040293
BP	GO:0003333	amino acid transmembrane transport	33/3538	100/18800	2.14	0.042811	0.040293
BP	GO:1904892	regulation of receptor signaling pathway via STAT	37/3538	116/18800	2.03	0.042811	0.040293
BP	GO:1903825	organic acid transmembrane transport	46/3538	153/18800	1.87	0.042811	0.040293
BP	GO:1903509	liposaccharide metabolic process	34/3538	104/18800	2.11	0.042811	0.040293
BP	GO:0071880	adenylate cyclase-activating adrenergic receptor signaling pathway	13/3538	27/18800	4.02	0.042811	0.040293
BP	GO:0032409	regulation of transporter activity	81/3538	305/18800	1.57	0.042817	0.040299
BP	GO:0038095	Fc-epsilon receptor signaling pathway	12/3538	24/18800	4.33	0.044393	0.041782
BP	GO:0031398	positive regulation of protein ubiquitination	38/3538	121/18800	1.99	0.048131	0.0453
CC	GO:0045121	membrane raft	94/3730	326/19594	1.74	0.003156	0.00291
CC	GO:0098857	membrane microdomain	94/3730	327/19594	1.73	0.003156	0.00291
CC	GO:0045095	keratin filament	38/3730	102/19594	2.54	0.003156	0.00291
CC	GO:0005938	cell cortex	87/3730	310/19594	1.68	0.012205	0.011254
CC	GO:0043025	neuronal cell body	124/3730	482/19594	1.49	0.019912	0.018361
CC	GO:0045177	apical part of cell	111/3730	424/19594	1.52	0.019912	0.018361
CC	GO:0030286	dynein complex	22/3730	54/19594	2.94	0.019912	0.018361
CC	GO:0062023	collagen-containing extracellular matrix	110/3730	429/19594	1.48	0.038923	0.03589
CC	GO:0030315	T-tubule	20/3730	51/19594	2.75	0.047592	0.043884
CC	GO:0005775	vacuolar lumen	51/3730	174/19594	1.77	0.047592	0.043884
CC	GO:0016324	apical plasma membrane	93/3730	358/19594	1.51	0.047592	0.043884
CC	GO:0043202	lysosomal lumen	32/3730	97/19594	2.10	0.047592	0.043884
MF	GO:0030280	structural constituent of skin epidermis	28/3640	37/18410	12.71	4.00E-10	3.66E-10
MF	GO:0005132	type I interferon receptor binding	16/3640	17/18410	65.21	4.37E-08	4.01E-08
MF	GO:0038024	cargo receptor activity	33/3640	79/18410	2.93	0.002444	0.00224
MF	GO:0004707	MAP kinase activity	11/3640	15/18410	11.19	0.003265	0.002992
MF	GO:0004896	cytokine receptor activity	37/3640	97/18410	2.52	0.004986	0.004569
MF	GO:0005044	scavenger receptor activity	21/3640	47/18410	3.29	0.012946	0.011862
MF	GO:0016493	C-C chemokine receptor activity	13/3640	23/18410	5.29	0.012946	0.011862
MF	GO:0015179	L-amino acid transmembrane transporter activity	25/3640	61/18410	2.83	0.012946	0.011862
MF	GO:0001637	G protein-coupled chemoattractant receptor activity	14/3640	26/18410	4.75	0.012946	0.011862
MF	GO:0004950	chemokine receptor activity	14/3640	26/18410	4.75	0.012946	0.011862
MF	GO:0004712	protein serine/threonine/tyrosine kinase activity	120/3640	446/18410	1.51	0.012946	0.011862
MF	GO:0008514	organic anion transmembrane transporter activity	58/3640	186/18410	1.85	0.012946	0.011862
MF	GO:0030169	low-density lipoprotein particle binding	11/3640	18/18410	6.39	0.012946	0.011862
MF	GO:0015293	symporter activity	47/3640	144/18410	1.98	0.014917	0.013669
MF	GO:0019957	C-C chemokine binding	13/3640	24/18410	4.81	0.015063	0.013802
MF	GO:0008237	metallopeptidase activity	58/3640	189/18410	1.81	0.016412	0.015038
MF	GO:0046943	carboxylic acid transmembrane transporter activity	51/3640	164/18410	1.84	0.025297	0.023179
MF	GO:0005342	organic acid transmembrane transporter activity	51/3640	165/18410	1.83	0.028034	0.025688
MF	GO:0005496	steroid binding	34/3640	100/18410	2.10	0.035983	0.032971
MF	GO:0008509	anion transmembrane transporter activity	86/3640	315/18410	1.54	0.041413	0.037947
MF	GO:0106310	protein serine kinase activity	96/3640	360/18410	1.49	0.046144	0.042281
MF	GO:0017080	sodium channel regulator activity	17/3640	40/18410	3.01	0.046144	0.042281
KEGG	hsa04622	RIG-I-like receptor signaling pathway	32/1614	70/8164	3.47	0.000242	0.000221
KEGG	hsa04974	Protein digestion and absorption	39/1614	103/8164	2.51	0.001775	0.001616
KEGG	hsa05167	Kaposi sarcoma-associated herpesvirus infection	63/1614	194/8164	1.99	0.001775	0.001616
KEGG	hsa04620	Toll-like receptor signaling pathway	37/1614	104/8164	2.27	0.008745	0.007958
KEGG	hsa05162	Measles	46/1614	139/8164	2.04	0.008745	0.007958
KEGG	hsa05161	Hepatitis B	51/1614	162/8164	1.89	0.011908	0.010835
KEGG	hsa05417	Lipid and atherosclerosis	64/1614	215/8164	1.75	0.011908	0.010835
KEGG	hsa04936	Alcoholic liver disease	45/1614	142/8164	1.91	0.019236	0.017504
KEGG	hsa00604	Glycosphingolipid biosynthesis - ganglio series	9/1614	15/8164	6.12	0.026153	0.023798
KEGG	hsa05171	Coronavirus disease - COVID-19	66/1614	232/8164	1.64	0.026621	0.024224
KEGG	hsa04217	Necroptosis	48/1614	159/8164	1.78	0.029579	0.026915
KEGG	hsa04621	NOD-like receptor signaling pathway	54/1614	184/8164	1.71	0.029579	0.026915
KEGG	hsa04630	JAK-STAT signaling pathway	48/1614	162/8164	1.73	0.036821	0.033505
KEGG	hsa04024	cAMP signaling pathway	62/1614	221/8164	1.61	0.036821	0.033505
KEGG	hsa04657	IL-17 signaling pathway	31/1614	94/8164	2.02	0.036821	0.033505

Note: GeneRatio denotes the ratio of input genes that are annotated in a term, and BgRatio denotes the ratio of all genes that are annotated in a term. The *P*-values are obtained by one-sided Fisher's exact test. They are further adjusted for multiple comparisons using the Benjamini-Hochberg procedure (denoted as the BH-adjusted *P*-value), and are also adjusted for the false discovery rate (denoted as the q-value).

Supplementary Table 20 Functional enrichment of genes with CDS intersecting with CPC-AIS in at least 10 samples

Database	ID	Description	GeneRatio	BgRatio	Odds ratio	p.adjust	qvalue
BP	GO:0031424	keratinization	24/1215	85/18800	5.79	2.18E-06	2.10E-06
BP	GO:0009913	epidermal cell differentiation	42/1215	230/18800	3.31	2.18E-06	2.10E-06
BP	GO:0030216	keratinocyte differentiation	34/1215	167/18800	3.78	3.27E-06	3.14E-06
BP	GO:0045109	intermediate filament organization	20/1215	68/18800	6.11	7.76E-06	7.45E-06
BP	GO:0045104	intermediate filament cytoskeleton organization	22/1215	88/18800	4.89	2.99E-05	2.87E-05
BP	GO:0045103	intermediate filament-based process	22/1215	89/18800	4.82	3.11E-05	2.98E-05
BP	GO:0008544	epidermis development	51/1215	355/18800	2.49	4.99E-05	4.79E-05
BP	GO:0043588	skin development	42/1215	296/18800	2.44	0.000869	0.000834
BP	GO:0033135	regulation of peptidyl-serine phosphorylation	25/1215	144/18800	3.08	0.003038	0.002917
BP	GO:0071287	cellular response to manganese ion	7/1215	13/18800	16.98	0.003038	0.002917
BP	GO:0030574	collagen catabolic process	12/1215	42/18800	5.84	0.00445	0.004271
BP	GO:0018105	peptidyl-serine phosphorylation	41/1215	315/18800	2.21	0.006206	0.005958
BP	GO:0033138	positive regulation of peptidyl-serine phosphorylation	20/1215	107/18800	3.37	0.006206	0.005958
BP	GO:0018209	peptidyl-serine modification	43/1215	338/18800	2.15	0.006325	0.006072
BP	GO:0007605	sensory perception of sound	25/1215	156/18800	2.80	0.008129	0.007803
BP	GO:0010042	response to manganese ion	8/1215	22/18800	8.32	0.014182	0.013613
BP	GO:0050954	sensory perception of mechanical stimulus	26/1215	177/18800	2.52	0.022934	0.022015
BP	GO:0030198	extracellular matrix organization	38/1215	307/18800	2.08	0.025391	0.024373
BP	GO:0001655	urogenital system development	42/1215	352/18800	2.00	0.025391	0.024373
BP	GO:0043062	extracellular structure organization	38/1215	308/18800	2.07	0.025391	0.024373
BP	GO:1902229	regulation of intrinsic apoptotic signaling pathway in response to DNA damage	10/1215	38/18800	5.20	0.025391	0.024373
BP	GO:0045229	external encapsulating structure organization	38/1215	310/18800	2.05	0.025391	0.024373
BP	GO:0007626	locomotory behavior	27/1215	192/18800	2.40	0.025391	0.024373
BP	GO:0048839	inner ear development	27/1215	192/18800	2.40	0.025391	0.024373
BP	GO:0072001	renal system development	38/1215	312/18800	2.04	0.027337	0.026242
BP	GO:0006814	sodium ion transport	32/1215	249/18800	2.16	0.032677	0.031367
BP	GO:0010975	regulation of neuron projection development	48/1215	431/18800	1.85	0.032677	0.031367
BP	GO:0043583	ear development	29/1215	219/18800	2.24	0.03594	0.034499
BP	GO:0045747	positive regulation of Notch signaling pathway	11/1215	48/18800	4.33	0.03594	0.034499
CC	GO:0045095	keratin filament	21/1275	102/19594	3.77	0.001306	0.001212
CC	GO:0043202	lysosomal lumen	17/1275	97/19594	3.08	0.049453	0.045878
CC	GO:0043025	neuronal cell body	52/1275	482/19594	1.77	0.049453	0.045878
MF	GO:0030280	structural constituent of skin epidermis	19/1238	37/18410	14.85	2.54E-10	2.37E-10

Note: GeneRatio denotes the ratio of input genes that are annotated in a term, and BgRatio denotes the ratio of all genes that are annotated in a term. The *P*-values are obtained by one-sided Fisher's exact test. They are further adjusted for multiple comparisons using the Benjamini-Hochberg procedure (denoted as the BH-adjusted *P*-value), and are also adjusted for the false discovery rate (denoted as the q-value).

Supplementary Table 21 Genes and Frequencies of CDS Regions Covered by CPC-specific Archaic Introgression Segments.

Frequency	Genes
50	*CSMD2*
48	*IL17RA,COL27A1*
46	*ANKS1B*
43	*CTNND2*
41	*PTPRN2*
40	*TSPAN11*
39	*SLC35F3,NCOR2*
38	*RIMBP2,AGBL1*
37	*SLC35F4,HECW2,ETFBKMT*
36	*NAV2*
35	*PCSK6,AMN1*
33	*COL13A1*
31	*TMEM121B,TIAM1,FZD10*
30	*TRA2B,LDLRAD3*
29	*NPAS3,CSMD1*
28	*MYO3B*
27	*MMP17*
26	*KAZN*
24	*KCNQ5,GRM7,B9D1*
23	*EPN2,ARHGAP26,ANK2*
22	*EYS*
21	*TMEM132C,TMED3,SLC9A9,SINHCAF,RELN,FSTL5*
20	*TMEM132D,KCNQ1,DYNC2I1,APP*
19	*SORL1,PYGL,MGAT5*
18	*ULK4,TRAK1,NTRK2,ESYT2*
17	*RPUSD1,PRKAG2,PFKFB3,HDHD5,GABRB3,GABRA5,GAB4*
16	*NLRP5,NCAPG2,MAP3K5,CLMP,ALPK1*
15	*H3-5*
14	*SLC38A1,SHANK2,PCNX2,LIPA,CCSER1,CACNA2D1*
13	*SLC47A1,SHC2,PTPRF,PRKN,GALNT9*
12	*ZGRF1,WDR27,TMEM247,SGSM1,RTN4R,PRTFDC1,PRMT8,LDLRAD4,ITGA11,EPB41L3,ENSG00000285733,ELMO1,ADA2*
11	*TRPM3,SLC13A3,SH2D4A,RFX2,RAI14,PACRG,MRO,KIRREL3,ICE1,FKBP5,EP400,DPP6,CRACR2A,CAPRIN2,ABCB5*
10	*ULK2,PIWIL1,PHACTR2,PDE7B,MYH13,MLNR,GZMH,FNDC3A,DDX11,CRYL1,CDADC1,CAMK1D,CAB39L,C10orf90,ANKRD44,ALDH3A1,ADAMTS16*
9	*ULK1,TRPV3,RNF112,RBM47,NOC4L,MFAP4,MAPK7,KLRD1,IPO8,IGSF21,GABRB2,DLGAP1,DLG2,DISC1,DGKH,CORO2B,CNIH3,CAMKK2,C1orf94*
8	*SNX5,SEZ6L,PUS1,PLCH1,NEUROG2,MLIP,MGME1,MAPK4,KCTD16,DDX51,AQP12B,AQP12A*
7	*ZDHHC8,USP2,TRMT2A,TBC1D17,SULT1C4,ST8SIA2,SP8,SLC39A11,RPH3AL,RBM20,RANBP1,PRRX2,PNKP,PHF21B,PEX3,NUGGC,NTMT1,MLPH,LIMS1,KCNIP4,GRIK3,GCC2,GAREM1,FUCA2,ETV6,DGCR8,CDCP1,C9orf50,ASB6,AKT1S1,AKAP12,ABTB1*
6	*ZBTB8A,UNC45B,TXNDC15,TSPYL5,STIMATE-MUSTN1,STIMATE,SPECC1,SLIT2,SLC39A12,SLC35G3,SFMBT1,SDK2,RNASE2,RFT1,RAX,PCBD2,PAXIP1,P2RX7,OLFML2B,MUSTN1,MOB3B,MGLL,MGAM2,METTL17,MEOX1,ITIH4,ITIH3,HERC1,GPR35,GOLIM4,GNG12,FRMD1,FOXN3,FCRLB,FCRLA,F13A1,ENSG00000272305,ENSG00000254553,ENSG00000243696,DNAH5,CREBL2,CCL3,CATSPER3,BRPF3,AKAP11,ADA,ACYP2*
5	*ZBTB8B,XRCC4,USP3,UAP1,TMTC4,TLN2,TIE1,TBC1D16,SULT1C3,SMPD3,SMIM31,SLFN11,RPS6KA2,RIPOR2,RCSD1,RANBP2,RAB37,PRSS41,PRMT7,POP1,PLSCR1,PLCH2,P2RX4,OVOL2,NUAK1,NTM,MDGA2,MAP4K2,LRP2,LMF1,ITIH1,ITGB2,INSR,IL20,HS3ST2,GREB1,GPR19,GPR176,GLRX5,GLIS1,GALNT17,FBXL22,FAM110A,ENSG00000258989,ENSG00000249209,ENPEP,ELP3,EDDM13,CXCL13,CNTN3,CLIC5,CD4,CCL20,CCDC138,C5orf49,C5orf15,ATP5PO,ATP2B2,ARNT2,ANGPT4,ANAPC5,AIG1,AFAP1L2,ADAT2*
4	*ZNF804A,ZNF438,ZBTB40,VIPR2,TMEM167A,TMEM127,TMEM120B,THEM5,TARBP1,TAB1,SZT2,SYNGR1,SYNE3,SUSD2,SULT1C2,STRN3,STARD7,SRPK1,SQOR,SOX8,SNRNP200,SLC2A9,SLC26A8,SCGB2A2,SCGB1D4,SCGB1A1,RNASE3,PPARD,PLXNC1,PGLYRP4,PGLYRP3,PCSK5,ORAI1,ODF3L2,OASL,NEURL3,NCAPH,MTHFD1L,MTARC1,MPL,MORN3,MED8,MAPK14,LORICRIN,LHFPL5,KDM2B,ITPRIPL1,HYI,HEATR5A,GPC5,GALNTL6,FXYD7,FXYD1,ENSG00000286231,ENSG00000249624,ELOVL1,EIF2AK4,EDIL3,DYNC1I1,DMRTB1,DENND5B,DEF6,CYP4F8,CLPSL1,CLIP1,CIAO1,CDC20,CD9,CCDC149,CABIN1,BPIFA3,ASRGL1,ARMC12,AMER2,AK4P3*

Frequency	Genes
3	*ZNF777,ZNF76,ZNF662,ZNF618,VDR,VDAC1,UHMK1,TSPEAR,TRPV1,TRIM9,TRIM66,TPD52L1,TNIP1,TNFAIP8,TMEM82,TMEM163,TMEM106C,STK32C,STAB1,SPNS3,SMIM29,SMARCB1,SLX4IP,SLITRK1,SLC5A7,SLC47A2,SLC2A1,SLC25A34,SIGLEC8,SEPTIN9,SCUBE3,SCGB2A1,SCGB1D2,SCGB1D1,RSC1A1,RPS24,RPL36AL,RNF34,RGL1,RESF1,POLR3A,POLE2,PLEKHM2,PIWIL4,NTNG1,NR3C2,NISCH,NCF2,MUC13,MTRF1,MRE11,MPC1,MMP11,MIS18BP1,MGAT2,MAPK13,LRR1,LGI4,LBHD2,KRBOX1,KCTD9,KCNMA1,ISG20L2,HTT,HSD17B4,HMGA1,HLX,GPR83,GNRH1,GFOD1,GASK1A,FXYD3,FGD4,FANCM,FANCC,EXOC3L4,ENSG00000285526,ENSG00000273291,EGR3,DOCK5,DNAJC16,DNAH9,DNAAF2,DIPK1C,DERL3,DDI2,DAP,COLEC12,COL2A1,CLPS,CHSY3,CCL18,CASP9,C3orf33,BTBD2,BPIFA2,BICD1,BCAT1,B3GALT5,ATP2A3,ATP12A,ARPC5,ARHGAP10,APOBEC4,AP4S1,AKAIN1,AGMAT,AFAP1L1*
2	*ZNF746,ZBTB41,ZBTB2,XPR1,WIPF3,WDR89,VWF,TUFT1,TRAV39,TMEM192,TMEM121,TMEFF2,TIA1,TGFB2,TESPA1,TEDC1,TDRKH,TBX15,STK33,STIM2,SPRTN,SPNS1,SPARC,SNX27,SNRPG,SMIM34,SMIM11,SLC9C1,SLC6A6,SLC22A18,SLC11A1,SKI,SHPK,SGPP1,SF3B1,SERPINB6,SCN8A,SCG2,RPS29,RORC,RNASE12,RNASE11,RMND1,RIIAD1,RGS1,RFTN2,RAP1GAP2,RABEP2,RAB6C,PTPRG,PIP5K1C,PHLDA2,PGAP1,PEX10,PELI2,PEBP4,PDHA2,PCYOX1,PCGF3,PAH,OR7E24,OR6C6,OR13D1,OR13C9,OR13C8,OR13C5,OR13C2,OAZ3,NXPH1,NOS1AP,NOL10,NIPSNAP3B,NIPSNAP3A,NFATC2IP,NEMF,MYH8,MYH4,MYH2,MYH1,MTA1,MRPS6,MRPL9,MOB4,MIF,MGAM,MAPK8,MAP4K3,MAN1B1,LRRK1,LRP5,LPP,LINGO4,LHFPL6,LAT,KRTDAP,KRTAP12-4,KRTAP12-3,KRTAP12-2,KRTAP12-1,KRTAP10-9,KRTAP10-11,KRTAP10-10,KLHDC2,KLHDC1,KLF12,KIAA1614,KCNE2,ITPR1,IL10RB,IGLL1,IFNAR2,IFNAR1,IBTK,HSPE1-MOB4,HSPE1,HSPD1,HSD17B7,HIF1A,HERPUD1,GTF3C4,GTF3C3,GSTT2B,GSTT2,GRM4,GRIN1,GPR107,GP2,GCSAM,GCNT2,GAK,FOXD1,FNBP1,FEM1B,FCER2,FBXO42,FAM136A,F13B,EXOC8,EXOC1L,EXOC1,ENSG00000289697,ENSG00000287856,ENSG00000285762,ENSG00000262304,ENSG00000259171,ENSG00000259060,ENSG00000257341,ENSG00000251357,ENSG00000203546,ELAVL4,EGLN1,DTD2,DRD5,DPYS,DPP7,DMKN,DENND2B,DDX31,DDTL,DDT,DDR2,DCC,CTNS,CRY1,CRIP2,CRIP1,CRADD,CRACDL,CPLANE2,COQ10B,CNTN6,CNTLN,CNGB3,CNGA3,CLU,CLN6,CFHR5,CFHR3,CFHR2,CFHR1,CFH,CEP83,CEP164,CELF3,CELA2B,CDC7,CD53,CD300LF,CD19,CCNB1IP1,CCDC68,CCDC170,CCDC150,C3orf52,C3,C2orf66,C2CD4D,C21orf140,C1orf134,BMF,B4GALT6,ATP6V1C2,ASPM,ARPIN-AP3S2,ARHGEF19,ARHGAP22,ARF6,APOLD1,APCDD1L,AP3S2,AOPEP,ANKRD34C,ANG,AL589765.1,AHNAK,AGMO,ABCA1*
1	*ZNF582,ZNF518B,ZNF219,ZNF133,ZKSCAN2,ZHX3,ZC3H12C,XRN1,XPO6,XPO4,XKR3,WRAP53,WNT5B,WLS,WDR1,VWCE,VPS18,VAPB,USP10,URI1,UNC5C,UHRF1,UGT1A9,UGT1A8,UGT1A7,UGT1A6,UGT1A5,UGT1A4,UGT1A3,UGT1A10,UGT1A1,UBR2,UBE2V1,UAP1L1,TYMP,TUFM,TTC5,TSPYL4,TSPYL1,TSPAN9,TSNAX-DISC1,TSC1,TRPM8,TRPM5,TRPC1,TRNT1,TRDV3,TRDJ4,TRDJ3,TRDJ2,TRDJ1,TRDD3,TRDC,TRAV9-2,TRAV8-6,TRAV14DV4,TRAV12-3,TPTE2,TPBG,TP53,TOP1,TOGARAM2,TOGARAM1,TNS1,TNFSF14,TNFRSF14,TNFAIP8L3,TNFAIP2,TMPRSS2,TMPRSS15,TMEM53,TMEM255B,TMEM253,TMEM250,TMEM233,TMEM216,TMEM178A,TMEM138,TMEM132B,TKFC,THUMPD2,THRAP3,THBS1,TGM6,TESMIN,TCP11,TCF21,TBP,TBC1D22A,SYT9,SYNJ2BP-COX16,SYNJ2BP,SYCE3,SVIL,SUSD5,STX2,STPG2,STON2,STK40,STK32B,SRD5A3,SPNS2,SPINT1,SPATA46,SPATA17,SPACA9,SOX18,SOGA3,SNX10,SNAP25,SMG7,SMARCD1,SLC49A4,SLC43A2,SLC39A8,SLC35E3,SLC19A3,SLC12A6,SKIDA1,SIM2,SH3D21,SH2B1,SGCA,SFXN5,SFXN1,SETD7,SEC24A,SEC16A,SDHAF2,SCTR,SCO2,SCN3B,SCN10A,SCLT1,SBF1,SAPCD2,SAMD14,RUFY4,RUBCNL,RSPO3,RRP1B,RPS8,RPS10-NUDT3,RPS10,RPL27A,RPGRIP1L,RNF220,RNF113B,RNASE9,RIMKLB,RIDA,RHOV,RGS21,RGS18,RFLNA,RELB,RDX,RDH10,RAP1B,RACGAP1,RABL6,RAB27B,RAB20,RAB11FIP3,QSER1,PXT1,PWWP2B,PTTG1IP,PTPRH,PTPN6,PTGIS,PTCHD4,PSMD1,PSMB1,PRXL2B,PRSS12,PRRG4,PRR5-ARHGAP8,PRPF39,PROC,PPP6R3,PPP6R2,PPP5C,PPP1R9B,PPP1R32,PPM1L,PPIL1,POLR3G,POLG,PNP,PNKD,PMEL,PLXNA4,PLS1,PLOD1,PLCL1,PLCG1,PKHD1,PHPT1,PHIP,PHF10,PGM1,PGA5,PGA4,PGA3,PEPD,PEDS1-UBE2V1,PEDS1,PDXP,PDXK,PDIA6,PDCD2,PCOLCE2,PCDH1,PCARE,PARP2,PAQR9,OTUD4,OSBPL3,OSBP2,OR6Q1,OR6C74,OR5AU1,ODF3B,NUP35,NUP107,NUDT3,NUBPL,NT5DC1,NRROS,NRP2,NPDC1,NFKB1,NETO1,NEK4,NDUFA10,NDST3,NDRG2,NCL,NCAPH2,NBPF3,MYO10,MVD,MTUS1,MTIF3,MSX1,MRPL46,MPHOSPH8,MMP21,MMEL1,MLLT10,MKRN3,MKKS,MIOX,MGAT4A,MFAP5,MERTK,MEP1B,MED10,MDM2,MDFIC2,MARS2,MAPK8IP2,MAP7D1,MAP3K19,MANBA,MAMDC4,LYZL2,LYSMD3,LSAMP,LRRTM1,LRFN2,LPIN3,LONRF2,LNPK,LMF2,LHX9,LGI2,LGALS1,LCN9,LARS2,KRTAP10-5,KRTAP10-4,KRTAP10-12,KRT19,KRT15,KRBA1,KLK14,KLHL3,KLHL28,KLHDC7B,KIF2C,KIAA2013,KIAA0408,KCNH5,KCNF1,KCNE1,KCNAB1,KATNBL1,JADE1,ITIH5,ITGA1,IRAK1BP1,INPP5A,INO80,INHBB,IL17D,IKZF3,IKZF2,IFT74,IFT43,HTR2B,HSPBAP1,HSF2BP,HPCAL1,HOXD9,HOXD13,HOXD12,HOXD11,HOXD10,HMCN1,HLA-DRB5,HK2,HECA,HDDC2,HDAC11,H2BC12L,GYS1,GTF3A,GRP,GRK1,GRHL1,GPX3,GPR33,GPR32,GPR158,GPD1,GPATCH2,GNG2,GNAO1,GLT6D1,GK2,GJB6,GFRA2,GBGT1,GAL,GABARAPL1,FUT7,FURIN,FSIP2,FSIP1,FRMD5,FOXL1,FMO4,FKBP3,FIG4,FGR,FGL1,FGFR2,FES,FBXO21,FBXL17,FBLIM1,FARP1,FANCI,FAM131B,EXOC3L2,EVX2,EVA1B,ESD,ERMARD,ERGIC1,ENTPD2,ENSG00000288695,ENSG00000285304,ENSG00000285245,ENSG00000279073,ENSG00000260234,ENSG00000260170,ENSG00000256591,ENSG00000255330,EMILIN3,EMC4,EFNB3,EEF1AKMT1,EDRF1,EDF1,DYNLT2,DRD1,DPYSL4,DMRTA1,DLL4,DHX15,DHCR24,DEPDC7,DENND1B,DDX46,DDB1,DACH1,CYP8B1,CYP24A1,CYB561A3,CTNNB1,CRMP1,CRB1,CPT1B,CPT1A,CPSF7,CPNE5,CPM,CPAMD8,COX16,COL4A2,COL1A1,CNTNAP5,CLRN1,CLPTM1L,CLPTM1,CLPSL2,CLNK,CLEC1B,CLEC12B,CLEC12A,CLDN6,CLASP2,CIZ1,CHODL,CHL1,CHKB-CPT1B,CHKB,CHD6,CHAC1,CFHR4,CFAP77,CFAP61,CFAP300,CFAP299,CEBPB,CDYL,CDKN1C,CDKN1B,CDH24,CDCA2,CDC42BPB,CD200,CCNT2,CCND1,CCN5,CCDC8,CCDC73,CCDC198,CCDC190,CCDC183,CCBE1,CAVIN3,CATSPERE,CATIP,CASP4,CAMSAP1,CAMLG,CABS1,C6orf89,C6orf120,C5orf24,C4orf51,C4orf33,C2orf72,C1orf53,C1orf226,C1GALT1,C14orf28,BSND,BOLL,BMPR1B,BEST4,BBLN,BACE2,ATXN2L,ATR,ATP4A,ATP2A1,ATL2,ASIC1,ARSA,ARPIN,ARMH1,ARID1B,ARHGEF40,ARHGAP8,ARHGAP5,ARHGAP21,ARHGAP20,AQP6,AQP5,AQP2,APOC4-APOC2,APOC4,APOC2,AOAH,ANKS1A,ANKRD10,AMOTL1,AKT1,AK8,AJM1,AF131215.5,ADM2,ADAM21,ACTN1,ACTB,ACMSD,ACKR2,ACAN,ABHD17C,ABCA4,ABCA2*

Supplementary Table 22 Functional enrichment of genes with CDS intersecting with CPC- specific-AIS

Database	ID	Description	GeneRatio	BgRatio	Odds ratio	p.adjust	qvalue
BP	GO:0052697	xenobiotic glucuronidation	9/1040	11/18800	77.51	1.22E-06	1.17E-06
BP	GO:0009812	flavonoid metabolic process	10/1040	16/18800	28.73	3.98E-06	3.80E-06
BP	GO:0052695	cellular glucuronidation	9/1040	20/18800	14.09	0.000791	0.000754
BP	GO:0006063	uronic acid metabolic process	9/1040	22/18800	11.92	0.001271	0.001211
BP	GO:0019585	glucuronate metabolic process	9/1040	22/18800	11.92	0.001271	0.001211
BP	GO:0007612	learning	23/1040	145/18800	3.27	0.0041	0.00391
BP	GO:0008306	associative learning	16/1040	80/18800	4.32	0.004868	0.004642
BP	GO:0050954	sensory perception of mechanical stimulus	25/1040	177/18800	2.85	0.00943	0.008992
BP	GO:0007605	sensory perception of sound	23/1040	156/18800	3.00	0.00943	0.008992
BP	GO:0061458	reproductive system development	45/1040	436/18800	2.01	0.022613	0.021561
BP	GO:0048608	reproductive structure development	44/1040	433/18800	1.97	0.035878	0.034209
BP	GO:0051928	positive regulation of calcium ion transport	18/1040	119/18800	3.08	0.040846	0.038946
CC	GO:0005938	cell cortex	37/1107	310/19594	2.31	0.009247	0.008447
CC	GO:0016324	apical plasma membrane	39/1107	358/19594	2.08	0.021721	0.019842
CC	GO:0045177	apical part of cell	43/1107	424/19594	1.92	0.025284	0.023096
CC	GO:0000145	exocyst	6/1107	16/19594	10.07	0.025284	0.023096
MF	GO:0015020	glucuronosyltransferase activity	10/1062	34/18410	6.86	0.013533	0.012885
MF	GO:0016758	hexosyltransferase activity	27/1062	198/18410	2.62	0.013533	0.012885
MF	GO:0015267	channel activity	50/1062	489/18410	1.90	0.015408	0.014672
MF	GO:0022803	passive transmembrane transporter activity	50/1062	490/18410	1.90	0.015408	0.014672
MF	GO:0008194	UDP-glycosyltransferase activity	21/1062	144/18410	2.83	0.015408	0.014672
MF	GO:0046873	metal ion transmembrane transporter activity	44/1062	428/18410	1.91	0.023747	0.022611
MF	GO:0005216	ion channel activity	44/1062	442/18410	1.84	0.035199	0.033516
MF	GO:0002039	p53 binding	12/1062	66/18410	3.66	0.035199	0.033516
MF	GO:0044325	transmembrane transporter binding	18/1062	127/18410	2.73	0.035199	0.033516
MF	GO:0099106	ion channel regulator activity	19/1062	138/18410	2.64	0.035199	0.033516
MF	GO:0016757	glycosyltransferase activity	30/1062	271/18410	2.06	0.0419	0.039896
MF	GO:0016247	channel regulator activity	19/1062	143/18410	2.53	0.046097	0.043892
KEGG	hsa00053	Ascorbate and aldarate metabolism	10/454	30/8164	8.66	0.000888	0.000805
KEGG	hsa00040	Pentose and glucuronate interconversions	10/454	35/8164	6.92	0.00211	0.001911
KEGG	hsa00982	Drug metabolism - cytochrome P450	13/454	72/8164	3.82	0.014998	0.013589
KEGG	hsa00983	Drug metabolism - other enzymes	13/454	80/8164	3.36	0.025621	0.023214
KEGG	hsa00140	Steroid hormone biosynthesis	11/454	61/8164	3.80	0.025621	0.023214
KEGG	hsa00860	Porphyrin metabolism	9/454	43/8164	4.57	0.025621	0.023214
KEGG	hsa04610	Complement and coagulation cascades	13/454	85/8164	3.13	0.034615	0.031363
KEGG	hsa00980	Metabolism of xenobiotics by cytochrome P450	12/454	78/8164	3.14	0.042856	0.03883
KEGG	hsa05131	Shigellosis	26/454	247/8164	2.06	0.042856	0.03883
KEGG	hsa05204	Chemical carcinogenesis - DNA adducts	11/454	69/8164	3.28	0.042856	0.03883

Note: GeneRatio denotes the ratio of input genes that are annotated in a term, and BgRatio denotes the ratio of all genes that are annotated in a term. The *P*-values are obtained by one-sided Fisher's exact test. They are further adjusted for multiple comparisons using the Benjamini-Hochberg procedure (denoted as the BH-adjusted *P*-value), and are also adjusted for the false discovery rate (denoted as the q-value).

Supplementary Table 23 GeneAnalytics annotation of genes with CDS intersecting with CPC-specific-AIS

Score	Disease	Matched Genes	Matched Genes with Genetic Associations
61.02	Colorectal Cancer	*AKT1, CCND1, CTNNB1, DCC, FANCC, FANCI, FGFR2, PKHD1, SLC9A9, TP53, UHRF1, BEST4, MTRF1, SCGB2A2, ACTB, CASP9, CCL20, CD4, CDCP1, CDKN1B, CLU, CYP24A1, DLL4, FES, FURIN, FZD10, GRP, HIF1A, HK2, INSR, KRT19, LGALS1, LRP5, MAPK13, MAPK14, MAPK8, MDM2, MIF, MMP11, MRE11, MTA1, NFKB1, NRP2, NUAK1, POLE2, PPARD, SAPCD2, SEPTIN9, SLC2A1, SLIT2, SPARC, TGFB2, THBS1, TIAM1, TMEFF2, TOP1, TPBG, TYMP, UGT1A1, UGT1A6, UGT1A7, UGT1A9, VDR, WNT5B, FCRLA, FUT7, GPATCH2, LGI4, SLC11A1, UNC5C, ACAN, BICD1, CAB39L, CCDC150, CD200, CLEC12B, COL1A1, CORO2B, DENND5B, F13A1, GAL, KAZN, LHX9, LORICRIN, MAP4K2, MFAP4, NPAS3, NRROS, OLFML2B, RGS1, SLC6A6, SULT1C2, SZT2, VWF, ZBTB8B*	*AKT1, CCND1, CTNNB1, DCC, FANCC, FANCI, FGFR2, PKHD1, SLC9A9, TP53, UHRF1*
60.18	Breast Cancer	*ABCA1, AKT1, AOPEP, FANCC, FANCM, FGFR2, MRE11, SF3B1, SLC22A18, TP53, KRT19, ASPM, KRT15, SCGB1D2, SCGB2A2, ACTB, ATR, CASP9, CCDC170, CCND1, CCNT2, CDKN1B, CEBPB, CLU, CTNNB1, CYP24A1, DLL4, GNRH1, GPATCH2, GREB1, HIF1A, HMGA1, INSR, LGALS1, LRP5, MAPK14, MAPK4, MAPK7, MAPK8, MDM2, MMP11, MMP17, MTA1, MTUS1, NCOR2, NFKB1, NR3C2, NRP2, NTRK2, PAXIP1, PHLDA2, PLCG1, PPP5C, PRMT7, PTPRG, RABL6, RELB, SLC2A1, SPARC, SUSD2, TAB1, TGFB2, THBS1, TOP1, TPD52L1, TSC1, TYMP, UHRF1, VDR, WNT5B, BCAT1, CHL1, COL1A1, CYP4F8, DMKN, FNDC3A, FSIP1, KLRD1, MGME1, MLLT10, OLFML2B, PDE7B, PNP, PPP6R3, QSER1, RAI14, RIDA, RPH3AL, ULK4, VAPB, WLS*	*ABCA1, AKT1, AOPEP, FANCC, FANCM, FGFR2, MRE11, SF3B1, SLC22A18, TP53, KRT19*
47.78	Schizophrenia	*CNTN6, CSMD1, CTNND2, DLG2, DPP6, HECW2, IRAK1BP1, LPP, NPAS3, PHIP, PRKN, RELN, RTN4R, ULK4, ZNF804A, DISC1, GABRB2, AKT1, APP, CHL1, CNIH3, CSMD2, CTNNB1, DLGAP1, DRD1, DRD5, EGR3, GABRA5, GABRB3, GRIK3, GRIN1, GRM4, GRM7, ITIH3, LRRTM1, NBPF3, NOS1AP, NTNG1, NTRK2, PAH, PPP1R9B, SLC2A1, SNAP25, ST8SIA2, TBP, TP53, TSNAX-DISC1, UHMK1, VIPR2, ZDHHC8*	*CNTN6, CSMD1, CTNND2, DLG2, DPP6, HECW2, IRAK1BP1, LPP, NPAS3, PHIP, PRKN, RELN, RTN4R, ULK4, ZNF804A, DISC1, GABRB2*
47.08	Nervous System Disease	*ARSA, ASPM, CLN6, CTNNB1, GNAO1, HECW2, HSD17B4, PNKP, POLG, RMND1, SRD5A3, TMEM216, ABCA1, ABCA4, ACTB, AKT1, APP, ATP12A, ATP4A, C3, CASP9, CCL3, CCND1, CD19, CD4, CDKN1B, CFH, CFHR2, CHL1, CLU, CRB1, ELAVL4, GJB6, GRIN1, HIF1A, HTT, IFNAR1, ITIH4, ITPR1, MAP3K5, MAPK14, MAPK8, MDM2, MERTK, NTRK2, PRKN, SCN8A, SLC2A1, SMARCB1, SNAP25, SORL1, TBP, TGFB2, THBS1, TP53, TRPV1, VDAC1, VWF*	*ARSA, ASPM, CLN6, CTNNB1, GNAO1, HECW2, HSD17B4, PNKP, POLG, RMND1, SRD5A3, TMEM216*
44.04	Lung Cancer	*AKT1, CTNNB1, FGFR2, PRKN, SLC22A18, TP53, CLIC5, GJB6, KRT15, PEBP4, SLC2A1, ARHGAP5, CASP9, CAVIN3, CCND1, CD9, CDCP1, CDKN1B, CDKN1C, COL4A2, DDR2, EFNB3, ELAVL4, EPB41L3, FGL1, GRP, HIF1A, HK2, IFNAR1, ITGA11, KRT19, LGALS1, MAPK14, MAPK8, MDM2, MIF, NFKB1, PLCG1, SCGB1A1, SCUBE3, SEZ6L, SLFN11, SPARC, SRPK1, THBS1, TOP1, TYMP, UGT1A1, VDAC1, ASPM, CDC20, SUSD2, TCF21, CAB39L, CCN5, COL1A1, CRIP1, CXCL13, FANCI, FGR, FXYD1, GPX3, KIF2C, MFAP4, MMP11, SLC39A8, UHRF1*	*AKT1, CTNNB1, FGFR2, PRKN, SLC22A18, TP53*
40.55	Alzheimer Disease, Familial, 1	*APP, UNC5C, ANKS1B, CAMKK2, DYNC1I1, GALNT17, KCNIP4, PAQR9, ABCA1, ABCA2, ACTB, AKT1, BACE2, CASP4, CASP9, CCL3, CFH, CLU, CTNND2, DHCR24, DRD1, FURIN, GAL, GRIN1, HTT, INSR, ITIH4, LRP2, MAP3K5, MAPK14, MAPK8, NCF2, NTRK2, P2RX7, PRKN, RELN, RTN4R, SLC5A7, SNAP25, SORL1, TBP, VDAC1, CAPRIN2, TNS1, AHNAK, CATSPERE, CRMP1, CSMD2, DDX46, DENND5B, DGCR8, FXYD1, FZD10, GPR158, ITGA11, ITGB2, ITIH3, KCNQ1, LRRTM1, NRP2, NTNG1, SLC2A1, TBC1D16, TPD52L1*	*APP, UNC5C*
40.52	Retinitis Pigmentosa	*ABCA4, CLRN1, CNGB3, CRB1, EYS, MERTK, PCARE, SNRNP200, ACTB, AKT1, APP, B9D1, CCND1, CEP164, CEP83, CFH, CLU, CNGA3, CNTLN, CTNNB1, CYP24A1, DNAAF2, DNAH5, ETV6, GABARAPL1, GJB6, GRK1, HIF1A, HTT, IFT43, IFT74, KLHL3, MDM2, MKKS, PKHD1, POLG, PRKN, RDH10, RPGRIP1L, SCLT1, SLC2A1, SLC6A6, SRD5A3, TBP, TMEM138, TMEM216, TMPRSS2, TP53, TRNT1, TSC1, ULK1, ZBTB8B*	*ABCA4, CLRN1, CNGB3, CRB1, EYS, MERTK, PCARE, SNRNP200*

Supplementary Table 24 Population-specific archaic introgressed segments in CPC (absent in HPRC) with size >150 kb.

Chr	Start	End	Frequency	Gene	Length	Population	Language Family
chr1	12274919	12604575	1	*VPS13D*	329656	Jingpo	Sino-Tibetan
chr1	31377957	31546044	1	*PUM1,SDC3*	168087	Bouyei	Tai-Kadai
chr1	59103130	59314469	1	*JUN,MYSM1*	211339	Zhuang	Tai-Kadai
chr1	160163303	160318092	1	*CASQ1,COPA,DCAF8,ENSG00000258465,NCSTN,PEA15,PEX19*	154789	CHS	Sino-Tibetan
chr1	165243851	165399810	1	*LMX1A,RXRG*	155959	Kazakh	Turkic
chr1	192135732	192348622	1	*RGS18,RGS21*	212890	Chosen	Koreanic
chr1	197215963	197932974	1	*C1orf53,CRB1,DENND1B,LHX9*	717011	She	Hmong-Mien
chr1	205676212	205896475	1	*NUCKS1,PM20D1,RAB29,SLC26A9,SLC41A1*	220263	Evenki	Tungusic
chr1	211900267	212165143	1	*INTS7,LPGAT1*	264876	Dong	Tai-Kadai
chr1	215178483	215535767	1	*KCNK2*	357284	Blang	Austro-Asiatic
chr1	217625505	218024114	1	*GPATCH2,SPATA17*	398609	Uyghur	Turkic
chr1	220155228	220530808	1	*BPNT1,EPRS1,IARS2,RAB3GAP2*	375580	Daur	Mongolic
chr1	222976514	223174846	1	*DISP1*	198332	Miao	Hmong-Mien
chr1	225067640	225505529	1	*DNAH14*	437889	Uyghur	Turkic
chr1	225801052	225954461	1	*ENAH*	153409	Deang	Austro-Asiatic
chr1	230539447	230694552	1	*PGBD5*	155105	Kyrgyz	Turkic
chr2	1903416	2222912	2	*MYT1L*	319496	Kyrgyz	Turkic
chr2	37338314	37596434	1	*CEBPZ,CEBPZOS,EIF2AK2,NDUFAF7,PRKD3,QPCT,SULT6B1*	258120	Uyghur	Turkic
chr2	55772688	56119899	1	*EFEMP1,PNPT1,PPP4R3B*	347211	Daur	Mongolic
chr2	56242634	56488624	1	*CCDC85A*	245990	Evenki	Tungusic
chr2	68639717	68870535	1	*APLF,FBXO48*	230818	Chosen	Koreanic
chr2	74783324	74969763	1	*DOK1,M1AP,SEMA4F*	186439	Mosuo	Sino-Tibetan
chr2	99286592	99451547	1	*CRACDL,MGAT4A*	164955	Evenki	Tungusic
chr2	99554531	100179357	1	*AFF3,C2orf15,EIF5B,ENSG00000241962,ENSG00000273155,LIPT1,LYG1,LYG2,MITD1,MRPL30,REV1,TSGA10,TXNDC9*	624826	Kazakh	Turkic
chr2	138885495	139391644	1	*SPOPL*	506149	Salar	Turkic
chr2	163597938	163836150	1	*KCNH7*	238212	Yi	Sino-Tibetan
chr2	172527229	172858214	1	*DYNC1I2,HAT1,SLC25A12*	330985	Miao	Hmong-Mien
chr2	186256354	186728959	1	*FSIP2*	472605	Lisu	Sino-Tibetan
chr2	198493703	198866362	1	*BOLL,MARS2,PLCL1,RFTN2*	372659	Man	Tungusic
chr2	223604192	223964153	1	*ACSL3,KCNE4*	359961	Tujia	Sino-Tibetan
chr3	9988502	10150755	1	*EMC3,FANCD2,FANCD2OS,PRRT3*	162253	Jino	Sino-Tibetan
chr3	12635224	12858557	1	*CAND2,RAF1,TMEM40*	223333	Jingpo	Sino-Tibetan
chr3	15529944	15717421	1	*ANKRD28,BTD,COLQ,HACL1*	187477	Dong	Tai-Kadai
chr3	17215272	17824490	1	*TBC1D5*	609218	Tu	Turkic
chr3	46711507	46866164	1	*ALS2CL,ENSG00000206549,PRSS50,TMIE*	154657	Yugur	Turkic
chr3	50430486	51840034	1	*C3orf18,CACNA2D2,CISH,DCAF1,DOCK3,GRM2,HEMK1,IQCF6,MANF,MAPKAPK3,RAD54L2,RBM15B,TEX264*	1409548	CHS	Sino-Tibetan
chr3	56532890	56739732	1	*CCDC66,TASOR*	206842	Kinh	Austro-Asiatic
chr3	119824963	120148805	1	*FSTL1,GPR156,LRRC58*	323842	Tujia	Sino-Tibetan
chr3	123524676	123704070	1	*CCDC14,MYLK,ROPN1*	179394	Dong	Tai-Kadai
chr3	132930743	133110681	1	*TMEM108*	179938	Kyrgyz	Turkic
chr3	141988438	142554469	1	*ATR,PCOLCE2,PLS1,TRPC1,XRN1*	566031	Achang	Sino-Tibetan
chr3	157630686	158542747	1	*GFM1,LXN,MFSD1,MLF1,RARRES1,RSRC1,SHOX2*	912061	Daur	Mongolic
chr3	169027133	169193421	1	*MECOM*	166288	Qiang	Sino-Tibetan
chr3	196769113	197040486	1	*DLG1*	271373	Yao	Hmong-Mien
chr4	9967255	10134929	1	*SLC2A9,WDR1*	167674	Kinh	Austro-Asiatic
chr4	20086222	20319549	1	*SLIT2*	233327	Chosen	Koreanic
chr4	26878844	27048542	2	*STIM2*	169698	Mosuo	Sino-Tibetan
chr4	30623248	31002392	2	*PCDH7*	379144	Lisu	Sino-Tibetan
chr4	81311243	81945451	1	*CFAP299*	634208	Uyghur	Turkic
chr4	103136669	103594918	1	*MANBA,NFKB1,SLC39A8*	458249	Wa	Austro-Asiatic
chr4	118998756	119245388	1	*NDST3,PRSS12*	246632	Mongol	Mongolic
chr4	123141070	123771473	1	*ADAD1,BBS12,FGF2,IL21,IL2,KIAA1109*	630403	Man	Tungusic
chr4	129723224	130135654	1	*C4orf33,JADE1,SCLT1*	412430	Zhuang	Tai-Kadai
chr4	145663798	145895241	1	*ANAPC10*	231443	Jingpo	Sino-Tibetan
chr4	164611270	164780098	1	*MARCHF1*	168828	Miao	Hmong-Mien
chr4	167975361	168231772	1	*SPOCK3*	256411	She	Hmong-Mien
chr4	169787542	169943358	1	*CBR4,PALLD*	155816	Hui	Sino-Tibetan
chr4	174108796	174265404	1	*GALNT7,HMGB2*	156608	She	Hmong-Mien
chr5	19767512	19921853	1	*CDH18*	154341	Miao	Hmong-Mien
chr5	53529539	53685087	1	*ARL15*	155548	Daur	Mongolic
chr5	54887450	55219194	1	*DDX4,IL31RA,SLC38A9*	331744	Kazakh	Turkic
chr5	59804300	60089436	1	*DEPDC1B,ELOVL7*	285136	Tu	Turkic
chr5	118154461	118318909	1	*DTWD2*	164448	Chosen	Koreanic
chr5	118393619	118604876	1	*DMXL1,TNFAIP8*	211257	Chosen	Koreanic
chr5	122437278	122647811	1	*PRDM6*	210533	Khatso	Sino-Tibetan
chr5	131628880	131813204	2	*IRF1,P4HA2,SLC22A4,SLC22A5*	184324	Wa	Austro-Asiatic
chr5	133975295	134212652	1	*C5orf24,CAMLG,DDX46,SEC24A,TXNDC15*	237357	Wa	Austro-Asiatic
chr6	496761	649113	1	*EXOC2*	152352	Salar	Turkic
chr6	24206948	24418348	1	*DCDC2,KAAG1,MRS2*	211400	Tujia	Sino-Tibetan

Chr	Start	End	Frequency	Gene	Length	Population	Language Family
chr6	27090305	27745557	1	*H2AC11,H2AC12,H2BC11,H2BC12,H4C9,POM121L2,PRSS16,ZNF184,ZNF391*	655252	Uyghur	Turkic
chr6	27782538	28679411	1	*ENSG00000276302,GPX5,GPX6,H1-5,H2AC15,H2AC16,H2AC17,H2BC14,H2BC15,H2BC17,H3C11,H3C12,H4C11,H4C12,H4C13,NKAPL,OR2B2,OR2B6,OR2B8P,PGBD1,ZBED9,ZKSCAN3,ZKSCAN4,ZKSCAN8,ZKSCAN8P1,ZNF165,ZSCAN12,ZSCAN16,ZSCAN23,ZSCAN26,ZSCAN31,ZSCAN9*	896873	Uyghur	Turkic
chr6	30367317	30718035	1	*ABCF1,ATAT1,C6orf136,DHX16,FLOT1,GNL1,HLA-E,IER3,MDC1,MRPS18B,NRM,PPP1R10,PPP1R18,PRR3,TUBB*	350718	Deang	Austro-Asiatic
chr6	34214670	34434670	1	*NUDT3,RPS10,RPS10-NUDT3,SMIM29*	220000	Bai	Sino-Tibetan
chr6	43251880	43427850	1	*ABCC10,CRIP3,DLK2,SLC22A7,TTBK1,ZNF318*	175970	Achang	Sino-Tibetan
chr6	46728894	46948534	1	*ADGRF5,MEP1A*	219640	Deang	Austro-Asiatic
chr6	70812175	71037500	1	*COL19A1,COL9A1*	225325	Yugur	Turkic
chr6	79541997	79864569	1	*IRAK1BP1,PHIP*	322572	Tujia	Sino-Tibetan
chr6	82881958	83228380	1	*IBTK,TPBG*	346422	Mongol	Mongolic
chr6	83605949	83761723	1	*UBE3D*	155774	Kazakh	Turkic
chr6	89324517	89700752	1	*RNGTT*	376235	Jino	Sino-Tibetan
chr6	99702599	99895392	1	*COQ3,FAXC,PNISR,USP45*	192793	Kazakh	Turkic
chr6	116489338	116704697	1	*NT5DC1,TSPYL1,TSPYL4*	215359	Uyghur	Turkic
chr6	122407176	122936177	1	*HSF2,SERINC1*	529001	Dong	Tai-Kadai
chr6	157551986	157874278	1	*TMEM242,ZDHHC14*	322292	Salar	Turkic
chr6	163038044	163279762	1	*PACRG,PRKN*	241718	Wa	Austro-Asiatic
chr6	170081378	170249806	1	*C6orf120,DYNLT2,ENSG00000285733,ERMARD,PHF10,WDR27*	168428	Daur	Mongolic
chr7	7169147	7350683	1	*C1GALT1*	181536	Uyghur	Turkic
chr7	17782423	17982646	1	*SNX13*	200223	Tu	Turkic
chr7	43619877	43903540	1	*BLVRA,COA1,STK17A*	283663	Mongol	Mongolic
chr7	51098849	51376659	1	*COBL*	277810	Miao	Hmong-Mien
chr7	54409069	54688696	1	*VSTM2A*	279627	Hui	Sino-Tibetan
chr7	94411719	94753588	1	*PPP1R9A*	341869	Salar	Turkic
chr7	96623278	96821078	1	*DLX5,DLX6,SDHAF3*	197800	Dong	Tai-Kadai
chr7	103652872	103816500	1	*ORC5*	163628	Miao	Hmong-Mien
chr7	128100038	128382855	1	*GARIN1A,GARIN1B,METTL2B*	282817	Tujia	Sino-Tibetan
chr7	135139041	135458941	1	*FAM180A,NUP205,SLC13A4,STMP1*	319900	Jino	Sino-Tibetan
chr7	146831849	147009311	1	*CNTNAP2*	177462	Kazakh	Turkic
chr8	32440257	32700343	1	*ENSG00000286131,NRG1*	260086	Salar	Turkic
chr8	87789872	88008773	1	*CNBD1*	218901	Yugur	Turkic
chr8	97633865	98028108	1	*CPQ*	394243	Drung	Sino-Tibetan
chr8	98631047	98804839	1	*LAPTM4B,MTDH*	173792	She	Hmong-Mien
chr8	104467252	105016502	1	*ENSG00000285982,RIMS2*	549250	Jino	Sino-Tibetan
chr8	105188195	105390852	1	*DCSTAMP,RIMS2*	202657	Jino	Sino-Tibetan
chr9	33784908	34429075	1	*C9orf24,DCAF12,FAM219A,KIF24,MYORG,NUDT2,PRSS3,UBAP1,UBAP2,UBE2R2*	644167	Hui	Sino-Tibetan
chr9	88474522	88670451	1	*GOLM1,NAA35*	195929	CHS	Sino-Tibetan
chr9	91775958	92000257	1	*CKS2,SECISBP2,SEMA4D,SHC3*	224299	Kyrgyz	Turkic
chr9	131137018	131300442	1	*CERCAM,GLE1,ODF2,URM1*	163424	Blang	Austro-Asiatic
chr9	135628768	135833461	1	*AK8,SPACA9,TSC1*	204693	Bouyei	Tai-Kadai
chr10	7827942	8015038	1	*ATP5F1C,KIN,TAF3*	187096	Qiang	Sino-Tibetan
chr10	21653034	21871622	1	*MLLT10,SKIDA1*	218588	Daur	Mongolic
chr10	23064897	23263058	1	*ARMC3*	198161	Zhuang	Tai-Kadai
chr10	24861862	25055660	1	*ARHGAP21*	193798	Tujia	Sino-Tibetan
chr10	32677575	33317783	1	*CCDC7,ITGB1*	640208	Hui	Sino-Tibetan
chr10	44017399	44176295	1	*ZNF239,ZNF32,ZNF485*	158896	Oroqen	Tungusic
chr10	88092138	88329724	1	*GRID1,WAPL*	237586	Daur	Mongolic
chr10	97742082	97969694	1	*BLNK,CC2D2B,CCNJ,ZNF518A*	227612	Hui	Sino-Tibetan
chr10	111600477	111913847	1	*ADD3,XPNPEP1*	313370	Mongol	Mongolic
chr10	134302618	134522203	1	*INPP5A*	219585	Achang	Sino-Tibetan
chr11	32644303	33048786	1	*CCDC73,DEPDC7,PRRG4,QSER1*	404483	Kyrgyz	Turkic
chr11	57695412	57862593	1	*OR6Q1*	167181	Zhuang	Tai-Kadai
chr11	59249745	59629067	1	*CBLIF,MRPL16,OR10V1,OR4D11,OR4D9,OSBP,PATL1,STX3,TCN1*	379322	Bai	Sino-Tibetan
chr11	60925066	61254024	1	*CPSF7,CYB561A3,DDB1,ENSG00000256591,PGA3,PGA4,PGA5,PPP1R32,SDHAF2,TKFC,TMEM138,TMEM216,VWCE*	328958	Kyrgyz	Turkic
chr11	68163456	68598407	1	*CPT1A,GAL,LRP5,PPP6R3,TESMIN*	434951	Mosuo	Sino-Tibetan
chr11	71575564	71842976	1	*ANAPC15,DEFB131B,IL18BP,LAMTOR1,LRRC51,LRTOMT,NUMA1,RNF121,TOMT,XNDC1N,XNDC1N-ZNF705EP-ALG1L9P*	267412	Daur	Mongolic
chr11	83718264	83983546	1	*DLG2*	265282	Tu	Turkic
chr11	85001672	85193570	1	*DLG2*	191898	Uyghur	Turkic
chr11	85206156	85406915	1	*CCDC89,CREBZF,DLG2,SYTL2,TMEM126A,TMEM126B*	200759	Uyghur	Turkic
chr11	85623619	85874244	1	*CCDC83,PICALM*	250625	Hui	Sino-Tibetan
chr11	107062975	107223013	1	*CWF19L2*	160038	Mongol	Mongolic
chr11	110482610	110663752	1	*ARHGAP20*	181142	Miao	Hmong-Mien
chr12	28238905	28741422	1	*CCDC91*	502517	Kyrgyz	Turkic
chr12	32304351	32481369	1	*BICD1*	177018	Mongol	Mongolic
chr12	44046046	44317153	1	*IRAK4,PUS7L,TMEM117,TWF1*	271107	Hezhen	Tungusic
chr12	44997302	45159998	1	*NELL2*	162696	Mosuo	Sino-Tibetan
chr12	48965055	49158490	1	*CCNT1,KANSL2,TEX49*	193435	Mongol	Mongolic

Chr	Start	End	Frequency	Gene	Length	Population	Language Family
chr12	50338036	50501566	1	*AQP2,AQP5,AQP6,ASIC1,GPD1,RACGAP1,SMARCD1*	163530	Kinh	Austro-Asiatic
chr12	56071317	56239848	1	*BLOC1S1,CD63,DNAJC14,ENSG00000257390,ENSG00000258311,GDF11,ITGA7,METTL7B,MMP19,ORMDL2,RDH5,SARNP*	168531	Oroqen	Tungusic
chr12	58007678	58511607	2	*AGAP2,ARHGEF25,ATP23,AVIL,B4GALNT1,CDK4,CTDSP2,CYP27B1,EEF1AKMT3,ENSG00000257921,ENSG00000287908,MARCHF9,METTL1,OS9,TSFM,TSPAN31*	503929	Blang	Austro-Asiatic
chr12	60063350	60257517	1	*SLC16A7*	194167	Uyghur	Turkic
chr12	63684107	64028097	1	*DPY19L2*	343990	Salar	Turkic
chr12	69007464	69269996	1	*CPM,MDM2,NUP107,RAP1B,SLC35E3*	262532	Salar	Turkic
chr12	69526680	69684617	1	*CPSF6*	157937	Yugur	Turkic
chr12	69851714	70101362	1	*BEST3,CCT2,FRS2,LRRC10*	249648	Salar	Turkic
chr12	70384464	70816663	1	*CNOT2,KCNMB4*	432199	Yugur	Turkic
chr12	76695981	77099421	1	*BBS10,OSBPL8*	403440	Lisu	Sino-Tibetan
chr12	95358327	95689373	1	*FGD6,NDUFA12,NR2C1,VEZT*	331046	Yao	Hmong-Mien
chr12	120907332	121388962	1	*ACADS,CABP1,COQ5,DYNLL1,ENSG00000288623,MLEC,POP5,RNF10,SPPL3,SRSF9,UNC119B*	481630	Yugur	Turkic
chr13	19988041	20234519	1	*MPHOSPH8,TPTE2*	246478	Blang	Austro-Asiatic
chr13	21290348	21519686	1	*EEF1AKMT1,IL17D,XPO4*	229338	Wa	Austro-Asiatic
chr13	27094377	27365541	1	*GPR12,WASF3*	271164	Yao	Hmong-Mien
chr13	27957831	28111997	1	*GTF3A,MTIF3*	154166	Yugur	Turkic
chr13	42154926	42578472	1	*VWA8*	423546	Tu	Turkic
chr13	46907369	47059573	1	*RUBCNL*	152204	Achang	Sino-Tibetan
chr13	73329489	73635568	1	*DIS3,KLF5,PIBF1*	306079	Daur	Mongolic
chr13	79854198	80027197	1	*RBM26*	172999	CHS	Sino-Tibetan
chr13	99878888	100038293	1	*GPR18,GPR183,UBAC2*	159405	Yugur	Turkic
chr13	114318259	114469073	1	*TMEM255B*	150814	Mosuo	Sino-Tibetan
chr14	32022637	32319562	1	*NUBPL*	296925	Daur	Mongolic
chr14	35387860	35822193	1	*ENSG00000258790,FAM177A1,PPP2R3C,PRORP,PSMA6,SRP54*	434333	Uyghur	Turkic
chr14	37719911	38285940	1	*FOXA1,MIPOL1,TTC6*	566029	Kazakh	Turkic
chr14	44802420	45012946	1	*FSCB*	210526	Daur	Mongolic
chr14	45368137	45628750	1	*C14orf28,FANCM,FKBP3,KLHL28,PRPF39,TOGARAM1*	260613	Jino	Sino-Tibetan
chr14	47019736	47367938	1	*MDGA2,RPL10L*	348202	Drung	Sino-Tibetan
chr14	47743566	48050906	1	*MDGA2*	307340	Kyrgyz	Turkic
chr14	50876861	51182989	1	*ATL1,MAP4K5,SAV1*	306128	Yugur	Turkic
chr14	55587391	55838108	1	*ATG14,DLGAP5,FBXO34,LGALS3*	250717	Yugur	Turkic
chr14	68325879	68709884	1	*RAD51B*	384005	Salar	Turkic
chr14	70078934	70325708	1	*SLC10A1,SRSF5,SUSD6*	246774	Yi	Sino-Tibetan
chr14	70749545	70976904	1	*ADAM21,COX16,SYNJ2BP,SYNJ2BP-COX16*	227359	Mosuo	Sino-Tibetan
chr15	39732018	39936774	1	*FSIP1,THBS1*	204756	Tu	Turkic
chr15	39983727	40136088	1	*FSIP1,GPR176*	152361	Tu	Turkic
chr15	41136182	41459786	1	*CHAC1,DLL4,INO80,RHOV,SPINT1,VPS18*	323604	Uyghur	Turkic
chr15	50765310	51051044	1	*ENSG00000288645,SPPL2A,TRPM7,USP50,USP8*	285734	Blang	Austro-Asiatic
chr15	63356446	63636878	1	*APH1B,CA12,LACTB,RAB8B,RPS27L,TPM1*	280432	Tujia	Sino-Tibetan
chr15	64339953	65108479	1	*CIAO2A,CSNK1G1,ENSG00000259316,OAZ2,PCLAF,PIF1,PPIB,RBPMS2,SNX1,SNX22,TRIP4,ZNF609*	768526	Deang	Austro-Asiatic
chr15	67678140	68047073	1	*C15orf61,IQCH,MAP2K5*	368933	Yugur	Turkic
chr16	25240437	25504351	1	*ZKSCAN2*	263914	Salar	Turkic
chr17	3184361	3400692	1	*ASPA,OR1E1,OR1E2,OR1R1P,OR3A1,SPATA22*	216331	She	Hmong-Mien
chr17	75864641	76109073	1	*TNRC6C*	244432	Wa	Austro-Asiatic
chr18	47266084	47735555	1	*ACAA2,ENSG00000266997,MYO5B*	469471	Khatso	Sino-Tibetan
chr18	50271715	50561613	1	*DCC*	289898	Hezhen	Tungusic
chr18	59866906	60021761	1	*RELCH,TNFRSF11A*	154855	Yugur	Turkic
chr18	74466187	74694135	1	*MBP,ZNF236*	227948	Achang	Sino-Tibetan
chr19	34730145	34997102	1	*ENSG00000266953,GARRE1,GPI,PDCD2L,UBA2,WTIP*	266957	Salar	Turkic
chr19	52340926	52502535	1	*ZNF350,ZNF577,ZNF613,ZNF615,ZNF649*	161609	Kazakh	Turkic
chr20	13908548	14272150	1	*MACROD2,SEL1L2*	363602	Mongol	Mongolic
chr20	14358105	14508363	1	*MACROD2*	150258	Evenki	Tungusic
chr20	14542687	14696882	1	*MACROD2*	154195	Evenki	Tungusic
chr20	39733170	40261659	1	*CHD6,EMILIN3,LPIN3,PLCG1,TOP1,ZHX3*	528489	Achang	Sino-Tibetan
chr21	44895972	45177719	1	*H2BC12L,HSF2BP,PDXK,RRP1B*	281747	Man	Tungusic
chr22	24666643	24836123	1	*ADORA2A,SPECC1L,SPECC1L-ADORA2A*	169480	Kazakh	Turkic

Supplementary Table 25 Summary of the population-specific archaic introgressed segments in CPC (absent in HPRC).

Population	AIS Length(Mb)	AIS P roportion (%)	AIS Number	Language Family
Kinh	4.02	0.14	146	Austro-Asiatic
Deang	5.71	0.20	181	Austro-Asiatic
Wa	5.87	0.20	173	Austro-Asiatic
Blang	6.29	0.22	173	Austro-Asiatic
Yao	1.86	0.06	39	Hmong-Mien
She	5.55	0.19	164	Hmong-Mien
Miao	6.81	0.24	119	Hmong-Mien
Chosen	5.90	0.20	163	Koreanic
Daur	8.18	0.28	165	Mongolic
Mongol	9.05	0.31	253	Mongolic
Naxi	1.03	0.04	57	Sino-Tibetan
Yi	1.93	0.07	50	Sino-Tibetan
Bai	2.19	0.08	65	Sino-Tibetan
Khatso	2.51	0.09	56	Sino-Tibetan
Drung	2.55	0.09	53	Sino-Tibetan
Jingpo	2.73	0.09	70	Sino-Tibetan
Lisu	2.86	0.10	65	Sino-Tibetan
Achang	3.46	0.12	69	Sino-Tibetan
Jino	3.71	0.13	80	Sino-Tibetan
Hui	4.96	0.17	91	Sino-Tibetan
Qiang	5.37	0.19	174	Sino-Tibetan
CHS	6.01	0.21	167	Sino-Tibetan
Tujia	6.52	0.23	144	Sino-Tibetan
Mosuo	8.14	0.28	206	Sino-Tibetan
Bouyei	2.07	0.07	66	Tai-Kadai
Zhuang	2.50	0.09	56	Tai-Kadai
Dong	4.80	0.17	113	Tai-Kadai
Hezhen	2.25	0.08	71	Tungusic
Oroqen	2.36	0.08	63	Tungusic
Man	2.92	0.10	68	Tungusic
Evenki	3.97	0.14	98	Tungusic
Tu	7.51	0.26	181	Turkic
Kyrgyz	8.21	0.28	167	Turkic
Kazakh	9.04	0.31	187	Turkic
Yugur	9.61	0.33	196	Turkic
Salar	10.08	0.35	201	Turkic
Uyghur	14.68	0.51	245	Turkic

Supplementary Table 26 CPC-specific SV-related genes affected by the population-specific archaic introgressed segments in CPC (absent in HPRC).

Gene	AIS (bp)	% AIS	Gene	AIS (bp)	% AIS	Gene	AIS (bp)	% AIS	Gene	AIS (bp)	% AIS
MACROD2	1661282	0.057663	*NRXN3*	1570549	0.054514	*CTNNA3*	1456162	0.050543	*TBC1D5*	1287655	0.044694
ANKS1B	1225998	0.042554	*LINGO2*	1197169	0.041554	*CNTN5*	1021258	0.035448	*EYS*	897618	0.031156
MGAT4C	883236	0.030657	*RAD51B*	880038	0.030546	*DLG2*	877998	0.030475	*MARCHF1*	842647	0.029248
MAGI2	818519	0.028411	*AGBL1*	785482	0.027264	*KAZN*	760153	0.026385	*RIMS2*	753820	0.026165
USH2A	723654	0.025118	*ULK4*	715532	0.024836	*CPNE4*	709588	0.024630	*DOCK3*	709271	0.024619
NELL1	693401	0.024068	*PDE4D*	670939	0.023288	*DCC*	661103	0.022947	*PRKN*	646154	0.022428
PTPRN2	634722	0.022031	*MDGA2*	629637	0.021855	*CSMD2*	594702	0.020642	*CDH18*	592892	0.020579
LHFPL3	579957	0.020130	*CFAP299*	573661	0.019912	*UTRN*	567699	0.019705	*UNC13C*	565463	0.019627
SLC4A10	560949	0.019470	*KCNIP4*	560207	0.019445	*KCNQ5*	554147	0.019234	*DAB1*	535534	0.018588
FAF1	523239	0.018162	*CTNND2*	517376	0.017958	*LUZP2*	510077	0.017705	*TENM3*	508317	0.017644
CDH12	504000	0.017494	*DNAH14*	503030	0.017460	*PPP2R2B*	501337	0.017401	*CCSER1*	497109	0.017255
TMEM132D	495063	0.017184	*COL25A1*	494027	0.017148	*CACNA2D3*	480300	0.016671	*CPQ*	479679	0.016650
PDE11A	471778	0.016375	*KCNH7*	467360	0.016222	*GRIK2*	467353	0.016222	*SOX5*	463603	0.016092
ITPR2	462235	0.016044	*KCNH1*	457149	0.015868	*HECW1*	453372	0.015737	*TMEM117*	450943	0.015652
CCDC91	448262	0.015559	*KALRN*	440668	0.015296	*RSRC1*	439875	0.015268	*CCDC7*	436734	0.015159
TRPM3	436238	0.015142	*NLGN1*	434101	0.015068	*BTBD9*	433124	0.015034	*RUNX1*	430782	0.014952
ESRRG	428743	0.014882	*DOK6*	428348	0.014868	*ESR1*	428218	0.014863	*COL24A1*	423565	0.014702
NALF1	420370	0.014591	*CNTN4*	420098	0.014582	*THSD7B*	415640	0.014427	*ROBO2*	412818	0.014329
SPOCK3	409965	0.014230	*GRM1*	409955	0.014230	*OSBPL10*	404508	0.014040	*SPATA5*	396363	0.013758
SHANK2	393689	0.013665	*CFAP54*	385984	0.013397	*ADAMTSL3*	385754	0.013389	*SGCZ*	385516	0.013381
VWA8	385478	0.013380	*CACNA1D*	384200	0.013336	*DSCAML1*	383677	0.013317	*SUGCT*	383261	0.013303
NTRK3	382753	0.013285	*WDR7*	376264	0.013060	*MYO5B*	372375	0.012925	*XIRP2*	371273	0.012887
SLC35F4	371099	0.012881	*DYNC2H1*	370452	0.012858	*AKAP13*	368787	0.012801	*FRMD4A*	361588	0.012551
ASTN2	361469	0.012547	*ELMO1*	361179	0.012537	*PCDH9*	357503	0.012409	*NAV2*	356526	0.012375
NUBPL	355556	0.012341	*JMJD1C*	354629	0.012309	*PCDH7*	353371	0.012265	*RNGTT*	348820	0.012108
ADGRB3	346190	0.012016	*DGKB*	345277	0.011985	*CAMK2D*	343873	0.011936	*NFIA*	342234	0.011879
LRMDA	336064	0.011665	*CDH23*	335353	0.011640	*PRIM2*	333773	0.011585	*IQCJ-SCHIP1*	333156	0.011564
SEMA3A	332859	0.011554	*DNAH7*	331134	0.011494	*FOCAD*	329999	0.011454	*CDC42BPA*	328627	0.011407
PDZD2	328540	0.011404	*FANCC*	326874	0.011346	*CCDC102B*	324266	0.011255	*NEK11*	323615	0.011233
MLIP	321607	0.011163	*SYN3*	320847	0.011137	*MYT1L*	319496	0.011090	*CERS6*	318885	0.011068
NELL2	318329	0.011049	*PDZRN4*	318097	0.011041	*AOPEP*	318068	0.011040	*FSTL5*	317284	0.011013
KSR2	316651	0.010991	*DNAH8*	315481	0.010950	*PLCH1*	312290	0.010840	*GRM7*	310495	0.010777
MSR1	309604	0.010746	*NRG1*	309578	0.010745	*RALGPS1*	308392	0.010704	*PDSS2*	307002	0.010656
ARHGAP42	306653	0.010644	*TTN*	304813	0.010580	*CACNA2D1*	303655	0.010540	*WWOX*	301387	0.010461
COBL	300606	0.010434	*NTRK2*	300287	0.010423	*GHR*	298439	0.010359	*HHAT*	298201	0.010351
CENPP	294576	0.010225	*GALNT13*	292621	0.010157	*SYNPR*	291708	0.010125	*EYA4*	291614	0.010122
BTBD11	289827	0.010060	*SRPK2*	288604	0.010017	*PCNX2*	287432	0.009980	*MYCBP2*	284620	0.009880
SPATA13	283995	0.009860	*B3GALT1*	283694	0.009850	*VPS13D*	282012	0.009790	*PIGN*	281863	0.009780
ALK	281152	0.009760	*AIG1*	280562	0.009740	*SASH1*	279548	0.009700	*SLC35F3*	279216	0.009690
RAB3C	277173	0.009620	*BICD1*	276798	0.009610	*MEI4*	276771	0.009610	*XRCC4*	276289	0.009590
TSPAN8	275857	0.009570	*RTN1*	274800	0.009540	*TNR*	274242	0.009520	*SLC30A10*	273591	0.009500
TMTC1	272366	0.009450	*DENND1B*	270948	0.009400	*UNC79*	270123	0.009380	*GALNT17*	270005	0.009370
FHIT	269984	0.009370	*FRMD3*	267930	0.009300	*SPECC1*	265937	0.009230	*LDLRAD3*	265629	0.009220
ATP2B2	264701	0.009190	*MAP2K5*	264414	0.009180	*CLYBL*	261062	0.009060	*PCSK2*	258471	0.008970
C12orf42	258387	0.008970	*PACRG*	257795	0.008950	*DLG1*	256770	0.008910	*FMN1*	255733	0.008880
TBC1D32	255251	0.008860	*SIK3*	255035	0.008850	*PDE1A*	254135	0.008820	*TP63*	253640	0.008800
PLD5	253287	0.008790	*RBFOX1*	249973	0.008680	*WDR72*	249137	0.008650	*MAST2*	249135	0.008650
TBC1D1	248091	0.008610	*PDE7B*	246599	0.008560	*IQCH*	246573	0.008560	*TAFA1*	246155	0.008540
TYW1	244341	0.008480	*PPP1R12B*	244007	0.008470	*ANK2*	242892	0.008430	*CNTNAP5*	241575	0.008390
KLF12	241566	0.008380	*ZBTB20*	240899	0.008360	*ATRNL1*	237411	0.008240	*NCALD*	237286	0.008240
LRFN5	237219	0.008230	*TRABD2B*	236857	0.008220	*NME7*	235417	0.008170	*BAZ2B*	234352	0.008130
PIBF1	234328	0.008130	*CNIH3*	233806	0.008120	*SIPA1L2*	232606	0.008070	*NOS1AP*	231376	0.008030
SCLT1	228679	0.007940	*SLC2A9*	226087	0.007850	*ZNF609*	225490	0.007830	*HERC1*	225330	0.007820
HTR4	224528	0.007790	*NCOR2*	224102	0.007780	*EPB41L2*	223975	0.007770	*FNDC3A*	223600	0.007760
SUMF1	223580	0.007760	*ANKRD11*	222931	0.007740	*SGCD*	222802	0.007730	*GRIK4*	222357	0.007720
PLXNA2	222142	0.007710	*MYO5A*	221851	0.007700	*WWC2*	220766	0.007660	*SPATA17*	220284	0.007650
PBX3	220032	0.007640	*GALNT18*	219217	0.007610	*SLC25A12*	218299	0.007580	*PHACTR2*	218261	0.007580
AGBL4	217845	0.007560	*TIAM1*	217585	0.007550	*SCMH1*	215104	0.007470	*ROR2*	214283	0.007440
FAM107B	214217	0.007440	*RGS6*	214037	0.007430	*NHSL1*	213397	0.007410	*VPS53*	213214	0.007400
VWA3B	213052	0.007390	*LRP1B*	212869	0.007390	*DPP6*	212055	0.007360	*MYO16*	212002	0.007360
DMXL1	211366	0.007340	*CRYBG1*	211300	0.007330	*CSMD1*	210953	0.007320	*DYNC1I1*	210615	0.007310
LDB2	209178	0.007260	*DENND5B*	208910	0.007250	*OSBPL8*	208012	0.007220	*RIMBP2*	207990	0.007220
SLC25A21	207549	0.007200	*DGKH*	206967	0.007180	*PRKCE*	205706	0.007140	*NPAS3*	205421	0.007130
TFDP2	205116	0.007120	*SVEP1*	204563	0.007100	*PREX1*	203746	0.007070	*GRK5*	203010	0.007050
XXYLT1	202875	0.007040	*DLGAP1*	202147	0.007020	*TEX9*	202026	0.007010	*FILIP1*	201941	0.007010
LDLRAD4	200726	0.006970	*TAF3*	198134	0.006880	*JAKMIP2*	197337	0.006850	*PLCL1*	197045	0.006840
GABRB2	195774	0.006800	*LMNTD1*	194769	0.006760	*SPIRE1*	194215	0.006740	*CNTNAP2*	194138	0.006740
GRB10	192263	0.006670	*VAT1L*	191543	0.006650	*PRDM16*	190389	0.006610	*FAM184A*	189624	0.006580
DNAH11	188299	0.006540	*GPC5*	188040	0.006530	*PAPPA*	187341	0.006500	*DSE*	187236	0.006500
DOCK9	186365	0.006470	*PDE3A*	185197	0.006430	*KCNQ1*	185172	0.006430	*CDH20*	183745	0.006380
RGL1	183350	0.006360	*DAAM1*	182759	0.006340	*KIAA1958*	182570	0.006340	*OSBPL6*	182484	0.006330
WDR27	181042	0.006280	*NDST3*	181033	0.006280	*SLCO1B3-SLCO1B7*	180609	0.006270	*MYO1B*	180204	0.006250
SLC39A8	180178	0.006250	*ATRN*	180100	0.006250	*SEMA3C*	179821	0.006240	*RFX4*	179799	0.006240
PTPRG	178932	0.006210	*GPATCH2*	178927	0.006210	*CTNNA2*	178884	0.006210	*GAS7*	178141	0.006180
MUC19	177437	0.006160	*SPATA6*	176835	0.006140	*NPAS2*	176690	0.006130	*RELN*	176003	0.006110
HSD17B12	175937	0.006110	*AGMO*	175665	0.006100	*WDR49*	175299	0.006080	*STAB2*	175160	0.006080

Gene	AIS (bp)	% AIS	Gene	AIS (bp)	% AIS	Gene	AIS (bp)	% AIS	Gene	AIS (bp)	% AIS
DMXL2	175122	0.006080	*AGPS*	174278	0.006050	*KMT2E*	174076	0.006040	*C10orf90*	174017	0.006040
EVL	172787	0.006000	*TCF4*	172536	0.005990	*SLC35F1*	172504	0.005990	*FLT1*	172027	0.005970
PPP2R5E	172013	0.005970	*CCDC73*	171905	0.005970	*SOBP*	171200	0.005940	*ADGRA3*	170995	0.005940
INPP5A	170912	0.005930	*SYNPO2*	170324	0.005910	*NMNAT2*	170143	0.005910	*GARNL3*	169395	0.005880
ACAP2	168299	0.005840	*DOCK2*	168272	0.005840	*MED12L*	168264	0.005840	*PHLDB2*	167477	0.005810
PRMT8	167273	0.005810	*CNTNAP4*	166864	0.005790	*ENAH*	166377	0.005770	*ATP2C1*	166117	0.005770
CDH13	165890	0.005760	*PTPRA*	165656	0.005750	*PLXNA4*	163722	0.005680	*SLC2A13*	162004	0.005620
LARP1B	161645	0.005610	*CAMTA1*	161292	0.005600	*SLCO3A1*	159943	0.005550	*EXOC2*	158969	0.005520
SVIL	158758	0.005510	*UBASH3B*	158751	0.005510	*ARHGEF10L*	158176	0.005490	*RFX7*	157802	0.005480
CRACD	157700	0.005470	*NR3C1*	157581	0.005470	*NTM*	156708	0.005440	*EXT2*	156314	0.005430
DDR2	156032	0.005420	*RIN3*	155893	0.005410	*CERT1*	155793	0.005410	*SRGAP3*	155571	0.005400
TRIM44	155234	0.005390	*AGAP1*	154947	0.005380	*LARGE1*	154855	0.005370	*LMX1A*	154848	0.005370
GPC6	154711	0.005370	*PPP6R3*	154603	0.005370	*LIMS1*	153575	0.005330	*CEP83*	153539	0.005330
ARHGEF12	153524	0.005330	*MAPK4*	153493	0.005330	*ELOVL6*	153353	0.005320	*RGS3*	153012	0.005310
DLC1	152953	0.005310	*KLC1*	152353	0.005290	*FAM184B*	152315	0.005290	*KDM2B*	152029	0.005280
CCBE1	151808	0.005270	*IMPG1*	151564	0.005260	*DCDC2*	151339	0.005250	*SLC24A4*	151178	0.005250
FGD4	150408	0.005220	*ZNF236*	150344	0.005220	*GPR141*	150021	0.005210	*GRIN2B*	149844	0.005200
SNX13	149739	0.005200	*RBM19*	149587	0.005190	*AP2B1*	148367	0.005150	*DAPK1*	148182	0.005140
STIM2	148159	0.005140	*SLC38A9*	147349	0.005110	*SEL1L2*	147196	0.005110	*SPECC1L*	146922	0.005100
NRF1	145361	0.005050	*STOX2*	144787	0.005030	*CERS3*	144600	0.005020	*BACH2*	144576	0.005020
SH3RF2	144396	0.005010	*RMDN2*	143955	0.005000	*PHIP*	143868	0.004990	*EBF2*	143860	0.004990
NTNG1	143538	0.004980	*TIAM2*	143183	0.004970	*SEMA6D*	142345	0.004940	*KCNJ6*	141950	0.004930
OBSCN	141949	0.004930	*CACNA2D2*	141631	0.004920	*RPS10-NUDT3*	141184	0.004900	*RUNX2*	140779	0.004890
FGD6	140733	0.004880	*SLC16A7*	140268	0.004870	*TMEM163*	140166	0.004870	*PRR5L*	139579	0.004840
TECPR2	139536	0.004840	*ZHX3*	139283	0.004830	*CNOT4*	139247	0.004830	*FAT1*	138928	0.004820
TPTE2	138749	0.004820	*RAI14*	138743	0.004820	*ROS1*	138589	0.004810	*FARP2*	138556	0.004810
MATN2	138058	0.004790	*PRDM5*	137824	0.004780	*HIBADH*	137441	0.004770	*INO80*	137400	0.004770
SEMA4D	137343	0.004770	*PRKCQ*	137245	0.004760	*LRP5*	136684	0.004740	*DLG5*	135247	0.004690
ANO3	135018	0.004690	*LARP1*	134619	0.004670	*DRAM1*	134550	0.004670	*PUM1*	134485	0.004670
PCSK6	134443	0.004670	*TRPM7*	134324	0.004660	*ERI3*	134190	0.004660	*CHFR*	134117	0.004660
MYRFL	133870	0.004650	*GALNTL6*	133849	0.004650	*LIFR*	133686	0.004640	*COL28A1*	133294	0.004630
LY75-CD302	132859	0.004610	*CDC73*	132832	0.004610	*CD109*	132532	0.004600	*LSAMP*	132363	0.004590
UPP2	132301	0.004590	*DST*	131976	0.004580	*PRTG*	131608	0.004570	*GLT1D1*	131541	0.004570
WASF3	131265	0.004560	*PPP4R4*	130980	0.004550	*EP400*	130518	0.004530	*HSF2BP*	130303	0.004520
CNBD1	130130	0.004520	*AUTS2*	130004	0.004510	*RAD54L2*	129948	0.004510	*ATR*	129726	0.004500
PRKCA	129688	0.004500	*ELF1*	129516	0.004500	*RABGEF1*	129300	0.004490	*CRACR2A*	128887	0.004470
WDR64	128695	0.004470	*ZSCAN5A*	128667	0.004470	*MCUB*	128510	0.004460	*TESK2*	128343	0.004450
DYSF	128261	0.004450	*JAZF1*	128216	0.004450	*ACYP2*	128060	0.004440	*PTPRM*	127721	0.004430
NOL10	127640	0.004430	*UNC5C*	127086	0.004410	*SLIT3*	125817	0.004370	*AK8*	125396	0.004350
RGS7	125380	0.004350	*STRN3*	125339	0.004350	*TENM2*	125288	0.004350	*RIPOR2*	124964	0.004340
KCTD16	124921	0.004340	*PPARGC1B*	124724	0.004330	*PLAGL1*	124299	0.004310	*RAB3GAP2*	124236	0.004310
TDRD9	124211	0.004310	*REEP1*	124090	0.004310	*ECPAS*	124060	0.004310	*UPF2*	123148	0.004270
RELCH	122994	0.004270	*PTPN3*	122847	0.004260	*CSRNP3*	122400	0.004250	*UBE2H*	122228	0.004240
ST18	122154	0.004240	*COL12A1*	121941	0.004230	*TXNDC16*	121926	0.004230	*PRMT3*	121780	0.004230
FIGN	121659	0.004220	*GLI3*	121317	0.004210	*COA1*	121261	0.004210	*DOCK1*	121243	0.004210
ATP8B1	121187	0.004210	*BTD*	120970	0.004200	*USP47*	120464	0.004180	*NALCN*	119853	0.004160
LPP	119828	0.004160	*LGR6*	119818	0.004160	*STPG2*	119452	0.004150	*C6*	119322	0.004140
GRK1	119238	0.004140	*METTL8*	119056	0.004130	*PTK2B*	118998	0.004130	*ATP10B*	118623	0.004120
MARK3	118454	0.004110	*UST*	118222	0.004100	*TSPEAR*	118118	0.004100	*LRFN2*	118007	0.004100
TMOD3	117667	0.004080	*JRKL*	117585	0.004080	*ATP2B4*	117294	0.004070	*TMEM179*	116953	0.004060
STXBP1	116657	0.004050	*KIRREL3*	116613	0.004050	*LIN52*	116537	0.004040	*FAM120B*	116364	0.004040
NEB	116140	0.004030	*TACC2*	116066	0.004030	*PSD3*	115811	0.004020	*RNF17*	115752	0.004020
ARHGEF3	115546	0.004010	*CRTC3*	115422	0.004010	*CRYL1*	114993	0.003990	*PPP1R13B*	114380	0.003970
BORCS5	114155	0.003960	*ZNF678*	113900	0.003950	*IGSF21*	113761	0.003950	*ADAMTS18*	113317	0.003930
NUDT3	112990	0.003920	*PDE4B*	112827	0.003920	*CD163L1*	112369	0.003900	*TOGARAM1*	112241	0.003900
NWD2	112027	0.003890	*ABCB11*	111907	0.003880	*FOXN3*	111858	0.003880	*ERBB4*	111652	0.003880
MUSK	111039	0.003850	*FOXO1*	110974	0.003850	*ZDHHC20*	110586	0.003840	*TMEM63C*	110456	0.003830
CNTN1	110164	0.003820	*ARHGAP25*	110098	0.003820	*TDRD12*	109827	0.003810	*RFTN1*	109825	0.003810
FAM78B	109575	0.003800	*FRS2*	109444	0.003800	*NDUFA12*	108451	0.003760	*MTAP*	108378	0.003760
STK32B	108103	0.003750	*RPS6KA2*	107676	0.003740	*BAZ1A*	107546	0.003730	*PHC2*	107472	0.003730
ANKRD30A	106969	0.003710	*ZNF717*	106923	0.003710	*PEPD*	106760	0.003710	*TMEFF2*	106670	0.003700
TBX15	106510	0.003700	*HK1*	106439	0.003690	*BRF1*	106303	0.003690	*ZNF365*	106096	0.003680
SEPTIN9	105710	0.003670	*FBXO42*	105640	0.003670	*MAML2*	105309	0.003660	*OR3A2*	105250	0.003650
TAX1BP1	105233	0.003650	*SFRP4*	105104	0.003650	*NNT*	104713	0.003630	*NRIP1*	104701	0.003630
GRAMD1C	104487	0.003630	*CABCOCO1*	103837	0.003600	*PSMD14*	103292	0.003590	*DEPDC1B*	103278	0.003580
INPP5F	103105	0.003580	*LPIN1*	102966	0.003570	*CHL1*	102868	0.003570	*DDX60*	102592	0.003560
EPHB2	102480	0.003560	*ADGRF5*	102421	0.003560	*EFCAB13*	102140	0.003550	*RCAN1*	101972	0.003540
RIMKLB	101495	0.003520	*LY75*	101401	0.003520	*AJAP1*	101018	0.003510	*CASZ1*	100637	0.003490
GLB1	100593	0.003490	*TMOD1*	100563	0.003490	*QSER1*	100505	0.003490	*ANK3*	100501	0.003490
CHODL	100275	0.003480	*MEGF11*	100217	0.003480	*DYNC2I1*	100189	0.003480	*PGR*	100189	0.003480
CXCL13	100081	0.003470	*TNFAIP8*	100056	0.003470	*OSMR*	99738	0.003460	*CRY1*	99438	0.003450
ESYT2	99258	0.003450	*PRKCB*	99132	0.003440	*HHIP*	99115	0.003440	*GALNT9*	99089	0.003440
GPR176	98930	0.003430	*FBXO34*	98709	0.003430	*TNC*	98682	0.003430	*PAMR1*	98478	0.003420
CHRM2	97951	0.003400	*IRAG2*	97939	0.003400	*PLEKHG4B*	97804	0.003390	*ZBTB40*	97573	0.003390
MFSD1	97521	0.003380	*DPH6*	97296	0.003380	*SNED1*	96948	0.003370	*ACOT11*	96935	0.003360
FER	96864	0.003360	*ADGRL2*	96554	0.003350	*ZNF83*	96444	0.003350	*DDX46*	96362	0.003340
CEP89	96066	0.003330	*NECTIN1*	95898	0.003330	*SYK*	95884	0.003330	*EVA1C*	95704	0.003320
SLIT2	95552	0.003320	*ITGA11*	95389	0.003310	*PRTFDC1*	95199	0.003300	*PGAP1*	94792	0.003290
RFX2	94789	0.003290	*RBM26*	94653	0.003290	*ZGRF1*	94532	0.003280	*MGAM*	94130	0.003270
PACS2	94004	0.003260	*CEP43*	93929	0.003260	*ITPKB*	93448	0.003240	*KIF26B*	93344	0.003240
AKAP12	93002	0.003230	*LZTFL1*	92726	0.003220	*SIDT1*	91972	0.003190	*C1GALT1*	91682	0.003180
PEX7	91370	0.003170	*NUP205*	90840	0.003150	*MME*	90714	0.003150	*NR1H4*	90706	0.003150
PCBD2	90688	0.003150	*PTCHD4*	90565	0.003140	*SEC63*	90452	0.003140	*UNC5B*	90294	0.003130
MCPH1	90260	0.003130	*ARL15*	90154	0.003130	*U2SURP*	89813	0.003120	*HDLBP*	89797	0.003120

Gene	AIS (bp)	% AIS	Gene	AIS (bp)	% AIS	Gene	AIS (bp)	% AIS	Gene	AIS (bp)	% AIS
ANKMY1	89787	0.003120	*POLK*	89388	0.003100	*C4orf19*	89313	0.003100	*AGFG1*	89065	0.003090
NIM1K	88779	0.003080	*CMTM8*	88512	0.003070	*STK32C*	88221	0.003060	*ALDH1L2*	87859	0.003050
USP3	87725	0.003040	*RPGRIP1L*	87582	0.003040	*PRKCZ*	87577	0.003040	*TTC27*	87178	0.003030
SNX27	87085	0.003020	*CROCC2*	86981	0.003020	*TNIK*	86771	0.003010	*SCGB2B2*	86515	0.003000
RHPN2	86296	0.003000	*LRP8*	86105	0.002990	*MTDH*	86076	0.002990	*SLC22A3*	85791	0.002980
OPCML	85765	0.002980	*EBF3*	85714	0.002980	*LHFPL6*	85591	0.002970	*GNPTAB*	85460	0.002970
CLIC5	85017	0.002950	*CCDC60*	84896	0.002950	*UBXN7*	84768	0.002940	*SP3*	84523	0.002930
RAB3IP	84344	0.002930	*TDRD7*	84029	0.002920	*DENND2B*	83955	0.002910	*TRPC1*	83854	0.002910
REPS1	83839	0.002910	*ACSL3*	83697	0.002910	*TP73*	83685	0.002900	*F13A1*	83441	0.002900
POU2F3	83305	0.002890	*TANC1*	83231	0.002890	*TPCN2*	82958	0.002880	*USP53*	82931	0.002880
RAG1	82784	0.002870	*CPT1A*	82765	0.002870	*KLHL23*	82501	0.002860	*MTMR7*	82482	0.002860
UMAD1	82207	0.002850	*HDDC2*	82116	0.002850	*KDM4C*	82049	0.002850	*STK38L*	81991	0.002850
SLC22A2	81854	0.002840	*ELOVL5*	81781	0.002840	*ORC5*	81707	0.002840	*DAP*	81667	0.002830
ZNF248	81580	0.002830	*ATL2*	81415	0.002830	*NT5DC1*	81322	0.002820	*YAF2*	81245	0.002820
SPTAN1	81104	0.002820	*B4GALT5*	80933	0.002810	*GRAMD2B*	80825	0.002810	*TGFA*	80768	0.002800
JPH2	80598	0.002800	*IL17RD*	80324	0.002790	*ITGAE*	80035	0.002780	*HS3ST2*	79982	0.002780
SH3GL2	79973	0.002780	*ARSJ*	79443	0.002760	*SPC25*	79239	0.002750	*SEC24A*	79124	0.002750
LARS2	79003	0.002740	*NOSTRIN*	78975	0.002740	*CHSY3*	78878	0.002740	*LIN9*	78720	0.002730
ST20-MTHFS	78604	0.002730	*ARPIN-AP3S2*	78574	0.002730	*CRMP1*	78162	0.002710	*EPRS1*	77930	0.002700
IBTK	77768	0.002700	*RFX8*	77753	0.002700	*USP28*	77698	0.002700	*ADARB2*	77328	0.002680
CCM2	76994	0.002670	*HIVEP2*	76966	0.002670	*COL27A1*	76869	0.002670	*DNAH9*	76809	0.002670
CHSY1	76321	0.002650	*RELL1*	76311	0.002650	*GNB5*	76292	0.002650	*MRE11*	76255	0.002650
GPM6A	75899	0.002630	*EPHA7*	75654	0.002630	*ZNF518A*	75572	0.002620	*FANCD2*	75562	0.002620
GLT8D2	75196	0.002610	*ME2*	75177	0.002610	*CCDC148*	74948	0.002600	*CTDP1*	74700	0.002590
FBXO15	74490	0.002590	*FAP*	74467	0.002580	*ACOX3*	74441	0.002580	*RBM47*	74411	0.002580
SLC39A11	74263	0.002580	*ATXN7L1*	74181	0.002570	*TNXB*	74181	0.002570	*ABCB5*	74155	0.002570
ST3GAL4	74084	0.002570	*KCNAB1*	73846	0.002560	*SPATA22*	73833	0.002560	*CHST11*	73714	0.002560
NIBAN2	73650	0.002560	*AHRR*	73640	0.002560	*NCAPG2*	73634	0.002560	*IRAK2*	73486	0.002550
GLYATL1	73367	0.002550	*FANCI*	73316	0.002540	*RASGEF1A*	72530	0.002520	*TIMP2*	72410	0.002510
NDST1	72392	0.002510	*TPD52L1*	72336	0.002510	*SH3BGRL2*	72326	0.002510	*LARS1*	72130	0.002500
ZDHHC14	72113	0.002500	*SGSM1*	72099	0.002500	*MOV10L1*	71811	0.002490	*LTBP1*	71752	0.002490
COLQ	71618	0.002490	*SORBS2*	71544	0.002480	*FAM177A1*	71513	0.002480	*IL31RA*	71497	0.002480
TMTC4	71468	0.002480	*FAM83B*	71324	0.002480	*CFI*	71142	0.002470	*RFLNA*	71119	0.002470
CDYL	71111	0.002470	*FBRSL1*	71109	0.002470	*SLC38A1*	70921	0.002460	*TARS3*	70877	0.002460
NT5DC3	70781	0.002460	*NEMF*	70738	0.002460	*RAF1*	70476	0.002450	*ADGRV1*	70272	0.002440
SLC1A3	70216	0.002440	*TSPAN11*	70175	0.002440	*DISC1*	70174	0.002440	*PRKAA2*	70021	0.002430
PCSK5	70019	0.002430	*PIEZO1*	69882	0.002430	*ST6GAL1*	69787	0.002420	*SLC5A1*	69768	0.002420
KATNBL1	69422	0.002410	*LMF1*	69124	0.002400	*CPB1*	69085	0.002400	*TNIP3*	68931	0.002390
RRBP1	68617	0.002380	*ADCY10*	68546	0.002380	*CTNNBL1*	68475	0.002380	*MAST4*	68475	0.002380
CFAP97D2	68394	0.002370	*NAALADL2*	68308	0.002370	*RASA3*	68306	0.002370	*CD226*	68193	0.002370
IL1RAP	68173	0.002370	*LEMD1*	68125	0.002360	*ACSF3*	68001	0.002360	*PIK3R4*	67895	0.002360
GPR137C	67359	0.002340	*SPG7*	67047	0.002330	*ZNF521*	67015	0.002330	*HSD17B3*	66870	0.002320
IQSEC3	66735	0.002320	*SLAIN1*	66555	0.002310	*ADCY9*	66395	0.002300	*DAPK2*	66286	0.002300
FNBP1	66081	0.002290	*TP53BP2*	66061	0.002290	*NSL1*	65629	0.002280	*SKAP2*	65605	0.002280
DNAJC16	65566	0.002280	*PPP6R2*	65546	0.002280	*SLC28A3*	65301	0.002270	*FGF2*	65132	0.002260
SLC17A8	64981	0.002260	*FANCM*	64960	0.002250	*ADTRP*	64809	0.002250	*TMOD2*	64766	0.002250
STIL	64753	0.002250	*PEDS1-UBE2V1*	64529	0.002240	*PTPRR*	64420	0.002240	*ACTN1*	64392	0.002240
SZT2	64365	0.002230	*MAPK8IP3*	64185	0.002230	*VIPR2*	64174	0.002230	*JAKMIP3*	64143	0.002230
NXN	64128	0.002230	*SDHAF3*	64065	0.002220	*AP3S2*	64039	0.002220	*WDR25*	63994	0.002220
EIF5B	63955	0.002220	*MTHFS*	63794	0.002210	*GABRG3*	63703	0.002210	*ITPR1*	63646	0.002210
POLE	63623	0.002210	*TMCO3*	63569	0.002210	*AP3D1*	63480	0.002200	*NCAM2*	63231	0.002190
ANKS1A	63210	0.002190	*CWF19L2*	63208	0.002190	*SERPINI1*	63074	0.002190	*GPD1L*	63020	0.002190
SMOC2	62861	0.002180	*ZNF318*	62513	0.002170	*NRDC*	62273	0.002160	*DOP1B*	61881	0.002150
VAPB	61872	0.002150	*NBL1*	61476	0.002130	*KCNQ2*	61274	0.002130	*SLC28A1*	61144	0.002120
MEGF6	61141	0.002120	*CAMKK2*	60633	0.002100	*SLC17A5*	60613	0.002100	*FKBP15*	60452	0.002100
FREM2	60391	0.002100	*GCC2*	60308	0.002090	*AKNA*	60249	0.002090	*GOLGA3*	60241	0.002090
NKAIN1	60142	0.002090	*KLHDC1*	60045	0.002080	*MICOS10-NBL1*	59859	0.002080	*PNPT1*	59802	0.002080
DGKI	59531	0.002070	*PATJ*	59349	0.002060	*HSP90AA1*	58957	0.002050	*TMEM120B*	58955	0.002050
NUP107	58858	0.002040	*ADAMTS14*	58752	0.002040	*RAP1B*	58217	0.002020	*EFEMP1*	58172	0.002020
LNPEP	57893	0.002010	*EVC*	57785	0.002010	*NLRP5*	57563	0.002000	*ZBTB8A*	57526	0.002000
TRMT44	57391	0.001990	*SCARA5*	57180	0.001980	*STXBP6*	57099	0.001980	*C6orf89*	57094	0.001980
PPIG	57064	0.001980	*MAPK10*	57033	0.001980	*KCNMB4*	56886	0.001970	*NAF1*	56817	0.001970
LRRK1	56816	0.001970	*TMEM132C*	56788	0.001970	*LSM14A*	56784	0.001970	*TFDP1*	56752	0.001970
MYSM1	56650	0.001970	*OSBPL1A*	56503	0.001960	*FUT8*	56488	0.001960	*SACM1L*	56353	0.001960
IPP	56329	0.001960	*ZNF827*	56292	0.001950	*CAMSAP1*	56063	0.001950	*PRRX2*	55995	0.001940
ASRGL1	55964	0.001940	*MLLT1*	55945	0.001940	*GRK7*	55944	0.001940	*CD82*	55931	0.001940
ONECUT2	55924	0.001940	*CPNE2*	55786	0.001940	*MCM9*	55481	0.001930	*LHFPL4*	55461	0.001930
CALN1	55407	0.001920	*SLC36A1*	55333	0.001920	*TAF15*	55133	0.001910	*ACMSD*	55123	0.001910
FUT10	55075	0.001910	*ZNF713*	55074	0.001910	*GABRA5*	55051	0.001910	*SLC5A8*	54745	0.001900
SLC6A6	54615	0.001900	*C14orf132*	54609	0.001900	*SPTB*	54593	0.001890	*BRD1*	54590	0.001890
AAGAB	54528	0.001890	*PKHD1*	54428	0.001890	*SYNE2*	54309	0.001890	*WNT3A*	54273	0.001880
PLA2G4A	54101	0.001880	*USP36*	54060	0.001880	*TMEM178B*	53964	0.001870	*IARS2*	53909	0.001870
CD200R1	53898	0.001870	*NKAIN2*	53403	0.001850	*NR2C1*	53399	0.001850	*PDK1*	53296	0.001850
DAW1	53290	0.001850	*ZNF804A*	53275	0.001850	*CACNA1I*	53229	0.001850	*RAB31*	53152	0.001840
KRT8	52767	0.001830	*THAP4*	52753	0.001830	*HIF1A*	52746	0.001830	*STON2*	52732	0.001830
USP50	52690	0.001830	*ZNF483*	52685	0.001830	*SLC6A3*	52646	0.001830	*GOLM1*	52550	0.001820
LMX1B	52470	0.001820	*STARD3NL*	52424	0.001820	*SNX1*	51967	0.001800	*SEC23IP*	51927	0.001800
IL2RA	51681	0.001790	*PDCD10*	51641	0.001790	*ARHGEF7*	51574	0.001790	*APOLD1*	51490	0.001790
EXOC1	51438	0.001790	*CYP2C9*	51433	0.001790	*CHURC1-FNTB*	51399	0.001780	*CAPN13*	51309	0.001780
RYR2	51225	0.001780	*SLC13A3*	51200	0.001780	*CYP4Z1*	51087	0.001770	*GFM1*	51054	0.001770
GMDS	50939	0.001770	*SYNE3*	50891	0.001770	*AGPAT4*	50881	0.001770	*AOAH*	50842	0.001760
PFKFB3	50656	0.001760	*STIM1*	50475	0.001750	*ATP1B3*	50425	0.001750	*MAGI1*	50310	0.001750

Gene	AIS (bp)	% AIS	Gene	AIS (bp)	% AIS	Gene	AIS (bp)	% AIS	Gene	AIS (bp)	% AIS
DHRS3	50277	0.001750	*BEGAIN*	50270	0.001740	*ADAMTS17*	50191	0.001740	*PTGR1*	50171	0.001740
ZNF124	50045	0.001740	*UGT3A1*	50022	0.001740	*TP53I11*	49953	0.001730	*LTF*	49588	0.001720
ACTA2	49347	0.001710	*RAB37*	49346	0.001710	*RHEX*	49315	0.001710	*CEP164*	49304	0.001710
CBX5	49191	0.001710	*LRP2*	49139	0.001710	*FAM174B*	49097	0.001700	*GRIK3*	49066	0.001700
CFAP210	48980	0.001700	*VWF*	48894	0.001700	*WDR1*	48883	0.001700	*BCAT1*	48766	0.001690
BLVRA	48662	0.001690	*EMC3*	48520	0.001680	*CCDC88C*	48465	0.001680	*GLRX3*	48130	0.001670
FLT4	48118	0.001670	*SEC14L1*	48118	0.001670	*SLC13A4*	48027	0.001670	*PARP1*	47944	0.001660
C2	47888	0.001660	*SLC35E3*	47839	0.001660	*SRP54*	47729	0.001660	*CYP19A1*	47576	0.001650
DHTKD1	47469	0.001650	*COQ8A*	47258	0.001640	*EXO1*	47181	0.001640	*RNF214*	47161	0.001640
PLCH2	47072	0.001630	*NUP37*	47017	0.001630	*EDIL3*	47001	0.001630	*ANKDD1A*	46932	0.001630
ACKR2	46791	0.001620	*ADCK1*	46719	0.001620	*EYA2*	46549	0.001620	*TTC39A*	46527	0.001610
CTXND1	46471	0.001610	*MEP1A*	46389	0.001610	*PAQR8*	46356	0.001610	*EIF2AK2*	46351	0.001610
ZNF487	46340	0.001610	*AXIN1*	46317	0.001610	*MTHFD1L*	46079	0.001600	*ZNF536*	46028	0.001600
POMT2	45927	0.001590	*SFMBT2*	45453	0.001580	*GABRB3*	45382	0.001580	*NLRP4*	45315	0.001570
LAMC2	45237	0.001570	*TCP11L2*	45074	0.001560	*PIP5K1C*	44973	0.001560	*DYNC1LI1*	44884	0.001560
TMPRSS4	44878	0.001560	*NBPF3*	44877	0.001560	*WDR89*	44832	0.001560	*CEP41*	44679	0.001550
EDRF1	44628	0.001550	*TCF7L1*	44581	0.001550	*FBXO21*	44573	0.001550	*CASP8AP2*	44542	0.001550
ZNF670	44174	0.001530	*PASK*	44165	0.001530	*TRPV1*	43967	0.001530	*WDR88*	43709	0.001520
ASB7	43564	0.001510	*PDCD6*	43353	0.001500	*POC5*	43324	0.001500	*GALNS*	43236	0.001500
ECM2	43101	0.001500	*ITIH2*	42988	0.001490	*GPI*	42933	0.001490	*SCARA3*	42908	0.001490
TM7SF3	42855	0.001490	*YARS1*	42794	0.001490	*PMP22*	42519	0.001480	*LRRC41*	42420	0.001470
CUL9	42403	0.001470	*SCTR*	42367	0.001470	*MAML3*	42340	0.001470	*METAP2*	42319	0.001470
ME1	42300	0.001470	*GRID2*	42258	0.001470	*S100PBP*	42108	0.001460	*NCKAP5*	42094	0.001460
HCFC2	41993	0.001460	*ZNF618*	41892	0.001450	*G3BP1*	41866	0.001450	*NPHP3*	41823	0.001450
C2orf76	41807	0.001450	*MLPH*	41724	0.001450	*LHPP*	41716	0.001450	*TEX264*	41630	0.001440
GRIP1	41598	0.001440	*DNAJC12*	41519	0.001440	*STX11*	41452	0.001440	*CNTN6*	41331	0.001430
NCAPH	41325	0.001430	*USP8*	41308	0.001430	*TTC23L*	41274	0.001430	*HABP4*	41167	0.001430
HACL1	41153	0.001430	*AHNAK2*	41130	0.001430	*ATPAF1*	41130	0.001430	*CCNT2*	41107	0.001430
SLC25A25	41044	0.001420	*DCAF12*	41012	0.001420	*CCDC82*	40715	0.001410	*DNAI2*	40650	0.001410
CENPJ	40610	0.001410	*KIF5A*	40607	0.001410	*LUC7L*	40494	0.001410	*NLRP11*	40236	0.001400
ZNF567	40089	0.001390	*TFRC*	40078	0.001390	*BCL2*	40075	0.001390	*GABRA1*	40061	0.001390
ZNF423	40049	0.001390	*FBXL17*	39938	0.001390	*RAB40C*	39915	0.001390	*ENG*	39645	0.001380
PROX1	39546	0.001370	*SCG3*	39534	0.001370	*GMEB2*	39499	0.001370	*FARP1*	39379	0.001370
TRPV3	39274	0.001360	*GLE1*	39219	0.001360	*RAB18*	39033	0.001350	*PTPRT*	38994	0.001350
PRKAA1	38985	0.001350	*SEPTIN2*	38927	0.001350	*TSPAN9*	38875	0.001350	*SLFN12L*	38790	0.001350
SDHA	38779	0.001350	*GPR107*	38777	0.001350	*PDXK*	38744	0.001340	*VSTM2B*	38557	0.001340
UGCG	38555	0.001340	*ATP13A4*	38550	0.001340	*SCLY*	38489	0.001340	*ZNF438*	38471	0.001340
SLC12A5	38460	0.001330	*OAS3*	38409	0.001330	*SMC3*	38364	0.001330	*ALDH1A3*	38246	0.001330
SULF2	38150	0.001320	*SEC61A2*	38116	0.001320	*YIPF1*	38079	0.001320	*SORCS2*	38068	0.001320
DAB2IP	38002	0.001320	*ZNF605*	38000	0.001320	*PTPA*	37996	0.001320	*KIF1A*	37955	0.001320
CDC16	37836	0.001310	*HGD*	37599	0.001310	*SPATS1*	37529	0.001300	*ANO7*	37467	0.001300
PLCXD2	37407	0.001300	*TMEM248*	37326	0.001300	*ZFP2*	37314	0.001300	*TAB1*	37282	0.001290
TEX49	37272	0.001290	*TNFSF8*	37252	0.001290	*SNRNP40*	37220	0.001290	*RASAL1*	37210	0.001290
RNF11	37174	0.001290	*URI1*	37108	0.001290	*KIN*	37031	0.001290	*PXDN*	36955	0.001280
ATG4B	36644	0.001270	*ANKRD10*	36529	0.001270	*RRP1B*	36519	0.001270	*SHISAL2A*	36515	0.001270
PON3	36503	0.001270	*CCDC32*	36371	0.001260	*ANKLE2*	36329	0.001260	*NETO1*	36236	0.001260
SH3BP4	36223	0.001260	*CRY2*	36130	0.001250	*DDX1*	36125	0.001250	*BRPF3*	36046	0.001250
DDX50	36030	0.001250	*CYBRD1*	35882	0.001250	*LEXM*	35765	0.001240	*CCL28*	35746	0.001240
SHC2	35690	0.001240	*NCK2*	35687	0.001240	*PEX14*	35645	0.001240	*PHF21B*	35631	0.001240
KLHL25	35599	0.001240	*RARRES1*	35597	0.001240	*MIGA2*	35461	0.001230	*CDH6*	35377	0.001230
FGGY	35266	0.001220	*NUS1*	35258	0.001220	*RNASET2*	35078	0.001220	*RORA*	35028	0.001220
ZNF439	35024	0.001220	*RNF168*	34985	0.001210	*GINS3*	34910	0.001210	*TGFBI*	34830	0.001210
PIWIL1	34750	0.001210	*CFHR5*	34668	0.001200	*TNFRSF10A*	34650	0.001200	*ARHGAP5*	34607	0.001200
TMEM242	34576	0.001200	*PTRH1*	34525	0.001200	*CAPSL*	34491	0.001200	*OTULINL*	34388	0.001190
SSBP3	34285	0.001190	*PRPF8*	34238	0.001190	*D2HGDH*	34214	0.001190	*PPP1R7*	34076	0.001180
ZBTB7C	34019	0.001180	*TNS1*	34014	0.001180	*MTUS1*	33933	0.001180	*IRS2*	33888	0.001180
TTLL8	33859	0.001180	*GDAP1L1*	33848	0.001170	*CPNE5*	33798	0.001170	*PSME3IP1*	33650	0.001170
PTDSS2	33520	0.001160	*ZNF790*	33359	0.001160	*OAS2*	33328	0.001160	*BBS9*	33251	0.001150
RABL6	33244	0.001150	*NCF2*	33237	0.001150	*VNN1*	33206	0.001150	*ZBTB43*	33204	0.001150
INTS13	33145	0.001150	*APH1B*	33108	0.001150	*METAP1D*	33081	0.001150	*ESPNL*	32947	0.001140
DDX24	32915	0.001140	*PLXNB2*	32667	0.001130	*SPAG16*	32520	0.001130	*CAP1*	32416	0.001130
FAM234A	32370	0.001120	*FSTL4*	32366	0.001120	*PATL1*	32321	0.001120	*PALD1*	32277	0.001120
NACC2	32176	0.001120	*CLPB*	32162	0.001120	*AMPH*	32142	0.001120	*C5AR1*	32043	0.001110
RAD54L	32019	0.001110	*MAPK9*	32004	0.001110	*ITGA7*	31836	0.001110	*ACAA2*	31789	0.001100
IKBIP	31708	0.001100	*PGBD5*	31661	0.001100	*ZBTB8B*	31614	0.001100	*DNAJC5*	31608	0.001100
PPARD	31540	0.001090	*PLA2G7*	31492	0.001090	*PAIP1*	31491	0.001090	*CEP170B*	31479	0.001090
RAB11A	31410	0.001090	*COL4A1*	31362	0.001090	*CCDC69*	31277	0.001090	*VIPAS39*	31277	0.001090
LRP3	31261	0.001090	*ZNF385B*	31128	0.001080	*CCDC138*	31102	0.001080	*PPA1*	31076	0.001080
TM2D3	30947	0.001070	*FGL1*	30941	0.001070	*STMP1*	30922	0.001070	*ZNF217*	30830	0.001070
CFHR4	30781	0.001070	*TCP11*	30539	0.001060	*LRTOMT*	30451	0.001060	*HSPBAP1*	30448	0.001060
PNPLA1	30375	0.001050	*MOB3B*	30209	0.001050	*NCL*	30110	0.001050	*ATP1A4*	30043	0.001040
ERMARD	29978	0.001040	*NSG1*	29928	0.001040	*ABCA1*	29864	0.001040	*UBE2D4*	29700	0.001030
POLG	29663	0.001030	*KIF19*	29610	0.001030	*NOXRED1*	29502	0.001020	*IFNAR1*	29435	0.001020
CAMK1D	29272	0.001020	*COL5A1*	29223	0.001010	*B4GALT4*	29214	0.001010	*ZMYND8*	29112	0.001010
MGA	29103	0.001010	*PPP1R2*	28985	0.001010	*TRIM26*	28972	0.001010	*WLS*	28848	0.001000
TMPRSS13	28818	0.001000	*GLDC*	28782	0.000999	*SULT6B1*	28778	0.000999	*VSTM2A*	28755	0.000998
FGFR2	28731	0.000997	*RAP1GAP2*	28670	0.000995	*ADRA1D*	28657	0.000995	*WDFY2*	28650	0.000994
LYSMD2	28574	0.000992	*BLZF1*	28570	0.000992	*MYH2*	28552	0.000991	*SMIM41*	28551	0.000991
ULK1	28528	0.000990	*F13B*	28519	0.000990	*C5orf34*	28439	0.000987	*XPR1*	28381	0.000985
TCERG1L	28371	0.000985	*LRRTM4*	28337	0.000984	*EGFR*	28302	0.000982	*PROSER1*	28224	0.000980
PRRG4	28193	0.000979	*DFFB*	28162	0.000977	*MTFMT*	28127	0.000976	*SHPK*	28010	0.000972
PPIA	27884	0.000968	*JAG2*	27842	0.000966	*FERMT2*	27822	0.000966	*CLEC1B*	27782	0.000964
ZNF578	27750	0.000963	*SH2B1*	27612	0.000958	*TMEM132B*	27467	0.000953	*GAL3ST2*	27465	0.000953
C2orf88	27408	0.000951	*AK5*	27350	0.000949	*CLPTM1L*	27347	0.000949	*TUBGCP6*	27334	0.000949
SPG21	27285	0.000947	*HCN2*	27278	0.000947	*TXNDC15*	27264	0.000946	*BMP7*	27251	0.000946
BBS5	27175	0.000943	*NME8*	27169	0.000943	*SERPINB12*	26960	0.000936	*VIPR1*	26860	0.000932

Gene	AIS (bp)	% AIS	Gene	AIS (bp)	% AIS	Gene	AIS (bp)	% AIS	Gene	AIS (bp)	% AIS
UBE2V1	26851	0.000932	*CABP1*	26698	0.000927	*ARHGAP24*	26626	0.000924	*MBD5*	26564	0.000922
MKRN1	26503	0.000920	*TRAF6*	26503	0.000920	*USP2*	26496	0.000920	*BAG3*	26439	0.000918
MYH1	26235	0.000911	*MMEL1*	26210	0.000910	*GRHL1*	26094	0.000906	*TSHZ3*	26092	0.000906
VPS16	26029	0.000903	*ZNF84*	26012	0.000903	*IRF2*	25978	0.000902	*ZBTB16*	25928	0.000900
RNF6	25635	0.000890	*CAPN11*	25590	0.000888	*GFPT2*	25578	0.000888	*MFAP5*	25542	0.000887
ASB1	25508	0.000885	*UCK2*	25453	0.000883	*ACAN*	25412	0.000882	*IP6K3*	25243	0.000876
TOGARAM2	25242	0.000876	*SKI*	25239	0.000876	*ZBTB34*	25239	0.000876	*GLOD4*	25197	0.000875
MLC1	25177	0.000874	*MTA1*	25174	0.000874	*SERINC2*	25115	0.000872	*KIRREL1*	25071	0.000870
CTNS	25062	0.000870	*TENM4*	25055	0.000870	*STOML3*	24934	0.000865	*ARID1B*	24850	0.000863
PPT1	24550	0.000852	*CLDN14*	24507	0.000851	*AKR1C8*	24225	0.000841	*UPF3A*	24216	0.000841
LRIT3	24208	0.000840	*SLC12A6*	24058	0.000835	*RTL1*	23969	0.000832	*STARD7*	23968	0.000832
MORN3	23889	0.000829	*ANKUB1*	23882	0.000829	*SLC22A18*	23871	0.000829	*PTPRK*	23816	0.000827
CDH15	23744	0.000824	*A2ML1*	23646	0.000821	*UPK1B*	23566	0.000818	*ARID3A*	23539	0.000817
DMBT1	23458	0.000814	*PCLAF*	23383	0.000812	*PRKG1*	23371	0.000811	*ZC3H12C*	23309	0.000809
EPC2	23277	0.000808	*CSAD*	23262	0.000807	*ANAPC5*	23209	0.000806	*DYNC2I2*	23194	0.000805
GSG1L	23091	0.000801	*RGS17*	22979	0.000798	*FRY*	22971	0.000797	*SDK2*	22963	0.000797
SKA3	22957	0.000797	*ARPP19*	22838	0.000793	*GRIK1*	22817	0.000792	*ACVR1*	22808	0.000792
BOK	22707	0.000788	*FBN3*	22647	0.000786	*CD9*	22562	0.000783	*IFI27L1*	22545	0.000783
ZNF350	22516	0.000782	*FGF12*	22500	0.000781	*PTPRD*	22488	0.000781	*RIN2*	22417	0.000778
RARG	22413	0.000778	*CELF2*	22396	0.000777	*RIOK2*	22380	0.000777	*PDGFD*	22378	0.000777
ZNF184	22375	0.000777	*C1orf87*	22367	0.000776	*PCDH15*	22364	0.000776	*MAN1B1*	22332	0.000775
EDARADD	22251	0.000772	*MMP21*	22241	0.000772	*MYH13*	22233	0.000772	*TIE1*	22126	0.000768
DLGAP2	22075	0.000766	*HTT*	22040	0.000765	*SLC19A2*	22035	0.000765	*WRAP73*	21994	0.000763
CACNG8	21758	0.000755	*DSCAM*	21739	0.000755	*TSPAN33*	21699	0.000753	*RPL30*	21618	0.000750
TTC22	21611	0.000750	*RRAGC*	21574	0.000749	*ZSWIM3*	21508	0.000747	*KRTAP5-11*	21498	0.000746
CPNE7	21488	0.000746	*TRPM8*	21488	0.000746	*DDX58*	21412	0.000743	*PHRF1*	21398	0.000743
SCHIP1	21350	0.000741	*SH3GLB2*	21295	0.000739	*CCDC127*	21285	0.000739	*DRICH1*	21238	0.000737
TP53	21073	0.000731	*CACNG6*	21029	0.000730	*SLC27A4*	20943	0.000727	*ADAMTS2*	20915	0.000726
TMTC2	20880	0.000725	*MCM3*	20867	0.000724	*PCLO*	20858	0.000724	*SLC6A18*	20808	0.000722
TMPRSS7	20778	0.000721	*TTC29*	20668	0.000717	*URM1*	20655	0.000717	*C4A*	20624	0.000716
C4B	20623	0.000716	*LARP7*	20619	0.000716	*CCDC197*	20598	0.000715	*MCEE*	20555	0.000713
PLCZ1	20437	0.000709	*DISP2*	20402	0.000708	*SULT1B1*	20260	0.000703	*RPH3AL*	20251	0.000703
ARHGEF16	20208	0.000701	*XAF1*	20200	0.000701	*KCTD7*	20186	0.000701	*TINCR*	20182	0.000701
COMMD9	20165	0.000700	*SGPP2*	20153	0.000700	*SSBP2*	20143	0.000699	*HK2*	20136	0.000699
HTATIP2	20097	0.000698	*ST6GALNAC6*	20087	0.000697	*TOP1*	19957	0.000693	*RBFOX3*	19918	0.000691
FMN2	19913	0.000691	*TBC1D4*	19882	0.000690	*MPHOSPH10*	19791	0.000687	*NUDT7*	19768	0.000686
FRMD1	19720	0.000684	*PCOLCE2*	19705	0.000684	*MITD1*	19631	0.000681	*NOM1*	19574	0.000679
ZNF778	19438	0.000675	*ITGB2*	19419	0.000674	*SPICE1*	19351	0.000672	*EPHA5*	19256	0.000668
TM4SF19	19161	0.000665	*HS6ST3*	19130	0.000664	*CARD6*	18867	0.000655	*CLIP1*	18740	0.000650
BTN2A1	18717	0.000650	*LDLRAP1*	18682	0.000648	*PAK1*	18621	0.000646	*CAB39L*	18541	0.000644
MANSC1	18514	0.000643	*ST6GALNAC3*	18506	0.000642	*PALM*	18479	0.000641	*MRPL30*	18409	0.000639
ATP6V0A4	18382	0.000638	*ZFP37*	18382	0.000638	*IL17REL*	18310	0.000636	*CHD1L*	18265	0.000634
ALG12	18209	0.000632	*CD300LF*	18197	0.000632	*CANT1*	18151	0.000630	*TBC1D16*	18129	0.000629
FEM1B	18117	0.000629	*SCGB1A1*	18092	0.000628	*PLCB4*	17996	0.000625	*DENND6B*	17982	0.000624
CACNB4	17967	0.000624	*EHMT2*	17939	0.000623	*SLC17A2*	17937	0.000623	*LEMD2*	17928	0.000622
XRCC3	17895	0.000621	*SHC3*	17775	0.000617	*GTSF1*	17652	0.000613	*RNF114*	17549	0.000609
SPATA45	17508	0.000608	*ERC2*	17478	0.000607	*AFF3*	17476	0.000607	*NDUFA10*	17466	0.000606
AHNAK	17458	0.000606	*NLRX1*	17448	0.000606	*RIMOC1*	17448	0.000606	*WRAP53*	17431	0.000605
LAMTOR1	17402	0.000604	*PXMP2*	17398	0.000604	*LRRC56*	17385	0.000603	*SLC37A1*	17360	0.000603
SLC8A1	17305	0.000601	*SCD5*	17300	0.000600	*SLFN11*	17231	0.000598	*DAPP1*	17113	0.000594
SYNE1	17101	0.000594	*STK25*	17060	0.000592	*MARCHF3*	17048	0.000592	*PGAP2*	16831	0.000584
GRM4	16749	0.000581	*MAPK13*	16715	0.000580	*SYT2*	16675	0.000579	*CCR9*	16665	0.000578
RASGEF1C	16657	0.000578	*ZNF30*	16641	0.000578	*SELENOO*	16605	0.000576	*LRRC51*	16558	0.000575
KLHL41	16556	0.000575	*NCAPH2*	16556	0.000575	*PLOD1*	16516	0.000573	*SVIP*	16500	0.000573
FBXO4	16489	0.000572	*IBA57*	16453	0.000571	*MYO1G*	16426	0.000570	*GK5*	16384	0.000569
TMIE	16358	0.000568	*DDX27*	16319	0.000566	*GRIN1*	16319	0.000566	*KLF17*	16319	0.000566
GALNT8	16169	0.000561	*HELZ2*	16153	0.000561	*CCT2*	16144	0.000560	*HTR6*	16091	0.000559
GPR132	16050	0.000557	*ATP2A3*	15983	0.000555	*PIK3R6*	15981	0.000555	*ITGB7*	15959	0.000554
TMEM138	15926	0.000553	*CDC42BPB*	15922	0.000553	*CFAP107*	15894	0.000552	*CFHR2*	15884	0.000551
CREB5	15836	0.000550	*PLCB1*	15820	0.000549	*CBFA2T3*	15795	0.000548	*ROBO1*	15760	0.000547
SLC10A7	15727	0.000546	*OAZ2*	15708	0.000545	*DCLK1*	15695	0.000545	*DEGS2*	15688	0.000545
PGAP6	15657	0.000543	*PRELP*	15546	0.000540	*TTC16*	15511	0.000538	*TMEM14A*	15475	0.000537
ENOX1	15401	0.000535	*QSOX2*	15365	0.000533	*MMP8*	15254	0.000529	*ABHD17C*	15243	0.000529
ABCC4	15193	0.000527	*C5AR2*	15154	0.000526	*TAGLN3*	15148	0.000526	*EXOC3L4*	15116	0.000525
DOCK5	15086	0.000524	*USP44*	15069	0.000523	*FBN2*	14868	0.000516	*OSER1*	14832	0.000515
OPTC	14796	0.000514	*PWWP2B*	14767	0.000513	*MYO1E*	14709	0.000511	*GRM6*	14667	0.000509
MS4A12	14652	0.000509	*FAT3*	14497	0.000503	*ASGR2*	14378	0.000499	*FAM151A*	14375	0.000499
ABHD10	14358	0.000498	*TARBP1*	14327	0.000497	*KLHL30*	14279	0.000496	*P2RX3*	14221	0.000494
RAD52	14209	0.000493	*NUDT2*	14205	0.000493	*VPS11*	14153	0.000491	*DOCK10*	14136	0.000491
RDH10	14088	0.000489	*C12orf54*	14026	0.000487	*COMMD2*	14023	0.000487	*TARP*	13993	0.000486
DHRS12	13959	0.000485	*RLF*	13951	0.000484	*UHRF1*	13912	0.000483	*SYCE1L*	13807	0.000479
SNRPA1	13772	0.000478	*TRABD*	13685	0.000475	*TAFA5*	13648	0.000474	*ZNF331*	13639	0.000473
ABCG4	13600	0.000472	*CIDEC*	13544	0.000470	*CYB561A3*	13529	0.000470	*STAC*	13502	0.000469
IL17RA	13498	0.000469	*SH2D4A*	13470	0.000468	*GSTA2*	13388	0.000465	*FBXL16*	13310	0.000462
SLC31A2	13164	0.000457	*PTGIS*	13138	0.000456	*ZNF735*	13087	0.000454	*MMACHC*	13082	0.000454
UQCRH	13078	0.000454	*SDC3*	13064	0.000453	*SPRR2E*	13049	0.000453	*PLD4*	13048	0.000453
BTN3A1	12971	0.000450	*BTN3A3*	12943	0.000449	*PRDM15*	12929	0.000449	*HBM*	12872	0.000447
OR5AN1	12819	0.000445	*CACNA1E*	12769	0.000443	*R3HDM4*	12751	0.000443	*SLC1A4*	12712	0.000441
SLC11A1	12706	0.000441	*TXN*	12697	0.000441	*ITGA1*	12678	0.000440	*PDIA6*	12597	0.000437
ANKRD66	12573	0.000436	*SYT9*	12559	0.000436	*MYO1D*	12508	0.000434	*LYPD6B*	12489	0.000433
SLC23A2	12416	0.000431	*NCS1*	12385	0.000430	*DCBLD2*	12316	0.000427	*DPAGT1*	12306	0.000427
LYZL2	12276	0.000426	*TIMP3*	12181	0.000423	*TSPYL1*	12170	0.000422	*ZNF598*	12167	0.000422
P2RX5	12104	0.000420	*OMD*	12091	0.000420	*HTR3A*	12046	0.000418	*IRAG1*	12020	0.000417
CAMK2A	11967	0.000415	*MICAL3*	11959	0.000415	*SWI5*	11946	0.000415	*ATP5PO*	11937	0.000414
PGAM5	11915	0.000414	*SQOR*	11882	0.000412	*HTR3B*	11829	0.000411	*NHLRC3*	11803	0.000410
IMPA1	11789	0.000409	*PRODH2*	11722	0.000407	*SGCA*	11714	0.000407	*MCRIP2*	11657	0.000405

Gene	AIS (bp)	% AIS	Gene	AIS (bp)	% AIS	Gene	AIS (bp)	% AIS	Gene	AIS (bp)	% AIS
ODF3L2	11637	0.000404	*NFATC1*	11618	0.000403	*KNSTRN*	11567	0.000401	*MON1B*	11563	0.000401
OR10G3	11559	0.000401	*GRM2*	11545	0.000401	*PRICKLE2*	11505	0.000399	*EIF5*	11481	0.000399
DYNLT2	11478	0.000398	*BMF*	11476	0.000398	*DTD2*	11470	0.000398	*PIK3R1*	11468	0.000398
C12orf40	11462	0.000398	*CIZ1*	11401	0.000396	*ZNF704*	11357	0.000394	*SLC2A8*	11286	0.000392
FLG2	11271	0.000391	*ZNF615*	11270	0.000391	*CATIP*	11242	0.000390	*TINAGL1*	11201	0.000389
RET	11105	0.000385	*SHLD1*	11091	0.000385	*EEF1AKMT3*	11049	0.000384	*RGL4*	11040	0.000383
PRKD1	10993	0.000382	*POP4*	10954	0.000380	*PRIMA1*	10931	0.000379	*MYCBP*	10875	0.000377
MAB21L4	10841	0.000376	*CCDC170*	10767	0.000374	*GABARAPL1*	10670	0.000370	*NPW*	10641	0.000369
FIZ1	10594	0.000368	*TLCD3A*	10556	0.000366	*ITIH5*	10542	0.000366	*UBE2T*	10440	0.000362
C22orf42	10316	0.000358	*C5orf49*	10309	0.000358	*LAP3*	10258	0.000356	*COPS9*	10244	0.000356
DPYSL4	10190	0.000354	*MRM3*	10156	0.000353	*ABCG1*	10063	0.000349	*CREG1*	10031	0.000348
ZNF860	9973	0.000346	*PRSS57*	9952	0.000345	*RNF166*	9909	0.000344	*RAB11FIP3*	9906	0.000344
GJC2	9896	0.000343	*MAMDC4*	9856	0.000342	*SLC22A7*	9844	0.000342	*PGA3*	9791	0.000340
FGR	9770	0.000339	*GALP*	9767	0.000339	*SAR1B*	9666	0.000336	*GLT6D1*	9664	0.000335
TFEC	9647	0.000335	*HBZ*	9602	0.000333	*BLCAP*	9587	0.000333	*GOLGA6L4*	9585	0.000333
LSM2	9570	0.000332	*PANX2*	9564	0.000332	*CCDC9B*	9487	0.000329	*CRELD2*	9373	0.000325
HMGA1	9358	0.000325	*PGA4*	9347	0.000324	*SLC6A19*	9343	0.000324	*OR4D9*	9335	0.000324
GPR35	9287	0.000322	*ANG*	9276	0.000322	*RNASE4*	9276	0.000322	*SRMS*	9260	0.000321
NME4	9255	0.000321	*PURB*	9231	0.000320	*KIAA1522*	9191	0.000319	*ST6GALNAC4*	9155	0.000318
CRIP3	9116	0.000316	*C15orf61*	9015	0.000313	*CFAP157*	9014	0.000313	*PDCD1*	9010	0.000313
IL17F	9008	0.000313	*LGI2*	8985	0.000312	*CYP4F8*	8983	0.000312	*SAP18*	8978	0.000312
HMBS	8968	0.000311	*ACOT6*	8943	0.000310	*CTU2*	8913	0.000309	*NFE2*	8904	0.000309
CPAMD8	8851	0.000307	*SNAI3*	8819	0.000306	*FNDC11*	8804	0.000306	*MMP1*	8759	0.000304
TSEN2	8718	0.000303	*HERPUD1*	8713	0.000302	*DUSP26*	8693	0.000302	*TM4SF1*	8670	0.000301
NLRP7	8590	0.000298	*NEU4*	8579	0.000298	*SNRPN*	8574	0.000298	*SLC25A29*	8566	0.000297
FAM240C	8563	0.000297	*NACAD*	8532	0.000296	*FXYD3*	8501	0.000295	*IQCF1*	8477	0.000294
RRP9	8475	0.000294	*SAPCD2*	8430	0.000293	*NOS1*	8405	0.000292	*TLCD5*	8390	0.000291
NUDT14	8385	0.000291	*NRP2*	8329	0.000289	*C14orf28*	8323	0.000289	*CHD5*	8307	0.000288
ATG101	8249	0.000286	*XDH*	8173	0.000284	*CLEC12B*	8169	0.000284	*C3*	8163	0.000283
SRSF6	8153	0.000283	*EPB41L3*	8087	0.000281	*BSG*	8049	0.000279	*NOC4L*	8016	0.000278
RNASE11	8004	0.000278	*COL1A1*	7964	0.000276	*B3GALT2*	7883	0.000274	*FBXO48*	7872	0.000273
RASGRF1	7823	0.000272	*CPA1*	7775	0.000270	*DDX51*	7725	0.000268	*SZRD1*	7724	0.000268
RIPOR3	7717	0.000268	*FAM131B*	7709	0.000268	*MMP9*	7653	0.000266	*DUSP7*	7652	0.000266
GJA9	7518	0.000261	*RFX3*	7509	0.000261	*LRCOL1*	7432	0.000258	*SURF6*	7413	0.000257
PPIB	7394	0.000257	*MRGPRE*	7387	0.000256	*OR3A1*	7343	0.000255	*SLC25A35*	7338	0.000255
SLCO1B3	7275	0.000253	*RPH3A*	7209	0.000250	*CRIP2*	7200	0.000250	*THBS2*	7197	0.000250
BTBD2	7187	0.000249	*CNTLN*	7180	0.000249	*FOXL1*	7110	0.000247	*RAD18*	7100	0.000246
PARVB	7078	0.000246	*PRDM11*	7072	0.000245	*MAPK11*	7054	0.000245	*UAP1L1*	7022	0.000244
NT5C1A	6953	0.000241	*CNDP2*	6933	0.000241	*HNRNPA1*	6895	0.000239	*TMEM255B*	6874	0.000239
ITSN1	6861	0.000238	*ZMIZ1*	6859	0.000238	*KIAA2013*	6837	0.000237	*PROSER2*	6727	0.000233
NPDC1	6716	0.000233	*SELENOS*	6683	0.000232	*MAP3K19*	6665	0.000231	*CYP27B1*	6652	0.000231
HPCAL1	6602	0.000229	*TRAPPC2L*	6466	0.000224	*GKN1*	6407	0.000222	*AJM1*	6323	0.000219
MVD	6242	0.000217	*ARHGAP22*	6217	0.000216	*CCDC163*	6213	0.000216	*EFNB3*	6170	0.000214
RPL26L1	6136	0.000213	*TRPC4*	6081	0.000211	*APCDD1L*	6044	0.000210	*CTR9*	5983	0.000208
FSTL3	5970	0.000207	*FFAR1*	5961	0.000207	*ENTPD2*	5954	0.000207	*MED8*	5891	0.000204
SLC22A31	5890	0.000204	*PCED1A*	5876	0.000204	*LBHD2*	5817	0.000202	*SMYD3*	5783	0.000201
SNX22	5766	0.000200	*VGLL4*	5759	0.000200	*SEMA5A*	5756	0.000200	*PRSS50*	5752	0.000200
HFE	5727	0.000199	*NGB*	5723	0.000199	*HSPA1L*	5664	0.000197	*RPUSD2*	5650	0.000196
MTCL1	5635	0.000196	*COLEC12*	5607	0.000195	*CDH4*	5606	0.000195	*SACS*	5600	0.000194
TMC6	5595	0.000194	*OTOS*	5536	0.000192	*KRT6B*	5495	0.000191	*EN1*	5477	0.000190
CDT1	5469	0.000190	*ARSA*	5419	0.000188	*POLR3G*	5370	0.000186	*NAGK*	5362	0.000186
MARCHF9	5308	0.000184	*KRT6C*	5289	0.000184	*SOX8*	5209	0.000181	*ITPRIPL1*	5200	0.000180
CFAP300	5191	0.000180	*CFAP74*	5161	0.000179	*EMC4*	5143	0.000179	*HRAS*	5079	0.000176
SHISAL1	5057	0.000176	*TTC39C*	5046	0.000175	*PTPRH*	5045	0.000175	*PRXL2B*	4978	0.000173
SLC7A10	4965	0.000172	*TLK1*	4931	0.000171	*SIAH3*	4880	0.000169	*WFIKKN1*	4878	0.000169
SMOC1	4860	0.000169	*TMEM247*	4860	0.000169	*RASGRF2*	4858	0.000169	*ACSS3*	4856	0.000169
KLHL29	4849	0.000168	*LRRC14B*	4839	0.000168	*LEFTY2*	4785	0.000166	*CHRM3*	4782	0.000166
TERT	4780	0.000166	*LMF2*	4753	0.000165	*APP*	4644	0.000161	*DPP7*	4631	0.000161
BEST4	4585	0.000159	*NPBWR1*	4552	0.000158	*TEX22*	4551	0.000158	*METRN*	4537	0.000157
TRIML1	4533	0.000157	*DEFB108B*	4510	0.000157	*TRNT1*	4495	0.000156	*CCDC183*	4439	0.000154
ZFYVE28	4432	0.000154	*TRRAP*	4422	0.000153	*ZNF669*	4410	0.000153	*GJA10*	4390	0.000152
RDH5	4338	0.000151	*MRPL28*	4320	0.000150	*COL4A2*	4316	0.000150	*EFCAB8*	4310	0.000150
BSX	4264	0.000148	*RTP5*	4223	0.000147	*BMPER*	4198	0.000146	*OSBPL9*	4159	0.000144
DECR2	4122	0.000143	*TSPYL4*	4111	0.000143	*TMEM18*	4097	0.000142	*TMPRSS2*	4082	0.000142
ZNF579	4074	0.000141	*HDAC4*	4072	0.000141	*PPP2R2C*	4067	0.000141	*BLOC1S1*	4049	0.000141
PITPNC1	4038	0.000140	*KRT18*	4035	0.000140	*PLA2G2D*	4030	0.000140	*VAV1*	4021	0.000140
IQCA1	4010	0.000139	*TMEM239*	3982	0.000138	*TWIST2*	3946	0.000137	*TUFM*	3937	0.000137
FRMD4B	3872	0.000134	*SLC47A2*	3845	0.000133	*KLF18*	3810	0.000132	*CYP24A1*	3785	0.000131
ADGRA1	3774	0.000131	*NKX1-2*	3772	0.000131	*FRMD5*	3743	0.000130	*SMPD3*	3722	0.000129
RILP	3721	0.000129	*SCUBE2*	3705	0.000129	*CFAP61*	3692	0.000128	*METTL1*	3648	0.000127
CA6	3638	0.000126	*POLRMT*	3625	0.000126	*TMEM121*	3619	0.000126	*ATP4A*	3613	0.000125
BCL2L10	3512	0.000122	*MIXL1*	3442	0.000119	*ORM1*	3381	0.000117	*TBC1D22A*	3370	0.000117
MBP	3352	0.000116	*PABPN1L*	3327	0.000115	*SYT8*	3305	0.000115	*FZD10*	3281	0.000114
SFXN5	3280	0.000114	*APOC4*	3259	0.000113	*CNPY3*	3212	0.000111	*NCR1*	3205	0.000111
USP10	3175	0.000110	*GAS1*	3144	0.000109	*HIVEP3*	3140	0.000109	*ZNF524*	3116	0.000108
SCARF1	3099	0.000108	*LRP6*	3095	0.000107	*ABHD12B*	3082	0.000107	*EDF1*	3077	0.000107
HOXA1	3007	0.000104	*SCGB2A2*	3002	0.000104	*DNAJB6*	2979	0.000103	*PHGR1*	2958	0.000103
ZNF80	2947	0.000102	*TMPRSS15*	2924	0.000101	*SLC38A8*	2896	0.000101	*C1orf21*	2772	0.000096
SMIM29	2727	0.000095	*PRRT4*	2664	0.000093	*MMP17*	2646	0.000092	*RPUSD1*	2613	0.000091
FIGNL2	2596	0.000090	*HSPA1B*	2516	0.000087	*PRB3*	2492	0.000087	*UNC5A*	2490	0.000086
ZBTB12	2473	0.000086	*SLC16A11*	2471	0.000086	*SAFB2*	2422	0.000084	*HSPA1A*	2403	0.000083
INF2	2381	0.000083	*ATP10A*	2349	0.000082	*JAGN1*	2347	0.000082	*LRRC10*	2343	0.000081
IQGAP2	2337	0.000081	*SLC41A3*	2325	0.000081	*PREX2*	2323	0.000081	*GREB1*	2298	0.000080
MFRP	2271	0.000079	*SPRR2D*	2199	0.000076	*MRPS26*	2198	0.000076	*ROR1*	2127	0.000074
GATD3	2093	0.000073	*CLDN8*	2091	0.000073	*ARHGAP8*	2042	0.000071	*FFAR3*	2029	0.000070
GLIS1	2007	0.000070	*COLCA2*	2000	0.000069	*C1QTNF5*	1998	0.000069	*GCH1*	1997	0.000069

Gene	AIS (bp)	% AIS	Gene	AIS (bp)	% AIS	Gene	AIS (bp)	% AIS	Gene	AIS (bp)	% AIS
CAPN15	1964	0.000068	*PRR5-ARHGAP8*	1960	0.000068	*MBOAT7*	1955	0.000068	*OR6B3*	1950	0.000068
METTL26	1939	0.000067	*FUT7*	1914	0.000066	*CCL3*	1913	0.000066	*GPR42*	1894	0.000066
IQCF5	1880	0.000065	*DPEP1*	1833	0.000064	*SLC43A2*	1819	0.000063	*S1PR4*	1731	0.000060
GAK	1717	0.000060	*SPRR1B*	1697	0.000059	*SYTL2*	1648	0.000057	*IL20*	1646	0.000057
KRTAP10-4	1642	0.000057	*H2AX*	1591	0.000055	*ARHGAP10*	1589	0.000055	*ERC1*	1557	0.000054
CRCT1	1526	0.000053	*C12orf71*	1521	0.000053	*KHDC3L*	1513	0.000053	*PCGF3*	1493	0.000052
TNFSF14	1449	0.000050	*PPDPF*	1441	0.000050	*CLEC10A*	1439	0.000050	*ZNRF4*	1439	0.000050
RANGRF	1438	0.000050	*ACOT7*	1402	0.000049	*KRTAP5-7*	1402	0.000049	*SPRR2F*	1395	0.000048
LCE5A	1374	0.000048	*CSNK2A3*	1308	0.000045	*RNASE12*	1303	0.000045	*LVRN*	1282	0.000045
GPR32	1268	0.000044	*C1QTNF9B*	1223	0.000043	*DPPA5*	1213	0.000042	*COX8C*	1174	0.000041
KRTAP10-5	1149	0.000040	*PHLDA2*	1147	0.000040	*CCL21*	1131	0.000039	*NDUFAF6*	1105	0.000038
CCNG1	1084	0.000038	*IQSEC1*	998	0.000035	*ATXN2L*	990	0.000034	*OR1L3*	974	0.000034
OR1E2	971	0.000034	*IFNA4*	969	0.000034	*OR3A3*	965	0.000034	*RNASE3*	963	0.000033
IFNA10	962	0.000033	*RNASE2*	949	0.000033	*OR13D1*	944	0.000033	*WBP11*	944	0.000033
OR4D11	935	0.000033	*OXT*	897	0.000031	*KRTAP13-2*	881	0.000031	*MRGPRG*	869	0.000030
COTL1	850	0.000030	*HBA1*	842	0.000029	*HBA2*	834	0.000029	*TRPM5*	799	0.000028
H1-5	796	0.000028	*H1-1*	755	0.000026	*IFNA7*	736	0.000026	*HBQ1*	720	0.000025
SMARCA2	708	0.000025	*NOTCH1*	547	0.000019	*PTGES2*	539	0.000019	*H3C11*	501	0.000017
PPIAP40	489	0.000017	*H2AC16*	481	0.000017	*H2AC14*	475	0.000017	*H2BC14*	467	0.000016
FGF1	412	0.000014	*H4C11*	388	0.000014	*H4C13*	386	0.000013	*H4C12*	386	0.000013
KRTAP25-1	369	0.000013	*AFAP1L2*	355	0.000012	*ABCA2*	298	0.000010	*MTFR2*	235	0.000008
SLC44A4	113	0.000004									

Note: Archaic proportion is calculated as the length of archaic introgressed segments (AIS) in each gene devided by the total AIS in the genome.

S23. Supplementary Figures

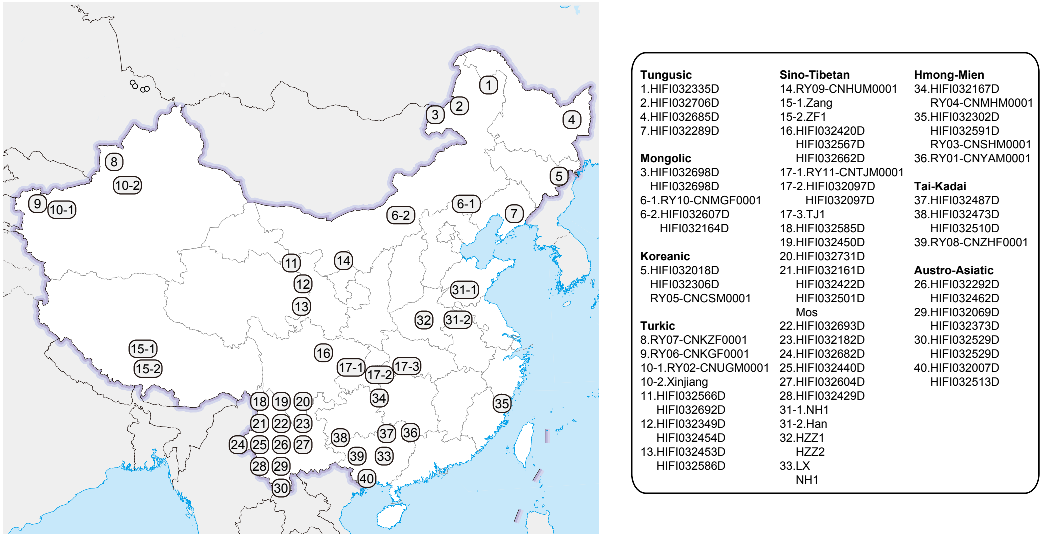

Supplementary Fig. 1 | Geographical distribution of CPC samples.

Showing here are the geographical locations of all samples included in the CPC Phase I. See Supplementary Table 1 for details of the CPC sample information. The China map used in this study was obtained from a standard map service (http://bzdt.ch.mnr.gov.cn) approved by the Ministry of National Resources of the People's Republic of China (GS [2020] 4618).

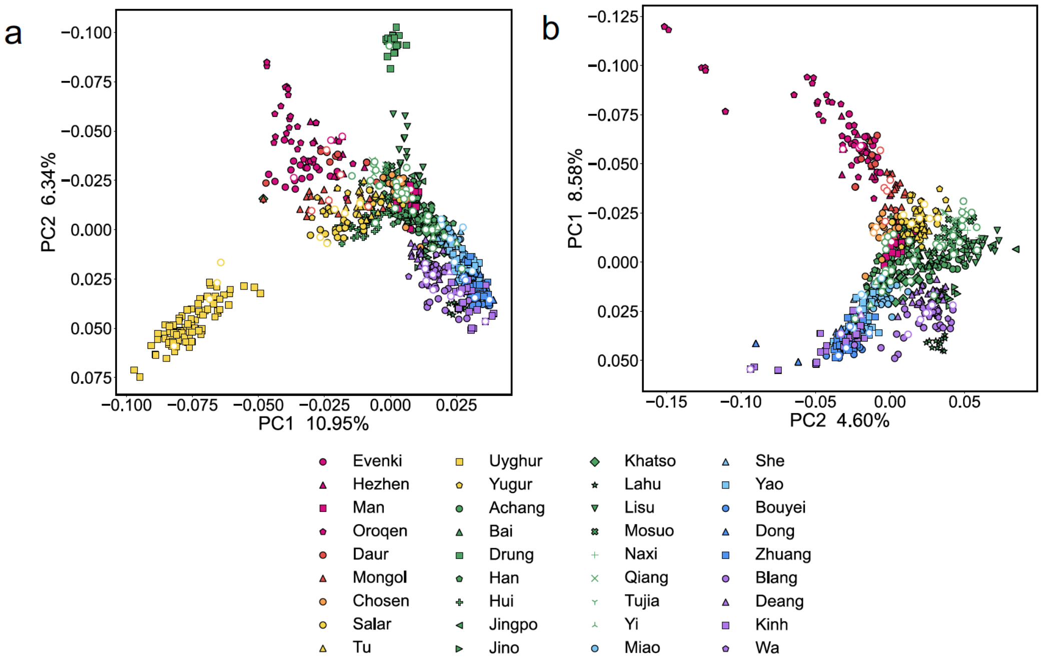

Supplementary Fig. 2 | Detailed PCA of CPC samples in the context of East Asian populations.

(a) PCA showing population information for all samples in the PC plot of Figure 1a. (b) PCA showing population information for samples in the PC plot of Fig. 1a after removing the two outlier clusters in lower left and upper right of (a). Different colors represent populations speaking different language families and correspond to the Fig. 1a. The CPC and HPRC samples are presented as hollow markers.

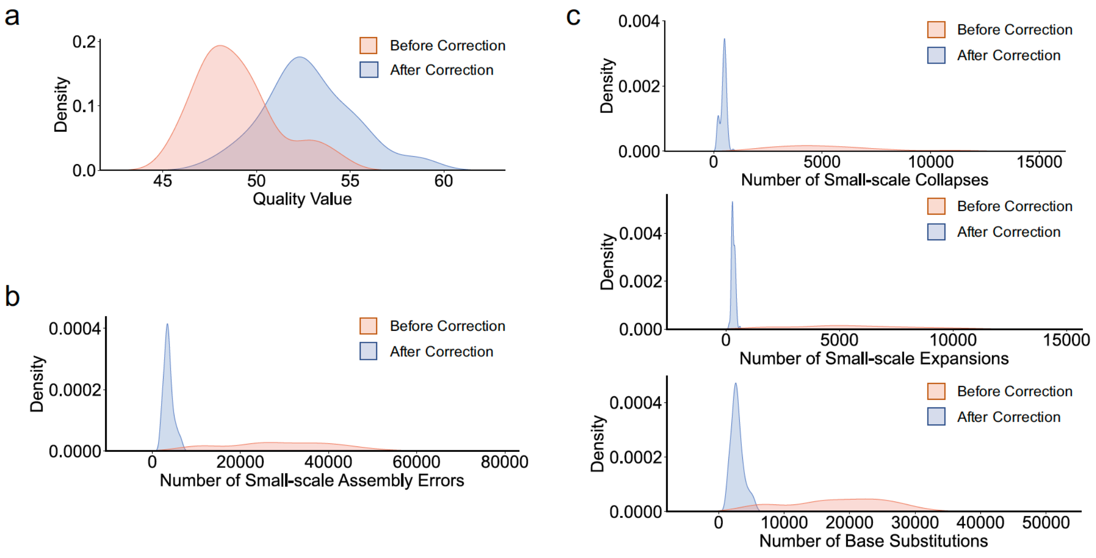

Supplementary Fig. 3 | Evaluation of assembly correction of CPC genome assemblies.

Comparison of (a) assembly quality value (QV), (b) number of all small-scale mis-assemblies, and (c) number of three types of small-scale mis-assemblies before and after assembly polishing, evaluated by the Inspector.

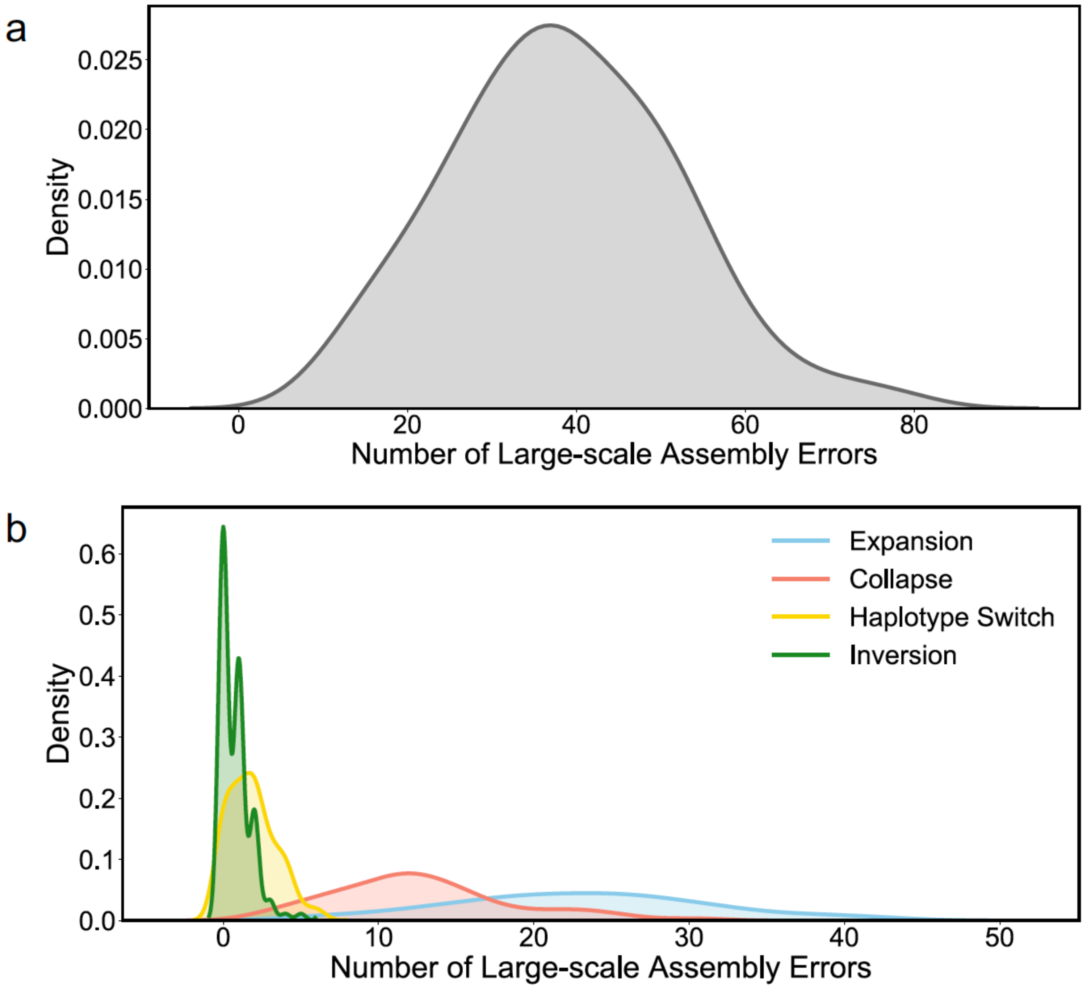

Supplementary Fig. 4 | Evaluation of large-scale assembly errors of CPC genome assemblies.

(a) Density plot showing the distribution of all large-scale assembly errors in the the core assembly of 58 CPC samples. (b) Density plot showing the four types of large-scale assembly errors for the core assembly of 58 CPC samples.

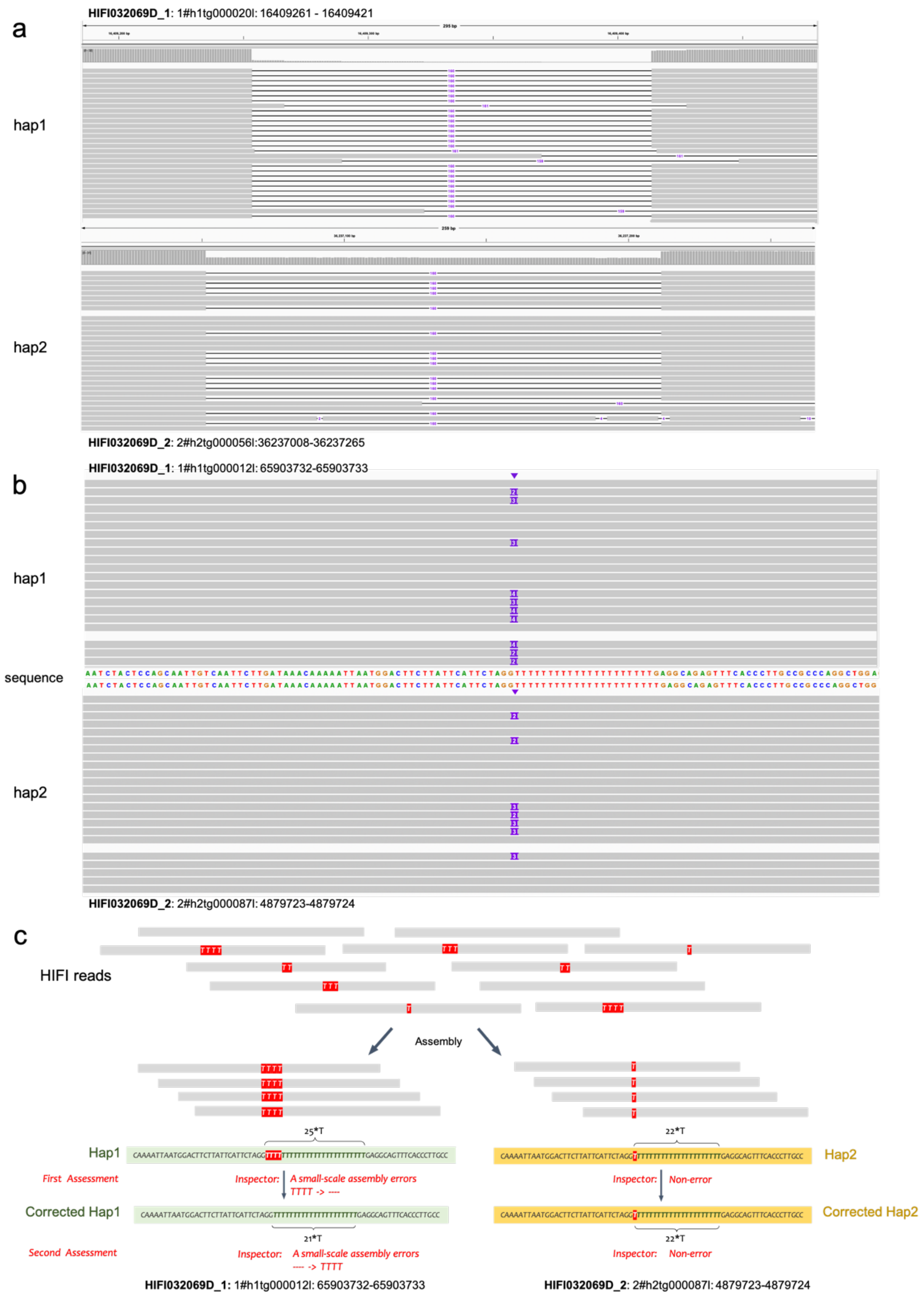

Supplementary Fig. 5 | IGV visualization of small-scale assembly error cases in HIFI032069D-H1.

Shown are (a) correctable and (b, c) uncertain cases of assembly errors assessed by Inspector in the polished assembly of HIFI032069D-H1. The (c) shows a difficult to determine case of sequencing errors mixed with assembly errors.

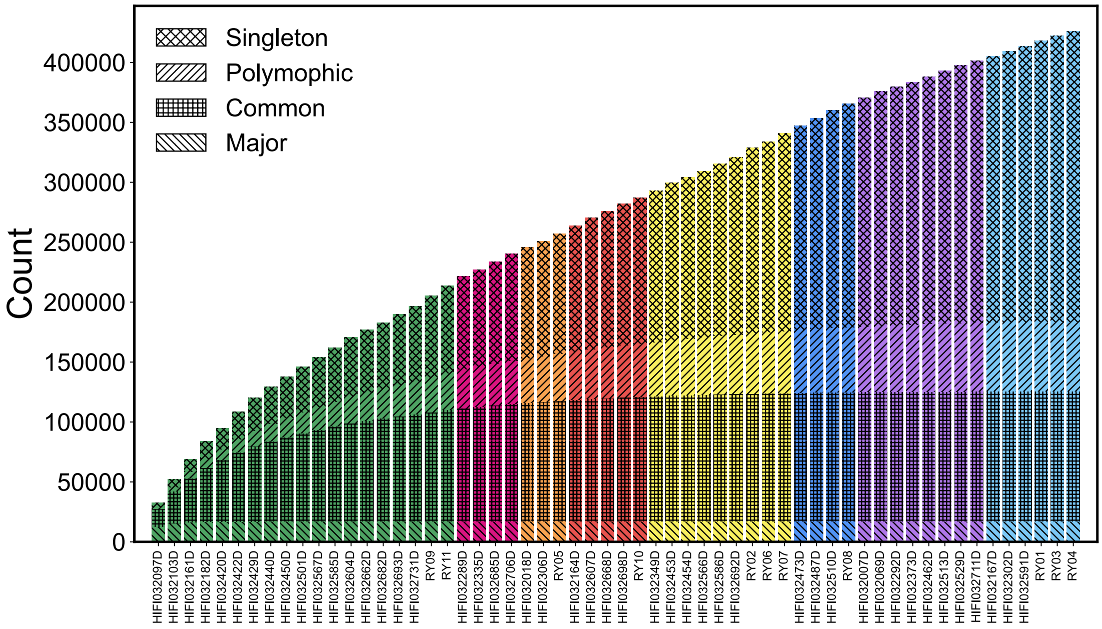

Supplementary Fig. 6 | Cumulative number of SVs by adding CPC samples one by one.

Different shadows in each bar indicate SVs shared among all CPC samples that not present in T2T-CHM13 reference. These varians are classified into major allele variants (AF ≥ 50%, purple), common allele variants (5% ≤ AF < 50%, purple), polymorphisms (≥ 2 haplotypes but AF < 5%), and singletons.

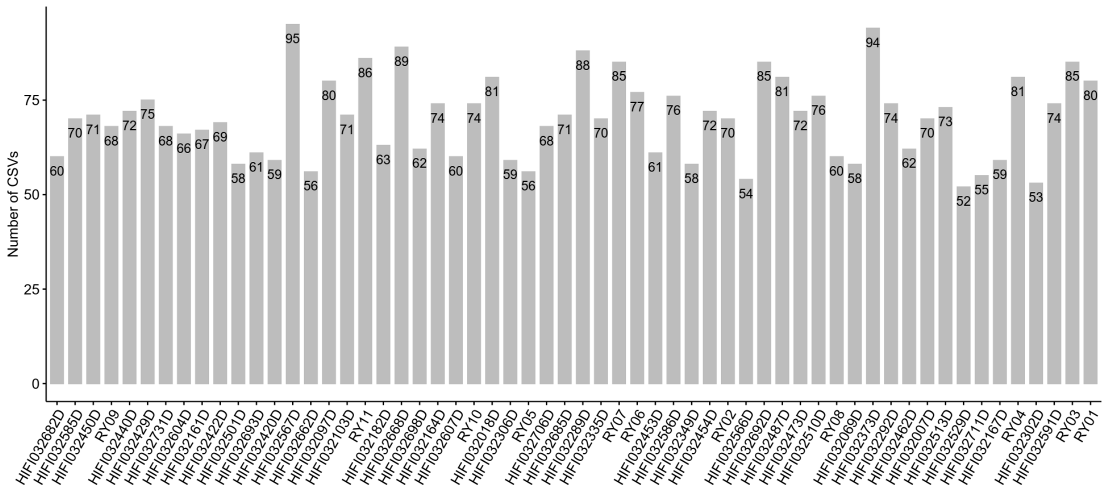

Supplementary Fig. 7 | The count of CSVs in each sample.

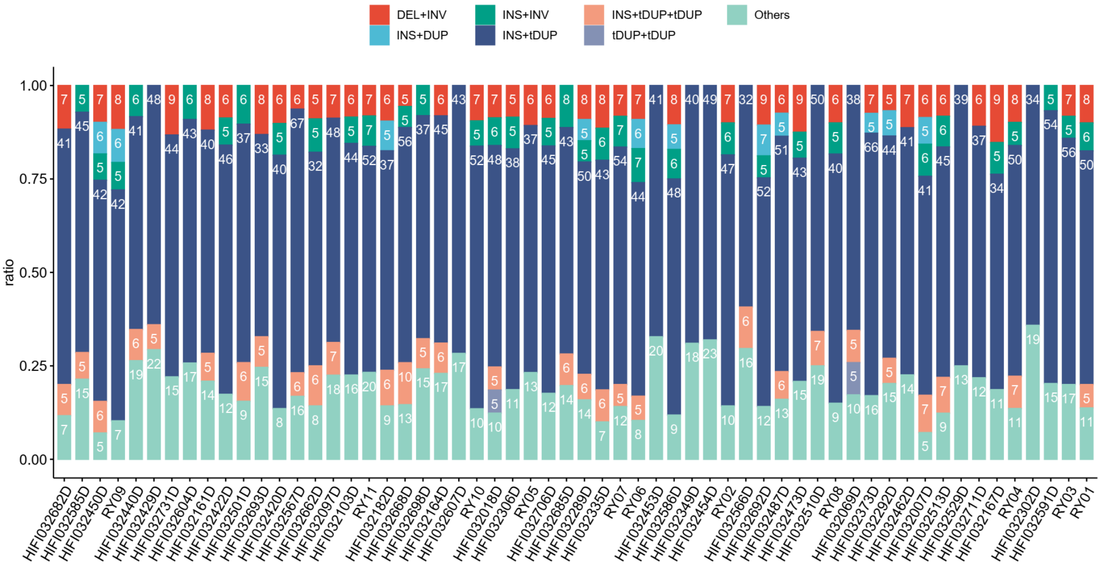

Supplementary Fig. 8 | The number of CSVs for each CSV type in each sample.

The type of INS+tDUP is the most abundant CSV across all samples. Others indicate the CSV types with count smaller than five in each sample.

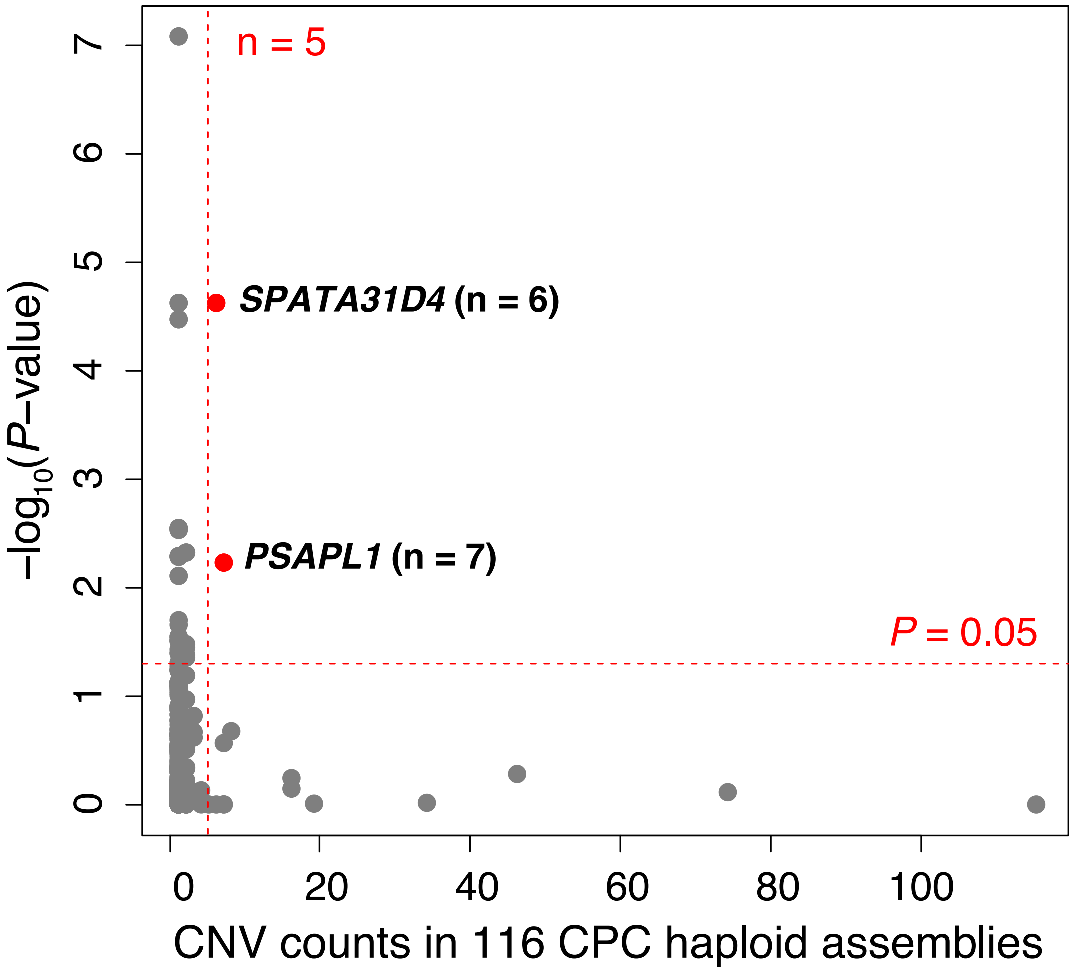

Supplementary Fig. 9 | Tajima's D estimated for the CNV genes identified in the CPC assemblies.

Tajima's D was calculated for the 58 CPC samples (116 haploid assemblies) with a sliding window spanning 20 kb in size, stepping 10 kb. The estimate for each gene was represented by the top window overlapping that gene. The horizontal dashed line shows the threshold of P = 0.05, which is obtained by the two-tailed significance test based on the beta distribution, and is further adjusted for multiple testing using the FDR method; the vertical dashed line indicates the CNV counts ≥ 5.

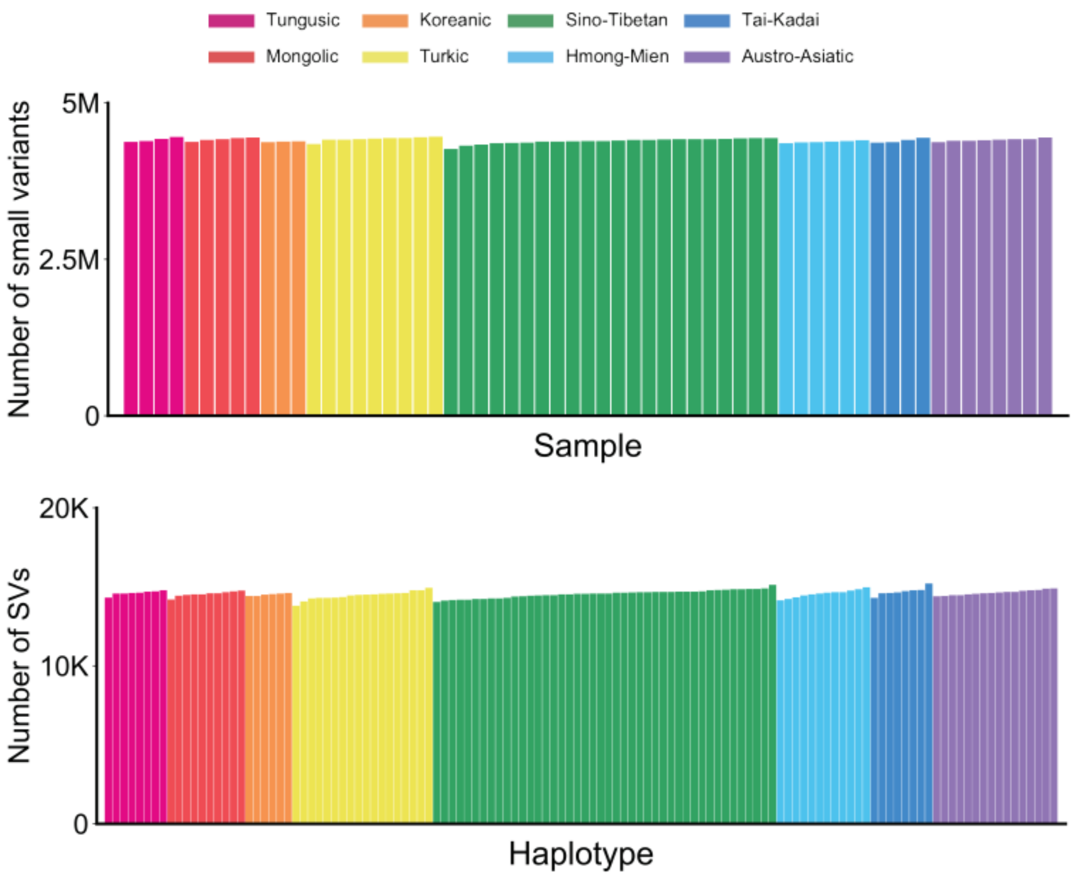

Supplementary Fig. 10 | Number of identified small variants and SVs inferred from the pangenome graph.

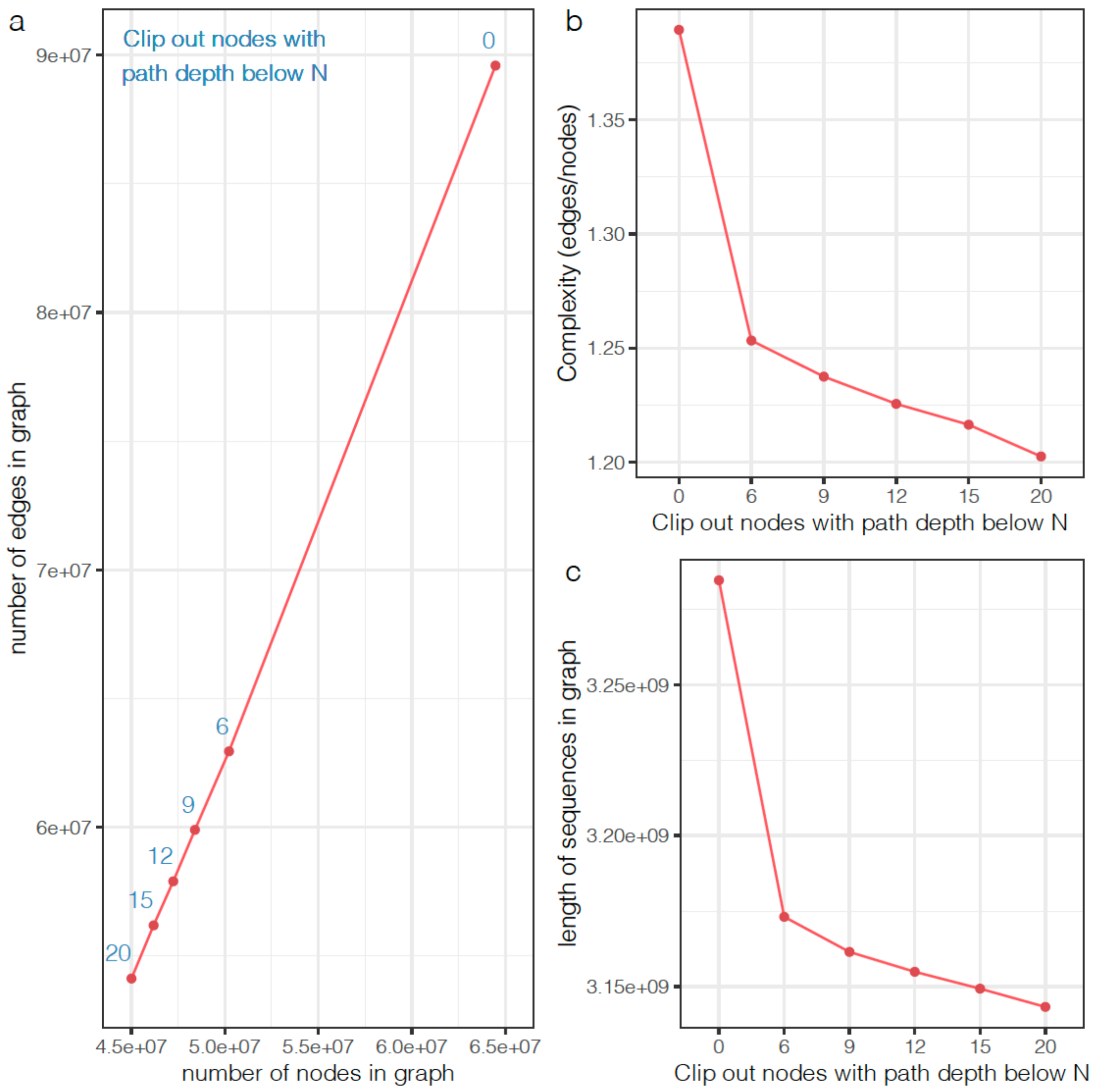

Supplementary Fig. 11 | Statistics of CPC graph reference with different filtering standards.

(a) The number of nodes and edges in graph. The blue numbers show the filter criteria (vg clip -d). (b) The complexity of graph genome. It is obtained by dividing the number of edges by the number of nodes. (c) The length of sequences in graph, i.e., the sum of the lengths of all nodes.

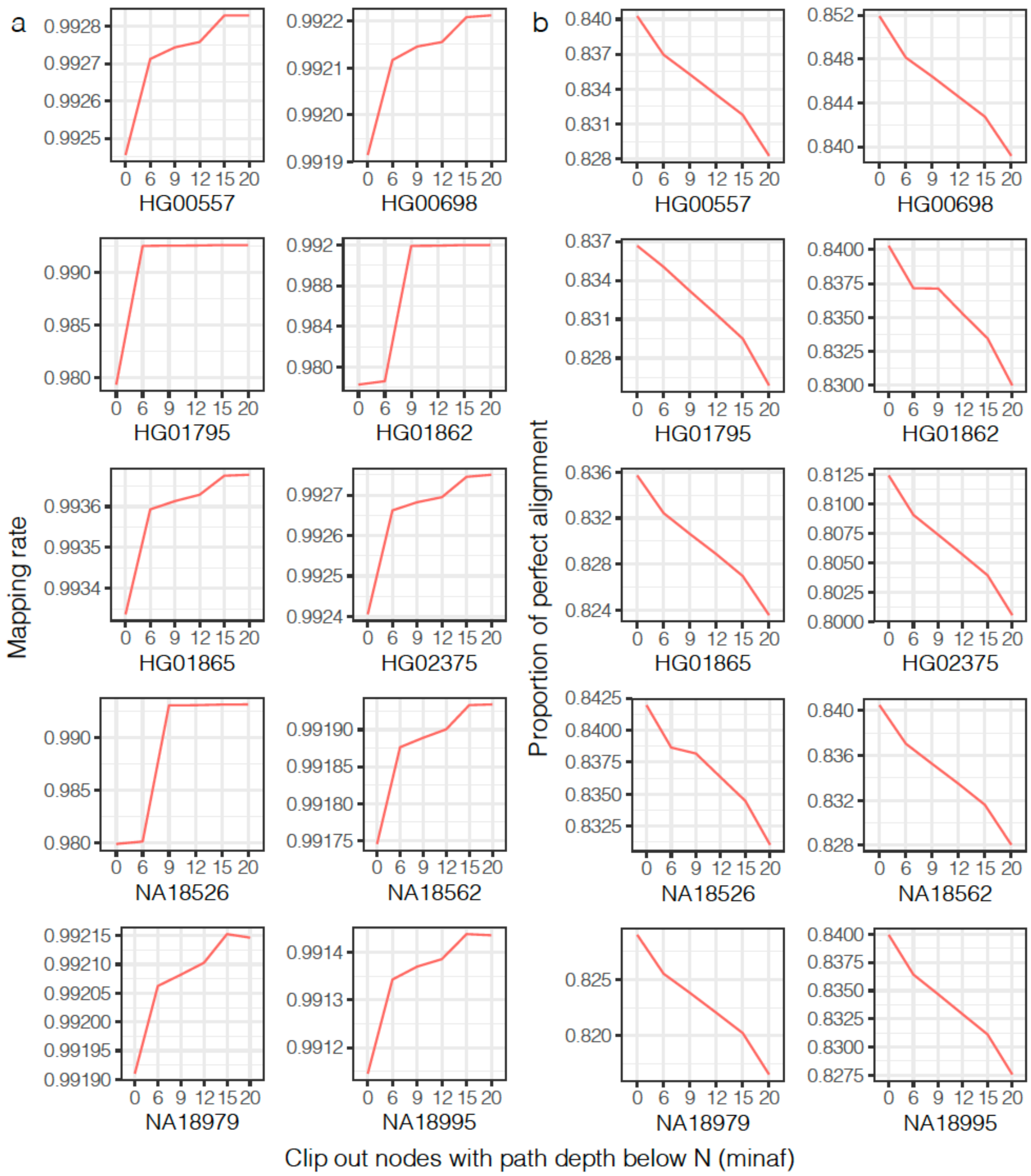

Supplementary Fig. 12 | Mapping rate and perfect rate of 10 East Asian samples using CPC graph reference with different complexity.

(a) Mapping rate. One sample in each figure. The abscissa shows different filtering criteria. (b) Proportion of perfect alignment is evaluated through vg stats.

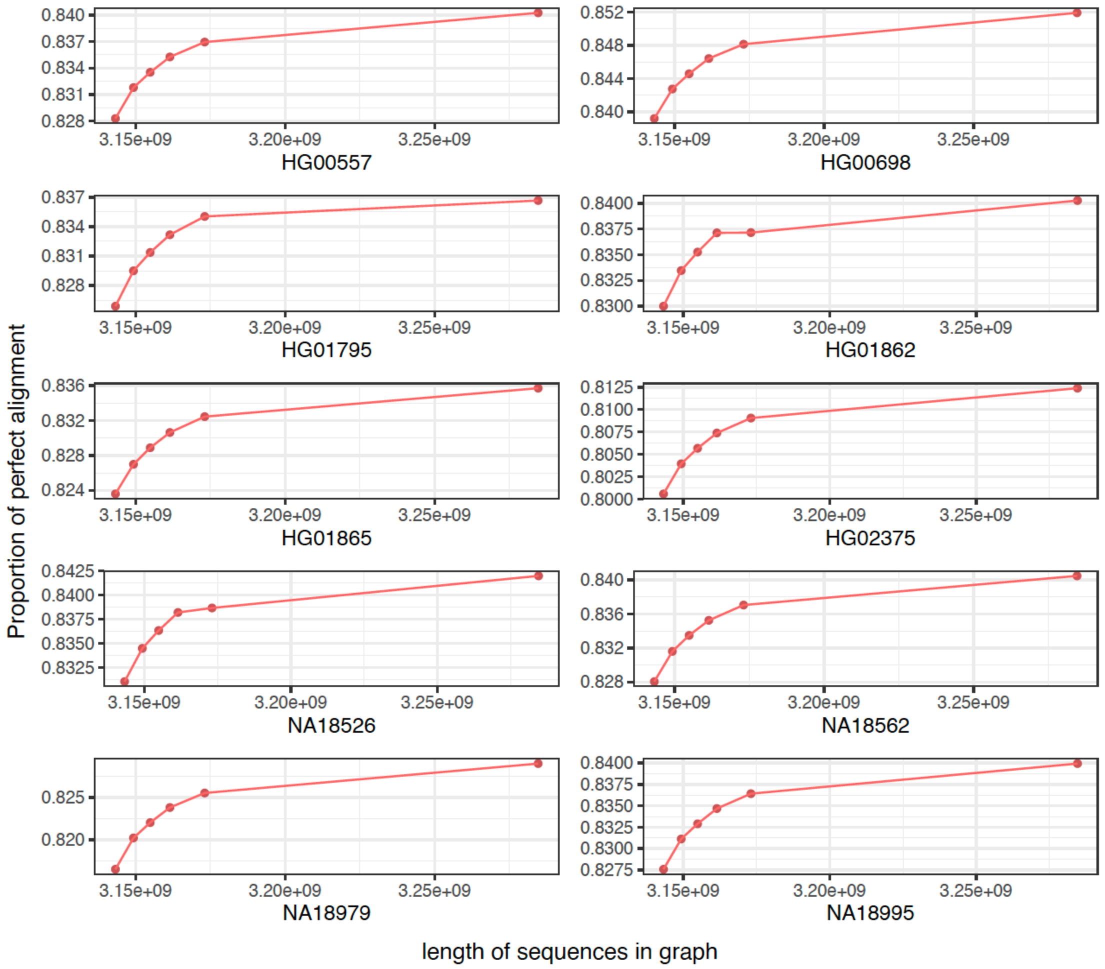

Supplementary Fig. 13 | The correlation between the diversity of graph reference and the proportion of perfect alignment.

The abscissa is the sequence length of graph genome. Larger represents more alternative sequences.

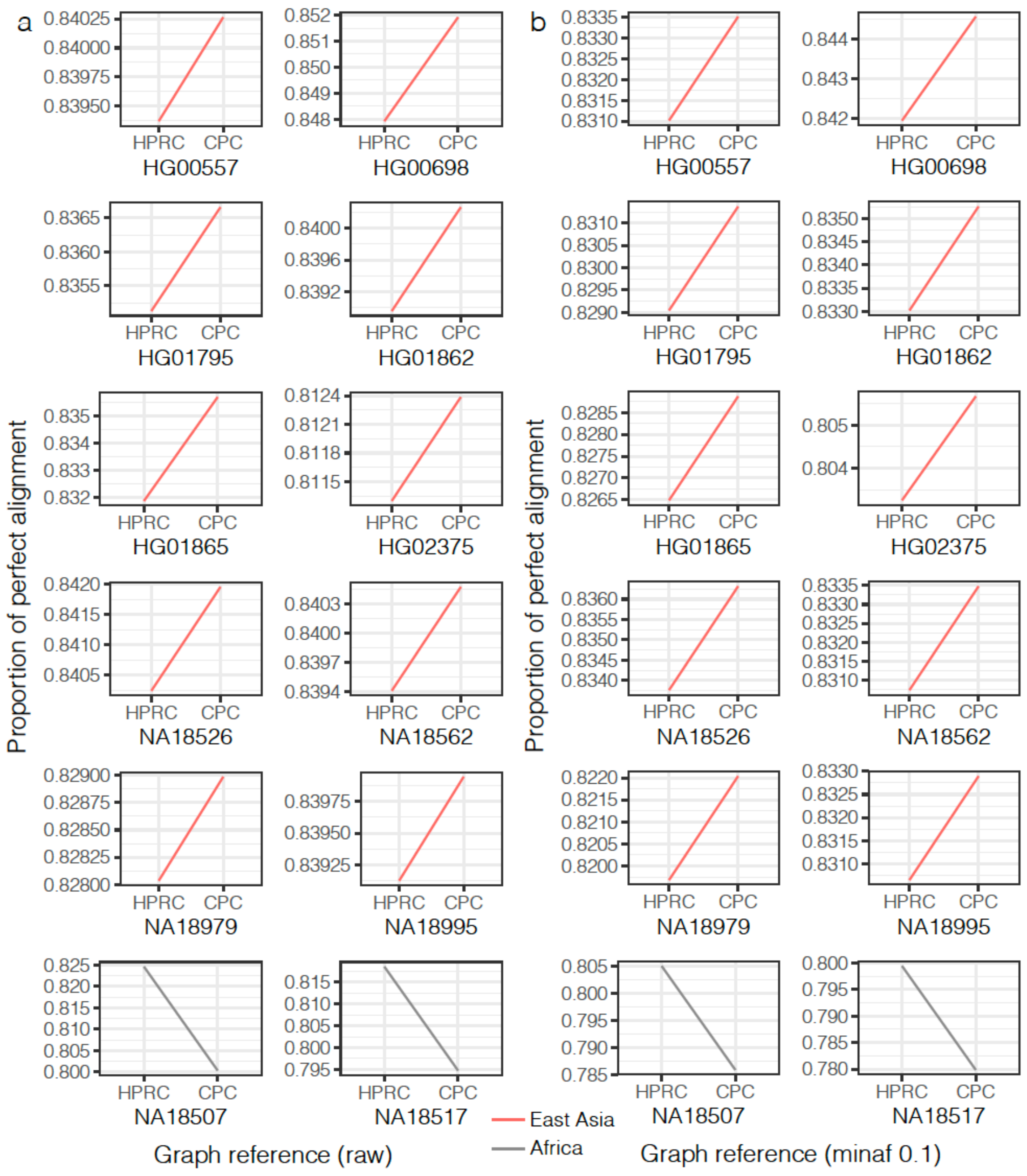

Supplementary Fig. 14 | The difference of perfect alignment ratio between CPC reference and HPRC reference when processing East Asian samples and African samples.

Each figure shows a sample. The red line represents the East Asian sample and the black line represents the African sample. (a) Use raw CPC graph reference and raw HPRC graph reference. (b) Use filtered CPC and HPRC reference. The filtering standard is the minimum frequency of 0.1.

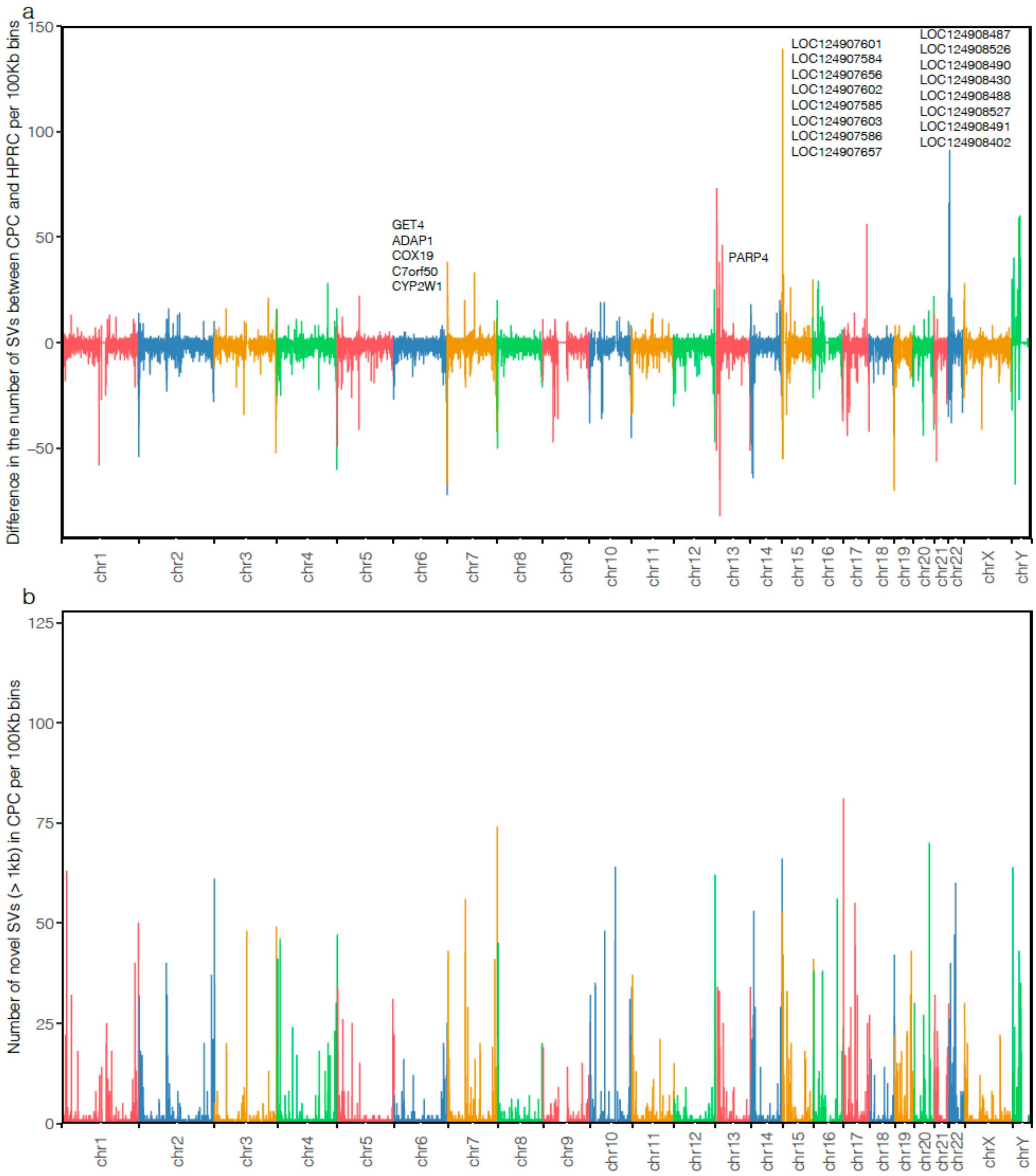

Supplementary Fig. 15 | Comparison of SV distribution between CPC and HPRC reference Pangenomes.

(a) The quantity difference of SV between CPC and HPRC genomes per 100kb bin. (b) The number of CPC-specific SV per 100kb bin.

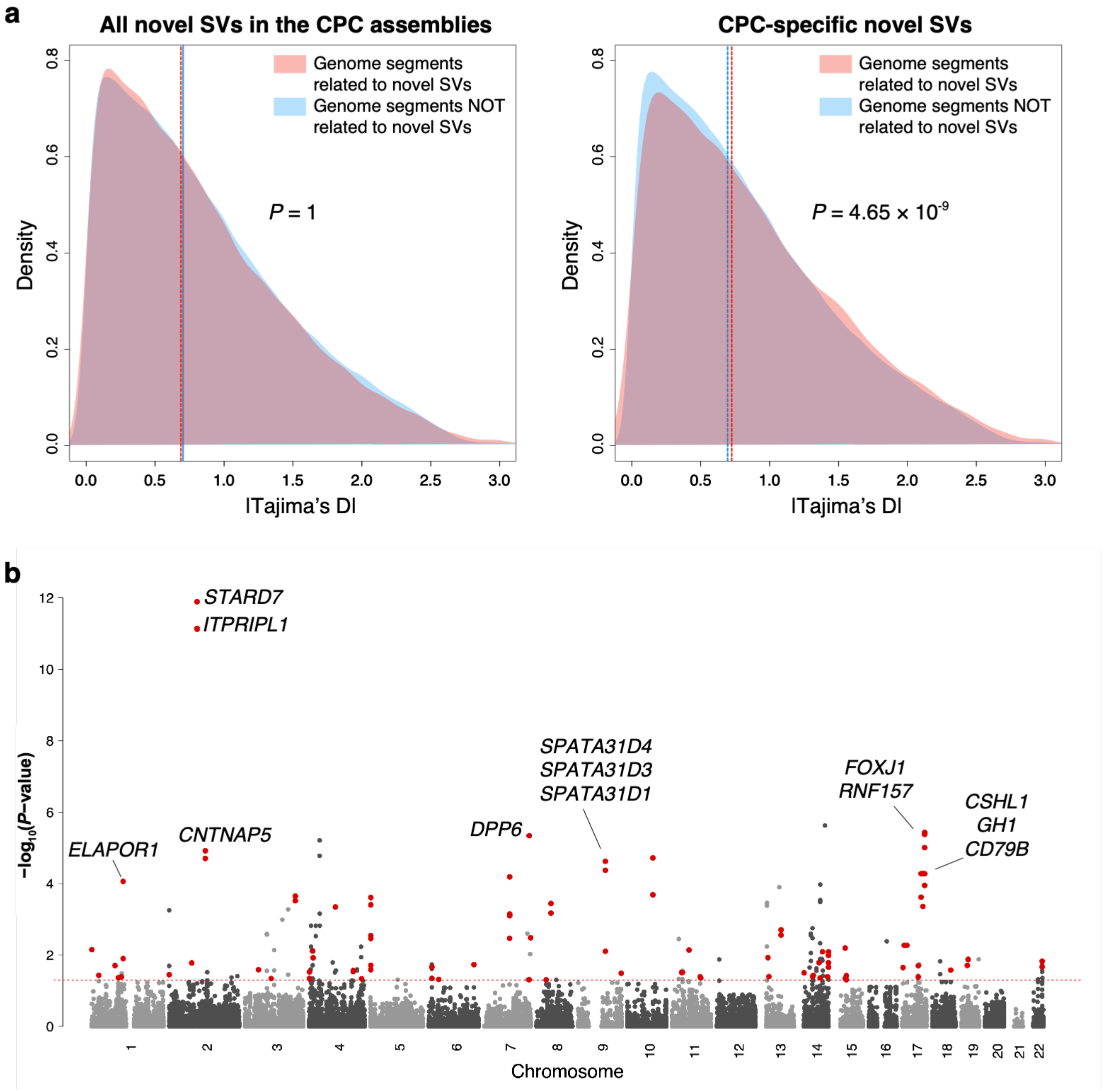

Supplementary Fig. 16 | Tajima's D estimated for the novel SVs identified in the CPC assemblies.

Tajima's D was calculated for the 58 CPC samples (116 haploid assemblies) with a sliding window spanning 20 kb in size, stepping 10 kb. (a) Comparison of the absolute Tajima's D values between the total novel SVs and the rest of the genome, and that between the CPC-specific novel SVs and the rest of the genome. The *P*-values were obtained by one-tailed Wilcoxon's rank-sum test. The vertical lines indicate the median of the |Tajima's D| distributions (red: genome segments overlapping the novel SVs; blue: genome segments do not overlap the novel SVs). (b) A manhatton plot for the |Tajima's D| estimated for the novel SVs. Each dot represents a genome segment, and the red dots highlight the CPC-specific outliers (FDR-adjusted $P < 0.05$).

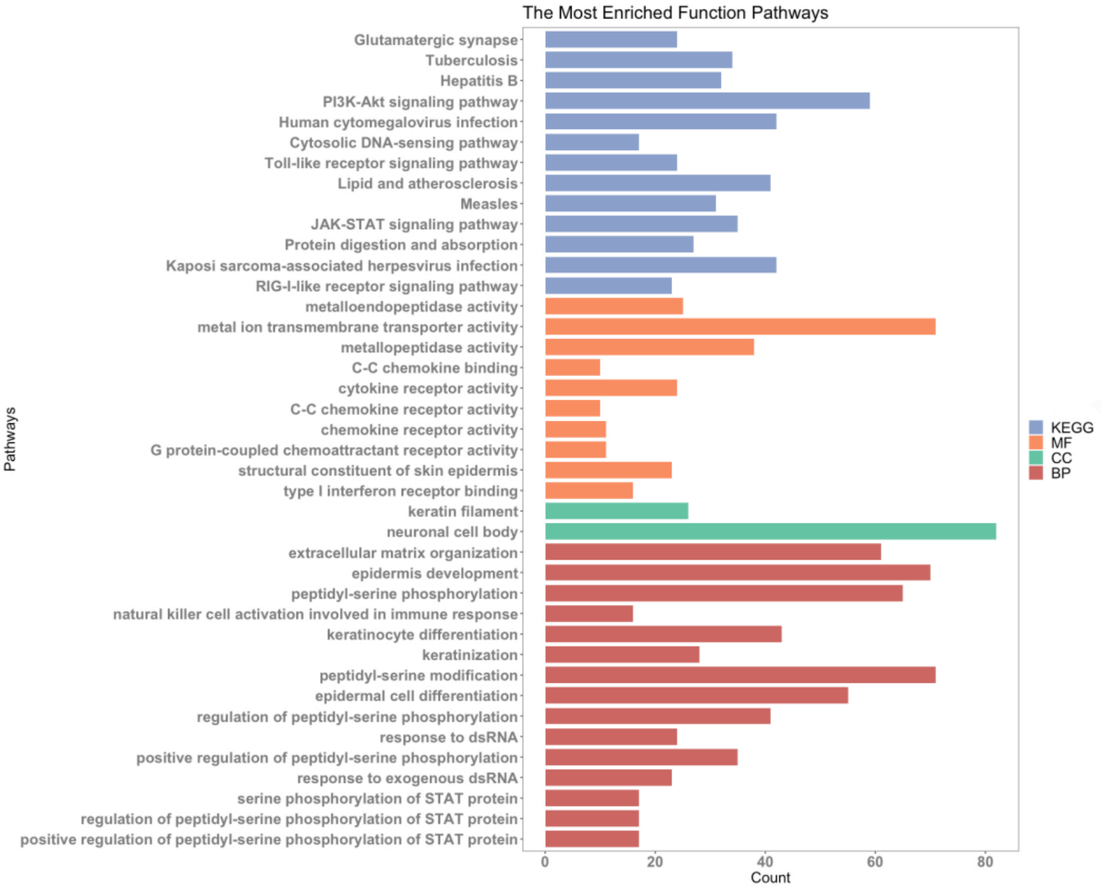

Supplementary Fig. 17 | Functional enrichment of 2617 genes in CDS where CPC-AIS are located and observed in at least 5 samples.

GO and KEGG databases were used here. Differential color means differential database (blue, BP: GO Biological process; orange, CC: GO cellular component; red, MF: GO metabolic function; green, KEGG: KEGG database). The *P*-values of these terms are all less than 0.05, which is obtained by the one-sided version of Fisher's exact test based on the hypergeometric distribution, and is further adjusted for multiple testing using the BH method.

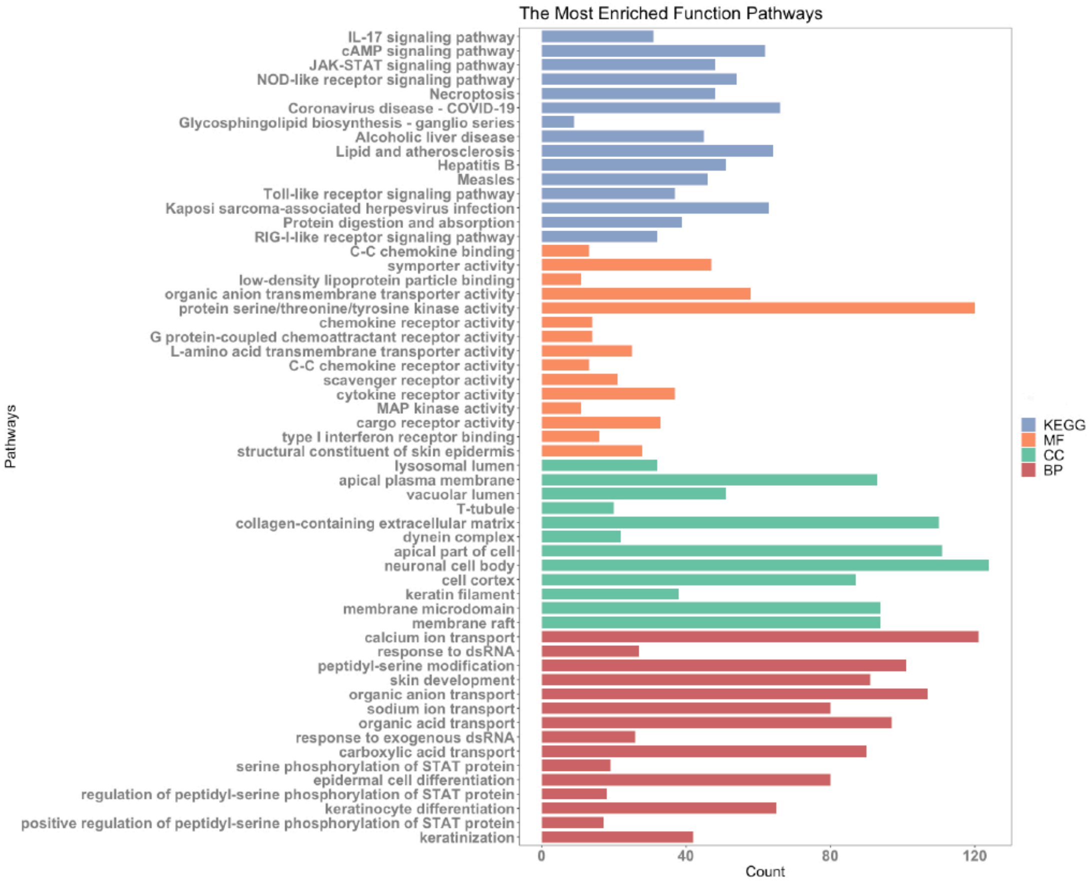

Supplementary Fig. 18 | Functional enrichment of genes in CDS where AIS are located and observed in at least 1 sample.

GO and KEGG databases were used here. Differential color means differential database (blue, BP: GO Biological process; orange, CC: GO cellular component; red, MF: GO metabolic function; green, KEGG: KEGG database). The *P*-values of these terms are all less than 0.05, which is obtained by the one-sided version of Fisher's exact test based on the hypergeometric distribution, and is further adjusted for multiple testing using the BH method.

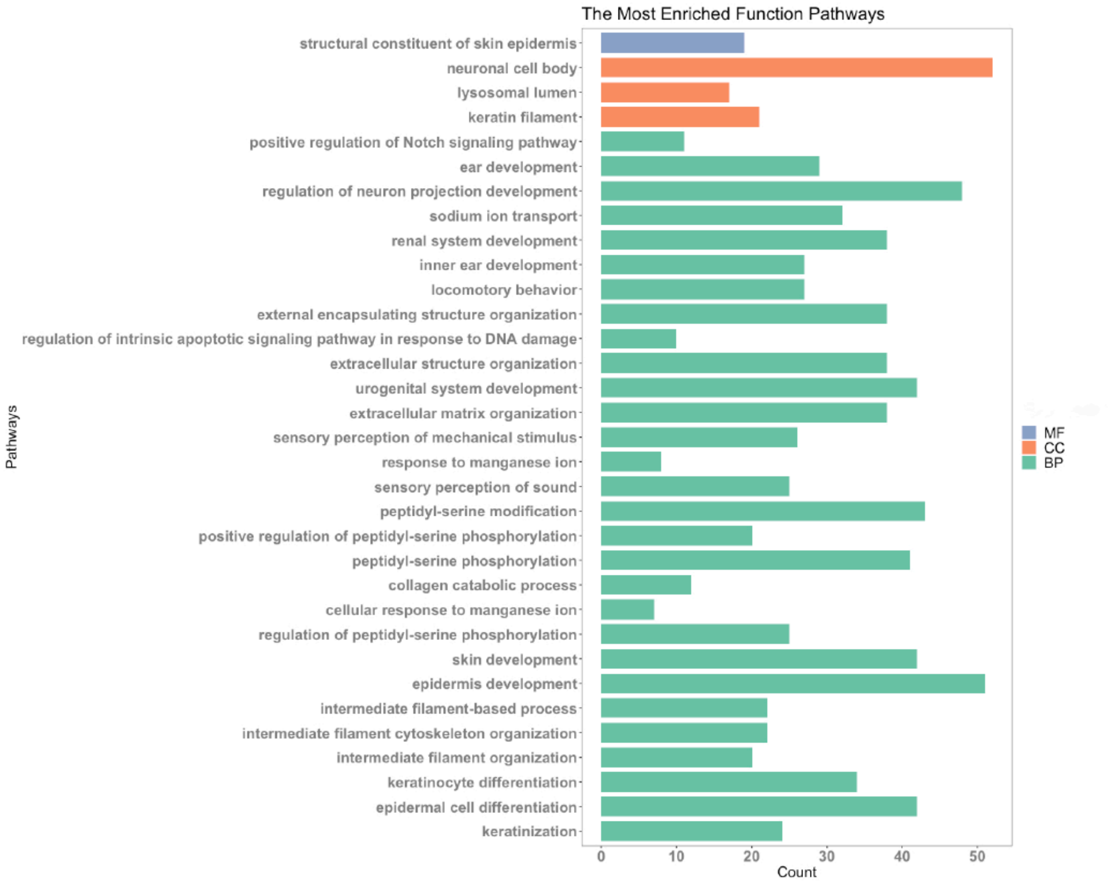

Supplementary Fig. 19 | Functional enrichment of genes in CDS where AIS are located and observed in at least 10 samples.

GO and KEGG databases were used here. Differential color means differential database (blue, BP: GO Biological process; orange, CC: GO cellular component; red, MF: GO metabolic function; green, KEGG: KEGG database). The *P*-values of these terms are all less than 0.05, which is obtained by the one-sided version of Fisher's exact test based on the hypergeometric distribution, and is further adjusted for multiple testing using the BH method.

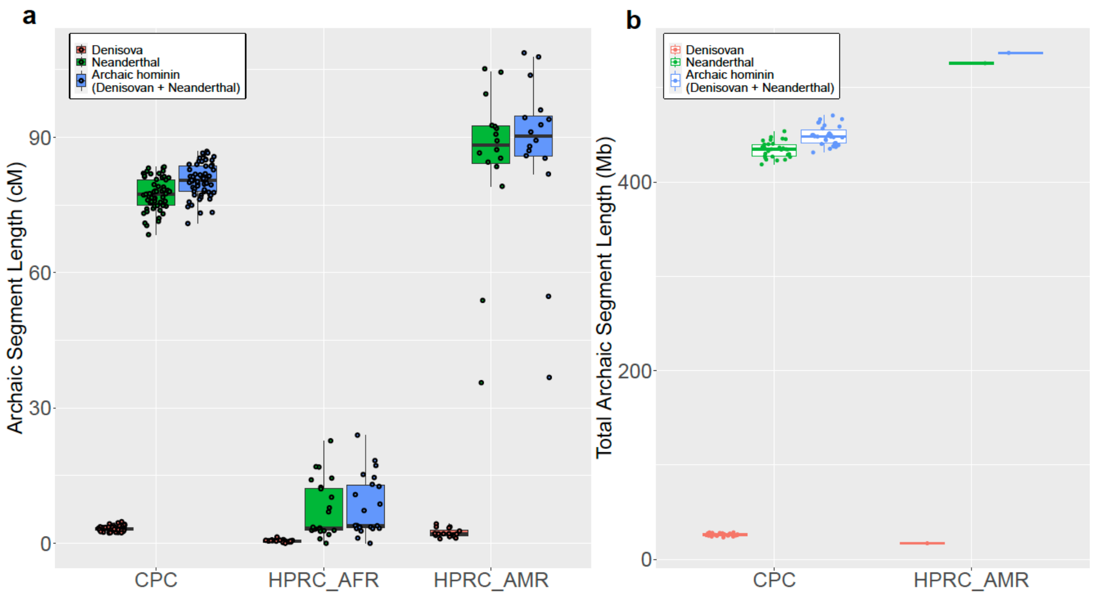

Supplementary Fig. 20 | Total length of the archaic introgression segments detected in CPC compared with that in HPRC.

(a) on the individual level and (b) on the population level given comparable sample size (n = 16,100 replicated sampling for the CPC samples). Each boxplot represents the median (thick black line), upper and lower quartiles (box), and 1.5× interquartile range (whiskers) of the archaic segment length detected (dots).

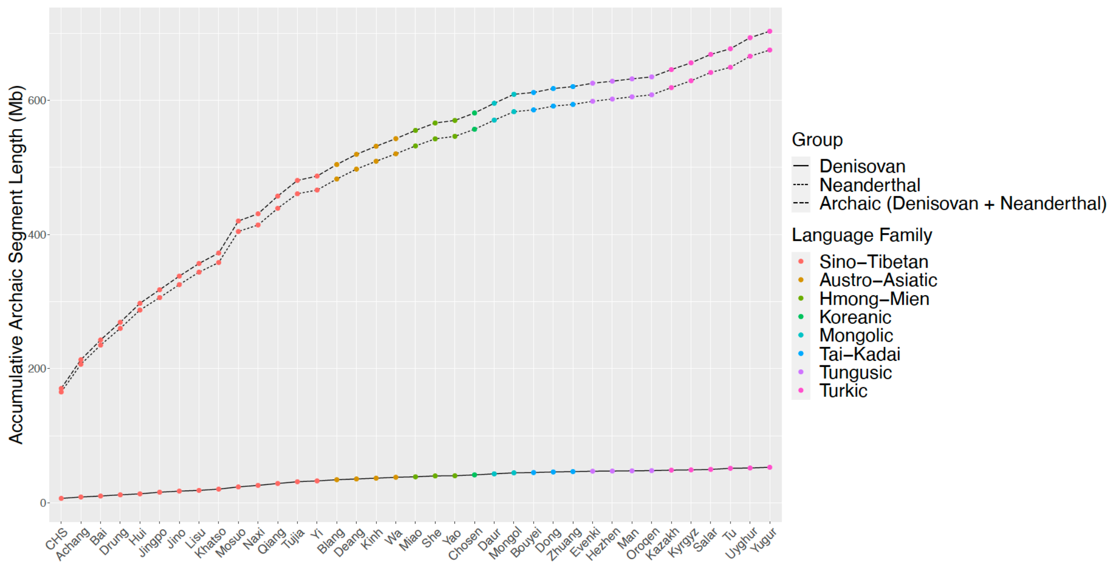

Supplementary Fig. 21 | Accumulative length of the archaic introgression segments in the East Asian populations studied in CPC.

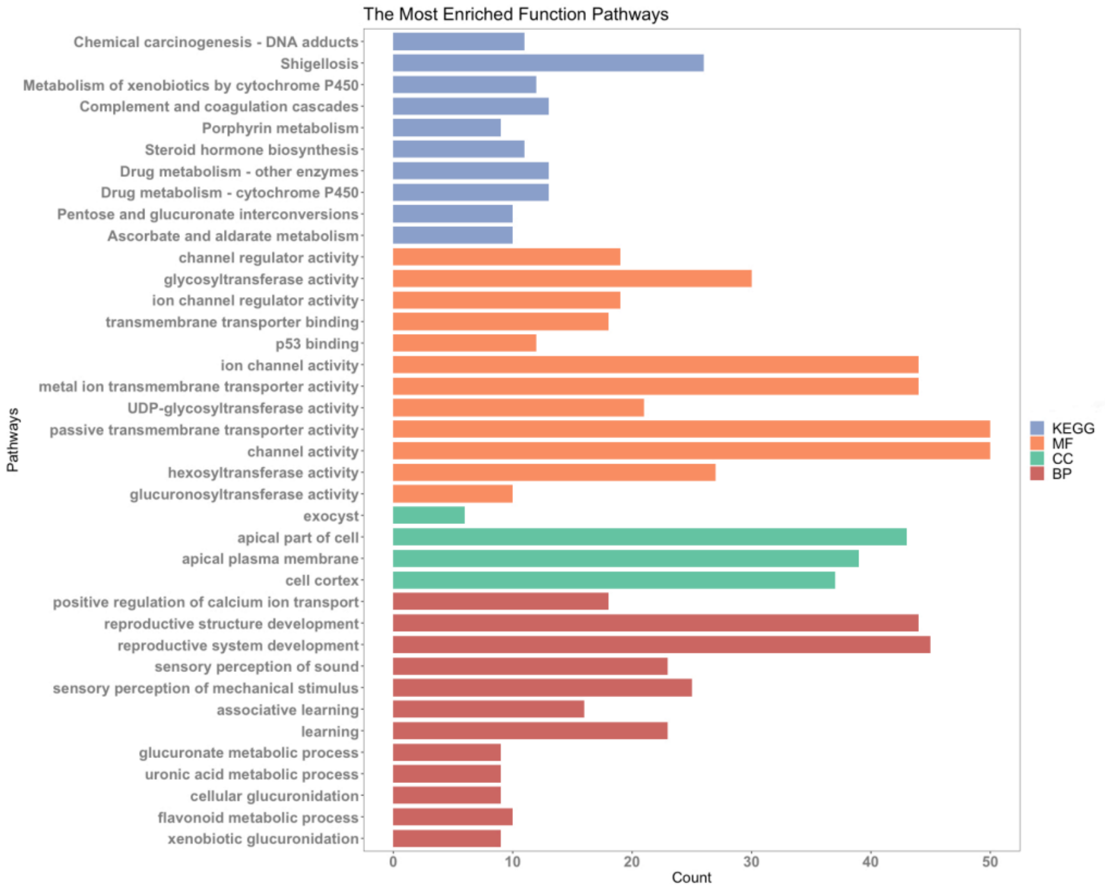

Supplementary Fig. 22 | Functional enrichment of 1211 genes in CDS where CPC-AIS-specific are located.

GO and KEGG databases were used here. Differential color means differential database (blue, BP: GO Biological process; orange, CC: GO cellular component; red, MF: GO metabolic function; green, KEGG: KEGG database). The *P*-values of these terms are all less than 0.05, which is obtained by the one-sided version of Fisher's exact test based on the hypergeometric distribution, and is further adjusted for multiple testing using the BH method.
