# Supplementary material for: A pangenome reference of 36 Chinese populations
Source: Nature. 2023 Jun 14;619(7968):112–21. doi: 10.1038/s41586-023-06173-7 (PMC10322713; doi:10.1038/s41586-023-06173-7)
Supplement: Supplementary file 2 — Reporting Summary [file 41586_2023_6173_MOESM2_ESM.pdf]

nature portfolio

Corresponding author(s): Shuhua Xu

Last updated by author(s): Apr 15, 2023

Reporting Summary

Nature Portfolio wishes to improve the reproducibility of the work that we publish. This form provides structure for consistency and transparency in reporting. For further information on Nature Portfolio policies, see our Editorial Policies and the Editorial Policy Checklist.

Statistics

For all statistical analyses, confirm that the following items are present in the figure legend, table legend, main text, or Methods section.

n/a	Confirmed	
☐	☒	The exact sample size (*n*) for each experimental group/condition, given as a discrete number and unit of measurement
☐	☒	A statement on whether measurements were taken from distinct samples or whether the same sample was measured repeatedly
☐	☒	The statistical test(s) used AND whether they are one- or two-sided<br>*Only common tests should be described solely by name; describe more complex techniques in the Methods section.*
☐	☒	A description of all covariates tested
☐	☒	A description of any assumptions or corrections, such as tests of normality and adjustment for multiple comparisons
☐	☒	A full description of the statistical parameters including central tendency (e.g. means) or other basic estimates (e.g. regression coefficient) AND variation (e.g. standard deviation) or associated estimates of uncertainty (e.g. confidence intervals)
☐	☒	For null hypothesis testing, the test statistic (e.g. *F*, *t*, *r*) with confidence intervals, effect sizes, degrees of freedom and *P* value noted<br>*Give P values as exact values whenever suitable.*
☒	☐	For Bayesian analysis, information on the choice of priors and Markov chain Monte Carlo settings
☒	☐	For hierarchical and complex designs, identification of the appropriate level for tests and full reporting of outcomes
☐	☒	Estimates of effect sizes (e.g. Cohen's *d*, Pearson's *r*), indicating how they were calculated

Our web collection on statistics for biologists contains articles on many of the points above.

Software and code

Policy information about availability of computer code

Data collection	No
Data analysis	The code to reproduce the pangenome from this work can be found on GitHub, https://github.com/Shuhua-Group/Chinese-Pangenome-Consortium-Phase-I. Relevant commands used in other analysis can be found in the methods or supplementary information.<br><br>Other software:<br>SMRTLink v9.0<br>ccs-v6.3.0<br>hifiasm v0.16.1<br>QUAST v5.2.0<br>inspector v1.2<br>RepeatMasker1 v4.1.2-p1<br>RMBLAST v2.10.0<br>Dfam2 v3.3<br>dna-brnn3 v0.1<br>minimap2 v2.24<br>PAV v1.2<br>SV-pop v3.0<br>primatR v0.1.0<br>liftoff v1.6.3

GATK v4.1.7.0
bwa v0.7.17
Minigraph v0.19
dna-brnn v0.1
Cactus v2.1.1
vg v1.42
hal2vg v1.0.17
GFAffix v0.1.3
RIdeogram v0.2.2
gfabase v0.6.0
bandage v0.9
GraphAligner v1.0.16
gggenes v0.3.1
SNPRelate v1.6.4
ADMIXTURE v1.3.0
samtools v1.15.1
clusterProfiler v3.10.1
ArchaicSeekerV2.0
bcftools v1.14
SVision v1.3.7
pheatmap 1.0.12

For manuscripts utilizing custom algorithms or software that are central to the research but not yet described in published literature, software must be made available to editors and reviewers. We strongly encourage code deposition in a community repository (e.g. GitHub). See the Nature Portfolio guidelines for submitting code & software for further information.

Data

Policy information about availability of data

All manuscripts must include a data availability statement. This statement should provide the following information, where applicable:

- Accession codes, unique identifiers, or web links for publicly available datasets
- A description of any restrictions on data availability
- For clinical datasets or third party data, please ensure that the statement adheres to our policy

The release of the CPC Phase I data by this work is permitted by The Ministry of Science and Technology of the People's Republic of China (permission no. 2022BAT2392) at the National Genomics Data Center (https://ngdc.cncb.ac.cn) under the BioProject PRJCA011422. The Pangenome References built based on the CPC core samples and combined with the HPRC samples are freely available at both the CPC website https://pog.fudan.edu.cn/cpc/#/data and GitHub (https://github.com/Shuhua-Group/Chinese-Pangenome-Consortium-Phase-I).

Other dataset:
GRCh38 reference (https://ftp.ncbi.nlm.nih.gov/genomes/all/GCA/000/001/405/GCA_000001405.15_GRCh38/seqs_for_alignment_pipelines.ucsc_ids/GCA_000001405.15_GRCh38_no_alt_plus_hs38d1_analysis_set.fna.gz),
T2T-CHM13 reference (https://s3-us-west-2.amazonaws.com/human-pangenomics/T2T/CHM13/assemblies/GCA_009914755.4/chm13v2.0.fa.gz),
HPRC data (https://github.com/human-pangenomics/hpgp-data),
GENCODE v38 (https://www.gencodegenes.org/human/release_38.html),
GIAB 3.0 (https://ftp-trace.ncbi.nlm.nih.gov/ReferenceSamples/giab/release/genome-stratifications/v3.0/GRCh38/),
gnomAD 1.6 (https://gnomad.broadinstitute.org/downloads),
1000 GP phase3 (https://www.ebi.ac.uk/ena/browser/view/PRJEB31736),
Denisovan (https://www.eva.mpg.de/genetics/genome-projects/denisova),
AltaiNean (https://www.eva.mpg.de/genetics/genome-projects/neandertal),
KEGG (https://www.genome.jp/kegg/),
GO (http://geneontology.org/).

Human research participants

Policy information about studies involving human research participants and Sex and Gender in Research.

Reporting on sex and gender	Gender of each sample is reported in the manuscript. we strived to maintain the balance of gender ratio in the sample selection of each population.
Population characteristics	The full set of CPC Phase I assemblies include 68 samples representing 36 Chinese minority ethnic groups and 8 linguistic groups. Apart from ethnicity and gender, no other Population characteristics are considered.
Recruitment	All 731 randomly collected samples were performed for the whole genome next generation sequencing. To select representative samples for third-generation sequencing, we applied a procedure to quantitatively evaluate the genetic diversity coverage based on PCA results. We selected individuals using a statistic Dd to measure the representation of population samples, it turned out that the selected samples are located close to the center of the cluster of each population on the plot of top two PCs. However, this sample selection method and small sequencing scale may underestimate the true genetic diversity of the population.
Ethics oversight	Informed consent was obtained from all individual participants included in the study. The personal identifiers of all samples, if any existed, were stripped off before sequencing and analysis. All procedures were in accordance with the ethical standards

of the Responsible Committee on Human Experimentation and the 1964 Helsinki Declaration, its later amendments (2000) or comparable ethical standards. The research content and procedures performed in studies involving human participants were approved by the Biomedical Research Ethics Committee of Shanghai Institutes for Biological Sciences (No. ER-SIBS-261408), the Biomedical Research Ethics Committee of Kunming Institute of Zoology, Chinese Academy of Sciences (No. SMKX-20180715-154), the Biomedical Research Ethics Committee of the First Affiliated Hospital of Xi'an Jiaotong University (No. XJTU1AF2021LSK-051).

Note that full information on the approval of the study protocol must also be provided in the manuscript.

Field-specific reporting

Please select the one below that is the best fit for your research. If you are not sure, read the appropriate sections before making your selection.

☒ Life sciences ☐ Behavioural & social sciences ☐ Ecological, evolutionary & environmental sciences

For a reference copy of the document with all sections, see nature.com/documents/nr-reporting-summary-flat.pdf

Life sciences study design

All studies must disclose on these points even when the disclosure is negative.

Sample size	We followed the HPRC to determine the sample size and applied a procedure similar to that of HPRC to select the representative samples of a subpopulation. For the Phase I of CPC, we selected 68 samples from 731 individuals with genomes deep-sequenced using next-generation sequencing.
Data exclusions	We removed 3 samples and 7 samples with relatively low assembly quality from primary assemblies and diploid assemblies, respectively.
Replication	Repetition is mostly at the individual level. For example, 1. 1-3 samples were selected from each population to perform third-generation sequencing, 2. 10 samples were selected to evaluate the power of short sequence mapping.
Randomization	All the samples are randomly collected from natural populations. In the study, only the population and gender of the sample were considered, and no other phenotypic information was involved.
Blinding	At different stages such as sampling, sequencing, and analysis, samples have different ID codes. All the analyses have been applied to all the population groups equally.

Reporting for specific materials, systems and methods

We require information from authors about some types of materials, experimental systems and methods used in many studies. Here, indicate whether each material, system or method listed is relevant to your study. If you are not sure if a list item applies to your research, read the appropriate section before selecting a response.

Materials & experimental systems

n/a	Involved in the study	
☒	☐	Antibodies
☐	☒	Eukaryotic cell lines
☒	☐	Palaeontology and archaeology
☒	☐	Animals and other organisms
☒	☐	Clinical data
☒	☐	Dual use research of concern

Methods

n/a	Involved in the study	
☒	☐	ChIP-seq
☒	☐	Flow cytometry
☒	☐	MRI-based neuroimaging

Eukaryotic cell lines

Policy information about cell lines and Sex and Gender in Research

Cell line source(s)

47 samples are stored in the Immortalize Cell Bank of Chinese Ethnics Groups hosted in the Institute of Medical Biology, CAMS.

Sample Sex

HIFI032682D M
HIFI032585D M
HIFI032069D F
HIFI032373D M

HIFI032487D F
HIFI032018D M
HIFI032306D F
HIFI032668D M
HIFI032698D F
HIFI032292D F
HIFI032462D M
HIFI032473D M
HIFI032510D F
HIFI032450D M
HIFI032706D M
HIFI032007D F
HIFI032513D M
HIFI032685D M
HIFI032440D M
HIFI032429D M
HIFI032731D F
HIFI032289D M
HIFI032167D M
HIFI032164D M
HIFI032607D F
HIFI032604D F
HIFI032161D F
HIFI032422D M
HIFI032501D M
HIFI032693D M
HIFI032335D U*
HIFI032420D M
HIFI032567D F
HIFI032662D M
HIFI032453D M
HIFI032586D F
HIFI032302D F
HIFI032591D M
HIFI032349D M
HIFI032454D F
HIFI032097D F
HIFI032103D M
HIFI032529D F
HIFI032711D M
HIFI032182D M
HIFI032566D M
HIFI032692D F

* Uncertain due to inconsistency of self reported sex and that based on genetic data.

Authentication

The cell line authentication testing was performed by using comparative analysis of genome sequencing data of the cell line and that of blood sample with the same sample ID.

Mycoplasma contamination

All cell lines were tested negative for mycoplasma contamination by polymerase chain reaction-based method and culture assay.

Commonly misidentified lines
(See ICLAC register)

NA.
